# Abstract Supplement Abstracts from IAS 2025, the 13th IAS Conference on HIV Science, 13 – 17 July, Kigali, Rwanda & Virtual

**DOI:** 10.1002/jia2.26518

**Published:** 2025-07-14

## ORAL ABSTRACTS

### OAA0102 HLA‐E restricted HIV‐1 TCR transductants efficiently reduce the size of the HIV reservoir

J.V. Garcia ^1^, H. Yang^2^, A. McMichael^2^, G. Doyon^3^

^1^University of Alabama at Birmingham, Microbiology, Birmingham, United States, ^2^University of Oxford, Dept. of Clinical Medicine, Oxford, United Kingdom, ^3^University of North Carolina, Infection Diseases, Chapel Hill, United States

**Background**: To effectively cure HIV‐1, it is essential to maintain ongoing antiviral T‐cell responses to achieve long‐term suppression or clearance of the virus. This study examines the ability of non‐polymorphic human leukocyte antigen‐E (HLA‐E) restricted T cells to suppress HIV‐1 replication in humanized mice, utilizing a T‐cell receptor (TCR)‐based immunotherapy approach.

**Methods**: Bulk CD8^+^ T cells derived from humanized mouse tissues were transduced with HLA‐E restricted TCRs specific for GAG epitope RMYSPTSIL (RL9) and REV epitope ILVESPAVL (IL9). TCR transductants were enriched and expanded in vitro prior to infusion into autologous humanized mice. To determine if the presence of the HIV‐1‐specific TCRs has an effect on suppressing HIV‐1 virus replication, we infected humanized mice with HIV‐1_JRCSF_, separately humanized mice receiving non‐HIV‐1 TCR transductants were used as controls.

**Results**: Significant higher frequencies of TCR transductants were detected in PB and tissues in mice receiving HIV‐1 TCRs compared to control mice, suggesting HIV‐1 antigen‐specific expansion and long‐term survival of HIV‐1 TCR^+^ cells in vivo. HIV‐1 TCR^+^ transduced cells were detected in all tissues analysed. Importantly, during acute HIV‐1 infection in the absence of antiretroviral therapy (ART), we observed significant reductions in plasma viral load (∼90%, *p* = < 0.04) and in cell‐associated viral RNA from tissues (> 80%, *p* = 0.0016). We then evaluated the efficacy of HLA‐E restricted HIV‐1 TCR transductants to reduce the size of the HIV reservoir in animal where HIV replication had been suppressed by ART. Using intact proviral DNA analysis, we were able to demonstrate a 50% reduction in the levels of intact provirus in tissues (*p* = 0.04).

**Conclusions**: Our findings demonstrate that HLA‐E restricted HIV‐1 TCR transductants are effective in reducing viral replication in vivo. Additionally, we have shown that these transductants efficiently decrease the size of the HIV reservoir. Combined, our results indicate that cell therapy‐based approaches targeting the minimally polymorphic HLA‐E molecules can provide a robust therapeutic effect in vivo, reducing the intact proviral DNA load in HIV‐infected T cells. HLA‐E‐based cell therapy, with its “universal” nature, holds significant potential as a novel approach to curing HIV.

### OAA0103 Association between reservoir size and markers of cardiovascular inflammation in children living with HIV

Y. Fougère^1^, M.T. Hawkes^2^, J. Brophy^3^, S. Read^4^, A. Bitnun^4^, H. Soudeyns^5^, F. Kakkar ^1^, EPIC4 Study Group

^1^University of Montreal, Pediatrics, Montreal, Canada, ^2^University of British Columbia, Pediatrics, Vancouver, Canada, ^3^University of Ottawa, Pediatrics, Ottawa, Canada, ^4^University of Toronto, Pediatrics, Toronto, Canada, ^5^University of Montreal, Microbiology and Immunology, Montreal, Canada

**Background**: There are limited data on long‐term cardiovascular disease risk among children living with perinatal HIV infection (CHIV). Our objective was to assess the association between HIV reservoir size and biomarkers of cardiovascular inflammation among Canadian CHIV.

**Methods**: Sub‐study of the « Early Pediatric Initiation, Canada Child Cure Cohort Study » (EPIC^4^, 2014−2019). In CHIV with sustained viral suppression (SVS) in whom the inducible HIV‐1 reservoir in CD4^+^ T cells was previously measured (prostratin stimulation assay), serum levels of CRP, IL‐1β, IL‐6, IL‐18, VEGF‐1, TNF‐α and IFN‐γ were measured using the Luminex 200^TM^ XMAP multiplex assay at the time of reservoir measurement. Multiple linear regression was used to assess potential associations. To avoid overadjustment, factors predictive of reservoir size (age at treatment initiation, duration of SVS) were not included in the models.

**Results**: Out of 225 EPIC^4^ participants, 121 had measures of inducible HIV‐1 RNA (iRNA) at baseline. Median age was 13 years (range = 6 months−23 years), age at initiation of combination antiretroviral therapy (cART) was 1.6 years (IQR = 4 months−4.7 years) and duration of SVS was 5.6 years (IQR = 2.1–8.3). There was an equal distribution of female and male participants (50%), and 81% had serological evidence of cytomegalovirus (CMV) co‐infection. Median number of copies of iRNA produced by 10^6^ CD4^+^ T cells was 5.8 (IQR = 1.1−57.5). Age at cART initiation, duration of cART and proportion of life with SVS were all predictors of iRNA levels (*p* = 0.004, *p* = 0.02 and *p* = 0.01, respectively). There was a significant association between levels of iRNA and serum levels of CRP (*p* = 0.009), IL‐6 (*p* = 0.04), IL‐1β (*p* = 0.002) and IL‐18 (*p* = 0.014), though no association with IFN‐γ, VEGF‐1 or TNF‐α were observed. These associations all remained significant after adjusting for age and CMV serostatus.

**Conclusions**: Reservoir size as measured by iRNA was strongly associated with biomarkers of cardiovascular inflammation in CHIV. These data reinforce the need for early SVS in CHIV to mitigate long‐term comorbidities, and the potential use of these biomarkers as proxy measures of long‐term cardiovascular disease risk.

### OAA0104 Unique cellular signatures and HIV transcripts identified in CD4 lymphoid T cells—HOPE Act organ transplantation collaboration

M. Tuyishime ^1^, D. Nattere^1^, N. Alavian^2^, S. Carr^3^, J. Granek^4^, T. Schappe^4^, N. Archin^5^, D. Margolis^5^, J. Pollara^1^, G. Ferrari^1^, C. Wolfe^2^

^1^Duke University Medical Center, Surgery, Durham, United States, ^2^Duke University Medical Center, Medicine, Durham, United States, ^3^Duke University Medical Center, Pediatrics, Durham, United States, ^4^Duke University Medical Center, Center for Human Systems Immunology, Durham, United States, ^5^The University of North Carolina, Chapel Hill, Medicine, Chapel Hill, United States

**Background**: The inability of ART to eradicate HIV‐1 infection is primarily due to the establishment of a latent reservoir that is resistant to current therapies. Lymphatic tissues, including lymph nodes (LNs), are the major tissue reservoirs where latent HIV persists in tissue‐resident CD4 T cells. We sought to analyse LN tissue from virologically suppressed people living with HIV (PLWH) to analyse the size of the reservoir and define transcriptional changes and impact of latency reversing agents (LRAs) on the tissue‐resident cells. The results can inform future efforts to target and eliminate HIV harbouring cells.

**Methods**: Through the Duke HOPE Act organ transplantation programme, PLWH were consented to the collection of otherwise discarded deep lymphatic tissue. We collected LNs from 24 PLWH during abdominal or thoracic transplantation. LNs were manually disassociated and washed with media prior to Ficoll separation if cell count was over 50 million. The frequency of latently infected cells was evaluated by quantitative viral outgrowth assay in 6/24 samples. For transcriptional changes following LRA treatment, CD4^+^ T cells were isolated from lymph nodes in 6/24 samples and were stimulated for 24 hours with LRA combinations (VOR+AZD5582, VOR+AZD5582+IL‐15 and VOR+AZD5582+IL‐15+iBet‐151) prior to single‐cell RNA sequencing (scRNAseq).

**Results**: Using scRNA analysis of a total 94,716 cells, we identified 57 cells with HIV transcripts most of which mapped to viral LTR. The majority of the HIV‐harbouring cells were identified in the VOR+AZD5582‐treated sample. Treatment with LRAs induces upregulation of transcripts related to activation and regulation, protein and lipid synthesis and post‐translational modifications, trafficking to cell membrane. Addition of other LRAs to VOR+AZD5582 combination did not produce significant changes in transcriptome analysis. Interestingly, LRA treatment downregulated TcR transcripts in CD4 cells affecting the ability to identify unique clonotypes of latently infected reactivated cells.

**Conclusions**: Lymph nodes obtained from PLWH undergoing solid organ transplantation represent a high yield source for deep lymphatic tissue. We identified LRA‐induced HIV reactivation and defined effects of LRA combinations on transcriptomic profiles of tissue‐resident CD4 T cells. This data can be used to evaluate future LRAs and assess their combination efficacy and their ability to unmask the lymphatic reservoir.

### OAA0105 The impact of acute stress on the HIV reservoir: a prospective interventional trial and analysis in vitro of stress compounds and latency reversal

J. Stern^1,2^, R.A. Shepherd ^1^, M. Roche^1,3^, W. Hartogensis^4^, P. Moran^4^, L. Cockerham^5^, A. Rhodes^1^, P.U. Cameron^1^, J.J. Chang^1^, N. Kumar^6^, W.B. Mendes^7^, S.G. Deeks^6^, F.M. Hecht^4,6^, S.R. Lewin^1,8,9^

^1^University of Melbourne/Doherty Institute, Department of Infectious Disease, Melbourne, Australia, ^2^University of Melbourne/Doherty Institute, Department of Microbiology and Immunology, Melbourne, Australia, ^3^Royal Melbourne Institute of Technology (RMIT), School of Health and Biomedical Sciences, Melbourne, Australia, ^4^University of California San Francisco, Osher Center for Integrative Health, San Francisco, United States, ^5^Medical College of Wisconsin, Division of Infectious Diseases, Milwaukee, United States, ^6^University of California San Francisco, Division of HIV, Infectious Diseases and Global Medicine, San Francisco, United States, ^7^Yale University, Department of Psychology, New Haven, United States, ^8^Monash University/Alfred Hospital, Infectious Diseases, Melbourne, Australia, ^9^Victorian Infectious Disease Service, Royal Melbourne Hospital at the Doherty Institute, Melbourne, Australia

**Background**: The persistence of latently infected CD4^+^ T cells is the major barrier to cure of people with HIV (PWH) on antiretroviral therapy (ART). While most latently infected cells are transcriptionally silent, some express low levels of cell‐associated (CA) HIV RNA. In this prospective controlled interventional study, we tested the hypothesis that acute psychological stress could drive HIV transcription in PWH on ART.

**Methods**: PWH on suppressive ART underwent the Trier Social Stress Test (TSST) and comparisons were made to a similar period of time without an intervention (control). After 10, 30 and 65 minutes of the acute stressor, we quantified: the stress markers salivary cortisol, pre‐ejection period, cardiac output and respiratory sinus arrhythmia; CA HIV RNA and HIV DNA in CD4^+^ T cells by qPCR; and markers of immune activation and exhaustion via flow cytometry. The direct effect of stress‐associated compounds (glucocorticoid agonists, hydrocortisone and dexamethasone; catecholamines, epinephrine and norepinephrine; the protein kinase A agonist, forskolin; the pituitary hormone, prolactin; and triiodothyronine [T3]) on HIV transcription in the latently infected cell lines J‐Lat A2 and TZM‐bl was measured by GFP and luciferase expression, respectively.

**Results**: Physiological markers of acute psychological stress including pre‐ejection period and cardiac output changed in all participants, as anticipated. Compared to the control day, at the 65‐minute time point, the TSST did not affect HIV DNA levels (*p* = 0.12) but led to a 1.57‐fold increase in CA HIV RNA (*p* = 0.04) which was correlated with pre‐ejection period (rho = −0.59, *p* = 0.002) and cardiac output (rho = 0.6, *p* = 0.003). CA US HIV RNA was positively correlated with CD38^+^HLADR^−^ CD4^+^ T‐cell frequencies (rho = 0.52, *p* = 0.048). HIV DNA was negatively correlated with the proportion of CD8^+^ T cells expressing CD69 (rho = −0.52, *p* = 0.05). Stress‐associated compounds did not upregulate HIV expression in latently infected cell lines.

**Conclusions**: In PWH on ART, HIV transcription increases shortly following acute stress. There was no direct effect of stress compounds on the HIV long terminal repeat. Changes in the half‐life of activated CD4^+^ T cells in circulation following stress could potentially contribute to the increase in CA HIV RNA. Our findings have implications on the importance of stress and the design of cure strategies for PWH.

### OAA0202 Altered viral rebound dynamics in chronically treated people with HIV given long‐acting broadly neutralizing antibodies and N‐803

M. Caskey ^1^, K. Millard^1^, M. Turroja^1^, D. Dong^1^, A. Nicola^1^, M. Fumagalli^1^, G. Breton^1^, K. Seaton^2^, J. Berardi^3^, E. Logue^4^, J.T. Safrit^5^, G. Tomaras^2^, R.B. Jones^6^, M.J. Glesby^6^, T. Wilkin^7^, P. Tebas^4^, M. Nussenzweig^1^

^1^The Rockefeller University, New York, United States, ^2^Duke University, Durham, United States, ^3^Rutgers New Jersey Medical School, Newark, United States, ^4^University of Pennsylvania, Philadelphia, United States, ^5^ImmunityBio, Inc., Culver City, United States, ^6^Weill Cornell Medicine, New York, United States, ^7^University of California San Diego, San Diego, United States

**Background**: Broadly neutralizing antibodies (bNAbs) are promising therapies for HIV. Clinical studies have suggested that bNAbs administered during analytical treatment interruption (ATI) or at ART initiation may affect reservoir dynamics and enhance autologous immune responses, with some participants achieving long‐term control. The interleukin‐15 superagonist, N‐803, enhances NK and cytotoxic T‐cell activity. In SHIV‐infected macaques, two bNAbs with N‐803 led to sustained virologic control. The effects of this combination in humans are not known. We report preliminary results of the antiviral activity of the combination of two long‐acting bNAbs and N‐803 in people with HIV (PWH) during ATI.

**Methods**: This open‐label, single‐arm study enrolled chronically treated PWH to receive single infusions of 3BNC117‐LS (30 mg/kg) and 10‐1074‐LS (10 mg/kg) followed by up to eight subcutaneous injections of N‐803 (6 mcg/kg) 3 weeks apart. Participants were not pre‐selected for bNAb sensitivity. They underwent ATI 2 days after bNAb administration. Participants resumed ART after four consecutive HIV‐1 RNA > 1000 copies/ml. Study objectives included safety, viral suppression through 24 weeks and sustained virologic control. All participants reached the first primary virologic endpoint and long‐term follow up is ongoing.

**Results**: Twenty‐eight participants enrolled in the study; three discontinued before viral rebound (weeks 8, 10 and 10). Most reported adverse events were grade 1 or 2 injection site reactions following N‐803. By week 24, 15 of 25 participants (60%; 95% CI: 40.8%–79.2%) remained off ART. Interestingly, 4 of these 15 participants experienced long periods of low‐level viraemia, two had low‐level viraemia followed by ongoing suppression and two continued to maintain suppression for 72 weeks. In contrast, individuals with early rebound typically resumed ART an average of 3 weeks after the initial return of viraemia. At 24 weeks, the average concentrations of 3BNC117‐LS and 10‐1074‐LS were 40 and 36 mcg/ml, respectively. Reservoir and immunologic analyses are ongoing.

**Conclusions**: In this ongoing study, we found that 15 of 25 chronically treated PWH receiving a combination of bNAbs and N‐803 controlled viraemia for at least 6 months. Of these, 53% experienced prolonged low levels of or no viral rebound.

### OAA0203 Complementary bnAbs: CD4 binding site and interface bnAbs demonstrate bidirectional phenotypic antagonism

R. Lynch^1^, H. Garg^2^, M. Kelly^1^, A. Mcfarland^2^, T. DeStefanis^1^, W. Jin^3^, R. Krause^2^, H. Schrader^2^, J. Best^1^, S. Docken^3^, D. Cromer^3^, M. Davenport^3^, K. Bar ^2^

^1^George Washington University, Microbiology, Washington, United States, ^2^University of Pennsylvania, Medicine, Philadelphia, United States, ^3^University of New South Wales, Kirby Institute, Sydney, Australia

**Background**: Broadly neutralizing antibodies (bnAbs) show promise in HIV cure and prevention, but their efficacy is limited by pre‐existing or emergent resistance. Current best practice calls for combination therapy, using bnAbs from distinct classes to maximize breadth and potency. When virus populations have resistance to one or more bnAbs, however, bnAbs targeting unrelated epitopes act as monotherapy and viruses readily escape. Akin to the bidirectional phenotypic antagonism observed with early antiretroviral therapy, we aim to identify “complementary bnAbs” with inverse susceptibility patterns due to conformationally linked epitopes such that evolving resistance to one would lead to enhanced susceptibility to the other.

**Methods**: We sequenced plasma virus envs by single genome sequencing from 42 participants of treatment interruption and viraemia clinical trials of CD4bs bnAbs. We performed phylogenetic analyses to identify 56 envs, representing the consensus of rebound lineages or replicating virus populations. The 56 Envs were tested for neutralization sensitivity to a panel of 20 bnAbs (CD4bs, V2, V3, interface, MPER) in the TZM.bl assay. Env‐antibody pair IC50s were compared via Spearman correlation, with correction for multiplicity requiring a *p* < 0.00026.

**Results**: A total of 1120 Env‐Antibody pairs were tested for bnAb neutralization sensitivity, generating susceptibility patterns similar to published Env panels. IC50s of all 56 Envs were compared between bnAbs, identifying many statistically significant direct correlations (i.e. similar resistance patterns), but just two statistically significant inverse relationships: CD4bs bnAb N49.P9.6FR and interface bnAb 8ANC195 (Spearman *R* = −0.53, *p* = 2.7e‐05); and CD4bs bnAb VRC01.23 and 8ANC195 (*R* = −0.51, *p* = 6.9e‐05). Modelling of CD4bs and interface epitopes reveals substantial epitope overlap. Structure‐function analysis of Envs sampled in viraemic study participants pre‐ and post‐CD4bs bnAb treatment revealed mutations within epitope overlap region (within V5) that confer increased resistance to CD4bs bnAbs and increased sensitivity to 8ANC195, without impacting MPER, V3 or V2 bnAbs.

**Conclusions**: Leveraging authentic HIV‐1 Envs sampled from participants in bnAb clinical trials, we identified highly statistically significant inverse bnAb susceptibility patterns between interface bnAb 8ANC195 and two CD4bs bnAbs: N49.P9.6FR and VRC01.23, putatively related to epitope overlap. Ongoing mechanistic and in vivo studies aim to determine if combination use 8ANC195 and N49/VRC01.23 will complementarily prevent emergent resistance.

### OAA0204 CAR‐T cell‐secreted bNAbs mediate Fc‐effector functions and reduce viral load in humanized mouse model of HIV infection

Z. Stylianidou ^1^, S. Gerlo^1^, M. Wejda^1^, E. Burg^1^, E. De Smet^1^, Y. Noppe^1^, L. Vandekerckhove^1^, W. Witkowski^1^

^1^HIV Cure Research Center, Ghent University, Faculty of Medicine and Health Sciences, Ghent, Belgium

**Background**: Combining cell and gene therapies with broadly neutralizing antibody (bNAb) administration to achieve a functional HIV cure is gathering interest. Here, we explored the concept of Hybrid CAR T cells, combining the cytotoxic effect of a CAR with the antiviral properties of bNAbs. The Hybrid CAR T cells are engineered to secrete bNAbs with an Fc‐IgG1 domain, which trigger secondary immune effector functions such as antibody‐dependent cellular cytotoxicity (ADCC) and phagocytosis (ADCP) on top of cell‐free HIV neutralization.

**Methods**: We engineered a bicistronic lentiviral vector encoding a third‐generation CD4‐based CAR and the 3BNC117 bNAb. Hybrid CAR CD8^+^ T cells were generated from HIV‐negative donors and assessed for their cytotoxic activity against autologous HIV‐infected CD4^+^ T cells, in real‐time fluorescent imaging‐ and flow cytometry‐based assays. The quantity and HIV neutralization capacity of secreted 3BNC117 was tested by ELISA and TZM‐bl assays, respectively. Evaluation of 3BNC117‐Fc‐mediated functions was performed by antibody‐dependent NK cell degranulation assay (ADNKDA) and by an in‐house developed ADCP assay quantifying phagocytosis of HIV Env‐expressing target cells by primary macrophages with real‐time fluorescent imaging. In vivo efficacy of Hybrid CAR T cells was studied in PBMC‐humanized NSG‐SGM3 mice infused with HIV‐infected CD4^+^ T cells and autologous CD8^+^ Hybrid CAR T cells.

**Results**: In vitro data demonstrated that upon coculture with autologous, HIV‐infected T cells, anti‐HIV Hybrid CAR T cells suppressed HIV replication to the same extent as the CD4 CAR control, while simultaneously secreting 3BNC117 (≈40 ng/ml) capable of neutralizing cell‐free HIV. The secreted antibody induced NK cell degranulation in the presence of recombinant HIV gp120 and mediated phagocytosis of Env‐expressing cells by primary macrophages. In vivo, Hybrid CAR T‐cell‐treated mice demonstrated 6.7‐fold lower median plasma HIV levels as compared to non‐transduced CD8‐treated mice (NTD) control.

**Conclusions**: We successfully engineered Hybrid CAR T cells that secrete bNAbs, exert cytotoxic killing against HIV‐infected cells, neutralize cell‐free HIV and mediate Fc‐dependent functions in vitro. The anti‐HIV activity of Hybrid CAR T cells was confirmed in vivo. Combining CAR T cells and bNAbs, the Hybrid CAR concept harnesses multiple arms of the immune system and, therefore, holds a strong potential for a functional HIV cure.

### OAA0205 HIV‐specific T‐cell responses in suppressed people with HIV‐1 receiving lenacapavir, teropavimab and zinlirvimab

J. Eron ^1^, Y. Cai^2^, H. Takata^3^, A.‐M. Ferreira^2^, J. Pacheco Mendez^3^, S. Nathanson^3^, S.J. Little^4^, J.J. Wallin^2^, S.E. Collins^2^, A. Okoye^3^, L. Trautmann^5^

^1^University of North Carolina, Chapel Hill, NC, United States, ^2^Gilead Sciences, Inc., Foster City, CA, United States, ^3^Vaccine and Gene Therapy Institute, Oregon Health and Science University, Portland, OR, United States, ^4^University of California, San Diego, CA, United States, ^5^Henry M. Jackson Foundation for the Advancement of Military Medicine, Bethesda, MD, United States

**Background**: HIV‐specific T‐cell responses are detectable in virologically suppressed (VS) people with HIV‐1 (PWH) on antiretroviral therapy (ART). Small increases in these responses were observed when the broadly neutralizing antibodies (bNAbs) 3BNC117 and 10‐1074 were dosed during analytical treatment interruption or at ART initiation. In a Phase 1b study (NCT04811040), VS PWH who switched to long‐acting lenacapavir (LEN; HIV‐1 capsid inhibitor) plus the bNAbs teropavimab (TAB) and zinlirvimab (ZAB) maintained virologic suppression for 6 months. We report HIV‐specific T‐cell responses in PWH receiving LEN+TAB+ZAB in this study.

**Methods**: VS PWH on ART for ≥18 months, with CD4^+^ T cells ≥500 cells/µl and high susceptibility to TAB (*n* = 5), ZAB (*n* = 5) or both (*n* = 20) (IC_90_ ≤2 µg/ml [Monogram PhenoSense mAb assay]) discontinued ART and received subcutaneous LEN 927 mg (with oral loading) plus intravenous TAB 30 mg/kg and ZAB 10 or 30 mg/kg. Blood specimens were collected at baseline and weeks 4, 26, 30, 38 and 52. Peripheral blood mononuclear cells were isolated and stimulated in vitro with overlapping 15‐mer peptide pools spanning Clade B HIV‐1 consensus sequences, followed by intracellular cytokine staining and flow cytometry to measure HIV‐specific T‐cell frequencies. Changes from baseline were assessed using Kruskal‐Wallis tests.

**Results**: We observed no significant changes in HIV‐specific IFN‐γ^+^CD8^+^ or IFN‐γ^+^CD4^+^ T cells between baseline and any subsequent time points, with/without stratification by bNAb susceptibility or ZAB dose (Figure 1). No durable changes in HIV‐specific IFN‐γ^+^CD8^+^ or IFN‐γ^+^CD4^+^ T cells were observed in two participants with transient low‐level plasma viraemia (50–200 copies/ml) during follow‐up.

**Figure 1:**
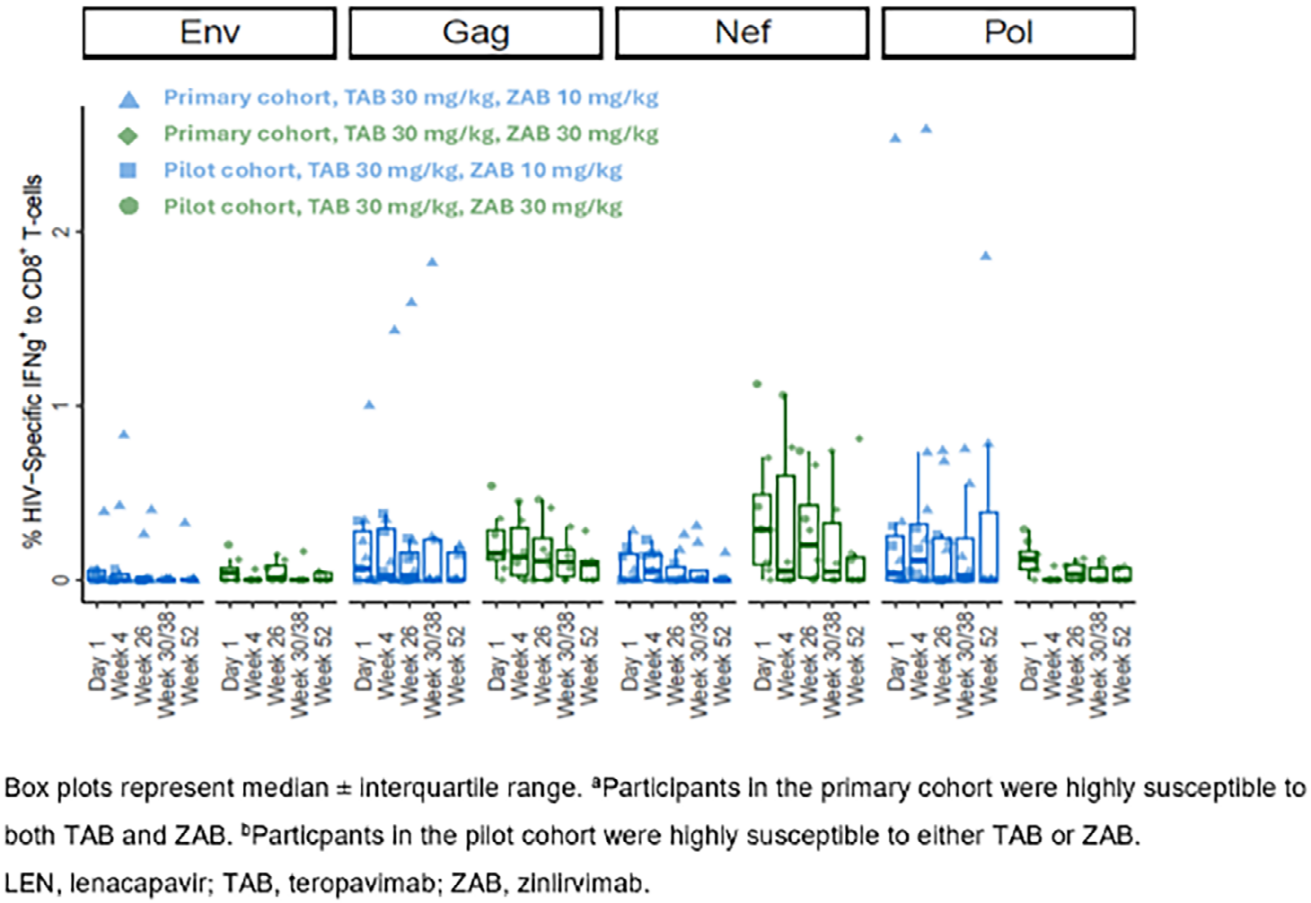
**OAA0205: Treatment With LEN+TAB+ZAB Did Not Impact HIV‐specific IFN‐y^+^CD8^+^ T‐Cells**.

**Conclusions**: Lack of increase from baseline in HIV‐specific T‐cell response following LEN+TAB+ZAB treatment in VS PWH suggests virologic suppression by LEN+TAB+ZAB did not increase viral antigen expression, which may have limited expansion of HIV‐specific T cells. This has implications for HIV‐1 cure studies if greater antigen exposure is required for increased HIV‐specific T‐cell responses after bNAb administration.

### OAA0302 Protection from pathogenic SHIV by T cell‐based vaccine targeting highly networked SIV epitopes

A. Alrubayyi ^1^, M. Getz^1^, M. Melo^2^, H. Huang^1^, S. Bhattacharyya^1^, B. Goldberg^1^, L. Rodriguez^2^, A. Nathan^1^, E.Y. Jeon^1^, R. Yim^1^, L. Maiorino^3^, D. Lim^4^, D.G. Carnathan^4^, A.V. Nacimento^4^, G. Silvestri^4^, D. Irvine^2^, G.D. Gaiha^1,5^

^1^Ragon Institute of Mass General, MIT and Harvard, Cambridge, United States, ^2^Scripps Research, La Jolla, United States, ^3^Massachusetts Institute of Technology, Koch Institute for Integrative Cancer Research, Cambridge, United States, ^4^Emory University, Emory National Primate Research Center, Atlanta, United States, ^5^Massachusetts General Hospital, Division of Gastroenterology, Boston, United States

**Background**: Functional CD8^+^ T‐cell responses targeting highly networked HIV epitopes (i.e. those derived from structurally constrained regions of the viral proteome and involved in important noncovalent interactions) have been strongly associated with HIV control. Based on this work, a highly networked HIV T‐cell vaccine is moving towards clinical evaluation. In this study, we evaluated whether a T cell‐based vaccine targeting highly networked simian immunodeficiency virus (SIV) epitopes could confer protection in the well‐established non‐human primate (NHP) model following simian‐human immunodeficiency virus (SHIV) challenge.

**Methods**: A replication‐incompetent chimpanzee adenovirus serotype 68 (ChAd68) vector and LNP‐mRNA vaccines encoding six highly networked epitopes from the SIV proteome (identified using structure‐based network analysis) were generated. Twenty Indian rhesus macaques (2 females and 18 males) aged between 4 and 5 years that express Mamu A*01 or A*02 were selected. RMs (10 per group) received either PBS control or ChAd68 vector prime (2.5×10^10^ viral particles) followed by two doses of LNP‐mRNA (100 µg) at weeks 0, 4 and 12. At week 16, animals were administered up to 10 weekly intrarectal challenges with low‐dose SHIV BG505.N332 until infected. Vaccine‐induced T‐cell responses were assessed by IFN‐γ ELISpot, intracellular cytokine staining and 6‐day CFSE‐based proliferation assay following stimulation with either individuals or a pool of vaccine‐incorporated SIV epitopes.

**Results**: Vaccination with ChAd68/LNP‐mRNA vaccines encoding highly networked SIV epitopes elicited detectable SIV‐specific CD8^+^ T‐cell responses in an appropriate Mamu‐restricted manner and highly proliferative responses in a subset of animals (*n* = 5; > 5% of CFSE^low^CD8^+^ T cells). Individual responses were enriched for those targeting Mamu A*01 CM9 (Gag) following ChAd68 prime and became additionally enriched for Mamu A*01 CL9 (Env) responses following LNP‐mRNA boost. Importantly, after exposure to the pathogenic intrarectal SHIV challenge, the acquisition was delayed in vaccinated animals relative to controls, with the five animals with highly proliferative networked CD8^+^ T‐cell responses exhibiting significant protection from acquisition (*p* = 0.0048).

**Conclusions**: A ChAd68/mRNA vaccine encoding highly networked epitopes is immunogenic in NHP and resulted in delayed acquisition from SHIV challenge. These data serve as a basis for continued investigation of highly networked CD8^+^ T‐cell vaccines currently advancing towards a human clinical trial evaluation.

### OAA0303 HVTN 307: phase 1 clinical trial of a ferritin nanoparticle preventive HIV vaccine with different adjuvants to induce HIV‐1 Env V3‐glycan‐specific broadly neutralizing antibody precursors

A.E. Shapiro ^1,2^, C.F. Kelley^3^, K.O. Saunders^4^, B.F. Haynes^4^, R. Parks^4^, M. Barr^4^, D. Montefiori^4^, A. Eaton^4^, W.O. Hahn^2^, O. Hyrien^2^, S. Regenold^5^, L.W.P. Church^2^, HVTN 307 Study Team

^1^University of Washington, Global Health and Medicine, Division of Allergy & Infectious Diseases, Seattle, United States, ^2^Fred Hutchinson Cancer Center, Seattle, United States, ^3^Emory University, Atlanta, United States, ^4^Duke University, Durham, United States, ^5^National Institutes of Health, NIAID, Division of AIDS, Rockville, United States

**Background**: V3G CH848 Pr‐NP1, a ferritin nanoparticle, was designed to expand precursors to the DH270 broadly neutralizing antibody (bnAb) lineage, targeting a GDIR/K motif at the V3 loop base and N301 and N332 glycans. V3G CH848 Pr‐NP1 prime elicited potent immune responses in mice and macaques. We tested this approach in human participants without HIV, comparing two adjuvants.

**Methods**: Participants in four groups received three intramuscular (IM) vaccinations with 60 or 100 mcg of V3G CH848 Pr‐NP1 adjuvanted with 3M‐052‐AF + alum or empty LNPs on months 0, 2 and 6. Sera and PBMCs were collected to assess safety and immunogenicity.

**Results**: Thirty‐six participants ages 19−51 enrolled (16 AMAB, 20 AFAB). The frequency of local and systemic reactogenicity was similar between groups following the first three priming vaccinations, with more frequent grade 3 systemic reactogenicity and grade 1/2 fevers in participants receiving 3M‐052‐AF + alum.

After three doses of V3G CH848 Pr‐NP1, serum IgG binding to the priming protein was similar between 60 and 100 mcg doses and slightly better with 3M‐052‐AF + alum adjuvant versus LNPs. Binding to an N332A knockout protein was similar. Neutralizing antibody levels after three 100 mcg doses were similar between adjuvants (Figure 1a). Envelope base binding antibodies to gp41 were not detected after three vaccinations.

**Figure 1:**
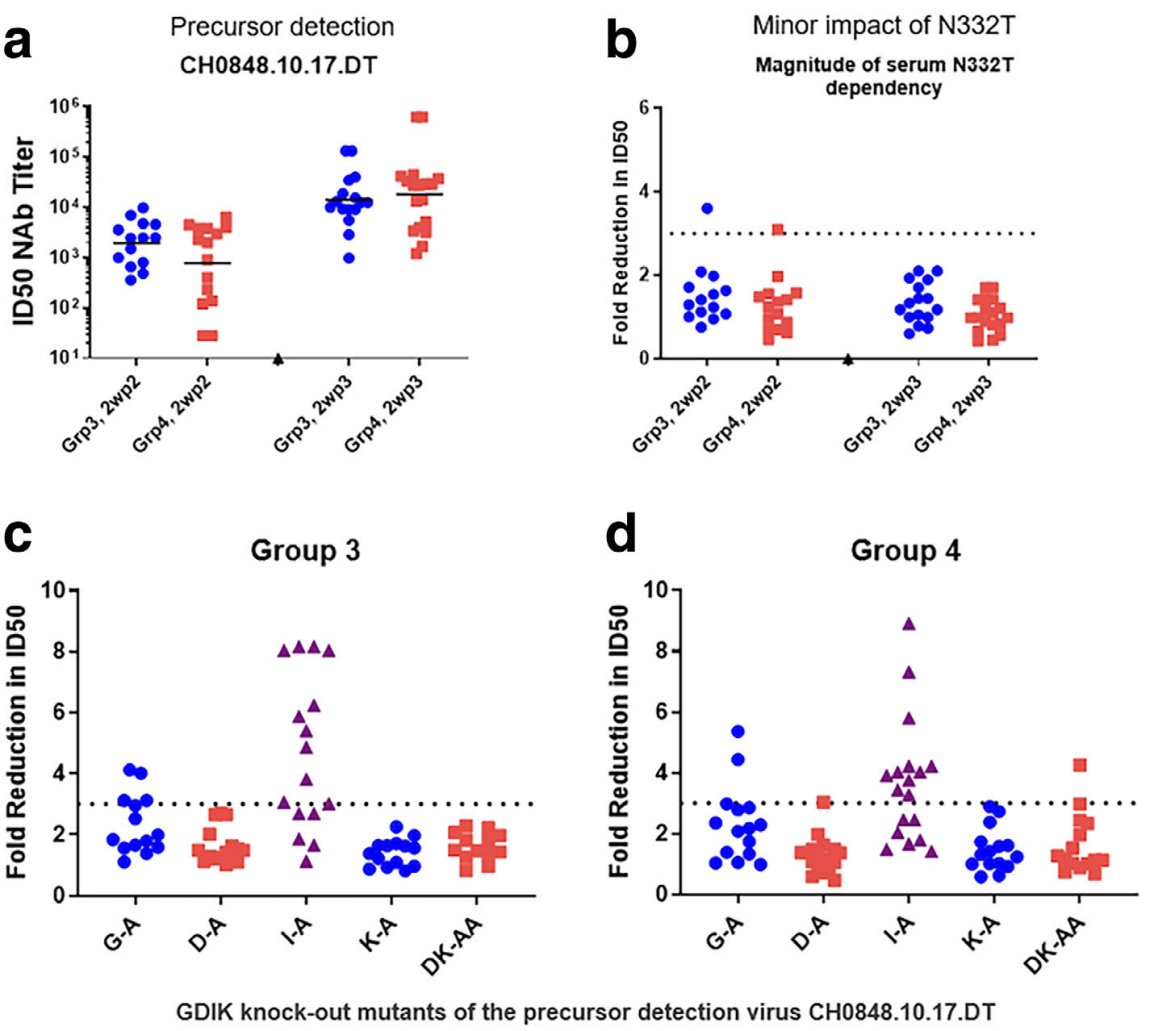
**OAA0303**: **Legend to Figure 1**. **Serum neutralization signatures of targeting at or near the V3‐glycan bnAb precursor binding site. Figure a** shows serum antibodies potently neutralize a tier 2 autologous precursor‐detection virus (CH0848.10.17.0T) 2 weeks post 2^nd^ (2wp2) vaccination and increase post 3^rd^ (2wp3) vaccination. **Panel b** shows infrequent dependency on the N332 Env glycan (as determined by the differential neutralization between CH084810.17.0T and CH0848.10.17.DT/N332A viruses). **Panel c** shows **Group 3 (100 mcg priming Env/3M052+Alum as adjuvant)** and **Panel d** shows **Group 4 (100 mcg priming Env/ empty LNP as adjuvant)** serum neutralization knock‐out by mutations in the GDIK motif in the bnAb binding site at the base of the V3 loop in both groups. Targeting the GDIK epitope was demonstrated by showing > 3‐fold reduction in neutralization ID50 by serum on viruses with the indicated GDIK mutations.

Differential serum autologous virus neutralization between the precursor detection virus CH0848.10.17.DT and the knockout CH0848.10.17.DT/N332T virus showed limited dependency of N332 on the magnitude of response (Figure 1b). However, after three priming immunizations, substitution of “A” for “I” in the GDIK binding motif reduced serum neutralization ID50 approximately four‐fold in both adjuvant groups (Figure 1c,d).

**Conclusions**: In healthy adults, V3G CH848 Pr‐NP1 priming X3 induced high‐titred serum neutralizing antibody responses to the V3‐glycan precursor detection virus that depended on the bnAb epitope GDIR/K motif but less so on the N332 glycan.

### OAA0304 Enhancement of neutralization potency and breadth by a nanoparticle vaccine containing TLR7/8 agonists

X. Xin ^1,2^, Y. Chen^3^, H. Li^1,2^, Y. Zhang^3^, M. Li^2^, W. Zhou^2^, Y. Yang^3^, W. Cao^2^, J. Yu^4^, W. Yao^4^, Y. Liu^4^, J. Qiu^1^, Q. Zhang^2^, Z. Fang^1^, X. Jiang^3^, F. Gao^1,2^

^1^Jinan University, Guangdong Second Provincial General Hospital, Integrated Chinese and Western Medicine Postdoctoral Research Station, School of Medicine, Guangzhou, China, ^2^Jinan University, Institute of Molecular and Medical Virology, Key Laboratory of Ministry of Education for Viral Pathogenesis & Infection Prevention and Control, School of Medicine, Guangzhou, China, ^3^Southern University of Science and Technology, Shenzhen Key Laboratory of Smart Healthcare Engineering, Guangdong Provincial Key Laboratory of Advanced Biomaterials, Department of Biomedical Engineering, Shenzhen, China, ^4^Jiangsu Rec‐biotechnology Co. Ltd, Taizhou, China

**Background**: Despite over 40 years of intensive research, effective HIV‐1 vaccines still remain elusive. The extensive genetic diversity of HIV‐1 underscores the need for vaccines capable of eliciting broadly neutralizing antibodies (bnAbs). However, this goal remains to be highly challenging. Therefore, innovative strategies to improve the neutralization breadth and potency of HIV‐1 vaccines are pivotal for advancing the vaccine design.

**Methods**: A novel TLR7/8‐activating nanoparticle (TNP) was developed using the microfluidic technology. TNP consists of poly(lactic‐co‐glycolic acid) (PLGA), poly(lactide‐co‐glycolide)‐b‐poly(ethylene glycol)‐N‐hydroxysuccinimide (PLGA‐PEG‐NHS), TLR7/8 agonist 1 and lipid (DLin‐MC3‐DMA). We then conjugated native‐like HIV‐1 Env trimers on the surface of TNP to generate an Env nanovaccine (Env_TNP). Lymphocytes obtained from lymph nodes and spleens of immunized BALB/c mice were analysed for antigen presentation and B cell proliferation by flow cytometry. Sera from three groups of guinea pigs immunized with Env_TNP, Env with BFA03 or Env alone were assessed for the HIV‐1‐specific binding antibodies and neutralizing antibodies (nAbs) against a panel of 20 viruses (3 tier 1s and 17 tier 2s).

**Results**: TNP containing lipid (DLin‐MC3‐DMA) formed uniform nanoparticles with better monodispersity and were more enriched in lymph nodes than TNPs containing other lipids. The final Env_TNP formed spheres with a diameter at ∼150 nm. After immunization in mice, Env_TNP enriched more Env in the lymph nodes, enhanced dendritic cell (DC) activation, increased expression of MHC‐II on DC surface and elicited strong B cell proliferation compared to Env alone or Env with BFA03. In immunized guinea pigs, Env_TNP induced significantly higher nAb titres against three tier 1 viruses compared to Env alone (*p* < 0.04). Importantly, sera from guinea pigs immunized with Env_TNP neutralized an average of 5.5 tier two viruses, significantly more than Env with BFA03 (2 viruses, *p* = 0.0138) and Env alone (2.3 viruses, *p* = 0.0140).

**Conclusions**: The new Env_TNP nanoparticles enhance the potency and breadth of nAb responses by presenting the Env trimers as large particles and through the TLR7/8 agonist's ability in promoting antigen presentation and B cell activation. Our study demonstrates that this new strategy has strong potentials to improve the immunogenicity of HIV vaccines and can be applied to other protein‐based vaccines.

### OAA0305 Luciferase‐based viral inhibition assay demonstrates ChAdOx1–MVA HIVconsvX vaccine elicits functional HIV‐1‐specific CD8^+^ T cells capable of inhibiting replication of a diverse panel of HIV‐1 isolates

N. Fernandez ^1,2^, P.J. Hayes^1,2^, N. Borthwick^3^, E.G.‐T. Wee^3^, P. Cicconi^3^, T. Hanke^3,4^

^1^IAVI Human Immunology Laboratory, Imperial College, London, United Kingdom, ^2^IAVI, New York, NY, United States, ^3^The Jenner Institute, Nuffield Department of Medicine, Oxford University, Oxford, United Kingdom, ^4^Joint Research Center for Human Retrovirus Infection, Kumamoto University, Kumamoto, Japan

**Background**: The ChAdOx1–MVA‐vectored HIVconsvX vaccine candidate targets conserved regions of the HIV‐1 proteome, thereby aiming to elicit robust, protective CD8^+^ T‐cell responses. In this first‐in‐human phase 1 trial (HIV‐CORE 0052, NCT04586673), we evaluate the vaccine's potential efficacy by assessing the functional capacity of induced CD8^+^ T cells to inhibit HIV‐1 replication in autologous CD4 T cells, using a luciferase‐based viral inhibition assay (VIA).

**Methods**: CD4^+^ and CD8^+^ T cells were polyclonally expanded from peripheral blood mononuclear cells (PBMCs) isolated from trial participants. CD8^+^ T cells from pre‐vaccination day 0 (D0), day 35 (D35) and day 140 (D140) time points were co‐cultured with pre‐vaccination autologous CD4^+^ T cells infected with a panel of eight replication‐competent HIV‐1 infectious molecular clones (IMCs) expressing the Renilla reniformis luciferase (LucR) reporter gene. CD8^+^ T‐cell‐mediated inhibition was calculated as log_10_ relative light units (RLU) reduction in CD4^+^/CD8^+^ T‐cell co‐cultures, compared with cultures of infected CD4^+^ T cells alone.

**Results**: HIVconsvX vaccination induced CD8^+^ T cells with broad, cross‐clade HIV‐1 inhibition against a panel of viruses derived mostly from transmitted/founder HIV‐1s isolates from different countries representing the major global clades A, B, C and D. Peak post‐vaccination CD8^+^ T‐cell responses at day 35 inhibited a mean of 7.8 (SD 0.4) IMCs, decreasing to a mean of 6.4 (2.7) at day 140.

**Conclusions**: The ChAdOx1–MVA HIVconsvX vaccine candidate induces functional HIV‐1‐specific CD8^+^ T‐cell responses capable of inhibiting HIV‐1 replication in vitro. These CD8^+^ T‐cell effectors inhibited HIV‐1 IMCs originating from diverse geographical regions, types (founder/transmitter vs. lab‐adapted) and tropisms (CCR5 vs. CXCR4). The luciferase‐based VIA provides a valuable tool for evaluating the potential efficacy of HIV‐1 vaccines in inducing protective CD8^+^ T cells, thereby informing on further vaccine development and testing.

### OAA0402 Spatial transcriptomics reveals persistent reservoirs in the rhesus macaque gut are associated with tertiary lymphoid organs and stress response activation

T. Hope ^1^, Y. Thomas^1^, S.P. Pascoe^1^, M.S. Arif^1^, C. Thuruthiyil^1^, M.A. Shaaban^2^, F.A. Engelmann^1^, J.M. Hasson^2^, I. Clerc^2^, M.D. McRaven^1^, F. Villinger^3^, R. Lorenzo‐Redondo^2^

^1^Northwestern University, Cell and Developmental Biology, Chicago, United States, ^2^Northwestern University, Department of Medicine, Chicago, United States, ^3^New Iberia Research Center, New Iberia, United States

**Background**: Despite effective antiretroviral therapy (ART), HIV‐1 persistence is the major obstacle to a functional cure. Thus, understanding the tissue microenvironment of the reservoirs of persistence during ART is key.

**Methods**: Utilizing immunoPET/CT‐identified foci of SIV gene expression utilizing the rhesus macaque model, we compared the spatial transcriptomics (10x Visium) of the local gut tissue neighbourhood of the rebound eclipse‐phase foci (4−6 days post‐ATI) from animals initiating ART 4 days (early‐seeded reservoir with short lifespan) or 10 weeks (persistent reservoir) after high‐dose challenge. Adjacent sections from tissues containing foci of rebounding virus were evaluated with SIV proviral PCR, immunofluorescence and spatial transcriptomics.

**Results**: SIV presence was always associated with higher transcriptional levels and up‐regulation of genes related to SIV infection and innate immune responses was observed for both types of reservoirs. Notably, we also detected significant differences between the short lifespan and persistent reservoirs. The biomarkers and pathways for the persistent reservoir revealed the activation of the integrated stress response with its characteristic translational reprograming through the disruption of cap‐dependent translation and cellular adaptation. Collectively, the pathway analyses show many parallels between the foci of the rebound of the persistent reservoir 5 or 6 days after ATI and the tumour microenvironment (TME) which is the contextual basis of modern cancer research. SIV in persistent reservoirs was distinctly associated with IgA plasma cells, monocytes and cycling gamma‐delta T cells, while in short lifespan, reservoir was associated with IgG plasma cells, Th17 cells, DCs and epithelial cells suggesting that persistent reservoirs are associated with gut tertiary lymphoid organs and characterized by a status of reprogramed translation and transcriptional activation of genes associated with the integrated stress responses function to restore cellular homeostasis.

**Conclusions**: We propose that the integrated stress response facilitates the development of a viral microenvironment characterized by altered cell metabolism, reduced viral translation (IRES‐dependent), altered extracellular matrix and compromised immune cell function very analogous to the well‐defined TME. The immune checkpoint markers that are remarkably identical between cancer and HIV infection (PD‐1, PDL‐1, CTLA‐4, etc.) are consistent with both utilizing the altered microenvironment as a physiological sanctuary where they can persist while evading immune responses.

### OAA0403 Alterations in plasma metabolomic biomarkers following HIV acquisition and association with reservoir size in people on antiretroviral therapy

O.O. Baiyegunhi ^1^, K. Naidoo^2^, M. Mohamed^3^, M. Maimela^1^, K. Reddy^1^, Z. Zhang^4^, L. Mazibuko^1^, A. Harms^4^, T. Hankemeier^4^, H. Mwambi^5^, M.J. Bunders^6,7^, M. Alfeld^2,8^, J. Mann^9^, T. Ndung'u^1,9,10^

^1^Africa Health Research Institute, Basic and Translational Science, Durban, South Africa, ^2^Leibniz Institute of Virology, Department of Virus Immunology, Hamburg, Germany, ^3^University of KwaZulu‐Natal, Statistics, Durban, South Africa, ^4^Leiden University, Metabolomics and Analytics Center, Leiden, Netherlands, the, ^5^University of KwaZulu‐Natal, School of Mathematics, Statistics and Computer Science, Pietermaritzburg, South Africa, ^6^University Medical Center Hamburg‐Eppendorf, III. Department of Medicine, Hamburg, Germany, ^7^Ragon Institute of MGH, MIT, and Harvard University, Boston Massachusetts, United States, ^8^German Center for Infection Research (DZIF), Partner Site Hamburg‐Lübeck‐Borstel‐Riems, Hamburg, Germany, ^9^University of KwaZulu‐Natal, HIV Pathogenesis Programme, Durban, South Africa, ^10^University College London, Division of Infection and Immunity, London, United Kingdom

**Background**: Metabolic disorders are common in people living with HIV with or without antiretroviral therapy (ART) compared to population controls. Metabolic biomarkers have also been associated with immune dysfunction and HIV post‐treatment control. The metabolic state of immune cells determines their susceptibility to the virus and the establishment of latency, but the mechanisms have not been fully explored in African populations with a high burden of HIV. We performed an unbiased investigation of alterations in plasma metabolic biomarkers in a longitudinal cohort of individuals who were followed before and after HIV acquisition and explored association with clinical outcomes and HIV reservoir size.

**Methods**: We studied 47 participants living with HIV, of whom 18 were diagnosed in hyperacute phase (before peak viraemia). Samples from before HIV acquisition, during untreated acute disease and after 1 year on ART were analysed. The plasma metabolome was measured using liquid chromatography‐mass spectrometry, and HIV reservoir size was measured using droplet digital PCR.

**Results**: We measured 209 metabolites, comprising amines and signalling lipids, with 46 metabolites (26 were higher [30% amines] and 20 [25% amines] lower) differing between HIV‐negative and acute HIV groups, confirmed by Random Forest analysis with 87.5% accuracy. These were linked to various metabolic pathways, including alanine, aspartate, glutamine metabolism, sphingolipid and butanoate metabolism, and the TCA cycle. One‐year post‐ART initiation, reservoirs were undetectable in 4 of 18 participants. Twelve metabolites previously linked to rebound were quantified, with glutamic acid and glycoursodeoxycholic acid correlating negatively and positively with reservoir size, respectively. Factors associated with reservoir size also included CD4/CD8 counts and CD4:CD8 ratio.

**Conclusions**: These findings highlight metabolome alterations during HIV, and potential metabolomic associations with reservoir size, guiding future studies. Metabolic biomarkers of HIV reservoir size will enhance the understanding of HIV pathogenesis and inform safer analytical treatment interruption strategies.

### OAA0404 Elevated CCR7 ligands CCL19 and CCL21 in aorta of PLWH promote immune cell recruitment

L.M. Obare ^1^, X. Zhang^1^, K. Nthenge^1^, V.R. Stephens^1^, Y.R. Su^1^, T. Absi^1^, C. Gabriel^1^, M. Mashayekhi^1^, S. Bailin^1^, J.R. Koethe^1^, C.N. Wanjalla^1^

^1^Vanderbilt University Medical Center, Medicine, Nashville, United States

**Background**: People living with HIV (PLWH) face a 1.5‐ to 2‐fold higher risk of cardiovascular disease (CVD), partly driven by chronic immune activation despite viral suppression. CCL19 and CCL21, ligands for C‐C chemokine receptor 7 (CCR7), play critical roles in immune cell migration and inflammation. We hypothesize that increased CCL19/CCL21 expression in the aorta contributes to immune cell recruitment, vascular inflammation and atherosclerosis in PLWH.

**Methods**: We used matched Formalin‐fixed paraffin‐embedded thoracic aorta from the National Disease Research Interchange (NDRI; PLWH, *n* = 4) and people without HIV (PWoH, *n* = 4). The whole transcriptional profile of the aorta with spatial resolution was performed using Visium v1. Differential expression of CCL19/CCL21 was analysed using a negative binomial model. For single‐cell resolution, spatial transcriptomics of coronary arteries from PLWH (*n* = 2) and PWoH (*n* = 2) were profiled using CosMX with the 1000‐plex RNA panel.

**Results**: Aortas of PLWH had significantly upregulated CCL19 (*p* = 1.25e‐9) and CCL21 (*p* = 5.20e‐63) compared to PWoH (Figure 1a). Spatial resolution shows that CCL19 and CCL21 transcripts are expressed by cells in the media and the adventitia (Figure 1b). In a separate analysis of coronary arteries from both PLWH and PWoH, CCL19 transcripts were expressed by vascular smooth muscle cells and CCL21 by endothelial cells.

**Figure 1:**
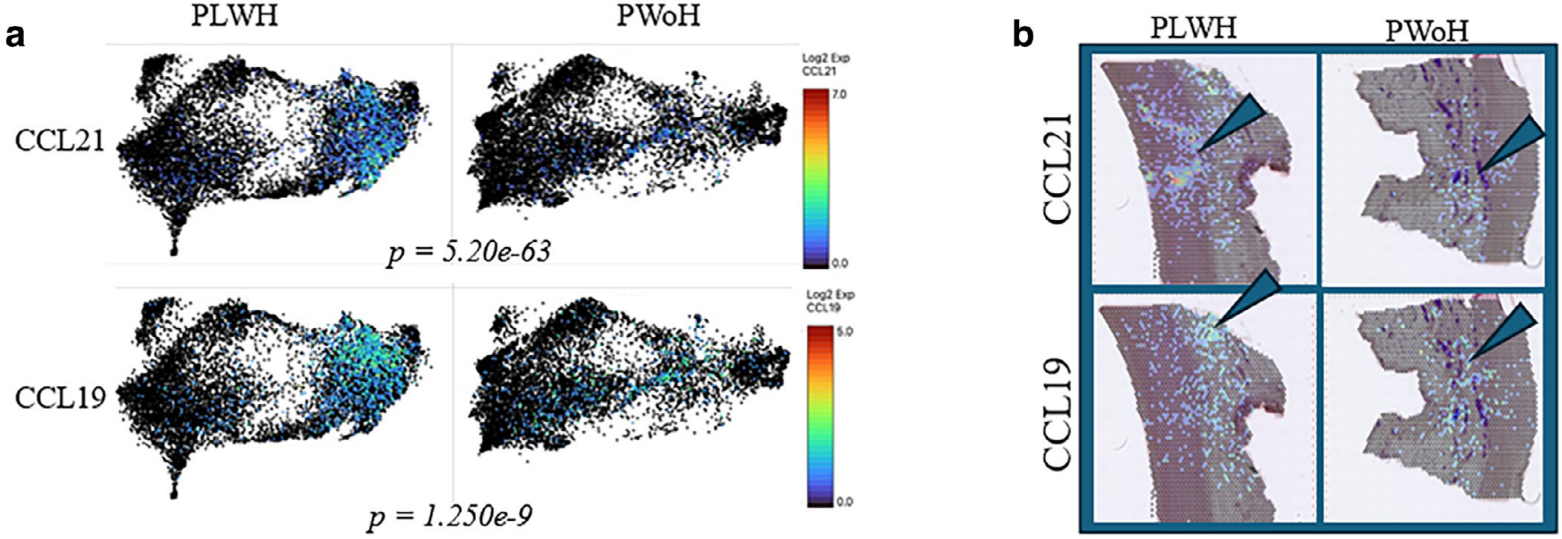
**OAA0404: Spatial gene expression of CCL19/CCL21 using the Visium platform**. (a) Uniform Manifold Approximation and Projection (UMAP) was used to integrate sections from PWoH (*n* = 4) and PLWH (*n* = 4), showing CCL19/CCL21 gene expression. (b) CCL19/CCL21 gene transcripts from Visium are shown on representative 54m sections from PLWH and PWoH. Blue arrows highlight areas of gene expression.

**Conclusions**: CCL19 and CCL21 have previously been implicated in atherosclerotic cardiovascular disease. Upregulated expression of CCL19/CCL21 in the aorta of PLWH may increase naïve CD4 and CD8 T‐cell recruitment and promote arterial inflammation. Additional studies are needed to investigate this potential mechanism contributing to heightened CVD risk in PLWH and the potential therapeutic value of targeting CCR7‐mediated pathways to mitigate vascular inflammation.

### OAA0405 The predominant effects of ART and sex on the plasma metabolomes of virally suppressed children living with perinatally acquired HIV: implications for cure studies

C. Herbert ^1^, L. Kuhn^2^, N. Tobin^3^, F. Li^3^, R. Strehlau^4^, F. Patel^5^, T. Wang^2^, S. Wang^2^, G. Aldrovandi^3^, C. Tiemessen^1^

^1^National Institutes for Communicable Diseases, and Faculty of Health Sciences, University of the Witwatersrand, Johannesburg, South Africa, Johannesburg, South Africa, ^2^Columbia University, Irving Medical Center, New York, United States, ^3^University of California Los Angeles, Department of Pediatrics, Los Angeles, United States, ^4^University of the Witwatersrand, VIDA Nkanyezi Research Unit, Rahima Moosa Mother and Child Hospital, Department of Paediatrics and Child Health, Faculty of Health Sciences, Johannesburg, South Africa, ^5^University of the Witwatersrand, Wits RHI, Faculty of Health Sciences, Johannesburg, South Africa

**Background**: The influence of host factors, such as the metabolome, on HIV remission and cure interventions has garnered increasing interest. Metabolism in children living with perinatally acquired HIV (CLWH) is less well understood than in adults.

**Methods**: To characterize the metabolic profiles of CLWH, plasma from pre‐pubescent, virally suppressed CLWH (*n* = 155) and HIV‐negative controls (HNCs, *n* = 155) were analysed by Metabolon using untargeted ultra high‐performance liquid chromatography/tandem accurate mass spectrometry. CLWH were on efavirenz (EFV, *n* = 101) or lopinavir/ritonavir (LPV, *n* = 54)‐based antiretroviral therapy (ART) regimens. Three main comparisons were used to explore the major metabolic patterns in CLWH and their relation to clinical parameters.

**Results**: The specific ART regimen produced the most pronounced patterns in the metabolic profiles of CLWH. The two regimens (comparison 1) were distinguished predominantly by altered bile acids, bilirubin, bilirubin catabolites and androgenic steroids. These changes likely originated from divergent antiretroviral interactions with human enzymes, transporters and the gut microbiome. The sexes (comparison 2) in all groups were consistently distinguished by cholesterol sulphate levels, but most of the sex differences in the CLWH were distinct from those in HNCs. Within a subset of EFV‐CLWH with long‐term viral suppression (comparison 3, undetectable viral load with occasional blipping, for > 6 years, with no blips > 1000 HIV RNA copies/ml, *n* = 49), two metabotypes were identified and compared. Metabotype A showed significantly more impaired mitochondrial oxidative metabolism relative to Metabotype B and had significantly lower contemporaneous (*p* < 0.001) and longitudinal (*p* = 0.008) CD4 T‐cell percentages (CD4%). Metabotypes were not associated with age at ART initiation, pre‐ART CD4% and viral load, time to suppression, longitudinal viral load or HIV DNA Ct value (reservoir size proxy).

**Conclusions**: The specific ART regimen dominated the plasma metabolic profile and significantly influenced the presentation of metabolic sexual dimorphism in CLWH. Two metabotypes among CLWH on the same regimen suggested divergent, sub‐clinical responses to the same treatment even among those with long‐term viral control and CD4% approaching healthy levels. It is unlikely that metabolic biomarkers will be reliable in predicting or monitoring intervention outcomes across regimens. Grouping of treated individuals in metabolic studies should be done with careful consideration for the ART regimen used.

### OAA0502 Early treated children with perinatal HIV show elevated monocyte activation into adolescence

C. Davies ^1^, F. Vaida^2^, T. Webber^3^, P. Bouic^4^, B. Tang^5^, K. Otwombe^6^, M.F. Cotton^7^, S. Browne^8^, S. Innes^7^

^1^Stellenbosch University, Division of Epidemiology and Biostatistics, Cape Town, South Africa, ^2^University of California San Diego, Division of Biostatistics and Bioinformatics, San Diego, United States, ^3^Stellenbosch University, Division of Immunology, Faculty of Medicine and Health Sciences, Cape Town, South Africa, ^4^Stellenbosch University, Division of Medical Microbiology, Cape Town, South Africa, ^5^University of California San Diego, Department of Psychiatry, San Diego, United States, ^6^University of the Witwatersrand, Perinatal HIV Research Unit, Johannesburg, South Africa, ^7^Stellenbosch University, Family Center for Research with Ubuntu, Department of Paediatrics & Child Health, Cape Town, South Africa, ^8^University of California San Diego, School of Public Health, San Diego, United States

**Background**: Universal early ART for infants born with HIV has dramatically improved long‐term outcomes in children living with perinatal HIV (CHIV). However, these children face decades of ART exposure and immune activation. Neither the long‐term inflammatory profile nor risk of non‐communicable diseases are yet known. We compared longitudinal biomarkers associated with inflammation, metabolic and cardiovascular damage and response markers in early ART‐treated children and controls unexposed to HIV.

**Methods**: One hundred and eighty‐five children (67 CHIV, 118 HIV‐unexposed) aged 7−16 years (87 female, 98 male), previously part of CHER early ART trial, were followed at Tygerberg Hospital, South Africa. CHIV initiated ART within 3 months of birth, and were virologically suppressed (> 90%), asymptomatic, with preserved CD4^+^ cell count and clinically well at the time of blood tests. Unexposed children arose from the same communities and socio‐economic background. Children had multiple measurements (median 2, 4 years apart), yielding 321 total observations. We measured 26 biomarkers in blood serum using ELISA and Luminex™ multiplex immunoassays, comprising monocyte and lymphocyte‐derived biomarkers, response, metabolic and cardiovascular markers. We used mixed effects models with child‐specific random effects to investigate the association between HIV group and each biomarker, adjusting for age, sex, ethnicity, smoke exposure and batch, and considered an interaction between age and HIV group. Results are adjusted for multiple comparisons.

**Results**: Early treated children had significantly higher CRP, sCD14 and VEGF than unexposed. Age‐specific effects were observed with significantly higher MCP‐1, IL‐6 and P‐selectin in CHIV aged 6−8 years, significantly higher MCP‐1, Adiponectin and IL1a in ages 8−10 years, and significantly higher MCP‐1 in ages 13−16 years. All other biomarkers showed no significant difference.

**Conclusions**: Despite early ART and sustained viral suppression, early treated children displayed extensive immune marker elevations, clustered around monocyte activation and markers associated with cardio‐metabolic disease. These children remain at high risk of premature vascular disease in adulthood.[Fig jia226518-fig-0004]

**Figure 1 jia226518-fig-0004:**
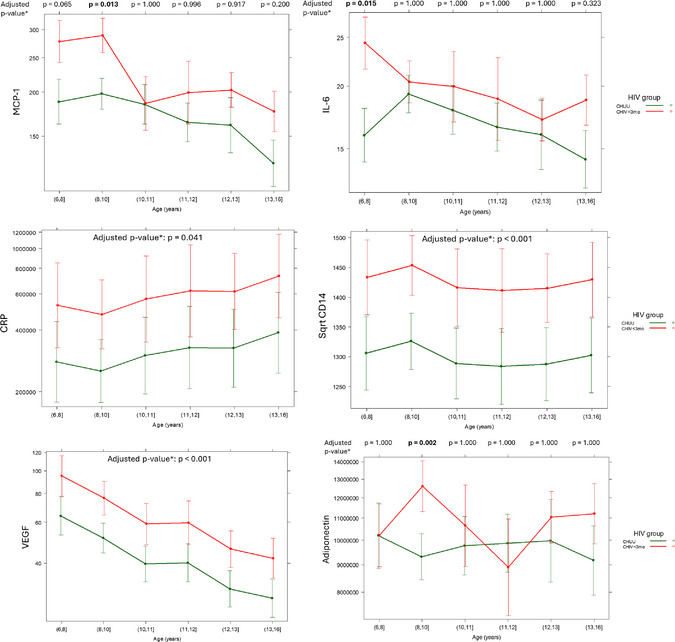
**OAA0502: Graph of selected predicted biomarkers by age for unexposed (CHUU) and early‐treated children living with HIV (CHIV). P‐values are adjusted for age, sex, smoke exposure, batch, ethnicity and multiple comparisons. Error bars represent 95% confidence intervals**.

### OAA0503 Impact of BCG vaccination on innate immune responses in HIV‐exposed infants who have not acquired HIV: a randomized controlled trial in Uganda

R. Namakula ^1^, S. Cose^2,3^, V. Nankabirwa^4^, O. Namugga^1^, M.G. Netea^5^, H. Steinsland^1^, H. Sommerfelt^1^, K. Hanevik^1,6^

^1^University of Bergen, Centre for International Health, Bergen, Norway, ^2^MRC/UVRI and LSHTM Uganda Research Unit, Department of Immunology, Kampala, Uganda, ^3^London School of Hygiene and Tropical Medicine, Department of Clinical Research, London, United Kingdom, ^4^Makerere University School of Public Health, Department of Epidemiology and Biostatics, Kampala, Uganda, ^5^Radboud University Medical Centre, Department of Internal Medicine, Nijmegen, Netherlands, the, ^6^Haukeland University Hospital, Department of Medicine, Bergen, Norway

**Background**: Bacillus Calmette‐Guérin (BCG) vaccination has been shown to train the innate immune system, leading to enhanced responsiveness to subsequent triggers. However, induction of training is not well understood in the growing population of HIV‐1‐exposed infants who have not acquired HIV (HEU) in sub‐Saharan Africa. We aimed to compare the trained immune response in HEU infants who received BCG at birth with the response in HEU infants who received BCG 14 weeks of age.

**Methods**: A randomized controlled trial was performed among 182 HEU infants born at a health facility in Kampala, Uganda, where 91 received BCG at birth (early arm) and 91 received BCG at age 14 weeks (late arm). Whole‐blood cultures stimulated with Toll‐like receptor (TLR) agonists (TLR2/1, TLR4, TLR7/8) and MTB lysate antigens for 18 hours were done using venous blood drawn at birth, and at weeks 6, 14 and 52. Cytokine concentrations (TNF, IL‐1β, IL‐6 and IL‐10) were measured using Luminex immunoassay of culture supernatants. Statistical analyses were done with Student's *t*‐test on log‐transformed cytokine concentration responses.

**Results**: Among 182 randomized infants, four died and one was lost to follow up before 52 weeks. Randomization successfully balanced relevant baseline characteristics. All TLR agonists generally elicited stronger responses in the early BCG arm than in the late arm, at both week 6 and week 14.The IL‐1β and IL‐10 responses increased significantly following TLR2/1 stimulation in BCG vaccinated infants compared to infants who had not yet been vaccinated, with 1.41 (95% CI 1.00−1.98; *p* = 0.048) and 1.33 (95% CI 1.02−1.73; *p* = 0.031) fold increases at week 14, respectively. Significantly higher IL‐6 response at week 6 and IL‐10 response at week 14 after TLR7/8 stimulation in the group receiving BCG at birth compared to the group yet to receive BCG was observed. At week 52, there were no significant differences in cytokine production between the two groups.

**Conclusions**: This study with low attrition rate, TLR agonists induced stronger innate cytokine responses in BCG vaccinated HEU infants. Our data indicates that BCG vaccination at birth induces robust trained immunity in HEU infants until 14 weeks of age and recommended in this vulnerable population.

### OAA0504 An immunoinformatics analysis pipeline (IMAP) identifies novel genetically conserved and immunogenic peptides found in rebound HIV‐1 during analytical treatment interruption

J. Marin‐Rojas ^1,2^, E. Lee^1^, S. Cronin^1,2^, K. Fisher^1,2^, J. Simpson^1,2^, S. Deeks^3^, A. Kelleher^4^, G. Duette^1,2^, S. Palmer^1,2^

^1^The Westmead Institute for Medical Research, Center for Virus Research, Sydney, Australia, ^2^University of Sydney, Faculty of Medicine and Health, Sydney, Australia, ^3^University of California, Department of Medicine, San Francisco, United States, ^4^University of New South Wales, Kirby Institute, Sydney, Australia

**Background**: HIV‐specific CD8 T‐cell responses directed against genetically conserved HIV‐1 protein regions are associated with viral control. Therefore, we applied a novel **IM**munoinformatics **A**nalysis **P**ipeline (IMAP) to identify 182 peptides (IMAP‐peptides) from five HIV‐1 protein regions (Gag, Pol, Vif, Vpr and Env), which were genetically conserved across global HIV‐1 variants while avoiding known immune escape mutations. We assessed whether the selected IMAP‐peptides were found within rebound virus derived from participants who experienced analytic treatment interruption (ATI). Furthermore, we characterized the corresponding CD8 T‐cell response.

**Methods**: During the PULSE clinical trial, 68 men who have sex with men living with HIV‐1 in Australia underwent three consecutive ATIs. Remarkably, seven participants transiently controlled HIV rebound during the third ATI. We obtained plasma and/or blood‐derived mononuclear cells from four non‐controllers (NCs) who experienced rapid viral rebound during all ATIs, and five transient‐controllers (TCs) who exhibited control during the third ATI. We assessed whether the IMAP‐peptides were present within HIV‐1 RNA sequences from rebound virus across the ATIs in NCs and TCs. In addition, the effector response to these IMAP‐peptides and NIH‐provided control peptide pool (without IMAP‐peptides) were determined by IFN‐γ/TNF‐α production and degranulation (CD107a/b).

**Results**: Near full‐length HIV‐1 RNA sequencing of rebound virus from three NCs and two TCs revealed the Gag, Pol, Vif, Vpr and Env IMAP‐peptides were found in 52−100% of the viral sequences obtained from these five participants across three ATI time points. Moreover, the CD8 T cells from three TCs had a 15‐ to 53‐fold higher effector response to the IMAP‐peptides compared to the CD8 T cells from two NCs. Notably, in NCs, the relative response to the IMAP‐peptides was 20‐fold lower compared to the control peptides, whereas in TCs, the IMAP‐peptide response was similar to the control response (1‐ to 1.58‐fold change).

**Conclusions**: The novel IMAP‐peptides were found within the HIV‐1 RNA sequences during viral rebound in transient‐controllers and non‐controllers, indicating these peptides are expressed during HIV‐1 infection. The higher potential of CD8 T cells from transient‐controllers to recognize and respond to mutational‐constrained HIV epitopes, may contribute to their virological control. These results highlight the potential of IMAP for identifying novel immunogens for therapeutic strategies to enhance CD8 T‐cell response.

### OAA0505 Plasma extracellular vesicles and their association with hepatocellular carcinoma in individuals living with HIV and hepatitis C virus: potential biomarkers

A.A. Osegueda Peña ^1^, L. Leicaj^1^, A. Adamczyk^1^, N. Cruz Molina^2^, L. Cruces^1^, L. Baquero^1^, T. Langer^1^, V. Gonzalez Polo^1^, M. Ostrowski^1^, N. Laufer^1,2^

^1^CONICET – Universidad de Buenos Aires. Instituto de Investigaciones Biomédicas en Retrovirus y SIDA (INBIRS), Buenos Aires, Argentina, ^2^División Infectología, Hospital Fernández, Buenos Aires, Argentina

**Background**: Plasma extracellular vesicles (pEV) play a crucial role in immune modulation. Natural killer (NK) cells are essential for immune surveillance, particularly in targeting tumour cells. Advanced liver fibrosis (LF) increases hepatocellular carcinoma (HCC) risk, even after successful HCV clearance with direct‐acting antivirals (DAA). We previously showed that NK cells exhibit an exhausted phenotype and reduced function in individuals with advanced LF post‐DAA. This study aimed to assess the impact of pEV from individuals with HIV/HCV: (a) with advanced LF, and (b) those with advanced LF who later developed HCC.

**Methods**: Plasma samples were collected from individuals with advanced liver disease (METAVIR F4, *n* = 11) achieving sustained virologic response (SVR) post‐DAA. Samples were taken at baseline (pre‐DAA) and post‐SVR intervals; five developed HCC with a median of 5.75 years (IQR 4−8) post‐clearance. Plasma from six healthy controls was also included.

pEV were isolated via size exclusion chromatography from plasma (2 ml), resuspended in PBS (60 µl) and incubated with healthy PBMC for 1 hour. NK cell degranulation was assessed by FACS analysis of CD107a, CD3, CD56 and a viability marker after coculture with K562 cells (10:1 ratio) for 2 hours.

**Results**: pEV from all participants, including controls, reduced NK CD107a expression compared to NK cells without pEV. pEV from individuals with advanced LF at baseline were more suppressive than controls (0.71 [0.60−0.85] vs. 0.88 [0.78−0.97]; *p* = 0.023). After ≥1‐year post‐DAA, pEV from those who developed HCC remained more suppressive than those who did not (0.75 [0.71−0.78] vs. 0.86 [0.84−0.90]; *p* = 0.008). In non‐HCC individuals, NK suppression decreased over time (*p* = 0.052), unlike in those who developed HCC. At ≥1 year, suppression by pEV in non‐HCC individuals was similar to controls (*p* = 0.79), whereas pEV from future HCC cases remained more suppressive (*p* = 0.03).

**Conclusions**: pEV from individuals with advanced LF contribute to the exhausted NK cell phenotype, and could be associated with the increased risk of HCC among those individuals. Notably, pEV from those who developed HCC sustained long‐term suppression of NK functionality, unlike pEV from non‐HCC individuals, which resembled healthy controls ≥1‐year post‐treatment. These findings suggest that persistent NK suppression by pEV after HCV clearance could serve as a biomarker for HCC risk.

### OAB0102 A double‐blind, active‐controlled, phase 2b study to evaluate the efficacy and safety of ulonivirine in combination with islatravir in virologically suppressed adults living with HIV‐1

J.‐M. Molina ^1^, M.N. Ramgopal^2^, C. Katlama^3^, D.L. Cunningham^4^, D.P. Hagins^5^, D.L. Braun^6^, O.O. Osiyemi^7^, E. Hofmann^8^, C.A. Dietz^9^, G. Lin^10^, K. Beckey^10^, S. Rawlins^10^, R.P. Matthews^10^, W. Greaves^10^

^1^St‐Louis and Lariboisière Hospitals, APHP, University of Paris Cité, Paris, France, ^2^Midway Immunology and Research Center, Fort Pierce, United States, ^3^Institut Pierre Louis d'Epidémiologie et de Santé Publique, APHP, Sorbonne Université, Paris, France, ^4^Pueblo Family Physicians Ltd, Phoenix, United States, ^5^Chatham County Health Department, Savannah, United States, ^6^University of Zurich, Zurich, Switzerland, ^7^Triple O Research Institute, West Palm Beach, United States, ^8^Department of Infectious Diseases, Inselspital, Bern University Hospital, University of Bern, Bern, Switzerland, ^9^KC Care Health Center, Kansas City, United States, ^10^Merck & Co., Inc., Rahway, United States

**Background**: Ulonivirine (ULO, MK‐8507) is a non‐nucleoside reverse transcriptase inhibitor (NNRTI) suitable for once‐weekly (QW) oral dosing, being developed with islatravir (ISL) for the treatment of HIV‐1. This double‐blind, phase 2b trial evaluated the efficacy and safety of ISL+ULO QW in virologically suppressed adults living with HIV‐1 (MK‐8591‐013; NCT04564547).

**Methods**: Participants without known NNRTI resistance who were virologically suppressed for ≥6 months on bictegravir/emtricitabine/tenofovir alafenamide (BIC/FTC/TAF), were randomized (1:1:1:1) to receive ISL 20 mg plus ULO (100, 200 or 400 mg) QW, or remain on BIC/FTC/TAF once‐daily. Due to decreases in total lymphocyte counts (TLC) and CD4^+^ counts observed across ISL programmes, the trial was stopped early and participants were transitioned to non‐study antiretroviral therapy with continued monitoring until recovery. Efficacy assessments included the proportion of participants maintaining virological suppression and measurement of CD4^+^ counts; safety assessments included adverse event (AE). ISL+ULO data were pooled across the doses.

**Results**: One hundred and sixty‐one participants were randomized (121 ISL+ULO, 40 BIC/FTC/TAF); 80.7% were male and 64.6% were White, with a mean age of 45 years and baseline CD4^+^ of 723 cells/mm^3^. When dosing of ISL+ULO was discontinued, 113 participants had reached Week 24. Virologic suppression (HIV‐1 RNA < 50 copies/ml) was maintained in all participants who had available data across groups at Week 24. Participants receiving ISL+ULO reported similar rates of AEs (76.9% vs. 67.5%), drug‐related AEs (17.4% vs. 10.0%) and AEs leading to study discontinuation (2.5% vs. 0.0%), as participants receiving BIC/FTC/TAF. There was no increase in the incidence of infection among participants receiving ISL+ULO (ISL+ULO: 39.6% vs. BIC/FTC/TAF: 36.1%). Mean percentage change from baseline in TLC and CD4^+^ counts showed decreases for participants receiving ISL+ULO (−23.4% and −19.4%) versus BIC/FTC/TAF (2.0% and 2.3%) at Week 24. Recovery in TLC and CD4^+^ counts towards baseline was observed in all ISL+ULO groups at Week 24 post‐treatment.

**Conclusions**: ISL+ULO showed efficacy in maintaining viral suppression through Week 24. Consistent with previous studies of ISL, decreases in TLC and CD4^+^ counts were observed with ISL 20 mg + ULO QW followed by a trend to recovery after discontinuation. Development of ISL 2 mg + ULO QW is ongoing.

### OAB0103 First experience of dual antiretroviral maintenance regimen in West and Central Africa compared with continuing 3‐drug current antiretroviral regimens (CARs): week 96 results from the MODERATO trial (ANRS ‐MIE 12372)

F. Fadiga ^1^, D. Gabillard^2^, R. Moh^3^, S. Niangoran^4^, Z. Diallo^5^, A. Poda^6^, I. Diallo^7^, C. Kouanfack^8^, V. Petrov‐Sanchez^9^, A. Diallo^10^, A. Benalycherif^11^, S.P. Eholié^12^, R. Landman^13^

^1^Programme PAC‐CI‐PRISME, Abidjan, Côte d'Ivoire, ^2^National Institute for Health and Medical Research UMR1219, IRD EMR271, Bordeaux, France, ^3^Unité Pédagogique de Dermatologie et Infectiologie, UFR Sciences Médicales et Programme PAC‐CI‐PRISME, Abidjan, Côte d'Ivoire, ^4^Programme PAC‐CI PRISME, Abidjan, Côte d'Ivoire, ^5^Unité Pédagogique de Dermatologie et Infectiologie, UFR des sciences, Université Félix Houphouet Boigny, Centre de Recherche en Maladies Infectieuses et Pathologies Associées, Abidjan, Côte d'Ivoire, ^6^CHU Souro Sanou, Hopital de Jour, Service des Maladies Infectieuses, Bobo‐Dioulasso, Burkina Faso, ^7^CHU Yaldago Ouedraogo, Service de Médecine Interne, Ouagadougou, Burkina Faso, ^8^Hopital Central de Yaoundé, Service des Maladies Infectieuses, Yaoundé, Cameroon, ^9^ANRS Maladies Infectieuses Emergentes ‐ MIE, Paris, France, ^10^ANRS Maladies Infectieuses Emergentes ‐ MIE, Pharmacovigilance, Paris, France, ^11^Institut de Médecine et d'epidémiologie Appliquée‐ IMEA, Recherches Cliniques, Paris, France, ^12^Centre de Recherches en Maladies Infectieuses et Pathologies Associées, Ministère de la Santé CHU de Treichville, Unité Pédagogique de Dermatologie et Infectiologie, Abidjan, Côte d'Ivoire, ^13^Institut de Médecine et D'épidémiologie Appliquée, IAME‐UMR1137, Hôpital Bichat Claude Bernard, Service des Maladies Infectieuses, Paris, France

**Background**: Switching to DTG/3TC or boosted protease inhibitor/3TC had demonstrated long‐term non‐inferior efficacy versus continuing tenofovir (TDF) 3 drugs‐based regimens and are recommended in guidelines from northern countries, but not in WHO recommendations. The MODERATO trial in West and Central Africa aims to demonstrate the non‐inferiority at 96 weeks of two arms of maintenance dual therapy DTG/3TC or ATV/r/3TC compared to CARs with TDF/3TC/efavirenz (EFV) (TLE) or TDF/3TC/DTG (TLD) in person living with HIV with virological success.

**Methods: ​**From November 2020 to February 2023, in three countries, we randomized HIV‐1‐infected adults on CARs with TLE or TLD for at least 2 years, without a history of virological failure, AgHbs‐ and CD4 cell count > 200/m. The primary endpoint was the proportion of randomized participants demonstrating non‐inferiority of a switching to dual‐drug maintenance therapy versus continuation of the 3 drugs CARs in terms of virological success at week 96, with no change in strategy. Virological success was estimated using the FDA Snapshot approach, with a 10% non‐inferiority margin.

**Results: ​**Overall, 480 adults (79% women; 52% aged ≥50 years, median duration on ARV regimen 9 years, mean CD4 count 768/mm^3^) were randomized. The ITT population included 480 participants (160 on DTG/3TC, 160 on ATV/r/3TC and CARs) (TLE *n* = 149 and TLD *n* = 11). At week 96, in the ITT population, the treatment success rate was 95.0% (304/320) in the dual‐drug group and 96.9% (155/160) in the CARs group (adjusted difference −1.9%, 95% CI −5.5% to 1.7%). Ten (3.1%) and five (3.1%) participants experienced virological failure, respectively. Comparisons by each dual‐drug versus CARs also show non‐inferiority.

One patient died in dual‐drug group. Grade 3/4 adverse events were reported in 39 (12.2%) and 15 (9.4%) participants, respectively (*p* = 0.36). There was no difference in mean change CD4 value at S96 (+70 vs. +65, *p* = 0.84).

**Conclusions**: Switching to dual maintenance therapy was non‐inferior to continuing CARs with 3 drugs regimen including TDF for maintaining virologic suppression on long‐term week 96 analysis and good safety profile. This first experience in West and Central Africa supports dual maintenance therapy as an option in sub‐Saharan African countries.

### OAB0104 Real‐world effectiveness of CAB+RPV LA in individuals with HIV viraemia at therapy initiation

R. Hsu ^1,2^, M. Sension^3^, J.S. Fusco^4^, L. Brunet^4^, B. Levis^4^, Q. Cochran^5^, G. Sridhar^6^, V. Vannappagari^6^, J. van Wyk^7^, M. Wohlfeiler^8^, G.P. Fusco^4^

^1^AIDS Healthcare Foundation, New York, United States, ^2^NYU Langone Medical Center, New York, United States, ^3^CAN Community Health, Fort Lauderdale, United States, ^4^Epividian, Inc., Raleigh, United States, ^5^AIDS Healthcare Foundation, Fort Lauderdale, United States, ^6^ViiV Healthcare, Durham, United States, ^7^ViiV Healthcare, London, United Kingdom, ^8^AIDS Healthcare Foundation, Miami, United States

**Background**: Cabotegravir + rilpivirine long‐acting injectable therapy (CAB+RPV LA) is indicated for people with HIV (PWH) who are virologically suppressed (HIV‐1 RNA viral load [VL] < 50 copies/ml) on a stable antiretroviral regimen. However, in the OPERA cohort, viraemic (VL ≥50 copies/ml) PWH have been observed initiating CAB+RPV LA. We describe these individuals and their outcomes in this real‐world setting.

**Methods**: Treatment‐experienced adults with viraemia switching to CAB+RPV LA from 21JAN2021‐31DEC2023 were followed through 29FEB2024. Complete initiation was defined as receiving two injections ≤67 days apart. Discontinuation was two consecutive missed injections (monthly: > 67 days without injection; every 2 months: > 127 days without injection). Non‐response (failure to suppress to < 50 copies/ml) and confirmed virologic failure (CVF; suppression to < 50 copies/ml followed by 2 VL ≥200 copies/ml or 1 VL ≥200 copies/ml + discontinuation) were assessed among those with ≥1 follow‐up VL.

**Results**: Of 3304 PWH initiating CAB+RPV LA, 368 (11%) initiated with viraemia (median VL: 120 copies/ml [IQR: 61, 2535]). Median age was 41 (IQR: 32, 51), 30% were female, 57% were Black, 18% were Hispanic and 63% received care in the Southern United States. Median years since HIV diagnosis was 9 (IQR: 3, 17), 29% had a BMI ≥30 kg/m^2^, median CD4 cell count was 578 cells/µl (IQR: 353, 808), 80% had ≥1 comorbidity, and 69% were on an INSTI and 13% on two core agents prior to CAB+RPV LA. Among 331 (90%) PWH who completed initiation, 258 individuals (78%) were on CAB+RPV LA with median follow‐up of 12 months (IQR: 8, 19) at the time of analysis, mostly (93%) dosed every 2 months. Of 313 PWH with ≥1 follow‐up VL, 277 (88%) suppressed to VL < 50 copies/ml during follow‐up, while 36 (12%) had non‐response during follow‐up. Fewer than 2% experienced CVF.

**Conclusions**: In the OPERA real‐world cohort, individuals with VL ≥50 copies/ml at CAB+RPV LA initiation had long‐standing HIV, a high prevalence of comorbidities and were failing an INSTI or multiple core agents. Most successfully initiated CAB+RPV LA and were able to suppress their VL to < 50 copies/ml. A majority remained on CAB+RPV LA at study end with few failures.

### OAB0202 Is long‐acting injectable antiretroviral therapy safe in countries with high hepatitis B prevalence?: Insights from the IMPALA study

U. Bahemuka ^1^, V. Ankunda^1^, V. Tumusiime^1^, P. Kafeero^1^, C. Norcross^1^, N. Garrett^2^, S. Mahomed^2^, S. Kassim^3^, L. Achieng Ombajo^4^, E. Laker Odongpiny^5^, N. Owarwo^5^, I. Yawe^6^, D. Grint^7^, A. Idahosa^8^, I. Eshun‐Wilsonova^9^, W. van Rein‐van der Horst van^10^, F. Addo Boateng^11^, F. Cresswell^7^, E. Ruzagira^7^

^1^MRC/UVRI & LSHTM, Viral Pathogens theme, Entebbe, Uganda, ^2^Centre for the AIDS Programme of Research in South Africa (CAPRISA), HIV pathogenesis and vaccine research, Durban, South Africa, ^3^Desmond Tutu HIV Foundation, Cape town, South Africa, ^4^University of Nairobi, Department of medicine, Nairobi, Kenya, ^5^Infectious Diseases Institute, Kampala, Uganda, ^6^Joint Clinical Research Centre, Fort Portal, Uganda, ^7^London School of Hygiene and Tropical Medicine, London, United Kingdom, ^8^Johnson & Johnson, Lagos, Nigeria, ^9^Johnson & Johnson, Cape town, South Africa, ^10^Johnson & Johnson, South Holland, Netherlands, the, ^11^Johnson & Johnson, Accra, Ghana

**Background**: Hepatitis B is a significant health concern among people living with HIV. The WHO recommended first‐line HIV regimen (tenofovir disoproxil, lamivudine, dolutegravir, TLD) both prevents and treats hepatitis B disease. Novel long‐acting (LA) injectable antiretrovirals, including cabotegravir (CAB) and rilpivirine (RPV), are not active against hepatitis B, raising concerns about reactivation in individuals with prior hepatitis B exposure. In addition, CAB+RPV LA HIV treatment is not recommended in individuals with active hepatitis B (HepBsAg+).

**Methods**: IMPALA (NCT05546242) is a phase 3b clinical trial in Uganda, Kenya and South Africa investigating efficacy of CAB+RPV LA in virally suppressed adults. Participants with active hepatitis B (HepBsAg+) or prior infection without immunity (HepBcAb+ with HepBsAb < 10 IU) were excluded. Eligible participants were randomized 1:1 to continue TLD or switch to CAB+RPV LA given 2 monthly. Regular assessments were conducted to monitor liver enzyme elevations and hepatitis infection/reactivation, with comparisons made between arms.

**Results**: Between December 2022 and March 2024, 845 volunteers were screened, of whom 222 (26.3%) had evidence of prior infection (HepBcAb+). Hepatitis B‐related reasons precluded 84 (9.9%) from randomization (29 [3.4%], active hepatitis B; 55 [6.5%], prior infection but no immunity). Among 540 randomized participants, 42 (7.8%) had evidence of vaccine‐mediated immunity. ALT elevations were observed in seven (2.6%) participants in the TLD arm versus two (0.7%) in the LAI arm. One incident hepatitis B infection occurred in a participant in the LA arm without vaccine‐mediated immunity. No reactivation of hepatitis B was detected during the study.

**Conclusions**: The high number of hepatitis B‐related screen‐outs highlights challenges for LA therapy in hepatitis B endemic countries. While hepatitis B reactivation did not occur in individuals with immunity (HepBsAb ≥ 10 IU), the risk of de novo infection remains in those receiving CAB+RPV LA without immunity. This demonstrates the need for strengthened vaccination efforts, particularly among those switching to CAB+RPV LA.[Fig jia226518-fig-0006]

**Figure 1 jia226518-fig-0006:**
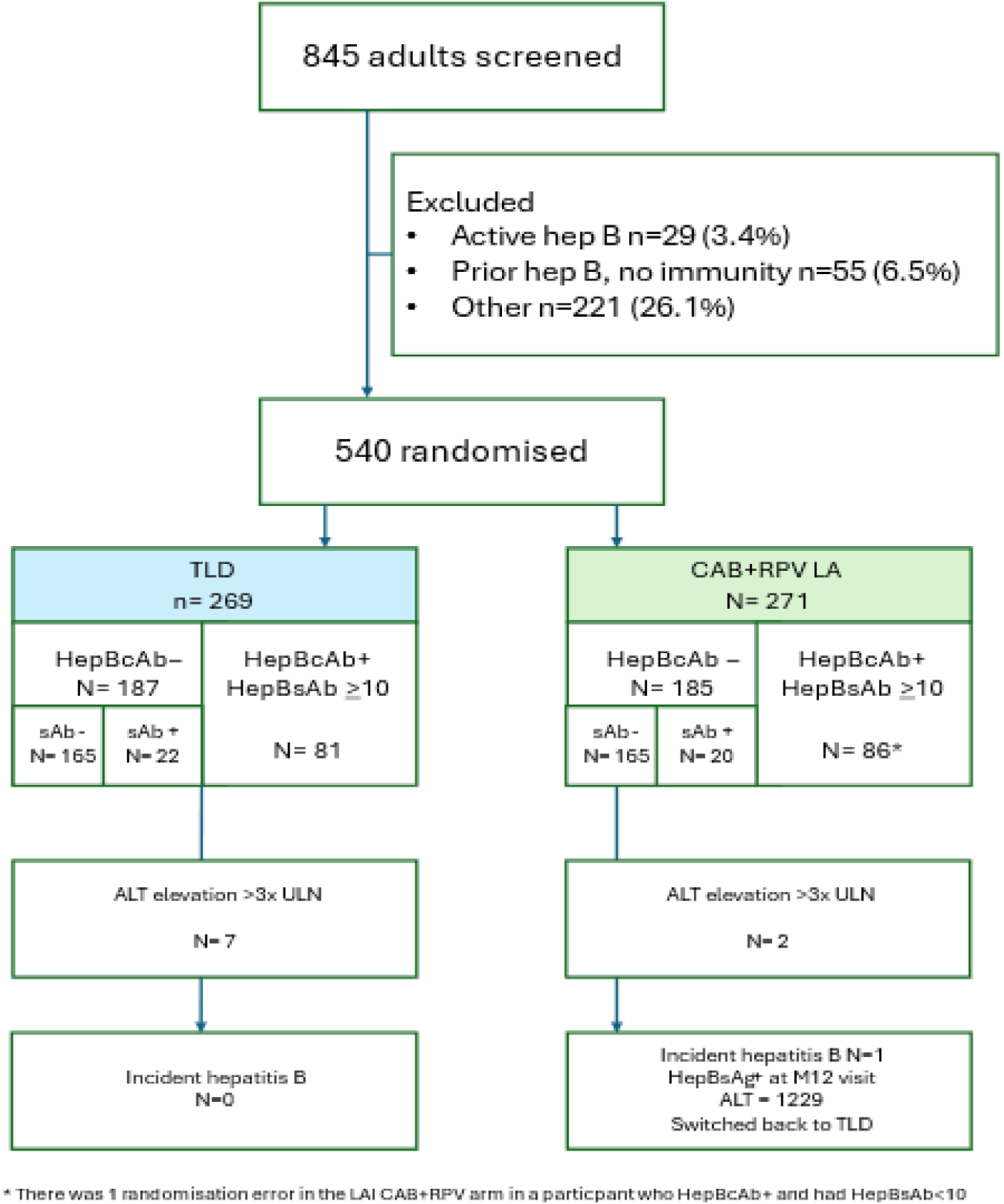
OAB0202

### OAB0203 Topical trichloroacetic acid versus electrocautery for the treatment of anal intraepithelial neoplasia in patients living with HIV: a multicentre randomized non‐inferiority trial (TECAIN‐study)

S. Esser ^1^, A. Kreuter^2^, A. Potthoff^3^, K. Bilbilis^4^, M. Oette^5^, U. Wieland^6^

^1^University Hospital Essen, University Duisburg‐Essen, Department of Dermatology and Venereology, Essen, Germany, ^2^Helios St. Elisabeth Hospital Oberhausen; University Witten/Herdecke, Department of Dermatology, Venereology and Allergology, Oberhausen, Germany, ^3^St. Elisabeth Hospital, Ruhr University Bochum, Interdisciplinary Immunological Outpatient Clinic, Department of Dermatology, Venereology and Allergology, Bochum, Germany, ^4^University Hospital Essen, University Duisburg‐Essen, Centre for Clinical Studies, Institute for Medical Informatics, Biometry and Epidemiology, Essen, Germany, ^5^Augustinerinnen Hospital Cologne, Department of Internal Medicine, Gastroenterology and Infectious Diseases, Cologne, Germany, ^6^University of Cologne, Faculty of Medicine and University Hospital Cologne, Institute of Virology, National Reference Center for Papilloma‐and Polyomaviruses, Cologne, Germany

**Background**: People living with HIV (PLWH) have a strongly increased risk for human papillomavirus (HPV)‐associated anal cancer compared to the general population. Therefore, screening for and treatment of anal intraepithelial neoplasia (AIN) for the prevention of anal cancer are recommended for PLWH. Currently, there is no gold standard for the treatment of AIN and the available options have rarely been evaluated in prospective randomized trials.

**Methods**: The TECAIN‐study was a prospective, multicentre, randomized, non‐inferiority trial investigating the efficacy and safety of TCA versus ECA for the treatment of AIN. PLWH with histologically confirmed AIN were recruited from seven anal dysplasia units in Germany between 2015 and 2020. The primary endpoint (PE) was therapeutic success defined as a combination of complete clinical response evaluated by high‐resolution anoscopy and histological resolution/regression of AIN 4 weeks after the end of treatment (4 weeks follow‐up, 4WFU). Secondary endpoints comprised therapeutic success 24 weeks after the end of treatment (24WFU), adverse events (AE) and HPV parameters (prevalence, multiplicity, DNA load, oncogene mRNA expression) at 4WFU and 24WFU.

**Results**: Two hundred and fifty‐seven PLWH with AIN were enrolled and randomly assigned to TCA (*n* = 129) or ECA (*n* = 128). One hundred and eighteen patients were treated with TCA, 115 with ECA (intention‐to‐treat population). The PE was reached in 52.5% of the TCA‐group and in 61.7% of the ECA‐group. Histological resolution/regression was documented in 66.9% (TCA‐group) and 67.0% (ECA‐group) at 4WFU. While the non‐inferiority of TCA could not be shown at 4WFU, TCA was non‐inferior to ECA at 24WFU (50.8% vs. 48.7% therapeutic success). Treatment‐related AEs were reported in 64.4% of the TCA‐ and in 65.2% of the ECA‐ group until 4WFU, without any persistent conditions. HPV parameters did not differ significantly between treatment groups and within the groups between baseline and 4WFU or 24WFU.

**Conclusions**: TCA is a well‐tolerated, inexpensive and simple to use treatment option for AIN in PLWH.

### OAB0204 Predictors of weight gain among people with HIV (PWH) over 3‐year period

R. Elion ^1^, J. Gruber^2^, J. Radtchenko^1^, M. Dunbar^2^, N. Prata Menezes^2^, J. Eron^3^, C. Cohen^2^, G. McComsey^4^, P. Sax^5^, G. Moyle^6^

^1^Trio Health, Louisville, United States, ^2^Gilead Sciences, Foster City, United States, ^3^University of North Carolina at Chapel Hill, Chapel Hill, United States, ^4^Case Western Reserve University, Cleveland, United States, ^5^Brigham and Women's Hospital, Boston, United States, ^6^Chelsea and Westminster Hospital, London, United Kingdom

**Background**: Trio previously reported no difference in 3‐year mean weight change in virally suppressed (VS) PWH versus non‐PWH after propensity score matching. This study assessed the effects of ART on weight gain among PWH.

**Methods**: Retrospective study using Trio Health HIV EMR data (US). Eligibility: ≥18 years, in care January 2015−August 2023, treatment‐experienced VS (viral load < 200 copies/ml) at baseline or on first ART for ≥6 months and ≥12 months since start, VS at 3 years; with baseline and 3‐year weights. Baseline characteristics were compared (Χ‐square, *t*‐test) for PWH with gain < 5% versus gain ≥10% and either shift to higher body mass index (BMI) or baseline obese (10% gain + BMI shift). Classification regression trees (CRTs) and logistic regression (LR) identified predictors of 10% gain + BMI shift including ART and baseline demographic and clinical characteristics.

**Results**: Of 10,413 PWH, 41% lost/did not gain weight, 26% gained < 5%, 12% had ≥ 10% gain + BMI shift. Compared to PWH with gain < 5%, those with 10% gain + BMI shift were younger (median age 44 vs. 51 years), more likely female (28% vs. 16%), Black (49% vs. 37%), with normal BMI (39% vs. 30%), substance use (12% vs. 7%) and less likely with cardiovascular disease (23% vs. 28%), and hyperlipidaemia (8% vs. 15%); 88% received INSTI. In CRT, younger PWH, female, Black, with lower baseline BMI and CD4 were predictors of 10% gain + BMI shift. In LR, Black, female, age 18−29, normal baseline BMI and CD4 < 200 cells/mm^3^, no TAF/TDF exposure 12 months pre‐baseline were associated with 10% gain + BMI shift (Figure 1).

**Figure 1:**
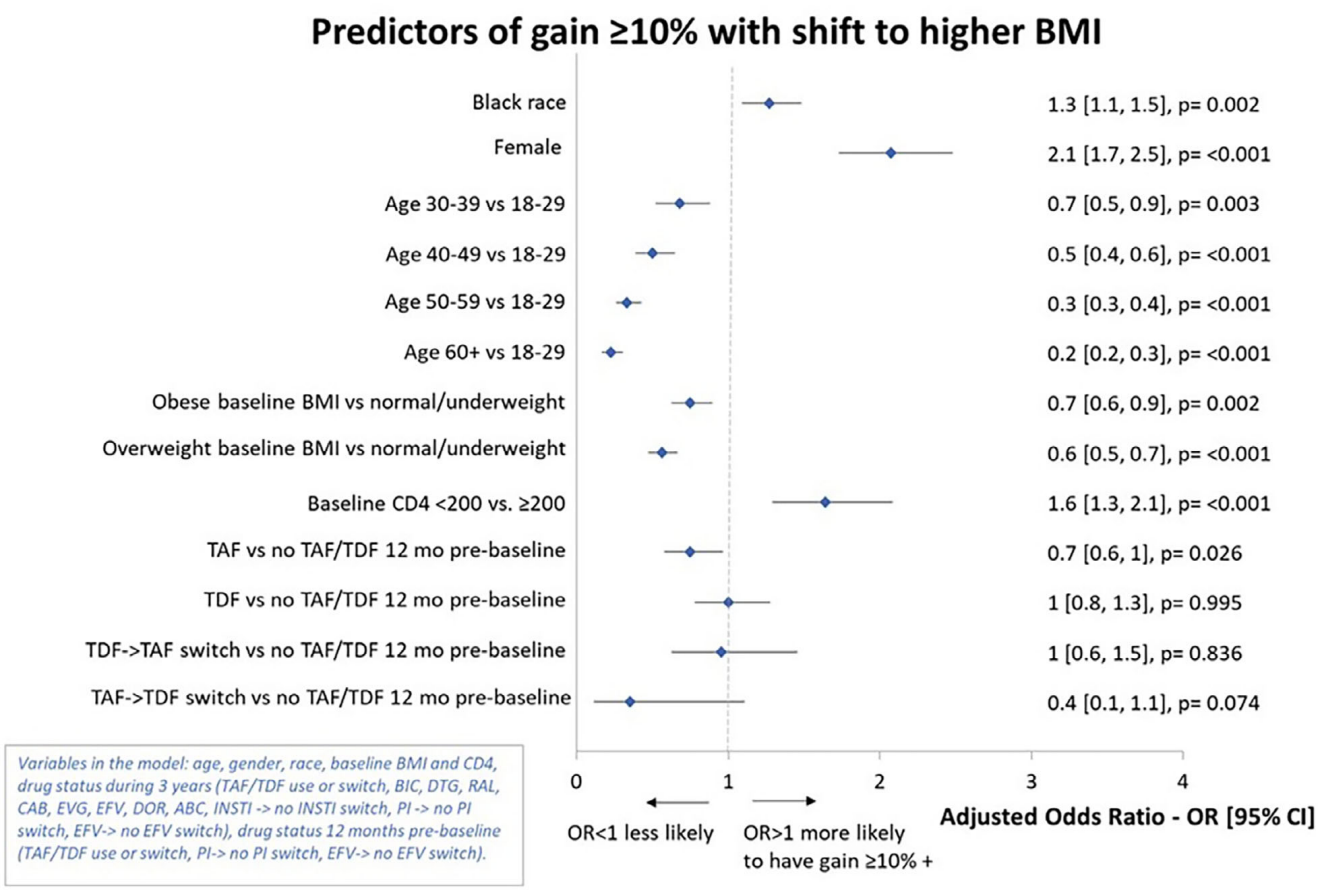
OAB0204

**Conclusions**: This is the largest study evaluating weight change over 3‐year period among VS PWH. Among gainers, we observed limited impact of ART on ≥10% weight gain and socio‐demographic and clinical predictors were similar to non‐PWH findings from the prior study.

### OAB0205 Prevalence and correlates of sleep apnoea using WatchPAT™: Chicago Women's Interagency HV Study (WIHS)

K. Weber ^1^, E. Daubert^2^, R. Hernandez^2^, D. Johnson^2^, R. Morack^2^, R. Ross^3^, M. Cohen^2^, A. French^4^

^1^Hektoen Institute of Medical Research, Chicago, United States, ^2^Hektoen Institute, Chicago, United States, ^3^Rush University Medical Center, Chicago, United States, ^4^University of Illinois, Chicago, United States

**Background**: Obstructive sleep apnoea (OSA) is often undiagnosed due to cumbersome lab‐based diagnostics. Midlife women with/without HIV (WWH/WWoH) have OSA risks, making home sleep apnoea testing (HSAT) desirable.

**Methods**: Chicago WIHS women had sleep apnoea assessed (2019−2024) using an overnight multi‐sensor, wrist‐worn HSAT device with finger‐probe peripheral arterial tonometry (WatchPAT™). Validated automated algorithms determined apnoea‐hypopnea index (AHI) using ≥ 3% oxygen desaturation threshold and categorized OSA severity based on number of hourly apnoea‐hypopnea events: none (< 5), mild (5−14), moderate (15−30), severe (> 30). Bivariate and multivariable logistic regression models controlling for age, income, education, employment, menopausal and HIV status determined factors associated with odds of moderate/severe versus none/mild OSA.

**Results**: One hundred and sixty‐nine (63% WWH) without previously diagnosed OSA had valid AHI (26 were invalid due to technical issues or < 3 hours sleep); 83.4% were African American with median (IQR) age in years 54.6 (47.6, 60.2); 73.2% postmenopausal; 67.5% ≤$18,000 household income; 64% unemployed; 64.5% completed ≤ high school. Current substances use included: 18.5% crack/cocaine/heroin, 25% cannabis and 46.7% smoke tobacco. Chronic conditions included: 58.9% obesity, 25.6% diabetes and 72.8% hypertension; 52.4% reported poor sleep quality (Pittsburgh Sleep Quality Index > 5); 28.7% used sleep medications (prescribed/non‐prescribed). Mean/standard deviation (m/sd) body mass index (BMI) and waist circumference (WC) were 32.9 (8.6) and 103.4 (18.1), respectively. Overall, 151 (89.4%) had any degree of OSA; 24.9% severe, 34.3% moderate, 30.2% mild. Only 10.6% had no sleep apnoea per guidelines. There were no significant differences by HIV status. In bivariate analyses (none/mild vs. moderate/severe), lower income ≤$18,000 (58.8% vs. 73.5%, *p* = .048), higher m(sd)BMI (29.7 [6.9] vs. 35.1 [9.0], *p* < .0001), larger m(sd)WC (96.8 [14.8] vs. 107.8 [18.9], *p* < .0001) and being obese (43.5% vs. 69.7%, *p* = .0008) were associated with OSA severity. In adjusted logistic regression models, only higher mBMI (aOR 1.11 [1.05−1.16; *p* < .0001]) and larger mWC (aOR 1.04 [1.02−1.06; *p* = .0002]) were associated with increased odds of moderate/severe OSA. Substance use and other chronic conditions were not significantly associated with OSA severity.

**Conclusions**: Undiagnosed sleep apnoea was highly prevalent, nearly 90% among midlife WWH/WWoH, with 59% classified as moderate/severe OSA. HSAT screening is warranted to identify women likely to benefit from further evaluation and treatment by a sleep specialist.

### OAB0302 Predictors of treatment failure in children living with HIV starting first‐line antiretroviral therapy in the ODYSSEY trial

J. Wyncoll ^1^, M. Archary^2^, H.A. Mujuru^3^, A.R. Kekitiinwa^4^, A. Violari^5^, S. Na‐Rajsima^6^, A. Lugemwa^7^, E. Variava^5^, M. Cotton^8^, C.M. Kityo^9^, T. Puthanakit^10^, C. Königs^11^, O. Behuhuma^12^, S. Welch^13^, Y. Saïdi^14^, L. Marques^15^, G.M. Ahimbisibwe^16^, L. Holden^1^, C. Giaquinto^17^, P. Rojo^18^, D.M. Gibb^1^, A. Turkova^1^, E. White^1^, D. Ford^1^, and the ODYSSEY Trial Team

^1^University College London, MRC Clinical Trials Unit, Institute of Clinical Trials and Methodology, London, United Kingdom, ^2^Enhancing Care Foundation, King Edward VIII Hospital, Durban, South Africa, ^3^University of Zimbabwe, Harare, Zimbabwe, ^4^Baylor College of Medicine Children's Foundation, Kampala, Uganda, ^5^University of the Witwatersrand, Perinatal HIV Research Unit, Johannesburg, South Africa, ^6^Mahasarakam Hospital, Pediatric Department, Maha Sarakham, Thailand, ^7^Joint Clinical Research Centre (JCRC), Mbarara, Uganda, ^8^Stellenbosch University, Family Center for Research with Ubuntu, Department of Paediatrics & Child Health, Tygerberg, South Africa, ^9^Joint Clinical Research Centre (JCRC), Kampala, Uganda, ^10^HIVNAT, Thai Red Cross AIDS Research Center, Bangkok, Thailand, ^11^Goethe University Frankfurt, Department of Paediatrics and Adolescent Medicine, Frankfurt, Germany, ^12^Africa Health Research Institute (AHRI), Kwazulu‐Natal, South Africa, ^13^University Hospitals Birmingham NHS Foundation Trust, Birmingham, United Kingdom, ^14^INSERM/ANRS SC10‐US19, Essais thérapeutiques et maladies Infectieuses, Villejuif, France, ^15^Centro Materno‐infantil do Norte, ULSSA, Porto, Portugal, ^16^Makerere University‐Johns Hopkins University (MU‐JHU) Research Collaboration, Kampala, Uganda, ^17^University of Padova, Department of Women and Child Health, Padova, Italy, ^18^Hospital 12 de Octubre, Pediatric Infectious Diseases Unit, Madrid, Spain

**Background**: There are few data on predictors of treatment failure in children starting antiretroviral therapy (ART) on dolutegravir‐based regimens (DTG).

**Methods**: The ODYSSEY randomized trial demonstrated superior efficacy for DTG versus standard‐of‐care (SOC). The prognostic value of baseline characteristics across four domains (anthropometrics, HIV indicators, haematology, demographics) for treatment failure by 96 weeks in children starting first‐line ART was investigated using Cox models, adjusting for trial arm and weight; fractional polynomials were considered for continuous covariates.

**Results**: Three hundred and eighty‐one children started first‐line ART (82% Africa, 13% Thailand, 5% Europe), 309 weighing ≥14 kg (154 DTG, 155 SOC [92% efavirenz‐based]) and 72 < 14 kg (35 DTG, 37 SOC [78% boosted lopinavir‐based]). At baseline, median age was 10.5 years (IQR: 6.5−14.0), CD4% 20% (IQR: 12−28), BMI‐for‐age z‐score −0.58 (IQR: −1.48 to +0.25; 15%<−2) and 73 (19%) children had ongoing WHO Stage 3/4 events. Seventy‐five children experienced treatment failure by 96 weeks (24 DTG, 51 SOC). DTG‐based ART protected against risk of treatment failure, and risk declined as weight increased, particularly in children < 14 kg. Within domain‐specific models, after adjustment for weight and trial arm, low BMI‐for‐age, low CD4%, an ongoing WHO Stage 3/4 event, high neutrophils and being at an African site independently predicted treatment failure (*p* < 0.2). Age and middle‐upper arm circumference were collinear with weight, and CD4/CD8 ratio with CD4%. Log viral load, haemoglobin, platelets, sex and caregiver were dropped due to lack of prediction (*p*≥0.2). In a full multivariable model, DTG‐based ART, weight, CD4%, ongoing WHO Stage 3/4 event and region were retained (*p* < 0.1); neutrophils were not retained after adjustment for ongoing WHO Stage 3/4 event, and BMI‐for‐age was not retained after adjustment for CD4% and region. There was no evidence that predictors differed between trial arms (interactions *p*> 0.1).[Table jia226518-tbl-0001]

**Table 1 jia226518-tbl-0001:** OAB0302

Baseline predictors of treatment failure in children starting ART	​Univariable model (adjusting for weight and trial arm)			Multivariable model		
	HR	95% CI	*p*‐value*	HR	95% CI	*p*‐value*​
**DTG** (vs. SOC)	0.44	(0.27−0.72)	0.001	0.45	(0.27−0.72)	0.001
**Weight**	**	**	<0.001	**	**	<0.001
**BMI‐for‐age z‐score** (per 1 unit increase)	0.77	(0.66−0.91)	0.002	–	–	–
**CD4 percentage** (per 1% increase)	0.97	(0.95−0.99)	0.002	0.97	(0.95−0.99)	0.007
**Ongoing WHO Stage 3/4 event**	1.94	(1.19−3.17)	0.01	1.57	(0.94−2.62)	0.09
**Neutrophil count** (per 1 cell/mm^3^ increase)	1.17	(1.05−1.31)	0.01	–	–	–
**Region** (African vs. non‐African site)	2.07	(0.81−5.25)	0.09	2.09	(0.82−5.31)	0.09

*All *p*‐values are based on likelihood ratio tests.

**Weight was transformed using the reciprocal (1/weight) based on the best‐fitting fractional polynomial. In the multivariable model, the HR for risk of outcome in a child weighing 14 kg versus 4 kg was 0.83 (95% CI 0.71−0.97), and in a child weighing 24 kg versus 14 kg was 0.97 (0.95−0.99).

**Conclusions**: Children living with HIV starting ART would benefit from screening for ongoing WHO Stage 3/4 events and CD4 testing to identify those at high risk of treatment failure, with low‐weight/younger children particularly vulnerable.

### OAB0303 Prevalence of suicidal behaviour and its associated factors among young people living with HIV accessing care at a primary healthcare centre in Zambia

S. Simwanza ^1^, B. Tapisha^1^, D. Ngosa^2^, L. Sigande^3^, C. Mulungu^4,5^, O. Mwenya^6^, J. Mwamba^1^, L. Moyo^5^, C. Moyo^7^, T. Mbambara^5^, G. Lungu^8^, M. Mbewe^9^, G. Moonga^10^, K. Shanaube^1^, B. Maila^5,11,12^

^1^Zambart, Research, Lusaka, Zambia, ^2^CIDRZ, Analysis Unit, Lusaka, Zambia, ^3^Zambart, Data, Lusaka, Zambia, ^4^University of Lusaka, Lusaka, Zambia, ^5^Ministry of Health, Lusaka, Zambia, ^6^Serenity Harm Reduction Programme Zambia, Lusaka, Zambia, ^7^Lifeline Childline Zambia, Lusaka, Zambia, ^8^Ministry of Health, Clinical Care and NCD Unit, Lusaka, Zambia, ^9^Seed Global Health, Lusaka, Zambia, ^10^University of Zambia, School of Public Health, Department of Epidemiology and Biostatistics, Lusaka, Zambia, ^11^University of California, San Diego, Department of Psychiatry, San Diego, United States, ^12^San Diego State University, School of Social Work, San Diego, United States

**Background**: Adolescents and young people living with HIV (AYPLHIV) are more likely to exhibit suicidal behaviour than their HIV‐uninfected peers. Up to half of AYPLHIV in sub‐Saharan Africa struggle with mental health disorders (MHDs) associated with suicidal behaviour and most remain undiagnosed. While piloting integrated MHD screening using a locally co‐developed digital application in routine HIV care, we determined the prevalence of suicidal behaviour and its associated factors among AYPLHIV.

**Methods**: We conducted a cross‐sectional study among AYPLHIV aged 15−24 years at a primary healthcare centre (PHC) in Lusaka, completely enumerating AYPLHIV in the hospital antiretroviral therapy (ART) register from September 2023 to May 2024. We measured suicidal behaviour using the Ask Suicide Questionnaire Toolkit administered by a research assistant at the PHC.

Descriptive statistics, including frequencies and proportions, were used to determine prevalence, while logistic regression models identified factors associated with suicidal behaviour.

**Results**: We reached 432/583 (74.1%) AYPLHIV in the ART register, 416/432 (96.3%) consented, with 268/416 (64.4%) females and a median age of 20 (IQR: 18−23). The prevalence of suicidal behaviour was 127/416 (30.5%; [95% CI: 26.1%−35.2%]) overall, higher among females (34.3%) than among males (23.6%).

Being female (AOR 1.69; 95% CI 1.18, 3.32; *p* = 0.009), feeling stigmatized because of HIV (AOR 1.54; 95% CI 0.89, 2.65; *p* = 0.120), having stigmatizing attitudes towards people living with HIV (AOR 1.75; CI 95% 1.08, 2.83; *p* = 0.022), having a positive PHQ 9 depression screen (AOR 3.02; CI 95% 1.63, 5.60; *p* < 0.001) and positive GAD 7 anxiety screen (AOR 2.61; CI 95% 1.60, 4.27; *p* < 0.001) increased the odds of exhibiting suicidal behaviour.

Conversely, compared to living alone, living with parents (AOR 0.33, CI 95% 0.13, 0.83, *p* = 0.018), a partner (AOR 0.32; CI 95% 0.11, 0.98; *p* = 0.046) or any other relative (AOR 0.29; CI 95% 0.11, 0.78; *p* = 0.014) was protective against suicidal behaviour.

**Conclusions**: Suicidal behaviour is prevalent among AYPLHIV in Lusaka, strongly influenced by stigma, common MHDs and socio‐demographic factors. It is feasible to integrate MHD and suicidal behaviour screening into routine HIV care among AYPLHIV. Comprehensive suicide prevention interventions addressing the drivers of suicidal behaviour among AYPLHIV should be explored.

### OAB0304 Electronic monitoring device adherence intervention improves viral suppression in young people living with HIV at risk of virological failure

N.V. Dzavakwa ^1,2^, C. Mackworth‐Young^1,2^, T. Bandason^1^, N. Redzo^1^, M. Paradza^1^, M. Chidembo^1^, D. Kembo^1^, D. Munemo^1^, M. Makamba^3^, R.A. Ferrand^1,2^, P.Y. Khan^2^, V. Simms^1,2^

^1^Biomedical Research and Training Institute, The Health Research Unit Zimbabwe, Harare, Zimbabwe, ^2^London School of Hygiene and Tropical Medicine, London, United Kingdom, ^3^Centre for Sexual Health and HIV AIDS Research Zimbabwe, Harare, Zimbabwe

**Background**: Adherence to antiretroviral therapy (ART) among young people living with HIV (YPLWH) is lower than in other age groups. We conducted an individually randomized controlled trial to investigate the impact of an electronic monitoring device (EMD) paired with text message reminders and monthly adherence feedback on HIV viral suppression among YPLWH with suboptimal adherence in Harare, Zimbabwe.

**Methods**: YPLWH aged 16−30 years taking ART with viral load (VL) > 200 copies/ml were recruited from five HIV clinics and randomized 1:1 to intervention or standard of care (existing enhanced adherence counselling). The EMD (Wisepill) was used for 6 months, and was configured to send SMS reminders if not opened within 30‐minutes of scheduled ART intake time. Monthly individualized in‐person feedback sessions were conducted using EMD data. The primary outcome was proportion of participants with VL< 50 copies/ml at 6 months. A mixed methods process evaluation was conducted concurrently to understand fidelity, feasibility and acceptability of the intervention.

**Results**: Two hundred and five YPLWH (101 randomized to intervention, mean age 22.6 [SD 3.8] years; 52% female) were enrolled between September 2023 and April 2024 with no significant differences in participant baseline characteristics between the two arms. Median VL at baseline was 12,700 copies/ml (IQR 1020, 105,686); 143 (69.8%) were on a dolutegravir regimen and 62 (30.2%) on an atazanavir regimen. Eighty‐seven (42.4%) had interrupted ART for ≥2 days in the 3 months preceding enrolment, the longest interruption being > 4 weeks (*n* = 17). Overall, 194 participants had a 6‐month VL (10 withdrew, 1 died) with a median VL of 104 copies/ml (IQR 19, 21,762); 85/194 (43.8%) were virally suppressed. At 6 months, 51.0% (*n* = 49/96) in the intervention versus 36.7% (*n* = 36/98) in the standard of care arm had a VL < 50 copies/ml (OR 1.80, 95% CI: 1.01, 3.19, *p* = 0.045). Adjusting for baseline VL and site, corresponding OR was 1.76 (95% CI: 0.97−3.19, *p* = 0.061). Qualitative findings showed that EMD‐supported adherence monitoring enabled YPLWH to have open and supportive conversations about adherence, which were otherwise difficult.

**Conclusions**: This EMD‐supported adherence intervention was feasible and acceptable and improved viral suppression among YPLWH at risk of virological failure in an age group where adherence has been a persistent challenge.

### OAB0305 Pharmacokinetic modelling to support WHO‐weight band dosing of the new paediatric darunavir/ritonavir (120/20 mg) fixed‐dose combination tablet for children

S. Abdalla^1^, M. Lallemant^2^, A. Compagnucci^3^, A. Nardone^4^, C. Giaquinto^5^, D. Gibb^6^, C. Chabala^7^, D. Burger^8^, D. Hirt^1,9^, T.R. Cressey ^2^, UNIVERSAL‐2 Study Team

^1^Hôpital Cochin, APHP centre, Université Paris cité, Service de pharmacologie Clinique, Paris, France, ^2^Chiang Mai University, AMS‐PHPT Collaboration, Chiang Mai, Thailand, ^3^INSERM, SC10‐US019 Essais Thérapeutiques et Maladies Infectieuses, Villejuif, France, ^4^Fondazione Penta ETS, Padova, Italy, ^5^Fondazione Penta ETS, Padova, Italy, ^6^University College London, Medical Research Council Clinical Trials Unit, London, United Kingdom, ^7^University of Lusaka, Department University Teaching Hospital, Lusaka, Mozambique, ^8^Radboud University Medical Center, Department of Pharmacy, Radboudumc Research Institute for Medical Innovation, Nijmegen, Netherlands, the, ^9^INSERM 1343, Université Paris cité, Pharmacologie et évaluation des thérapeutiques chez l'enfant et la femme enceinte, Paris, France

**Background**: Darunavir‐boosted with ritonavir (DRV/r) is an HIV protease inhibitor used in children ≥3 years old but no child‐friendly fixed‐dose combination (FDC) is available. Following prioritization by the WHO‐PADO group, a 120/20 mg DRV/r FDC tablet was developed (Laurus Labs), in collaboration with Penta and CHAI under the framework of the Global Accelerator for Paediatric Formulations (GAP‐f). We performed a pharmacokinetic (PK) modelling/simulation study to support DRV/r dosing using this FDC once‐(OD) and twice‐(BID) daily per WHO‐weight bands for children ≥3 years old weighing 10 to < 35 kg.

**Methods**: Darunavir plasma concentration data from three clinical trials in children living with HIV (DELPHI, ARIEL and CHAPAS‐4 studies) were combined to develop a population PK model. Simulations were performed in a virtual population of children receiving DRV/r OD and BID dosing regimens to determine the dose that would achieve geometric mean (GM) DRV exposure (AUC_0‐tau_) in each WHO‐weight band within 80%−130% of the value reported in adults.

**Results**: DRV/r concentration data from 162 children aged 3−16 years old were included. DRV PK was described using a 2‐compartment model with first‐order absorption (lag‐time for DRV tablets vs. suspension) and linear elimination. Clearance and volume of distribution parameters were allometrically scaled to body weight, and alpha1‐glycoprotein concentrations inversely influenced DRV clearance. Simulations predicted the following DRV/r doses achieved target exposures: DRV/r OD: 480/80 mg (4 tablets) from 10 to < 14 kg and 600/100 mg (6 tablets) from 14 to < 35 kg. DRV/r BID: 240/40 mg (2 tablets) from 10 to < 14 kg; 360/60 mg (3 tablets) from 14 to < 25 kg; and 480/80 mg (4 tablets) from 25 to < 35 kg (Figure 1).

**Figure 1:**
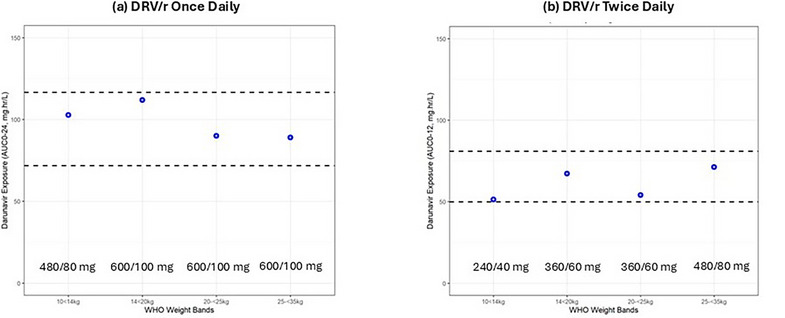
**OAB0305: Predicted GM DRV AUC_0‐tau_, in children 10 to < 35 kg receiving DRV/r FDC (120/10 mg) per WHO weight bands** (a) once daily and (b) twice daily. Dotted lines represent target 80‐130% range of the GM reported in adults.

**Conclusions**: The model‐based dosing of the new paediatric DRV/r FDC tablets supports once‐ and twice‐daily dosing according to WHO weight bands in children aged ≥3 years and weighing 10 to < 35 kg.

### OAB0402 Pregnancy and neonatal outcomes following prenatal exposure to cabotegravir (CAB): data from the Antiretroviral Pregnancy Registry (APR)

V. Vannappagari ^1^, J. Albano^2^, L. Ragone^1^, A. Scheuerle^3^, L. Mofenson^4^, W. Short^5^, C. Thorne^6^, N. Carneal‐Frazer^2^, T. Cook^2^, C. Zhang^2^, K. Brown^1^, A. de Ruiter^7^

^1^ViiV Healthcare, Durham, United States, ^2^Syneos Health, Morrisville, United States, ^3^University of Texas Southwestern Medical Center, Dallas, United States, ^4^Elizabeth Glaser Pediatric AIDS Foundation, Silver Spring, United States, ^5^The Perelman School of Medicine, University of Pennsylvania, Philadelphia, United States, ^6^University College London Great Ormond Street Institute of Child Health, London, United Kingdom, ^7^ViiV Healthcare, London, United Kingdom

**Background**: Cabotegravir (CAB) is indicated for the treatment of HIV‐1 infection in combination with rilpivirine and as a single agent for prevention of HIV. There are limited human data on the use of CAB during pregnancy to adequately assess effect on pregnancy outcomes.

**Methods**: The APR is a prospective, international exposure‐registration cohort study, monitoring for early warning signals of major teratogenic effects of antiretrovirals (ARVs) used during pregnancy. This descriptive analysis assesses pregnancy and neonatal outcomes including birth defects among infants with prenatal exposure to CAB using APR data through 31 July 2024.

**Results**: Forty‐two reported pregnancies with exposure to CAB (28 [66.7%] as CAB for treatment and 14 [33.3%] as CAB for pre‐exposure prophylaxis [PrEP]) resulted in 43 outcomes including 35 (81.4%) live births, 1 (2.3%) stillbirth, 3 (7.0%) spontaneous abortions and 4 (9.3%) induced abortions (Table 1). Among live births, one (2.9%) reported a birth defect (congenital ptosis). Among 33 singleton, live births without defects, five (15.2%) were preterm and six (18.2%) had low birth weight (LBW) including three (9.1%) very LBW. Of the 42 CAB‐exposed pregnancies, 39 had CAB pre‐conception exposure (27 with earliest exposure 0−6 months prior to pregnancy and 12 with earliest exposure 6−12 months prior to pregnancy) and three had earliest exposure during pregnancy (one each in the first, second and third trimesters). For the 41 pregnancies where route of administration was known, 38 (92.7%) were exposed to injectable CAB, while three (7.3%) were exposed to oral CAB.

**Table 1:** OAB0402: Pregnancy and neonatal outcomes of pregnant individuals exposed to CAB using APR data through 31 July 2024

Total Pregnancy Outcomes	43
Live Births	35 (81.4)
Stillbirths	1 (2.3)
Spontaneous Abortions	3 (7.0)
Induced Abortions	4 (9.3)
Total Live Births	35
Birth Defect Cases	1 (2‐9)
Total Singleton, Live Births without Defects	33
Gestational Age > = 37 weeks	27 (81.8)
Gestational Age < 37 weeks (preterm)	5 (15.2)
Missing	1 (3.0)
Birth Weight > = 2500 grams	22 (66.7)
Birth Weight < 2500 grams (LBW)	6 (18.2)
Birth Weight < 1500 grams (very LBW)	3 (9.1)
Missing	5 (15.2)

**Conclusions**: While no significant safety concerns were seen, definitive conclusions on the safety of CAB use in pregnancy cannot be drawn due to a limited number of pregnancies reported and the data should be interpreted with caution. Providers are encouraged to register ARV‐exposed pregnancies, especially those exposed to newer ARVs such as CAB, at APR (apregistry.com).

### OAB0403 Determinants of increased antenatal viral load and its association with pregnancy adverse outcomes in pregnant women living with HIV

A. Mendes Muxlhanga ^1^, A. Figueroa‐Romero^2^, G. Mombo‐Ngoma^3^, J. Mischlinger^4^, M. Mazuze^5^, M. Esen^6^, A. Tchouatieu^7^, L. Stoeger^8^, E. Sevene^9^, N. Bortella^2^, C. Menendez^2^, T. Nhampossa^1^, R. González^8^

^1^Manhiça Health Research Center, Maternal and Child Health, Manhiça, Mozambique, ^2^Barcelona Institute for Global Health, Maternal and Child Health Initiative, Barcelona, Spain, ^3^Centre de Recherches Médicales de Lambaréné, Lambaréné, Gabon, ^4^German Center for Infection Research, partner site Hamburg‐Lübeck‐Borstel‐Riems and Tübingen, Hamburg‐Lübeck‐Borstel‐Riems, Germany, ^5^Manhiça Health Research Center, Manhiça, Mozambique, ^6^. German Center for Infection Research, partner site Hamburg‐Lübeck‐Borstel‐Riems and Tübingen, Hamburg‐Lübeck‐Borstel‐Riems, Germany, ^7^Medical University of Vienna, Department of Medicine I, Division of Infectious Diseases and Tropical Medicine, Vienna, Austria, ^8^Barcelona Institute for Global Health, Maternal and Child Health Initiative, Barcelona, Spain, ^9^Centro de Investigação em Saúde de Manhiça, Maternal and Child Health, Manhiça, Mozambique

**Background**: An estimated 1.3 million HIV‐positive women are pregnant annually. Without any intervention, the mother‐to‐child HIV transmission rate ranges from 15% to 45%, with maternal HIV viral load being the strongest risk factor. This study investigated the determinants of elevated antenatal viral load and its association with adverse pregnancy outcomes in African women.

**Methods**: This is a secondary analysis of data collected in a randomized controlled trial conducted in Gabon and Mozambique (2019−2023) evaluating dihydroartemisinin‐piperaquine for malaria prevention in HIV‐positive pregnant women. Pregnant women attending their first antenatal care visit were enrolled and monitored until delivery. Descriptive statistics, and bivariate and multivariable logistic regression analyses were conducted to identify factors associated with high viral load and its association with adverse pregnancy outcomes.

**Results**: Among 666 enrolled women, 35% (*n* = 232) presented with high viral load (> 150 copies/ml). Anaemia at baseline (OR 1.75, 95% CI 1.18−2.59, *p* = 0.005) and delayed antenatal care antiretroviral therapy (ART) initiation (OR 7.71, 95% CI 5.04−11.81, *p* = 0.000) were significantly associated with high viral load. Regarding the adverse pregnancy outcomes, high viral load was associated with a three‐fold increased risk of placental malaria (OR 3.28, 95% CI 1.54−7.11, *p* = 0.002).

**Conclusions**: Anaemia and delayed ART initiation were associated with elevated viral load, which in turn increased the risk of placental malaria. These findings highlight the need for comprehensive antenatal care, including early ART access, to improve pregnancy outcomes and reduce vertical transmission risk. Further research should explore the interplay between viral load and placental malaria.

### OAB0404 Enhancing HIV screening in pregnant women across 17 quality improvement (QI) supported LGAs: a scalable QI model for PMTCT in Jigawa State, Nigeria

S.J. Abdullahi ^1^, O. Ogundare^1^, R. Abba^1^, C. Anoke^1^, P. David Gbado^1^, R. Atteh Omolade^2^, T. Ilori^2^, M. Abdulwahab^3^, S. Magaji^1^

^1^Jhpiego, Abuja, Nigeria, ^2^National Agency for Control of AIDS, Abuja, Nigeria, ^3^Jigawa State Ministry of Health, Department of Public Health, Dutse, Nigeria

**Background**: HIV testing at initial antenatal care (ANC1) is essential for preventing mother‐to‐child transmission (PMTCT). In Jigawa state, Nigeria, where HIV prevalence is 0.3%, average HIV testing coverage among pregnant women during ANC1 in 17 QI project‐supported LGAs was just 37% from January to March 2024, posing significant challenges to PMTCT efforts. This study evaluates the impact of a Quality Improvement approach in addressing poor testing in low‐resource communities.

**Description**: A QI training facilitated by Jhpiego, a multidisciplinary team—comprising a midwife, facility manager, lab technician and Ward Development Committee chairman—was established at the LGA. Trained in the WHO Point of Care QI methodology, the team aimed to achieve 90% HIV testing coverage for first antenatal care (ANC1) attendees by December 2024. Utilizing the Plan‐Do‐Study‐Act cycle, they identified challenges—including interrupted kit supplies, poor inventory management, complex client flows and inadequate staff coordination—using Fishbone diagrams, flow charts and the 5 Whys. Interventions such as securing alternative procurement channels via the Basic Health Care Provision Fund (BHCPF) or Drug Revolving Fund (DRF), role reassignment, weekly inventory reporting and prompt requisitions at 70% stock depletion were tested in 3–4 cycles. Monthly data from PMTCT registers, analysed via the National HMIS and monitored with run charts, guided the scaling up of successful strategies across 17 LGAs, supported by on‐site coaching to ensure sustained improvements.

**Lessons learned**: HIV testing coverage for ANC1 improved from 37% in March to 82% in November 2024, identifying 16 positive cases placed on treatment. Sustained gains through monthly performance reviews, inventory management and real‐time data collection.

**Conclusions/Next steps**: QI demonstrated the potential for targeted improvements in HIV testing and case findings, where service providers identify key barriers to care and propose solutions. This model has the potential for scalable, context‐specific improvements in PMTCT programmes and can be replicated in similar settings.[Fig jia226518-fig-0009]

**Figure 1 jia226518-fig-0009:**
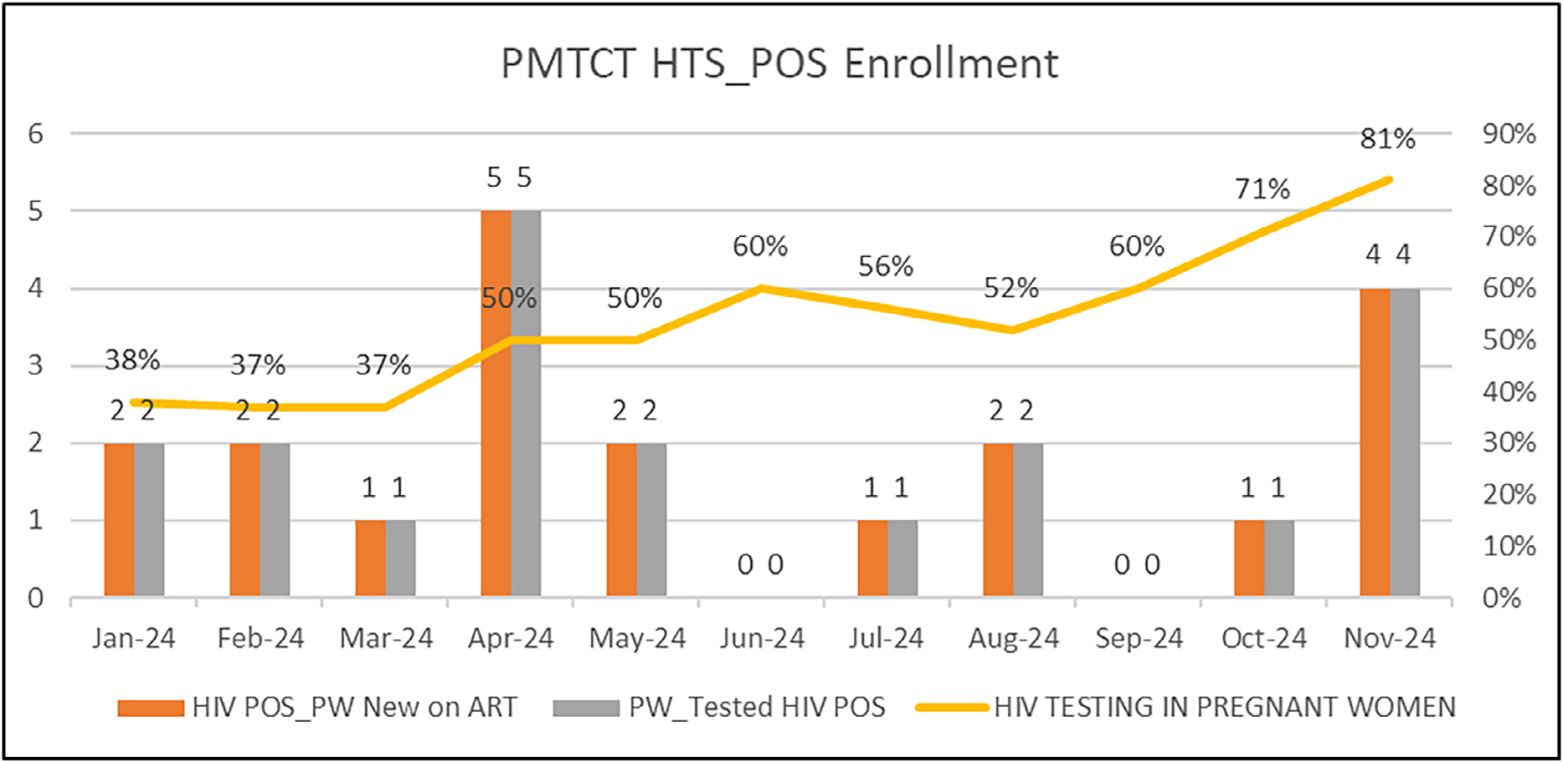
OAB0404

### OAB0405 Improving data quality as a key strategy towards achieving triple elimination of the vertical transmission of HIV, syphilis and hepatitis in Chikwawa district, Malawi

B. Sato ^1^, C. Issah^2^, A. Mdolo^3^, B. Gausi^2^, A. Mwansambo^1^, D. Telela^4^

^1^National AIDS Commission, Lilongwe, Malawi, ^2^Ministry of Health‐Chikwawa District Health Office, Chikwawa, Malawi, ^3^United Nations Children's Fund, Malawi Country Office, Lilongwe, Malawi, ^4^Clinton Health Access Initiative, Lilongwe, Malawi

**Background**: Malawi targets to eliminate vertical transmission of HIV, syphilis and viral Hepatitis B (HepB) by 2027. This requires effective decision‐making and interventions informed by consistently high‐quality data. However, non‐reporting and poor and incomplete data have been consistent challenges, including in high‐prevalence districts like Chikwawa. UNICEF supported the National AIDS Commission and the Chikwawa District Council to pilot an intervention to improve the quality of HIV, syphilis and HepB data for pregnant and breastfeeding women and their infants.

**Description**: The pilot was implemented from July to September 2024, in 10 high‐volume health facilities in Chikwawa district. Facility staff including nurses, clinicians, diagnostic assistants, data clerks and expert clients were trained in correct data recording and reporting to ensure complete high‐quality data. Complete data was defined as the record of every mother, showing tests for HIV, syphilis and HepB, with clear results, appropriate actions taken and follow‐up actions recommended. The staff were further trained to analyse and use the data to inform appropriate actions; identifying eligible mothers, initiating their tests and following up on those requiring further support. The district team supervised the facility teams fortnightly to reinforce their capacity.

**Lessons learned**: The pilot intervention improved the data completeness in antenatal registers from 93% in Q2/2024 to 99.1% in Q3/2024 across the 10 facilities. This improved decision‐making at the facility and district levels resulting in improved testing services and case management for mothers returning positive results. The intervention further improved quantification for test kits leading to uninterrupted integrated testing services. This resulted in Chikwawa's highest‐ever syphilis testing rate among pregnant women (99%) from an annual rate of 75.8% from July 2023 to June 2024. Similarly, HIV retesting in the third trimester improved from 5% at baseline to 33% after the pilot phase. The progress was, however, undermined by the deployment of new staff who were yet to be trained in data quality in the target facilities.

**Conclusions/Next steps**: Continuous mentorship and combined efforts of clinical and non‐clinical facility staff are key in ensuring data completeness and quality. Future plans include mentoring all new facility staff and scaling up the intervention to other high‐prevalence districts.

### OAB0502 A two‐by‐two factorial randomized trial of immediate and/or sepsis‐specific dose anti‐tuberculosis therapy to improve mortality for adults with sepsis and HIV in East Africa

S. Heysell ^1^, S. Mpagama^2^, M. Null^1^, B. Said^2^, E. Nuwagira^3^, T. Thomas^1^, D.R. Boulware^4^, M. Conaway^1^, C. Muzoora^3^, C.C. Moore^1^, ATLAS Trial Study Group

^1^University of Virginia, Charlottesville, United States, ^2^Kibong'oto Infectious Diseases Hospital, Sanya Juu, Tanzania, the United Republic of, ^3^Mbarara University of Science and Technology, Mbarara, Uganda, ^4^University of Minnesota, Minneapolis, United States

**Background**: The greatest global burden of sepsis is found in persons living with HIV (LWH) in Africa, in whom tuberculosis (TB) is an often overlooked but leading cause of underlying infection. We aimed to determine whether empiric immediate and/or increased dose anti‐TB therapy improved 28‐day mortality for participants LWH and admitted to hospital with sepsis in Tanzania or Uganda.

**Methods**: We conducted a phase 3, multisite, open‐label, randomized controlled clinical 2×2 factorial superiority trial of (1) immediate and (2) sepsis‐specific dose anti‐TB therapy in addition to standard of care for adults LWH admitted with sepsis at four hospitals in Tanzania or Uganda. Standard of care antibiotic regimen was ceftriaxone daily for 7 days. The primary endpoint was 28‐day mortality with a superiority margin of 13% and assuming 20% unevaluable.

**Results**: We randomized 437 participants of whom 44 were excluded, leaving 393 analysable. Of these, 224 (57%) were male. The mean (+SD) age was 43 (+13) years. The median CD4^+^ T‐cell concentration was 125 (IQR, 30−326) cells/mcl, and 202 (51%) were ultimately diagnosed with TB by urine, sputum or blood‐based tests. No difference occurred in 28‐day mortality between the four treatment groups: immediate/conventional dose (22%; 22/99), immediate/sepsis‐specific dose (28%; 28/99), diagnosis‐dependent/conventional dose (28%; 27/96) and diagnosis‐dependent/sepsis‐specific dose (23%; 23/99); chi‐squared *p* = 0.66. Among those with TB, a significant difference in 28‐day mortality occurred across treatment groups (*p* = 0.045). Compared to a 28‐day mortality of 35% (18/52) for the 52 participants with TB who received standard diagnosis‐dependent/conventional dose anti‐TB therapy, the 28‐day mortality for the 50 participants with TB who received immediate/conventional dose anti‐TB therapy was 12% (6/50) (adjusted odds ratio [aOR] 0.26, 95% CI, 0.09−0.73, *p* = 0.01), and for the 57 participants with TB who received diagnosis‐dependent/sepsis‐specific dose anti‐TB therapy, the 28‐day mortality was 19% (11/57) (aOR 0.42, 95% CI, 0.17−1.03, *p* = 0.06). No differences in adverse events occurred between treatment groups.

**Conclusions**: No overall benefit in 28‐day mortality was observed with immediate or sepsis‐specific dose anti‐TB therapy in adults with HIV‐related sepsis. However, immediate conventional anti‐TB therapy significantly reduced 28‐day mortality in those later confirmed to have TB.

### OAB0503 Same‐day antiretroviral therapy initiation in people with HIV and presumptive tuberculosis: a randomized, non‐inferiority trial in Lesotho and Malawi (SaDAPT)

F. Gerber ^1,2,3^, R. Semphere^4^, B. Lukau^5^, P. Mahlatsi^5^, G. Sanchez‐Samaniego^1,3^, M. Molatelle^6^, N.B. Marake^7^, T. Tarumbiswa^7^, A. Amstutz^1,3,8^, M. Nliwasa^4,9^, I. Ayakaka^5^, P. MacPherson^10^, T.R. Glass^2,3^, R.M. Burke^11^, N.D. Labhardt^1,3^

^1^University Hospital Basel, Department of Clinical Research, Division of Clinical Epidemiology, Basel, Switzerland, ^2^Swiss Tropical and Public Health Institute, Allschwil, Switzerland, ^3^University of Basel, Basel, Switzerland, ^4^Kamuzu University of Health Sciences, Helse Nord Tuberculosis Initiative, Blantyre, Malawi, ^5^SolidarMed Lesotho, Maseru, Lesotho, ^6^Seboche Mission Hospital Laboratory, Seboche, Lesotho, ^7^Ministry of Health Lesotho, Disease Control Department, Maseru, Lesotho, ^8^Osloo University, Oslo Center for Biostatistics and Epidemiology, Oslo, Norway, ^9^Malawi‐Liverpool‐Wellcome Trust, Clinical Research Programme, Blantyre, Malawi, ^10^University of Glasgow, School of health and Wellbeing, Glasgow, United Kingdom, ^11^London School of Hygiene and Tropical Medicine, London, United Kingdom

**Background**: Same‐day initiation of antiretroviral therapy (ART) is recommended for most people with HIV (PWH) to promote retention in care, but may increase the risk of tuberculosis‐immune reconstitution inflammatory syndrome (TB‐IRIS). It is unknown whether same‐day ART should be offered to PWH with TB symptoms or whether ART should be initiated only after TB diagnostic results are available.

**Methods**: We conducted an open‐label, 1:1, individually randomized, non‐inferiority trial comparing “ART‐first” versus “TB‐results‐first” initiation among PWH in Lesotho and Malawi, aged ≥12 years, (re)initiating ART who had at least one TB symptom (cough, fever, night sweats or weight loss) and no signs of meningitis. Participants in the “ART‐first” arm were offered same‐day ART; those in the “TB‐results‐first” arm were offered ART after TB was confirmed or refuted. The primary analysis was a non‐inferiority comparison of viral suppression (< 400 copies/ml) 26 (22−40) weeks after enrolment (non‐inferiority margin 10%). Secondary outcomes included: retention in care; mortality; non‐fatal serious adverse events (SAEs); ART initiation within 7 days; and TB‐IRIS. NCT05452616.

**Results**: From 19/10/2022 to 21/02/2024, 581 participants were randomized and included in intention‐to‐treat analysis (60% male; median age 37 years; median CD4 201 cells/mm^3^); 573 included in per‐protocol analysis. 296/297 (99%) and 60/284 (21%) in the “ART‐first” and “TB‐results‐first” arms started ART on enrolment day and 59/297 (20%) and 55/284 (19%) were diagnosed with TB by 30 weeks. Viral suppression rates at 26 weeks were 209/296 (71%) in the “ART‐first” arm and 196/277 (71%) in the “TB‐results‐first” arm in the per‐protocol population (absolute risk difference −0.8%; 95% CI: −8.4% to 6.8%) with similar results in the intention‐to‐treat population, confirming non‐inferiority of “ART‐first.” Retention in care was 81% in both arms; ART initiation within 7 days was higher in “ART‐first” (99% vs. 86%). Nine and six deaths, 11 and 10 non‐fatal SAEs, and six and five TB‐IRIS events occurred in “ART‐first” and in “TB‐results‐first” arms, respectively.

**Conclusions**: Outcomes of same‐day initiation, and ART initiation after TB investigations were comparable and implementation should be guided by programme and client preference.[Table jia226518-tbl-0003]

**Table 1 jia226518-tbl-0003:** OAB0503

	ART‐first arm	TB‐results‐first arm	Adjusted % risk difference (95% CI)
1° endpoint VL < 400 copies/ml after 26 (22−40) weeks (per‐protocol)	209/296 (71%)	196/277 (71%)	−0.8 (−8.4 to 6.8)
1° endpoint VL < 400 copies/ml after 26 (22−40) weeks (intention‐to‐treat)	209/297 (70%)	201/284 (71%)	−1.0 (−8.5 to 6.5)
2° endpoint retention in care after 22−30 weeks	240/297 (81%)	229/284 (81%)	0.1 (−6.3 to 6.6)
2° endpoint ART initiation within 7 days	296/297 (99%)	243/284 (86%)	14.2 (10.0−18.3)

### OAB0504 Post‐tuberculosis treatment mortality in children and youth initiating ART in the International Epidemiology Databases to Evaluate AIDS (IeDEA) Network

H. Root ^1^, J. Brust^1^, V. Rouzier^2^, L. Enane^3,4^, K. Anderson^5^, O. Marcy^6^, Q.T. Du^7^, L. Diero^8^, I. Ayakaka^9^, J. Dame^10^, Q.D. Nguyen^11^, D.M. Machado^12^, F. Odhiambo^13^, J. Euvrard^14^, C. Yonaba^15^, A. Kinikar^16^, J.A. Pinto^17^, W. Muyindike^18^, M.T. Luque^19^, M. Ballif^20^, L. Bagnan^21^, D.K. Wati^22^, Q. Shi^23^, M. Yotebieng^24^, and International Epidemiology Databases to Evaluate AIDS (IeDEA), United States

^1^Albert Einstein College of Medicine, Department of Medicine, Division of Infectious Diseases, Bronx, United States, ^2^GHESKIO Center, Port‐au‐Prince, Haiti, ^3^CCASAnet, Nashville, United States, ^4^Indiana University School of Medicine, Indianapolis, United States, ^5^University of Cape Town, Cape Town, South Africa, ^6^University of Bordeaux, Talence, France, ^7^Children's Hospital 1, Ho Chi Minh City, Viet Nam, ^8^Moi University, Medicine, Kesses, Eldoret town, Kenya, ^9^Solidarmed Lesotho, Maseru, Lesotho, ^10^University of Ghana Medical School and Korle bu Teaching Hospital, Child Health, Accra, Ghana, ^11^Children's Hospital 2, Infectious Diseases, Ho Chi Minh City, Viet Nam, ^12^Universidade Federal de São Paulo, São Paulo, Brazil, ^13^The Centre for Microbiology Research, The Research Care and Training Program, Kenya Medical Research Institute, Nairobi, Kenya, ^14^Khayelitsha ART Programme, Western Cape Government Health and Wellness, Cape Town, South Africa, ^15^Yalgado Ouégraogo University Hospital, Ouagadougou, Burkina Faso, ^16^BJ Medical College and Sassoon General Hospital, Pune, India, ^17^Federal University of Minas Gerais, Belo Horizonte, Brazil, ^18^Mbarara University of Science and Technology, Mbarara, Uganda, ^19^Instituto Hondureño de Seguridad Social, Tegucigalpa, Honduras, ^20^Institute of Social and Preventive Medicine, University of Bern, Bern, Switzerland, ^21^National University Hospital, Cotonou, Benin, ^22^Prof. dr. I.G.N.G. Ngoerah General Hospital, Udayana University, Pediatrics, Bali, Indonesia, ^23^School of Health Sciences & Practice, New York Medical College, Valhalla, United States, ^24^Albert Einstein College of Medicine, Medicine, Bronx, United States

**Background**: Tuberculosis (TB) is a major cause of death among children and youth living with HIV (CYLWH). In adults, even after cure, those with former TB are three to seven times more likely to die following TB treatment. However, the risk of mortality after TB treatment in CYLWH is unknown. We sought to compare the post‐treatment mortality in CYLWH who were treated for TB disease with those without TB.

**Methods**: We analysed data from CYLWH (0−24 years) who initiated ART at < 25 years old from 2004 to 2021 at IeDEA‐participating sites in six regions and who had at least 9 months of follow‐up from time of ART initiation. Participants who had initiated treatment for active TB disease 3 months prior to or after ART initiation were the TB disease group; those without documentation of any TB treatment through 9 months after ART initiation were the TB‐negative/control group. Cumulative incidence of death (CID) from 9 months after ART initiation onwards was calculated with lost‐to‐follow‐up and transferred‐out treated as competing risks. Fine‐Gray competing proportional hazard modelling was used to compare the hazard of death, adjusting for age, sex, CD4, WHO Stage and weight‐for‐age Z‐score.

**Results**: Among 75,259 CYLWH included, 3622 had been treated for TB disease and 62,495 had no TB history. The CID rose constantly from 2 to 15 years post‐ART initiation from 3.8% (95% CI: 3.2%−4.5%) to 20.4% (95% CI: 18.3%−22.5%) in the TB disease group versus 1.6% (95% CI: 1.5%−1.7%) to 10.9% (95% CI: 10.2%−11.5%) in the control group, respectively (Figure 1). In bivariate analysis, the sub‐hazard risk of death among those in the TB disease group was 2.2 (95% CI: 2.0−2.4) times that of the controls. This difference remained unchanged after adjustment (aHR 2.1 [1.9−2.4]).

**Figure 1:**
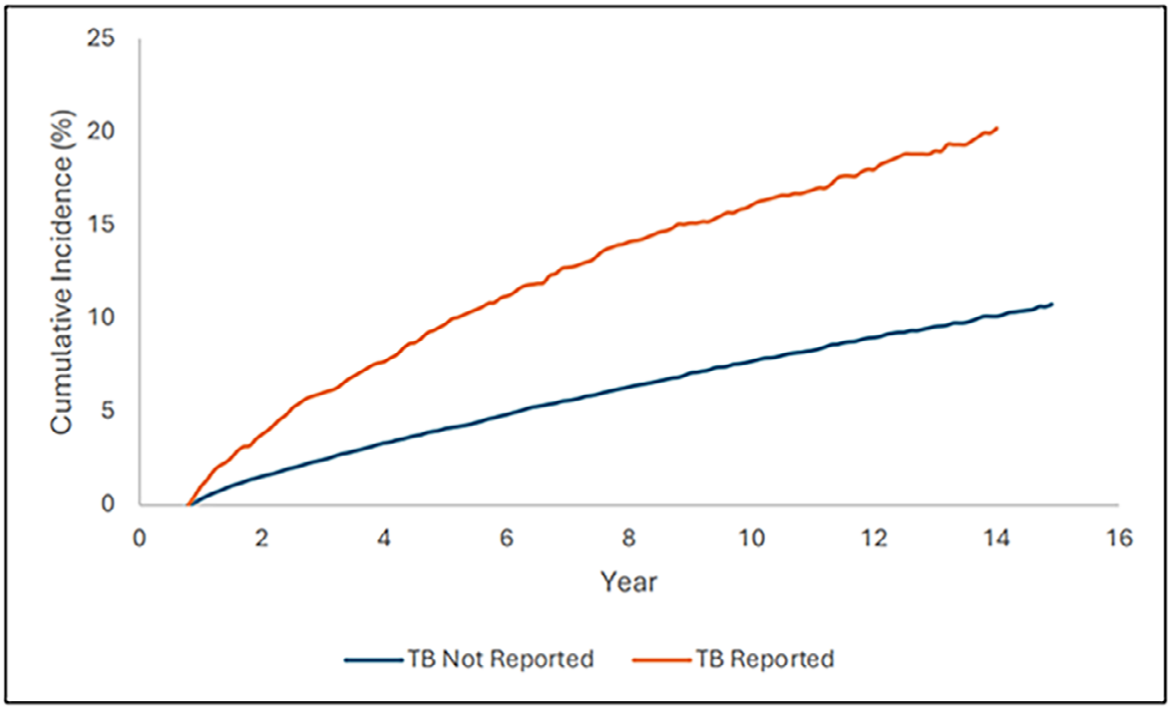
**OAB0504: Cumulative incidence of death from 9 months after date of ART initiation among those treated for TB disease (orange) and those without TB (blue). Log‐Rank *p* < 0.00071**.

**Conclusions**: Among CYLWH on ART, receiving TB treatment near ART initiation (vs. those without TB) was associated with increased mortality even 15 years after TB treatment.

### OAB0505 Predictors of recurrent tuberculosis among people living with HIV under routine ART care: an observational cohort study, Andhra Pradesh, India, 2024

M. Thogarucheeti^1^, S. Attada^1^, K. Prasad^1^, N. Chava^2^, G. Ramesam^2^, P. Kumar^2^, A. Rao^2^, J. Kurada^2^, V. Yeldandi ^2^, P. Turaka^2^, R.P. Poluru^2^, C. Sriramulu^1^, P. Nadol^3^, R. Allam^3^, C. Das^4^

^1^Andhra Pradesh State AIDS Control Society, Vijayawada, India, ^2^Society for Health Allied Research & Education India, Hyderabad, India, ^3^U.S Centres for Disease Control and Prevention, DGHT, Delhi, India, ^4^National AIDS Control Organization, Delhi, India

**Background**: Recurrent tuberculosis (TB) is associated with poor treatment outcomes compared to primary TB infection and has implications for TB control. India's national guidelines recommend post anti‐TB treatment (ATT) follow‐up for 2 years from the last date of ATT, spaced at 6‐month intervals. The TB recurrence rate among non‐people living with HIV (PLHIV) under programme settings is 2.7%. However, limited data is available on recurrent TB predictors among PLHIV in India.

**Methods**: We analysed secondary programme data from 14 antiretroviral therapy (ART) clinics located in three high‐burden districts (East Godavari, Krishna and Guntur) of Andhra Pradesh. PLHIV were screened for TB (four symptom screening) during every visit for ART pill pick‐up. We included adult PLHIV aged > 15 years who initiated and completed ATT successfully during January 2017–December 2021 and followed‐up for 2 years from ATT completion and calculated recurrent TB incidence rate (IR) per 100 person‐years (PY). We used adjusted Cox proportional hazard models and calculated hazard ratios (HR) to determine predictors of recurrent TB.

**Results**: The 3357 PLHIV meeting the inclusion criteria accounted for 6372 PY of follow‐up. A total of 270 PLHIV were diagnosed with recurrent TB during follow‐up (IR: 4.24 PY, 95% CI: 3.75−4.77). Select variables, such as microbiologically diagnosed pulmonary TB (HR: 1.79, 95% CI: 1.12−2.88), CD4 count ≤200 cells/mm^3^ at recurrent TB diagnosis (HR: 1.38, 95% CI: 1.03−1.87), HIV treatment interruption during follow‐up (HR: 2.38, 95% CI: 1.79−3.16), no exposure to dolutegravir‐based regimen during follow‐up (HR: 2.38, 95% CI: 1.79−3.16), HIV viral load ≥1000 copies/ml at recurrent TB diagnosis (HR: 3.25, 95% CI: 2.14−4.94), no exposure to secondary prophylaxis of isoniazid (HR: 1.32, 95% CI: 1.04−1.68) and > 9‐month delay of isoniazid preventive therapy initiation after completion of ATT (HR: 1.66, 95% CI: 1.21−2.28), were associated with significantly higher risk of recurrent TB.

**Conclusions**: The higher rate of incidence for post‐ATT recurrent TB could be due to increased opportunity for TB screening at ART clinics compared to community‐based or non‐HIV settings. ART clinic staff may focus on modifiable risk factors, such as preventing HIV treatment interruption, enabling viral load suppression and ensuring early initiation of secondary TB preventive treatment to reduce the rates of recurrent TB among PLHIV.

### OAC0102 Averting new HIV acquisitions through micro‐targeting of integrated community interventions with precision. A case of Nyarutojo Parish Kambuga TC, Kanungu district Southwestern Uganda

A. Mbabazi ^1^, G. Tugume^1^, M. Asiimwe^1^, A. Twiine^1^, L. Linda^1^, C. Tiri^1^

^1^Joint Clinical Research Centre, Programs, Kabale, Uganda

**Background**: Uganda still experiences challenges in the identification of new HIV acquisitions despite commendable progress towards attainment of the UNAIDS global 95‐95‐95 targets. To end AIDS by 2030, more efficient and targeted case finding approaches need to be employed to reach the remaining pockets of the population with unknown HIV status. USAID LPHS Kigezi & Lango Activity piloted the use of precision targeting to guide community interventions focused to reaching the most at‐risk populations with HIV services geared at averting new acquisitions.

**Description**: Patient‐level data on viral load non‐suppression, interruption in treatment and HIV positivity was extracted from Uganda EMR and utilized to make geospatial maps that informed the identification of geographical locations with the highest HIV transmission risk. Nyarutojo parish in Kanungu district was identified as one of the “hottest hotspots” parishes. Dialogue meetings were conducted with health workers at health facilities serving this parish, key population peers and key community gate keepers. Profiling of these hotspots revealed; many bars with high numbers of “trafficked” adolescent girls and young women working as bar attendants but also engaged in commercial sex work, mobile men with disposable income from coffee trade and high prevalence of STIs. Client HIV charts were reviewed to validate the missing services for clients with non‐suppressed viral load and those who had dropped out of care. Integrated outreaches to provide HIV prevention, care and treatment services have been conducted and linkages established with livelihood programmes.

**Lessons learned**: Four hundred and twenty‐seven individuals were reached with HIV prevention services. 92.3% (394) were offered risk‐based HTS with eight HIV positives and linked into care. Two hundred and ninety‐eight (85%) of the 386 who tested HIV negative were screened for PrEP and 103 (67%) of the 152 PrEP initiated.

- Community livelihood skilling project that attracts AGYW can provide a platform for accessing HIV combination prevention packages and testing services.
- Missed opportunity for HIV testing for STI clients majorly presenting at HC IIs without HIV testing services.
- Precision targeting revealed new hot spots beyond the traditional ones.

**Conclusions/Next steps**: Precision targeting optimizes access to a combined HIV prevention package for the most at‐risk populations.

### OAC0103 High acceptability and accuracy of combined HIV self‐testing and genital secretion self‐collection for oncogenic HPV molecular detection among female sex workers in Democratic Republic of the Congo

S. Tonen‐Wolyec^1,2^, J. Muwonga Tukisadila^3,4^, L. Belec ^5^

^1^University of Bunia, Infectious Diseases, Bunia, Congo, the Democratic Republic of the, ^2^University of Kisangani, Infectious diseases, Kisangani, Congo, the Democratic Republic of the, ^3^University of Kinshasa, Clinical biology, Kinshasa, Congo, the Democratic Republic of the, ^4^Ecole Doctorale Régionale d'Infectiologie Tropicale, Franceville, Gabon, ^5^Laboratoire de Virologie, Hôpital Européen Georges Pompidou and Faculty of Medicine Paris Descartes, University of Paris Cité, Paris, France

**Background**: HIV infection is one of the major cofactors of oncogenic high‐risk human papillomavirus (HR‐HPV) which is associated with cervical cancer. Female sex workers (FSWs) in sub‐Saharan Africa (SSA) are at very high risk of both HPV‐ and HIV‐co‐infections. Synergy of innovative methods such as genital self‐sampling (GSS) and HIV self‐testing (HIVST) constitute promising way of controlling both viral infections. This study aimed to assess the effect of peer education‐based rapid training (PERT) on the acceptability and usability of GSS and HIVST in adult FSWs living in Kisangani and Bunia, Democratic Republic of the Congo (DRC).

**Methods**: The study is a community‐based, individually randomized controlled trial (1:1). In control group, FSWs were asked to execute in private setting at home, at their own pace and in an autonomous way the GSS with vaginal veil (V‐Veil Up UP2™, V‐Veil‐Up Production SRL, Romania) and HIVST using capillary‐based HIV self‐test (Biosynex, Strasbourg, France). In intervention group, FSWs received approximately 1 hour of PERT by two trained peer educators. PERT was systematically carried out with the help of a vade‐mecum including key education messages concerning HIV infection, HPV and the simplified instructions for GSS and HIVST.

**Results**: A total of 613 FSWs were prospectively included, with 309 in control group and 304 in intervention group. Rates of acceptability (GSS: 98% vs. 76%; OR: 4.3 [95% CI: 2.0−6.6] and HIVST: 96% vs. 84%; OR: 2.3 [95% CI: 1.1−4.8]) and usability (GSS: 99% vs. 81%; OR: 3.8 [95% CI: 1.5−5.6] and HIVST: 100% vs. 89%; OR: 3.2 [95% CI: 1.8−7.3]) of GSS and HIVST were higher in FSWs offered PERT than in those not offered. The educational level and age of FSWs were not associated with acceptability and usability in multivariate analyses. Moreover, the average of satisfaction level on Likert scale (1−5) after performing GSS and HIVST was higher among FSWs receiving PERT than those not receiving it (GSS: 4.2 vs. 3.7; *p* = 0.003 and HIVST: 4.4 vs. 4.1; *p* = 0.012).

**Conclusions**: This study highlights the need for educational intervention adapted to vulnerable poorly educated populations living in SSA to support innovative interventions such as combined HIVST and GSS.

### OAC0104 Upscaling HIV testing and clinical service uptake among male sexual partners of adolescent girls and young women in rural Eswatini communities through invitation cards for adolescent boys and young men

T. Dlamini ^1^, M. Maseko^2^, M. Dlamini^2^, H. Nxumalo^2^, H. Mkhatshwa^1^

^1^World Vision, Programs, Mbabane, Eswatini, ^2^World Vision, Strategic Information, Mbabane, Eswatini

**Background**: World Vision Eswatini (WVE) is implementing a project that is targeting orphaned and vulnerable children as well as adolescent girls and young women, with the goal of reducing new HIV infections in Eswatini. HIV testing is essential for the awareness of HIV status and an important component of HIV epidemic control. According to a recent HIV incidence survey (SHIMS 3), 38.9% of males between the ages of 15−24 reported to have received an HIV test in the last 12 months before the survey, while females were at 61.4%. The Multi‐Indicator cluster survey (MICS) 2021 findings show that 56.4% of males aged 15−24 years who are sexually active tested for HIV and know the results, while their female counterparts reported 80.8%. Observed low clinical service uptake among men necessitates innovations in demand creation.

**Description**: WVE piloted an innovation themed “Lisango – The Men Cave” which uses an exclusively designed invitation card to invite males between the ages of 15−45 years, who are sexual partners of enrolled AGYW. AGYW shared the card with their sexual partners and other male counterparts. Community‐based mentors also distributed the invitations on behalf of AGYW who expressed discomfort in personally extending the invitation to their sexual partners. Males confirmed their attendance on the cards. Sessions end with an officer issuing health referrals prioritizing combination HIV prevention services including HIV testing services (HTS), and other clinical services. In 2024, WVE reached 564 males who accessed health services including HTS, 44 were initiated on PrEP, 100 were screened for STI, while 441 accessed condoms.

**Lessons learned**: Empowering AGYW to advocate for themselves with their sexual partners is important in the efforts to end HIV. AGYW‐led mobilization strategies yielded improvements in HIV testing and PrEP and condom uptake among sexual partners, and can be prioritized for scale up.

**Conclusions/Next steps**: Upscaling HIV prevention service uptake requires innovative demand creation strategies and using AGYW in mobilizing their sexual partners can increase case finding in communities.

### OAC0105 Adoption of HIV preexposure prophylaxis (PrEP) among female sex workers (FSWs) in Côte d'Ivoire: complex trajectories and early adopters

J. Larmarange ^1,2^, M. Plazy^3^, M. Nouaman^4,5^, E. Kissi^4^, R.‐M. Dedocoton^4^, P.A. Coffie^4,5^, S. Eholié^4,5^, V. Becquet^1,2^, ANRS 12381 PRINCESSE Study Group

^1^Institut de Recherche pour le Développement (IRD), Ceped (Centre Population et Développement, UPC, IRD, Inserm, USPN), Paris, France, ^2^Institut National des Etudes Démographiques (Ined), DemoSud, Aubervilliers, France, ^3^Université de Bordeaux, GHiGS, Bordeaux Population Health, Bordeaux, France, ^4^Programme PAC‐CI, Abidjan, Côte d'Ivoire, ^5^Université Félix Houphouët Boigny, Abidjan, Côte d'Ivoire[Fig jia226518-fig-0011]

**Background**: The ANRS 12381 PRINCESSE project (11/2019−06/2023) enrolled 489 FSWs and implemented community‐based sexual and reproductive healthcare, including PrEP, delivered through mobile clinics at prostitution sites in the San Pedro region. This presentation aims to describe PrEP trajectories and the factors associated with its use, focusing on “early adopters.”

**Methods**: This analysis included 400 FSWs eligible for PrEP (HIV negative, HBsAg negative) enrolled up to December 2022 (> 6 months of follow‐up). Clinical records were analysed to describe PrEP cascade, follow‐up and PrEP trajectories. A multivariable logistic regression identified factors associated with PrEP early adopters.

**Results**: Although 98% of eligible FSWs were interested in PrEP, only 62% initiated PrEP, and 39% renewed it at least once.

Follow‐up was very short (< 6 months) for 48%, short (6−12 months) for 15%, seasonal (> 12 months with a gap of 6 months between two visits) for 31% and regular (> 12 months with no gap) for 6%.

PrEP initiation was high among FSWs with regular (96%), seasonal (83%) or short (80%) follow‐up, versus 39% for those with very short follow‐up. Among those who initiated, only 88% (regular), 70% (seasonal), 74% (short) and 35% (very short) renewed PrEP.

Among 148 FSWs with regular/seasonal follow‐up, four PrEP trajectories were identified (cf. figure showing individual trajectories): 15% “never initiated” PrEP, 39% “initiated and were later not interested anymore,” 18% “re‐initiated after non‐interest” and 28% “initiated and never expressed non‐interest,” the last two groups being considered as “early adopters.” Factors associated with early PrEP adoption included the usual price of intercourse with clients < 1500 FCFA (aOR = 2.4 [1.2−4.8]) and working in brothels (aOR = 3.2 [1.6−6.6]).

**Conclusions**: In PRINCESSE, loss‐to‐follow‐up and seasonality of risks limited PrEP adoption. FSWs who were more precarious and less mobile became early adopters of this new prevention tool and could serve as potential ambassadors for promoting its uptake.

**Figure 1 jia226518-fig-0011:**
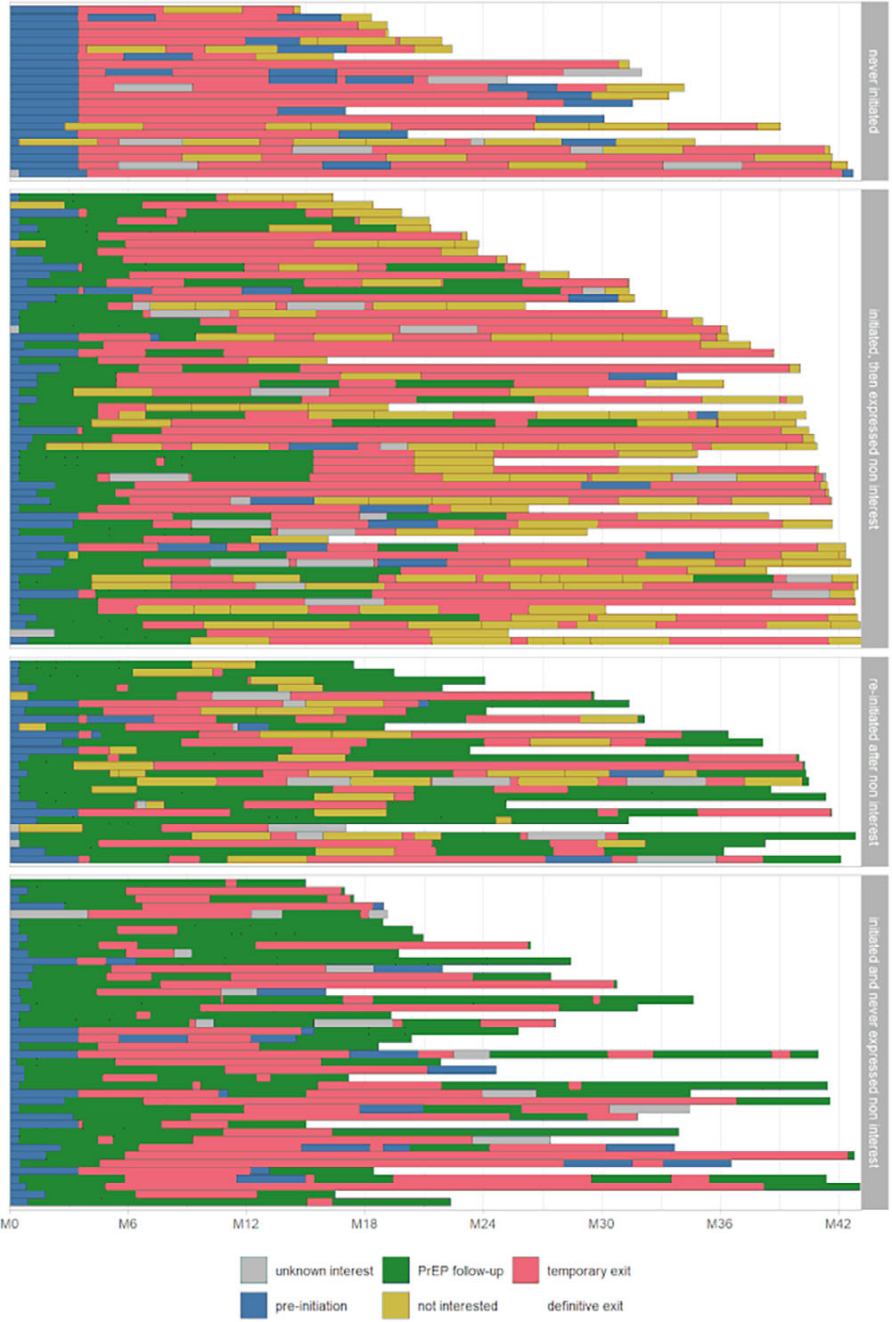
OAC0105

### OAC0202 USAID‐supported PrEP introduction and scale up, 2016−2024

A. Kimmel^1^, A. Dam ^2^, M. Cobourne^1^, J. Rodrigues^3^, T. Mukherjee^3^

^1^Unaffiliated, Washington, United States, ^2^Johns Hopkins Bloomberg School of Public Health, Baltimore, United States, ^3^Unaffiliated, New York, United States

**Background**: Pre‐exposure prophylaxis (PrEP) is a safe and highly efficacious form of biomedical HIV prevention; however, only an estimated 3.5 million people in 2023 were using oral PrEP, short of the UNAIDS global target of 21.2 million users by 2025. The U.S. Agency for International Development (USAID), through PEPFAR and in partnership with host countries’ ministries of health, provided support for PrEP implementation beginning in fiscal year 2017 (FY17) until ordered to stop work by the Trump administration in January 2025.

**Methods**: This analysis used the PEPFAR Monitoring, Evaluation, and Reporting data to describe USAID‐supported PrEP initiations globally from 1 October 2016 (FY17 quarter 1) through 30 September 2024 (FY24 quarter 4), by prioritized population (adolescent girls and young women age 15−24 [AGYW], pregnant and breastfeeding women [PBFW], key populations [KPs], female sex workers [FSWs], men who have sex with men [MSM], transgender people [TG], people who inject drugs [PWID], and persons in prisons and other closed settings) and geographies.

**Results**: USAID expanded support for PrEP initiation from 14 countries in FY17 to 43 countries in FY24. In total, USAID supported 3,776,559 people to initiate PrEP. Ninety‐two percent of these initiations occurred between FY21 and FY24, following a PEPFAR‐wide initiative to initiate 1 million people on PrEP in FY21. Distribution by population was: 2,187,746 (58%) KP (FSW: 1,199,332; MSM: 773,304; people in prisons and other closed settings: 33,648; PWID: 109,150; TG: 72,492); 2,724,770 (72%) AGYW; and 57,628 (1.5%) PBFW (FY24 data only). In FY24, USAID supported the introduction of long‐acting injectable cabotegravir (CAB‐LA), with 2490 CAB‐LA initiations. Distribution by geography was: 47,304 (1.3%) the Americas; 149,165 (3.9%) Southeast Asia; and 3,580,090 (95%) sub‐Saharan Africa.

**Conclusions**: USAID contributed an estimated 18% towards the UNAIDS 2025 global target. The U.S. Secretary of State has stated that USAID will not operate after 1 September 2025. Without USAID‐supported PrEP, global populations will be more vulnerable to infection, erasing decades of work towards HIV epidemic control and creating preventable HIV morbidity and mortality.

### OAC0203 Cost‐effectiveness of cabotegravir long‐acting for HIV pre‐exposure prophylaxis (PrEP): a systematic review of modelling studies

F. Effiong ^1^, E. Ekpor^2^, R. Dine^3^, D. Olawuyi^4^, D. Adewole^5^

^1^University of Calabar Teaching Hospital (UCTH), Medical Laboratory Services, Calabar, Nigeria, ^2^School of Nursing and Midwifery, University of Ghana, Legon, Ghana, ^3^Rinda‐Ubuzinma, Kigali, Rwanda, ^4^University of Ibadan, Department of Medicine and Surgery, Faculty of Clinical Science, College of Medicine, Ibadan, Nigeria, ^5^University of Ibadan, Department of Health Policy and Management, College of Medicine, Ibadan, Nigeria

**Background**: Cabotegravir long‐acting (CAB‐LA) is a promising HIV prevention strategy; however, its cost‐effectiveness compared to oral pre‐exposure prophylaxis (PrEP) varies across settings. This systematic review examines the economic viability of CAB‐LA interventions using modelling studies in diverse populations.

**Methods**: We searched literature databases for modelling studies on the cost‐effectiveness of CAB‐LA in various settings. The search was executed in PubMed, Web of Science, Scopus and the Cochrane Library. The search was conducted in January, 2025, and was limited to English studies; but there was no limitation on year of publication. Quality assessment was based on the 2022 CHEERS checklist for economic evaluation studies. A narrative synthesis was conducted to summarize the findings. Key outcomes to be extracted included study characteristics and design, incremental cost‐effectiveness ratios (ICERs), adherence rates and willingness‐to‐pay (WTP) thresholds.

**Results**: The search retrieved 19 results, but only six modelling studies meeting predefined inclusion criteria were included. These studies evaluated CAB‐LA among various populations, including heterosexual men, women at high risk of HIV, men who have sex with men (MSM), transgender women (TGW) and large simulated cohorts. The studies employed static epidemiological models, deterministic compartmental models and Markov cohort models to evaluate CAB‐LA alongside oral PrEP. Quality assessment results show that studies were of moderate and high quality. CAB‐LA demonstrated potential cost‐effectiveness under specific conditions. In sub‐Saharan Africa, CAB‐LA achieved ICERs below $1000 per disability‐adjusted life year (DALY) averted at adherence rates exceeding 75%. In high‐income settings, ICERs for CAB‐LA remained below $98,000 per quality‐adjusted life year (QALY) when drug costs were reduced to $4100/year. Low‐income settings required annual costs below $16 for cost‐effectiveness. Epidemiological benefits included a 30%−40% reduction in HIV incidence with optimal adherence. Drug pricing, adherence and quarterly monitoring were identified as key determinants of cost‐effectiveness. Comparisons with oral PrEP indicated that CAB‐LA could be more cost‐effective in populations with low adherence to oral regimens.

**Conclusions**: CAB‐LA is a cost‐effective HIV prevention intervention under specific economic and adherence scenarios. Reducing drug costs and enhancing adherence strategies are critical to optimizing its economic and epidemiological impact.

### OAC0204 Equivalent performance of HIV oral fluid self‐testing and rapid testing compared to nucleic acid amplification test in screening adolescents for long‐acting injectable cabotegravir in Brazil

B. Oliveira Leite ^1^, L. Magno^2^, F. Soares^1^, D. Zeballos^1^, L. Dezanet^3^, O. Ferreira^4^, M. Westin^5^, D. Greco^5^, A. Grangeiro^6^, I. Dourado^1^, The PrEP15‐19 Choices study group

^1^Federal University of Bahia, Institute of Collective Health, Salvador, Brazil, ^2^University of State of Bahia, Life Sciences Department, Salvador, Brazil, ^3^Oswaldo Cruz Foundation, René Rachou Institute, Belo Horizonte, Brazil, ^4^Federal University of Rio de Janeiro, Molecular Virology Laboratory, Rio de Janeiro, Brazil, ^5^Federal University of Minas Gerais, Belo Horizonte, Brazil, ^6^University of São Paulo, Medical School, São Paulo, Brazil

**Background**: Limited data are available on the risk of developing integrase inhibitor resistance among individuals initiating long‐acting injectable cabotegravir (CAB‐LA) during acute HIV infection. While nucleic acid amplification test (NAAT) is considered the gold standard in this scenario, its routine implementation during CAB‐LA initiation may be impractical due to its high cost and longer processing time. This study aimed to assess the performance of the HIV oral fluid self‐test (ST) and the rapid test (RT) in comparison to NAAT for guiding CAB‐LA initiation among adolescents in Brazil.

**Methods**: The PrEP15‐19 Choices study is a real‐world implementation project evaluating the use of oral and CAB‐LA PrEP among cisgender men who have sex with men, transgender and non‐binary individuals aged 15−19 years in three cities in Brazil. Data were collected between April 2024 and January 2025. Adolescents enrolled in the CAB‐LA arm underwent the HIV Detect® Oral Fluid ST. Peripheral venous blood samples were also collected to perform the fourth‐generation RT (Determine™ HIV Early Detect) and the NAAT (Xpert® HIV‐1 Qual), which served as the reference standard. The ST and RT were evaluated as index tests against NAAT, with sensitivity, specificity, positive predictive value (PPV), negative predictive value (NPV) and kappa agreement coefficients calculated.

**Results**: Of the 479 adolescents enrolled in the study, 180 (37.6%) opted to initiate CAB‐LA and were screened for HIV infection. Two participants were excluded: one due to a missing ST result and another due to an inconclusive ST result recorded by the participant. Among the remaining 178 participants (98.9%) with completed tests, five (2.8%; 95%confidence interval [CI]: 1.0–6.8) tested positive across all methods, while 173 (97.2%; 95% CI: 93.2–99.0) tested negative across all methods. Both the oral fluid ST and the RT demonstrated a sensitivity, specificity, PPV and NPV exceeding 99.9% (*p* = 0.006; kappa = 1).

**Conclusions**: Despite limitations in the sample size, the findings suggest that both ST and RT produced results that were consistent with the NAAT, indicating that they might be viable for diagnosing adolescents, including those from diverse sexual and gender identities. This could have implications for improving accessibility to testing and treatment for this group.

### OAC0205 Evaluating HIV testing strategies to reduce drug resistance during CAB‐LA rollout in Thailand. A modelling study

D. van de Vijver ^1^, J. van Kampen^1^, A. Wongsa^2^, R. Janamnuaysook^2^, T. Mesplede^1^, L. Matrajt^3^, M.R. Jordan^4,5^, N. Phanuphak^2^, D. Dimitrov^3^

^1^Erasmus Medical Centre, Rotterdam, Netherlands, the, ^2^Innovative HIV Research Institute (IHRI), Bangkok, Thailand, ^3^Fred Hutchinson Cancer Center, Seattle, United States, ^4^Tufts Medical Center, Boston, United States, ^5^Tufts University School of Medicine, Boston, United States

**Background**: Long‐acting cabotegravir (CAB‐LA), an integrase strand transfer inhibitor (INSTI), is effective as pre‐exposure prophylaxis (PrEP). CAB‐LA delays HIV detection, allowing its continued use after HIV acquisition and increasing the risk of INSTI resistance mutations. Resistance to CAB‐LA leads to cross‐resistance with dolutegravir, a key drug in WHO‐recommended HIV treatment regimens. CDC recommends sensitive antigen and RNA tests for timely HIV detection in CAB‐LA users, but these tests are expensive and not widely available. We study the impact of testing strategies on HIV transmission and drug resistance after CAB‐LA implementation.

**Methods**: An HIV transmission model calibrated to the Thai HIV epidemic among men who have sex with men (MSM) assumed an increase in PrEP users from the current 50,000−70,000 to 100,000 by 2034. CAB‐LA use ranged from 0% to 100% of PrEP users by 2034. Literature indicated CAB‐LA during HIV carries a 50%–100% risk of resistance development, and INSTI resistance reduces viral transmissibility by 30%–40%. The study compares CAB‐LA implementation to the current situation (0%−5% resistance during viral failure with a dolutegravir containing regimen, no transmission of INSTI resistance). The model assumed CAB‐LA use for 1 year with HIV testing every 2 months. Three HIV testing scenarios were assessed: (1) the most sensitive scenario in which all new infections are identified, and two less sensitive testing scenarios; (2) acute infections undetected; and (3) 50% of infections undiagnosed during the first year.

**Results**: Without CAB‐LA, 40,000 new infections occur between 2024 and 2034, and 524 individuals are expected to have INSTI drug resistance during viral failure with a dolutegravir containing regimen. CAB‐LA can avert 1600−6200 infections (20%–100% uptake) but increases INSTI drug resistance cases by 28–94 individuals during viral failure (increase 5−18%) and by 4–15 individuals through transmitted INSTI resistance. More sensitive HIV testing reduced resistance during viral failure by 9–36 (reduction 32−38%) individuals and transmitted resistance by 2–6 individuals (40−50%).

**Conclusions**: CAB‐LA introduction in Thailand can reduce new HIV infections but may simultaneously increase drug resistance to INSTIs. More sensitive HIV testing will prevent INSTI resistance. However, given the low INSTI resistance incidence, more sensitive testing is not critical for CAB‐LA rollout.

### OAC0302 Evaluating the impact of HIV self‐test distribution on HIV diagnosis across health facilities in Kenya

A. Bershteyn ^1^, K. Bhamidipati^1^, E. Kirui^2^, A. de Nooy^3^, H.‐Y. Kim^1^, B.E. Nichols^3,4,5^, A. Monroe‐Wise^6^, C.C. Johnson^6^, C. Kisia^7^, B. Mambo^7^, J.O. Magare^8^

^1^NYU Langone Health, Department of Population Health, New York, United States, ^2^Ministry of Health, National Public Health Laboratory, Nairobi, Kenya, ^3^Amsterdam University Medical Center, Department of Global Health, Amsterdam, Netherlands, the, ^4^University of the Witwatersrand, Wits Diagnostic Innovation Hub, Faculty of Health Sciences, Johannesburg, South Africa, ^5^Boston university School of Public Health, Department of Global Health, Boston, United States, ^6^World Health Organization, Global HIV, Hepatitis and STI Programmes, Geneva, Switzerland, ^7^World Health Organization, Kenya Country Office, Nairobi, Kenya, ^8^Ministry of Health, National AIDS and STI Control Program, Nairobi, Kenya

**Background**: HIV self‐testing (HIVST) can reduce barriers to HIV diagnosis, especially in resource‐limited settings with limited staffing and high HIV burden. While the impact of HIVST rollout on HIV diagnosis rates has been evaluated in randomized controlled trials, few studies have assessed its effects in routine practice at a national scale. This study evaluates the impact of HIVST on HIV diagnosis across Kenyan health facilities using routine programme data.

**Methods**: We used multivariable linear regression models to evaluate the association between the number of HIVST kits distributed and the number of HIV‐positive diagnoses monthly at the health‐facility level. As a sensitivity analysis, we adjusted the model for either the monthly facility‐based HIV testing volume or the number of facility‐based HIV‐negative tests, as two separate models. Both models were further adjusted for county‐level HIV prevalence. In the subgroup analyses, we modelled the effect of total HIVST kits distributed on HIV‐positive diagnoses by age and sex subgroups. These models were also adjusted for either facility‐based HIV testing volume or HIV‐negative tests within the respective subgroup, as well as county‐level HIV prevalence. Analyses were restricted to health facilities with available data for both HIVST and facility‐based tests.

**Results**: From 2019 to 2023 in Kenya, across 5883 health facilities, 1,399,865 HIVST kits were distributed and 20,556,949 facility‐based tests were conducted. Every 1000 distributed HIV self‐tests were associated with an estimated 4.33 additional HIV‐positive diagnoses (95% CI: 4.30–4.35; *p* < 0.001), in the facility‐based tests adjusted model, and 4.61 (95% CI: 4.58–4.63; *p* < 0.001) in the HIV‐negative tests adjusted model. Subgroup analysis showed the largest impact among women aged 25+, in whom every 1000 distributed self‐tests (regardless of the age/sex of recipient) was associated with an additional 38.85 (95% CI: 38.17, 39.52; *p* < 0.001) HIV‐positive diagnoses in the facility‐based tests adjusted model, and 42.69 (95% CI: 42.00, 43.38; *p* < 0.001) in the HIV‐negative tests adjusted model.

**Conclusions**: HIVST was associated with large numbers of HIV‐positive diagnoses at Kenyan health facilities, especially among women aged 25+. These findings support continued integration of HIVST into national HIV programmes. Strengthening routine data systems is essential for ongoing evaluation of HIVST impact.

### OAC0303 Closing the gap: the impact of HIV self‐testing distribution on newly diagnosed cases in South Africa

A. de Nooy ^1^, M. Majam^2^, A. Tembo^2^, K. Bhamidipati^3^, H.‐Y. Kim^3^, T. Chidarikire^4^, S. Rosen^5,6^, WDF. Venter^2^, A. Marsh^7^, A. Bershteyn^3^, C.C. Johnson^8^, B.E. Nichols^1,5,9^

^1^Amsterdam UMC, Global Health, Amsterdam, Netherlands, the, ^2^Ezintsha, Faculty of Health Sciences, University of the Witwatersrand, Johannesburg, South Africa, ^3^New York University Grossman School of Medicine, Department of Population Health, New York, United States, ^4^World Health Organization, Johannesburg, South Africa, ^5^Boston University School of Public Health, Department of Global Health, Boston, United States, ^6^Health Economics and Epidemiology Research Office, Wits Health Consortium, Johannesburg, South Africa, ^7^National Department of Health, Pretoria, South Africa, ^8^World Health Organization, Geneva, Switzerland, ^9^Wits Diagnostic Innovation Hub, Faculty of Health Sciences, University of the Witwatersrand, Johannesburg, South Africa

**Background**: HIV self‐testing (HIVST) provides convenient and confidential HIV testing. However, these characteristics can make evaluating the impact of HIVST investment challenging. We used routine programme data to assess the impact of HIVST based on the number of people confirmed HIV positive at healthcare facilities in South Africa.

**Methods**: The association between district‐level HIVST distribution in 2022 and district‐level confirmed HIV‐positive tests at public healthcare facilities in 2023 was analysed using multivariable linear regression (temporal lag to account for potential delays between self‐testing and facility‐based, provider administered, HIV testing [FTs] uptake). Models were adjusted for total FTs conducted and HIV prevalence. Subgroup analyses stratified models by sex and distribution type, distinguishing between tests intended for the recipient's use (primary) and those for recipient contacts (secondary). Sex and age for FTs and confirmed HIV‐positive tests were unavailable.

**Results**: Routine data showed 884,493 HIVSTs distributed across 34 districts in 2022 and 17,512,893 FTs conducted in all 52 districts in 2023. Every 100 FTs were associated with 2.4 (SE 0.1, *p* < 0.0001) people confirmed HIV positive at healthcare facilities (Table 1). Every 100 HIVST distributed were associated with 3.2 (SE 1.3, *p* = 0.02) additional individuals confirmed HIV positive. Impact was concentrated among men, with 31.0 (SE 13.5, *p* = 0.03) confirmed HIV‐positive individuals per 100 HIVST distributed to men, and no significant association for HIVST distributed to women. Distribution type was not statistically significant: primary HIVST distribution showed a non‐significant increase of 7.5 (SE 4.4, *p* = 0.1); secondary distribution showed a non‐significant decrease of 3.4 (SE 8.4, *p* = 0.69).

**Table 1:** OAC0303: Prevalence‐adjusted multiple linear regression estimates: new facility‐confirmed positives per 100 tests distributed

Model	Explanatory variables	Estimate (number of new facility‐confirmed HIV‐positive cases per 100 tests distributed)	Standard error	*p*‐value
1 [r‐squared 0.94]	Total HIVST 2022 [*n* = 884,493]	3.2	1.3	0.02*
	Facility HIV tests 2023 [*n* = 17,512,893]	2.4	0.1	<0.0001 ***
2 [r‐squared 0.94]	Female [*n* = 449,552]	−10.4	8.8	0.25
	Male [*n* = 290,337]	31.0	13.5	0.03*
	Sex unknown [*n* = 144,604]	−2.3	5.5	0.67
	Facility HIV tests 2023 [*n* = 17,512,893]	2.3	0.2	<0.0001 ***
3 [r‐squared 0.94]	Primary [*n* = 549,945]	7.5	4.4	0.10
	Secondary [*n* = 55,884]	−3.4	8.4	0.69
	Facility HIV tests 2023 [*n* = 17,512,893]	2.4	0.2	<0.0001 ***

**Conclusions**: HIVST is associated with a significant increase in the identification of new HIV‐positive cases in South Africa, with the impact primarily observed among men. Routine HIVST distribution can address gaps in HIV testing and linkage, particularly for engaging men.

### OAC0304 Overcoming stigma: applying peer‐based outreach and linkage strategies to enhance linkage to care after HIV self‐testing (HIVST) among key populations (KP) in Uganda

J.T. Komunyena ^1^, B. Mirembe^1^, P. Kikobye^1^, P. Kyambadde^2^, G. Taasi^2^, D. Canagasabey^3^, C. Tendo‐Bugondo^4^, K. Granger^5^, I. Thior^5^, K. Green^6^

^1^PATH‐Uganda, Primary Health Care, Kampala, Uganda, ^2^Ministry of Health, AIDS Control Program, Kampala, Uganda, ^3^PATH‐Washington DC, Primary Health Care, Washington, DC, United States, ^4^PATH, Primary Health Care, Lubumbashi, Congo, the Democratic Republic of the, ^5^PATH, Primary Health Care, Washington, DC, United States, ^6^PATH, Primary Health Care, Gevena, Switzerland

**Background**: Uganda's KP communities face a higher burden of HIV, with prevalence rates two to five times higher than the general population. Stigma, social exclusion, harsh cultural and political environments limit access to HIV testing and care. Despite the increased accessibility of and privacy offered by HIVST, KP communities still encounter social and self‐imposed stigma that hinders linkage to care. Through the Unitaid HIVST Africa III initiative, PATH and Uganda Ministry of Health leveraged peer‐driven HIVST distribution and follow‐up strategies to improve linkage to testing and care among KPs.

**Description**: The project introduced peer‐based community distribution models, using peer supporters recommended by KP communities who shared similar identities. Peer supporters and their supervisors (healthcare providers/contact points at linkage health facilities) were trained on HIVST, basic counselling techniques, including HIV stigma reduction strategies. Peer supporters distributed HIVST kits at popular congregation points, and through door‐to‐door and community outreach. They leveraged KP networks on social media platforms to share information and videos on HIVST, and used phone calls, WhatsApp or a toll‐free line for client follow up for post‐test counselling and results. Clients with reactive HIVST results were escorted to health facilities by peer supporters and linked to supervisors for guided referrals through confirmatory diagnosis and care.

**Lessons learned**: 104,739 (51%) among 203,377 people provided with HIVST kits from November 2020 through May 2022 were from KP communities. 35,087 (33%) individuals from KP communities self‐tested reactive, among whom 29,578 (84%) were successfully linked to diagnosis and care, reflecting a higher linkage than the national average (79%).

Leveraging peer supporters and facility‐based supervisors trusted among KP communities and training them on HIV stigma reduction counselling was crucial for establishing a foundation of trust that facilitated follow‐up and linkage. Using technology and social media to counsel and follow up with KP communities afforded greater privacy that contributed to higher follow‐up and linkage rates.

**Conclusions/Next steps**: Peer‐led outreach and follow‐up can significantly improve linkage to care following HIVST among KP communities in Uganda, highlighting the importance of introducing and scaling tailored peer‐based interventions and stigma reduction strategies to enhance continuity in HIV care among KP.

### OAC0305 Acceptability and feasibility of HIV self‐testing for partner notification among sexual and drug‐injecting partners of people living with HIV in low‐ and middle‐income countries: a systematic review

L. Khandu ^1^, J. Hallett^1^, G. Crawford^1^, J.E. Leavy^1^, D. Vujcich^2^

^1^Curtin University, School of Population, Faculty of Health Sciences, Bentley, Perth, Australia, ^2^WAAC, Perth, Australia

**Background**: HIV self‐testing (HIVST) enhances HIV diagnosis, but information on its integration for index testing to partner notification is limited in middle‐income countries. Assessing the acceptability and feasibility of index HIVST and partner testing among people living with HIV (PLHIV) and undiagnosed key populations is critical to ending the AIDS epidemic.

**Methods**: A mixed‐method systematic review using a convergent segregated approach was conducted using Joanna Brigs Institute's methodology. Four databases were used for a literature review from October 2023 to March 2024 and included studies published between 2016 and 2023. Rayyan software was used for full‐text screening. Meta‐analysis was found infeasible, but qualitative meta‐aggregation was conducted.

**Results**: A total of 4076 studies were retrieved, and 72 studies met the inclusion criteria after a full review. Most of these studies were from the African region, with only one from South Asia and a few from East Asia. Index HIVST and partner testing were found acceptable and feasible among PLHIV and undiagnosed key populations. Despite low partner elicitation ratios by assisted partner notification, a higher positivity rate among notified individuals was noted. Preferences for index HIVST and partner testing varied with more inclination for assisted and passive partner referrals to overcome the risk concerning HIV status disclosure. The index HIVST showed a low cost per infection averted, indicating that it was a cost‐effective intervention. Findings were skewed towards married couples, with less evidence on unmarried and undiagnosed key populations. The need for uniform policy, legal power and standardized protocol for trained providers was highlighted.

**Conclusions**: Index HIVST were found acceptable and feasible in reaching index partners, including untested and undiagnosed partners of key populations, provided various testing approaches are used. Assisted partner notification resulted in high positivity rates despite low‐index partner elicitation, suggesting the need for various referral options to address social harms, stigma and discrimination. There is a critical need to study unmarried HIV index cases and undiagnosed key populations, particularly in the Southeast Asian region, to bridge HIV case detection gaps. Further research should address the overrepresentation of married couples and focus on key population for a more holistic understanding of index HIVT and partner testing.

### OAC0402 Expanding pre‐exposure prophylaxis (PrEP) access: lessons learned from the introduction of PEPFAR‐supported long‐acting injectable cabotegravir (CAB‐LA) in shaping global prevention efforts

T. Mukherjee ^1^, A. Kimmel^1^, L. Martindale^1^, E. Dorward^1^, R. Eakle^1,2^, C. LoVullo^3^, N. Naqvi^3^, A. Vij^4^, N. Thaweesee^3^, M. Musonda^5^, G. Tidwell^6^, V. Thonyiwa^7^, M. Maulidi^8^, A. Muchara^9^, A. Abubakar^8^

^1^Former USAID, Office of HIV/AIDS, Washington, DC, United States, ^2^London School of Hygiene and Tropical Medicine, Department of Global Health and Development, London, United Kingdom, ^3^Global Health Security and Diplomacy, Department of State, Washington, DC, United States, ^4^Former USAID, Department of State, Washington, DC, United States, ^5^Former USAID, Lusaka, Zambia, ^6^Health Resources and Services Administration, Washington, DC, United States, ^7^Global Health Security and Diplomacy, PEPFAR Coordination Office, Lilongwe, Malawi, ^8^Former USAID, Lilongwe, Malawi, ^9^Former USAID, Harare, Zimbabwe

**Background**: 1.3 million people acquired HIV in 2023, falling short of UNAIDS's goal of fewer than 370,000 individuals by 2025. In fiscal year (FY) 2024, PEPFAR supported the introduction of long‐acting injectable cabotegravir (CAB‐LA) in Eswatini, Malawi, Ukraine, Zambia and Zimbabwe. This analysis summarizes CAB‐LA uptake, oral PrEP to CAB‐LA switching and the contribution of CAB‐LA to overall PrEP uptake.

**Methods**: PEPFAR Monitoring, Evaluation, and Reporting (MER) indicators PrEP_NEW (PrEP naive individuals initiated on PrEP) and PrEP_CT (individuals returning or re‐initiating PrEP at least once that quarter), disaggregated by PrEP type (oral or injectable) were analysed. FY24 results were restricted to quarters that CAB‐LA was available. The number of individuals returning for PrEP was averaged across quarters that CAB‐LA was available in all five countries. Switching from oral PrEP to CAB‐LA was only measured in the first quarter CAB‐LA was introduced in each country using PrEP_CT. Gender and age disaggregations by PrEP type were unavailable.

**Results**: Across all five countries, 441,269 individuals initiated PrEP and, on average, 127,292 returned for PrEP when both PrEP options were available. In FY24, 3772 PrEP‐naive individuals initiated CAB‐LA, and 900 switched from oral PrEP to CAB‐LA in the first quarter of CAB‐LA introduction (Figure 1). Eswatini, Malawi and Zambia reported more PrEP‐naive individuals initiated on CAB‐LA, compared to individuals switching from oral PrEP to CAB‐LA. Compared to the same quarters in FY23, CAB‐LA introduction correlated with a 25% increase in PrEP initiations and a 33% increase in PrEP return.

**Figure 1:**
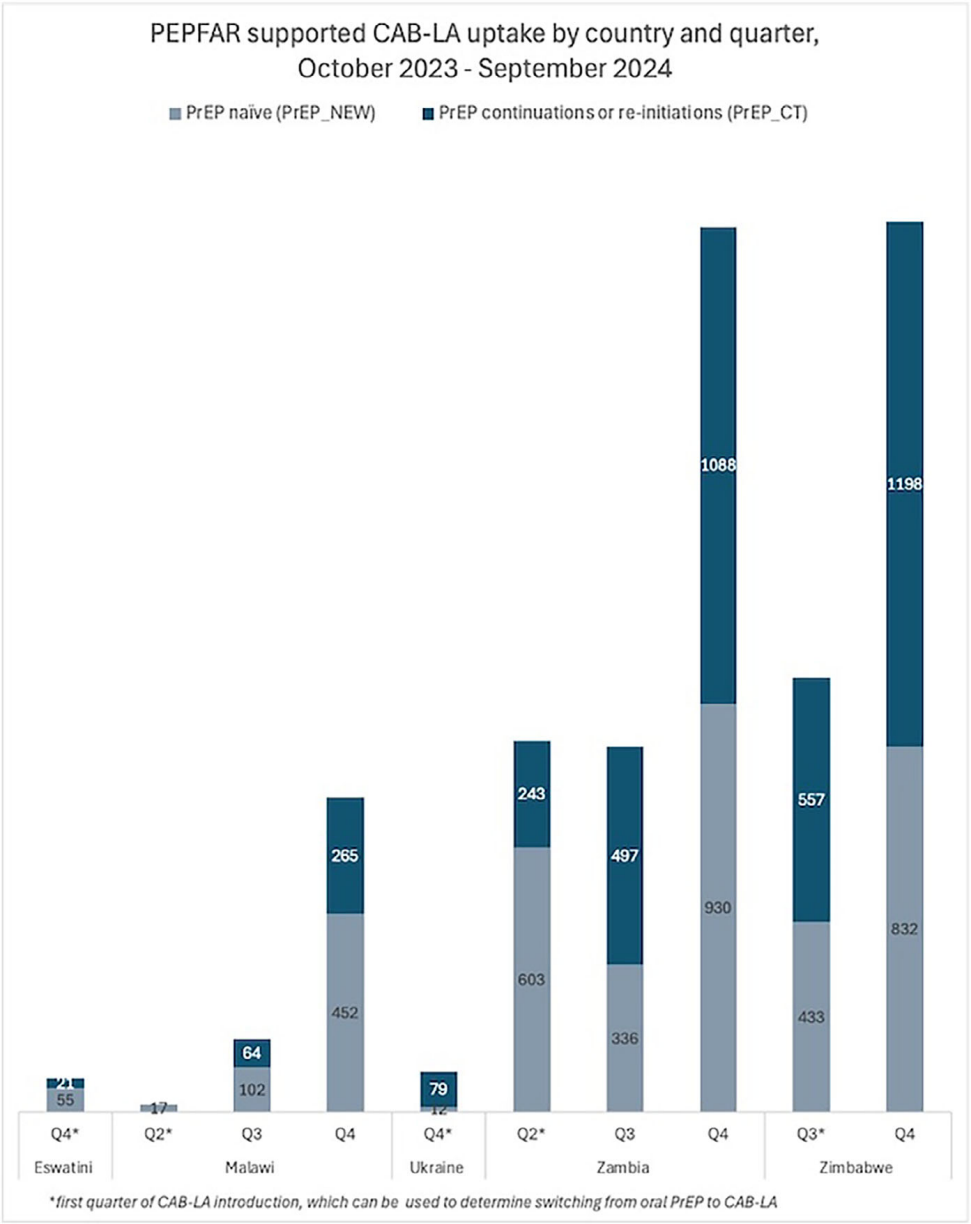
OAC0402

**Conclusions**: The introduction of CAB‐LA demonstrates the potential to expand access and reach PrEP‐naive populations across varying contexts. By diversifying prevention options, CAB‐LA complements existing oral PrEP programming and may catalyse PrEP uptake, enabling broader prevention coverage. Lessons learned from early introduction can inform scale‐up and expansion of long‐acting PrEP, including CAB‐LA and lenacapavir.

### OAC0403 Choices in motion: how young people in the PrEPared to Choose study (Cape Town, South Africa) navigate PrEP product switching

K. Lebelo ^1^, C. Pike^1^, E. Rousseau^1^, P. Macdonald^1^, P. Mapukata^1^, O. Vanto^1^, L.‐G. Bekker^1^

^1^Desmond Tutu Health Foundation, Woodstock, South Africa

**Background**: The introduction of novel, long‐acting HIV pre‐exposure prophylaxis (PrEP), including injectables and the vaginal ring, ushers in an era of PrEP choice. While much focus has been on the initial choice of PrEP product, PrEP users are likely to show dynamic PrEP product preference and use over time. PrEPared to Choose (PtC) is one of the first implementation studies allowing for unscheduled PrEP product switching between three available PrEP formulations.

**Methods**: PtC delivers PrEP choice, including oral PrEP, cabotegravir long‐acting (CAB‐LA) injectable PrEP and the dapivirine‐containing vaginal ring (DVR), to 1164 adolescents and young people (15−29 years) and their intimate male partners in Cape Town, South Africa. HIV counsellors provide comprehensive PrEP counselling that prioritizes accurate PrEP information and supports individual choice. Participants can switch between PrEP products, if clinically eligible, at any time over the 18‐month study period. We report early product switching patterns between baseline and month 3 follow‐up.

**Results**: One thousand one hundred and sixty‐four individuals initiated PrEP, including oral PrEP (*n* = 290), DVR (*n* = 12) and CAB‐LA (*n* = 862). The median age was 24 (20–28) years, with adolescent girls and young women (AGYW) comprising 62.8% of participants. Of 82 oral PrEP users who returned at month 1, 25.61% transitioned to CAB‐LA, with 71.43% of those transitioning being AGYW (see Diagram 1). Of 21 individuals who switched to CAB‐LA, 15 (71.43%) continued with CAB‐LA at month 3. Of 608 individuals who initiated and continued CAB‐LA at month 1, 97.37% remained on CAB‐LA.

**Diagram 1:**
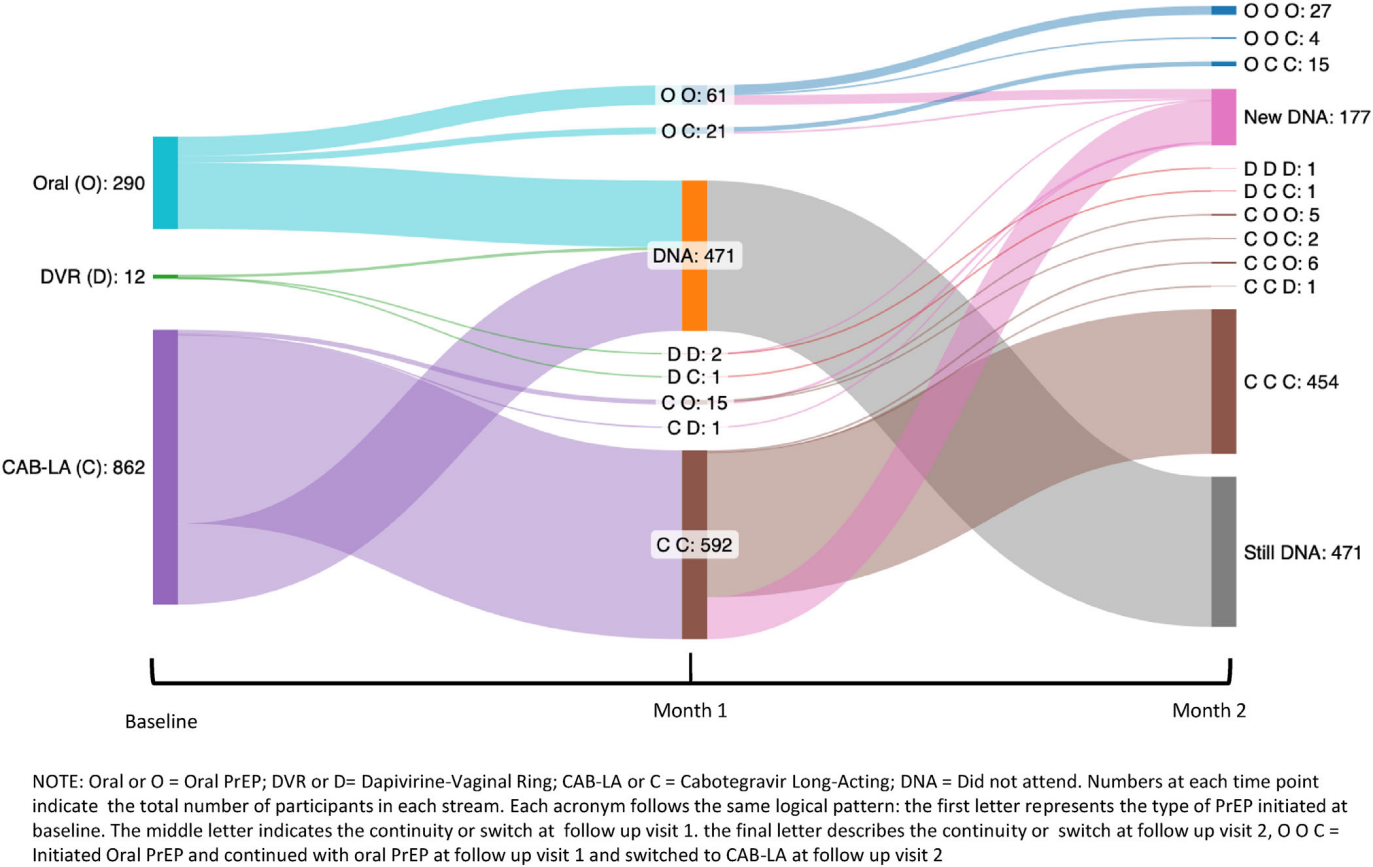
**OAC0403: Pathways of PrEP Usage: Transitions Between Oral PrEP, DVR, and CAB‐LA from Initiation to 3‐month Follow‐up (*N* = 1164)**.

**Conclusions**: Early continuation on PrEP overall and on the initiated PrEP product was substantially greater among PrEP users initiating CAB‐LA compared to oral PrEP. With a quarter of persistent oral users at month 1 opting to switch to CAB LA, it is evident that true choice is an ongoing process that requires appropriate counselling support and clinical management for transitioning between products.

### OAC0404 Continuation rates across different pre‐exposure prophylaxis (PrEP) modalities of cabotegravir long‐acting (CAB‐LA), event‐driven PrEP (PrEP 2‐1‐1), dapivirine ring (DPV‐R) and oral PrEP: evidence from Zimbabwe

M. Munjoma ^1^, J. Mavudze^1^, N. Zimuto^1^, L. Bidi^1^, N. Shoko^1^, G. Ncube^2^, T. Moga^1^, B. Mutede^1^, N. Taruberekera^1^, O. Mugurungi^2^

^1^Population Solutions for Health, Harare, Zimbabwe, ^2^Ministry of Health and Child Care, Harare, Zimbabwe

**Background**: Pre‐exposure prophylaxis (PrEP) is now widely recognized as a viable HIV prevention method globally. In sub‐Saharan Africa, including Zimbabwe, while PrEP coverage is increasing, continuation rates are low. Recent data from Zimbabwe's Ministry of Health shows about 40% continuation at 1 month, dropping to less than 30% by the third month. To address low PrEP continuation, the World Health Organization (WHO) has approved various PrEP options beyond oral PrEP. Population Solutions for Health (PSH), in partnership with the Ministry of Health and with USAID support, conducted a study comparing oral PrEP continuation rates with newly introduced alternatives.

**Methods**: A retrospective cohort study was conducted among high‐risk key populations of adolescent girls and young women (AGYW), female sex workers (FSWs) and men who have sex with other men (MSM) eligible and enrolled across each PrEP modality per WHO guidelines between May 2023 and December 2025. Data were collected using an electronic record management system (Bahmni) and exported into STATA 17 for cleaning and analysis. Cohort analyses were conducted calculating PrEP continuation rates at 1, 2 and 3 months following initiation. Further, 95% confidence intervals were plotted around the point estimates for statistical significance tests across the different proportions.

**Results**: A total of 9609 unique individuals were initiated across the four modalities. The figure below shows continuation rates by PrEP method. CAB‐LA had the highest PrEP continuation rates, followed by dapivirine ring and PrEP 2‐1‐1 with oral PrEP having significantly the lowest continuation rates by the third month (*p* < 0.001).

**Conclusions**: The study showed that PrEP continuation rates differ significantly by PrEP method. The more recent PrEP methods have higher continuation rates and should be scaled up urgently to reduce HIV transmission among high‐risk key populations.[Fig jia226518-fig-0014]

**Figure 1 jia226518-fig-0014:**
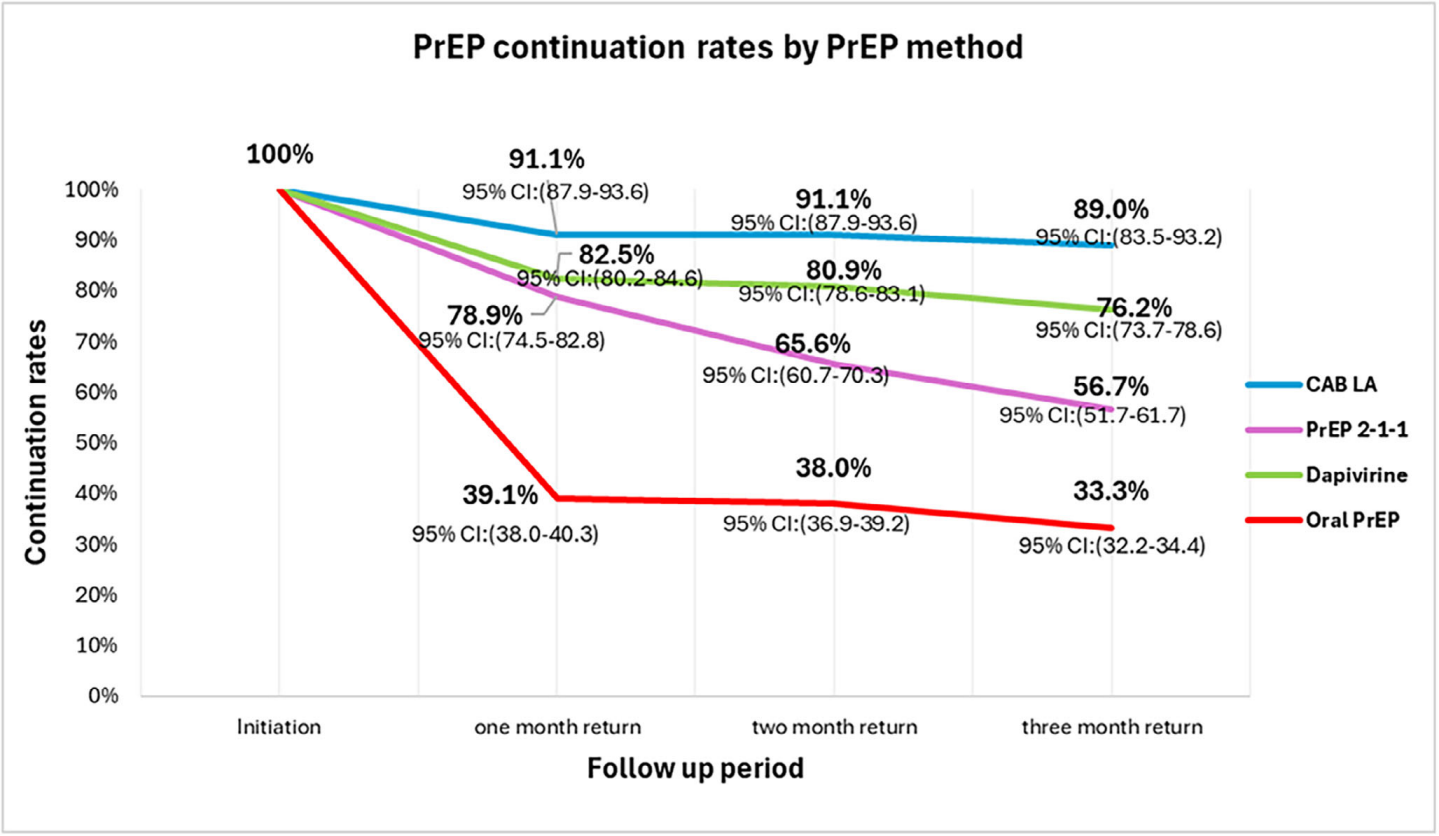
OAC0404

### OAC0405 PrEP awareness, willingness and behavioural characteristics of men who have sex with men and transgender individuals in the Philippines: a latent class analysis of the PrEP‐APPEAL study

R.A. Olete ^1,2^, P. Eustaquio^3^, W. Tieosapjaroen^4^, K. Leyritana^1^, J. Ong^4^, H.‐M.A. Schmidt^5,6^, N. Phanupak^7^, C. Chan^8^, B.R. Bavinton^8^

^1^Sustained Health Initiatives of the Philippines, Mandaluyong City, Philippines, the, ^2^National Cheng Kung University, Department of Public Health, Tainan City, Taiwan, Province of China, ^3^Independent Researcher, Manila, Philippines, the, ^4^Faculty of Medicine, Nursing and Health Sciences, Monash University, Melbourne, Australia, ^5^Joint United Nations Programme on HIV/AIDS (UNAIDS), Geneva, Switzerland, ^6^World Health Organization (WHO), Geneva, Switzerland, ^7^Institute of HIV Research and Innovation (IHRI), Bangkok, Thailand, ^8^Kirby Institute, UNSW Sydney, Sydney, Australia

**Background**: Despite increasing PrEP enrolment in the Philippines, the number of Filipino men who have sex with men (MSM) and transgender individuals (TGIs) using PrEP remains below the national target. This research examined distinct demographic and behavioural patterns associated with PrEP awareness, willingness to adopt specific PrEP options and willingness to pay among Filipino MSM and TGIs.

**Methods**: A total of 1662 Filipino respondents were included from the 2022 multinational cross‐sectional PrEP‐APPEAL dataset. Latent class analysis (LCA) models were developed using STATA, segmenting the population into distinct subgroups based on associations between key variables (i.e. PrEP awareness, willingness towards various PrEP options, e.g. daily, event‐driven or monthly pills, CAB‐LA), willingness to pay and sexual behaviours (e.g. multiple sexual partners, condom use). Subsequently, multinomial logistic regression was employed to identify significant covariates of class membership.

**Results**: Among the respondents, 79.6%(1323/1662) were cisgender men, 5.3%(88/1662) self‐identified as transgender individuals (men and women), mean age was 29.2 years old (SD = 8.00), 17.9%(297/1662) were PrEP‐experienced and 14.0% (297/1662) were currently taking PrEP. LCA revealed three classes of Filipino MSM/TGIs: (1) Unaware and Reluctant (154/1662,9.3%)—with lowest PrEP awareness, lowest willingness to pay, lowest willingness to adopt any PrEP options and lowest number of sex partners; (2) Informed Condom‐Users (1282/1662,77.1%)—with higher PrEP awareness, moderate willingness to daily and monthly oral PrEP but low willingness to CAB‐LA, high condom use and higher number of sex partners; and (3) Enthusiastic Adopters (226/1662,13.6%)—having the high PrEP awareness, substantial willingness to adopt all PrEP options and highest number of sex partners. The Enthusiastic Adopters were more likely to have previously taken PrEP (RRR = 3.64, *p* < 0.001), while the Informed Condom‐Users were more likely from urban areas (RRR = 1.84, *p* = 0.015).

**Conclusions**: Person‐centred community outreach should prioritize increasing PrEP awareness among the “unaware and reluctant,” emphasizing the availability of free oral PrEP and education on all PrEP options. For “Informed Condom‐Users,” address indirect cost barriers and simplify access to free oral PrEP. Introducing CAB‐LA to “Enthusiastic Adopters” with integrated STI test‐and‐treat programmes can mitigate behavioural risks. Holistic strategies that combine education, accessibility and PrEP innovations are essential to achieving national PrEP targets among Filipino MSM and TGIs.

### OAC0502 Estimation of the counterfactual HIV incidence in the PURPOSE trials

C. Kelley ^1^, F. Matovu Kiweewa^2^, O. Ogbuagu^3^, S. Facente^4^, E. Grebe^5^, D.V. Glidden^6^, Y. Shao^7^, Y. Zhao^7^, S. Cox^7^, A. Kintu^7^, L.B. Brown^7^, C.C. Carter^7^, M. Das^7^, L.‐G. Bekker^8^

^1^Division of Infectious Diseases, Emory University School of Medicine and Grady Health System, Atlanta, United States, ^2^Makerere University‐Johns Hopkins University Research Collaboration, Kampala, Uganda, ^3^School of Medicine, Yale University, New Haven, United States, ^4^School of Public Health, University of California, Berkeley, United States, ^5^Vitalant Research Institute, San Francisco, United States, ^6^Department of Epidemiology and Biostatistics, University of California, San Francisco, United States, ^7^Gilead Sciences, Inc., Foster City, United States, ^8^The Desmond Tutu HIV Centre, University of Cape Town, Cape Town, South Africa

**Background**: PURPOSE 1 (NCT04994509;P1) and PURPOSE 2 (NCT04925752;P2) evaluated twice‐yearly lenacapavir for HIV pre‐exposure prophylaxis. These trials employed a novel counterfactual HIV‐incidence design comparing observed HIV incidence among participants receiving investigational agents to estimated background HIV incidence (bHIV) using a recent infection testing algorithm (RITA) in a pre‐randomization cross‐sectional cohort of screened participants. We report bHIV in P1/2, estimated by a RITA, with varying parameters and additional counterfactual estimates.

**Methods**: Samples from participants diagnosed with HIV during screening underwent testing for recent infection; bHIV was calculated using a RITA with recency assay parameters reported in Kassanjee 2016 (mean duration of recent infection and false recent rate based on weighted average of included subtypes; T = 2 years). Multiple counterfactual estimates of HIV incidence were also considered: observed and RITA‐estimated incidence in the ECHO trial, predicted HIV incidence using modified VOICE score among participants (P1) and predicted HIV incidence calculated using emtricitabine‐tenofovir disoproxil fumarate (F/TDF) efficacy‐adherence relationship and correlation with rectal gonorrhoea incidence (P2). Additionally, bHIV was compared using varying recency assay parameters.

**Results**: Estimated bHIV in P1 was 2.41/100 person‐years (py; 95% confidence interval 1.82,3.19) and 2.37/100py (1.65,3.42) in P2 (Figure 1). Predicted HIV incidence in P1 using modified VOICE score was 9.35/100 py (8.11, 11.09); observed HIV incidence in ECHO was 4.77/100 py (4.24, 5.35). Predicted HIV incidence in P2 ranged from 2.87/100 py (1.09, 6.72) using the F/TDF efficacy‐adherence relationship to 6.19 (3.53, 8.85) using rectal gonorrhoea incidence. Varying RITA parameters had minimal impact on the bHIV estimate.

**Figure 1:**
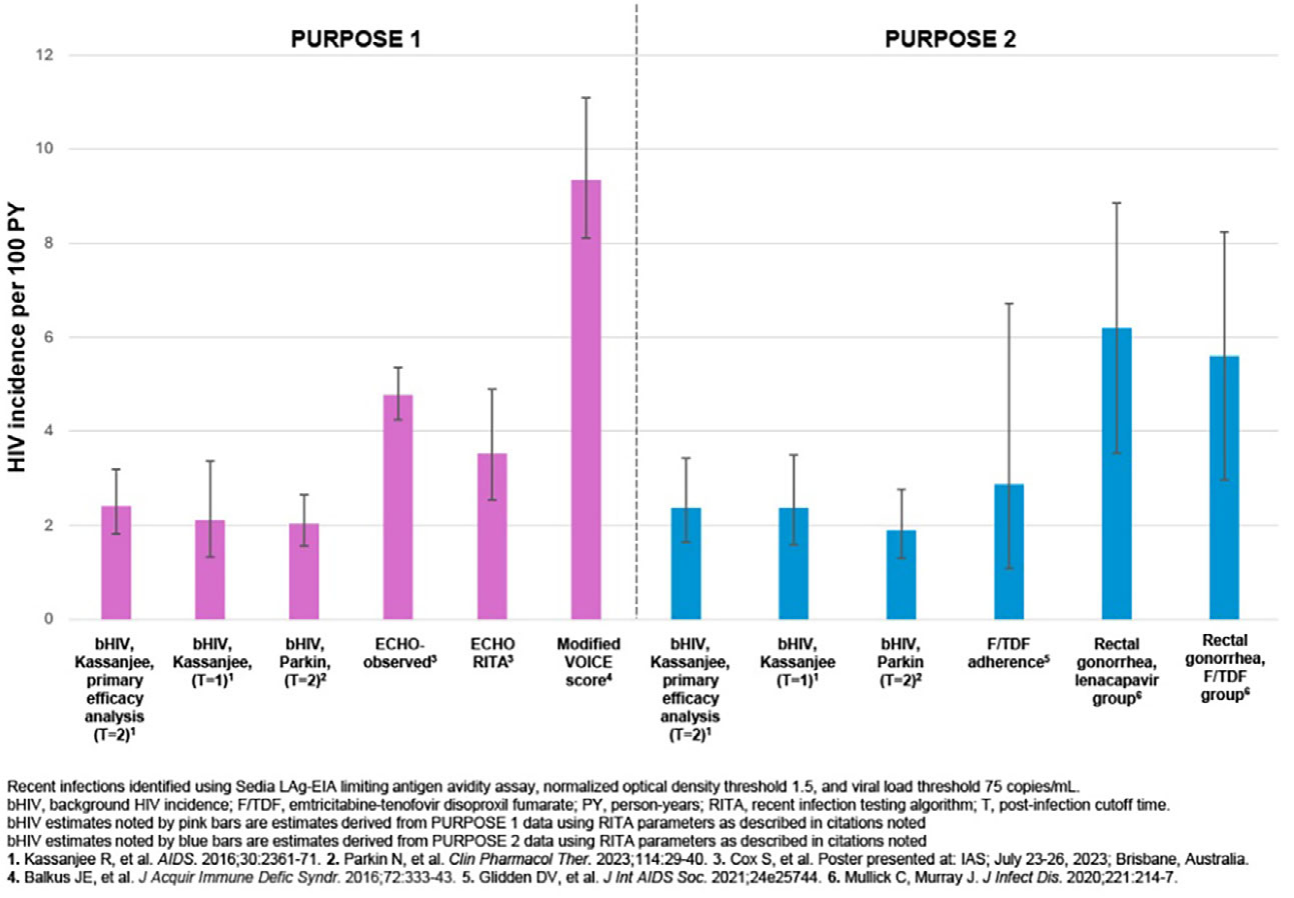
**OAC0502: Comparison of estimated background HIV incidence and 95% Cl using RITA and alternative counterfactual estimates in PURPOSE 1 and PURPOSE 2**.

**Conclusions**: RITA‐based bHIV for P1/2 were consistent with conservative incidence estimates. Of additional counterfactual estimates evaluated, the F/TDF efficacy‐adherence estimate was most similar to RITA‐based bHIV. Modified VOICE score and rectal gonorrhoea correlation yielded higher HIV‐incidence estimates, which may be explained by the calibration of these methods earlier in the HIV epidemic. These findings support RITA‐based bHIV estimates as comparators in future HIV prevention trials.

### OAC0503 Efficacy, safety and pharmacokinetics of twice‐yearly subcutaneous lenacapavir for PrEP among adolescents and young people in the phase 3 trials PURPOSE 1 and PURPOSE 2

K. Gill ^1^, Q. Abdool Karim^2^, S. Anugulruengkitt^3^, L.‐G. Bekker^1^, J.A. Gallardo‐Cartagena^4^, A. Gaur^5^, M. Manentsa^6^, M. Naidoo^2^, D. Potloane^2^, Y. Singh^1^, A. Kintu^7^, P. Wong^7^, Y. Zhao^7^, P. Arora^7^, R. Singh^7^, L.B. Brown^7^, C.C. Carter^7^, M. Das^7^, A. Agwu^8^

^1^The Desmond Tutu HIV Centre, University of Cape Town, Cape Town, South Africa, ^2^Centre for the AIDS Programme of Research in South Africa, University of KwaZulu Natal, Durban, South Africa, ^3^Department of Pediatrics, Faculty of Medicine, Chulalongkorn University, Bangkok, Thailand, ^4^Centro de Investigaciones Tecnológicas, Biomédicas y Medioambientales, Universidad Nacional Mayor de San Marcos, Lima, Peru, ^5^St. Jude Children's Research Hospital, Memphis, United States, ^6^The Aurum Institute, Johannesburg, South Africa, ^7^Gilead Sciences, Inc., Foster City, United States, ^8^Division of Infectious Diseases, Department of Pediatrics, Johns Hopkins University School of Medicine, Baltimore, United States

**Background**: Adolescents and young people are disproportionately affected by HIV globally and experience unique challenges with uptake of, adherence to and persistence on daily oral HIV pre‐exposure prophylaxis (PrEP). PURPOSE 1 (NCT04994509; P1) and PURPOSE 2 (NCT04925752; P2) demonstrated that twice‐yearly subcutaneous lenacapavir was efficacious and safe for PrEP. We describe lenacapavir's efficacy, safety and pharmacokinetics (PK) in participants aged 16−25 years (youth) from both studies.

**Methods**: P1 and P2 were parallel Phase 3 trials assessing lenacapavir for PrEP in cisgender women aged 16−25 years (P1) and cisgender men and gender‐diverse persons aged ≥16 years (P2). We report incident HIV infections and compared a pooled analysis of safety and PK in youth in both studies with that in adults (aged > 25 years) in P2.

**Results**: Among youth, two incident HIV infections occurred on lenacapavir (in P2); six incident infections in P2 and all 55 incident infections in P1 occurred in those on daily oral PrEP. The most common adverse events (excluding injection‐site reactions [ISRs]) were chlamydial infection, upper respiratory tract infection and headache (Table 1). The most common ISRs were nodules, pain, induration and swelling (and erythema in P2 only). Lenacapavir was well tolerated in youth and adults. Lenacapavir plasma concentrations were generally comparable between youth and adults (Figure 1).

**Table 1:** OAC0503: HIV infections, adverse events, and injection‐site reactions in youth vs adults in PURPOSE 1 and PURPOSE 2.

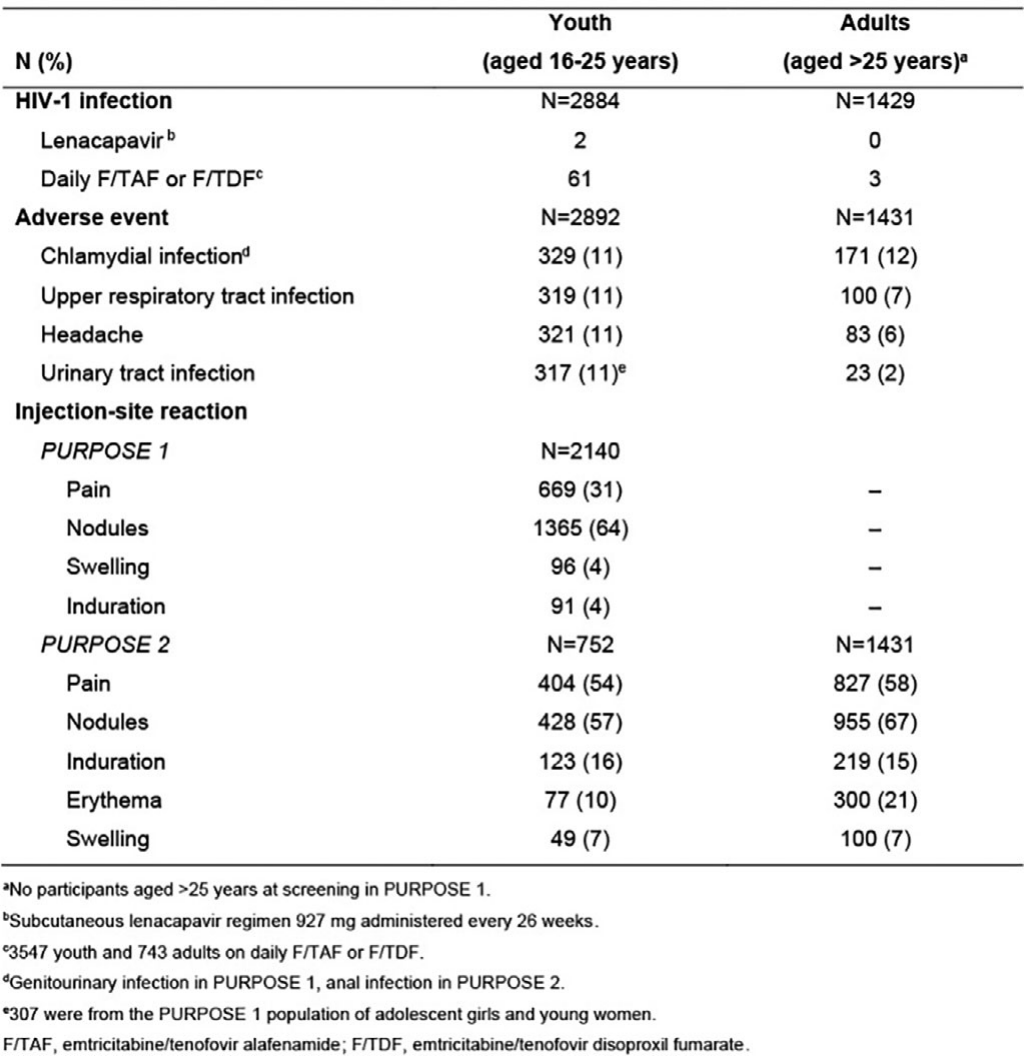

**Figure 1:**
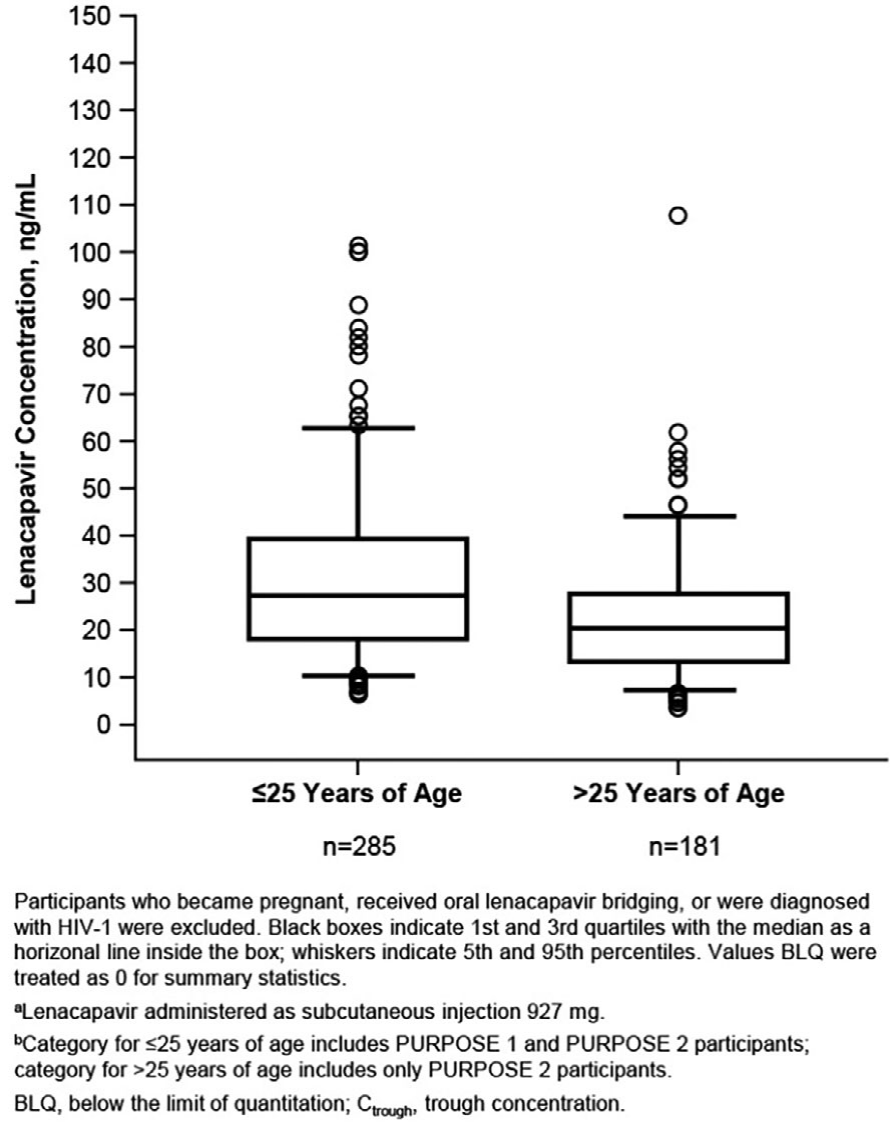
**OAC0503: Boxplots of lenacapavir^a^ plasma concentrations at week 26 (C_trough_) in the randomized blinded phase of the PURPOSE 1 and PURPOSE 2 studies (youth vs adults)^b^**.

**Conclusions**: Twice‐yearly subcutaneous lenacapavir had high efficacy, favourable safety and no clinically relevant PK differences in youth and adults, supporting the potential of lenacapavir to address challenges with daily oral PrEP and help reduce new HIV infections among youth.

### OAC0504 Preference for twice‐yearly injections versus daily oral pills for HIV PrEP in cisgender men, transgender women, transgender men and gender nonbinary people enrolled in PURPOSE 2

K. Mngadi ^1^, N. Phanuphak^2^, J.C. Hinojosa^3^, K. Mounzer^4^, R. Vasconcelos^5^, M. Ramgopal^6^, P. Wong^7^, L.B. Brown^7^, D. Mezzio^7^, C.C. Carter^7^, N. Shah^8^

^1^The Aurum Institute, Clinical Research Tembisa, Tembisa, South Africa, ^2^Institute of HIV Research and Innovation – Pribta Tangerine Clinic, Bangkok, Thailand, ^3^Asociación Civil Selva Amazónica, Iquitos, Peru, ^4^Philadelphia FIGHT Community Health Centers – Jonathan Lax Treatment Center, Philadelphia, United States, ^5^Hospital das Clínicas da Faculdade de Medicina da Universidade de São Paulo, São Paulo, Brazil, ^6^Midway Immunology and Research Center, Fort Pierce, United States, ^7^Gilead Sciences, Inc., Foster City, United States, ^8^Whitman‐Walker Health, Washington, DC, United States

**Background**: Daily oral pre‐exposure prophylaxis (PrEP) is efficacious for HIV prevention; however, consistent adherence, a predictor of effectiveness, can be challenging, especially among some populations disproportionately affected by HIV. In the Phase 3 PURPOSE 2 trial (NCT04925752), twice‐yearly subcutaneous (SC) lenacapavir lowered HIV incidence by 96% compared with background incidence and by 89% compared with oral emtricitabine–tenofovir disoproxil fumarate (F/TDF), with no safety concerns, in cisgender men, transgender women, transgender men and gender nonbinary people who have sex with partners assigned male at birth. We present PrEP administration preferences reported among PURPOSE 2 participants.

**Methods**: Participants were randomized 2:1 in a blinded fashion to receive SC lenacapavir every 26 weeks or oral F/TDF daily, plus an alternative injection/tablet placebo. During injection visits, participants completed an electronic questionnaire about PrEP administration preference (twice‐yearly injections or daily pills) and how administration type impacts HIV risk perception and PrEP adherence. Data were collected at baseline (prior to injection), Week (W) 26 and W52. Categorical responses were analysed descriptively.

**Results**: Of 3271 treated participants, 2918 and 1126 completed the questionnaire at baseline and W52 (primary analysis), respectively. Over 75% of participants preferred twice‐yearly injections over daily pills; 11–15% preferred daily pills (Figure 1). Among those with a preference for injections, over half reported a strong preference. Most participants reported that they would feel more protected from HIV (baseline: 66%; W26: 66%; W52: 69%) and be more confident about not missing a dose (baseline: 76%; W26: 73%; W52: 77%) with twice‐yearly injections versus daily pills. Results were generally consistent across geographies included in the trial.

**Figure 1:**
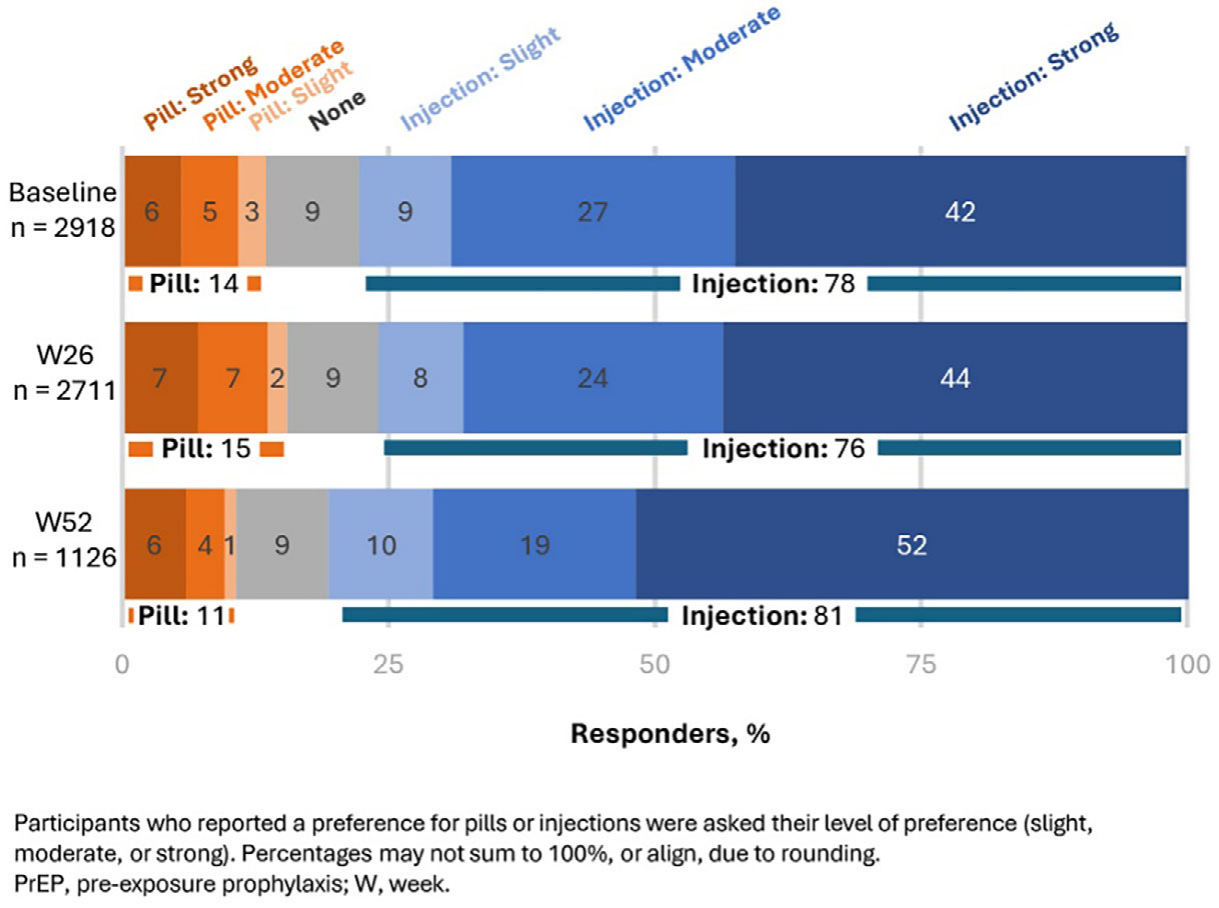
**OAC0504: PrEP administration preferences (daily oral pills vs twice‐yearly injections) among all participants in the PURPOSE 2 trial**.

**Conclusions**: Most participants preferred and felt more protected from HIV with twice‐yearly injectable PrEP, although results highlighted the importance of choice. These data indicate twice‐yearly lenacapavir could increase the uptake of, adherence to, and persistence with PrEP among men and gender‐diverse people.

### OAC0505 Inclusion of pregnant and lactating people in the PURPOSE 1 study: efficacy, safety and pharmacokinetics

L.‐G. Bekker ^1^, D. Moodley^2^, I. Harkoo^2^, G. Kigozi^3^, C.E. Louw^4^, M. Malahleha^5^, T. Palanee‐Phillips^6^, R. Panchia^7^, N. Singh^8^, D.D. Diallo^9^, L. Mworeko^10^, S. Puryear^11^, P. Wong^11^, P. Arora^11^, M. Imperial^11^, C. Deaton^12^, A. Kintu^11^, M. Das^11^, F. Matovu Kiweewa^13^

^1^The Desmond Tutu HIV Centre, University of Cape Town, Cape Town, South Africa, ^2^Centre for the AIDS Programme of Research in South Africa, University of KwaZulu‐Natal, Durban, South Africa, ^3^Africa Medical and Behavioral Sciences Organization, Kalisizo, Uganda, ^4^Madibeng Centre for Research, Brits, South Africa, ^5^Synergy Biomed Research Institute, East London, South Africa, ^6^Wits RHI, University of the Witwatersrand, School of Public Health, Johannesburg, South Africa, ^7^Perinatal HIV Research Unit, University of the Witwatersrand, Soweto, South Africa, ^8^HIV and Other Infectious Diseases Research Unit, South African Medical Research Council, Durban, South Africa, ^9^SisterLove, Inc., Atlanta, United States, ^10^International Community of Women Living with HIV Eastern Africa, Kampala, Uganda, ^11^Gilead Sciences, Inc., Foster City, United States, ^12^Gilead Sciences, Cambridge, United Kingdom, ^13^Makerere University‐Johns Hopkins University Research Collaboration, Kampala, Uganda

**Background**: Pregnant and lactating people (PLP) are disproportionately vulnerable to HIV‐1 acquisition but historically excluded from Phase 3 HIV trials. PURPOSE 1 (NCT04994509) was the first pre‐exposure prophylaxis (PrEP) trial to intentionally include PLP to address their urgent unmet need for HIV prevention options.

**Methods**: We engaged community stakeholders, regulatory agencies, ethics committees and maternal/paediatric health experts to responsibly include PLP in PURPOSE 1. To respect autonomy and reproductive choice, contraception was offered but not required; participants who became pregnant could remain on study drug following additional informed consent. We describe pregnancy outcomes, adverse events (AEs) and HIV infections in PLP randomized to twice‐yearly subcutaneous lenacapavir up to the primary analysis. Lenacapavir plasma concentrations in PLP during each trimester/postpartum were compared with non‐PLP using a population pharmacokinetics (popPK) model. Lenacapavir concentrations in breastmilk and infant plasma were measured (smaller subset).

**Results**: Of 2140 participants receiving lenacapavir, 184 participants had 193 pregnancies, of which 88 (45.6%) were ongoing. The 105 pregnancies with outcomes included 52 live births (49.5%) and 53 losses (50.5%), including 30 induced/elective abortions (28.6%), 20 spontaneous abortions (19.0%) and 3 stillbirths (2.9%). Maternal pregnancy‐associated AEs were uncommon, with gestational hypertension/pre‐eclampsia (*n* = 4) and hyperemesis gravidarum (*n* = 3) most reported. No HIV infections occurred in PLP receiving lenacapavir. In the PopPK analysis, predicted lenacapavir exposure was not statistically significantly different by pregnancy trimester or postpartum status compared with non‐PLP (Table 1). Lenacapavir was present in breastmilk (median milk‐to‐plasma ratio: 0.63 [*n* = 8 matched pairs]); however, lenacapavir exposure in infant plasma was minimal (median breastfed‐infant‐to‐mother plasma ratio: 0.05 [*n* = 11 matched pairs]).

**Table 1:** OAC0505: Summary of model‐predicted lenacapavir exposures stratified by pregnancy group^a^.

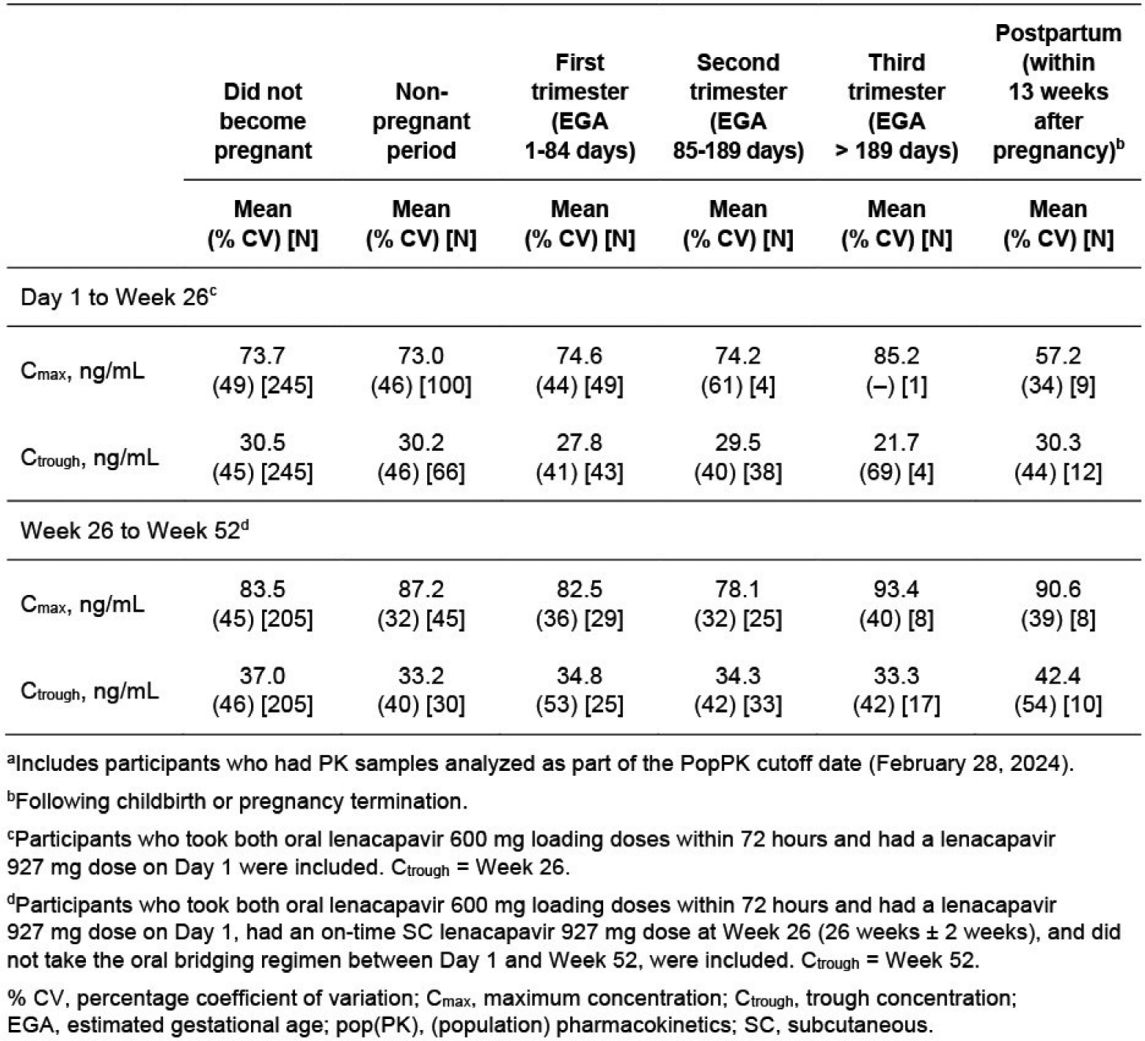

**Conclusions**: Lenacapavir was efficacious, safe and well tolerated, with no clinically significant exposure differences in PLP and minimal exposure in breastfed infants. Proactive evaluation of lenacapavir efficacy, safety and PK data in PLP can support accelerated access to lenacapavir for PLP who need or want PrEP.

### OAC0602 If funding falls short: projecting the impact of international HIV budget cuts across 26 countries

D. ten Brink ^1^, R. Martin‐Hughes^1^, A. Bowring^1^, N. Wulan^1^, S. Dalal^2^, N. Scott^1^

^1^Burnet Institute, Melbourne, Australia, ^2^World Health Organization, Geneva, Switzerland

**Background**: International funding for HIV has been critical in reducing new HIV transmissions and deaths. While treatment financing is commonly prioritized domestically, HIV prevention and testing, especially for key populations, remain more vulnerable to cuts in international funding. Reductions in international aid of 10%−70% (19% weighted average) have been announced commencing in 2026, in the five countries that account for over 90% of international HIV funding. We investigated the impact of these funding reductions on the HIV epidemic in 24 countries through mathematical modelling.

**Methods**: We used existing, country‐validated Optima HIV models in 24 countries (Albania, Armenia, Azerbaijan, Belarus, Bhutan, Cambodia, Colombia, Cote d'Ivoire, Eswatini, Georgia, Kazakhstan, Kenya, Kyrgyzstan, Malawi, Malaysia, Moldova, Mongolia, Mozambique, South Africa, Sri Lanka, Tajikistan, Uganda, Uzbekistan, Zimbabwe). We compared a status quo scenario, with most recent HIV spending continued from 2024 to 2040, to scenarios with 18.8% cuts to international funding from 2026. Country‐specific impacts of funding cuts on HIV prevention and testing programme coverage were derived based on the proportion of international funding, with lower bounds assuming equal spending reductions across all programmes and overheads and upper bounds assuming all spending reductions directly impacted service coverage. Treatment and facility‐based testing were assumed to continue through domestic funding. We projected HIV incidence, HIV‐related deaths and cascade outcomes to 2040.

**Results**: Across the 24 countries considered, a 19% reduction in international funding could lead to a 10−64% reduction in HIV prevention and testing programme coverage, a cumulative increase of 14−68% in new HIV transmissions and a 4−13% in HIV‐related deaths across 2026−2040. In addition, there was a 4−21% increase in total people living with HIV and a 2−16% increase in people on ART by 2040 (despite lower diagnosis rates), which could lead to higher long‐term domestic financial costs. Impacts were greatest in countries with a higher percentage of international funding, and those with increasing incidence among key populations.

**Conclusions**: Persistent reductions in international HIV funding could significantly reverse progress by 2040, disproportionately affecting key and vulnerable populations. Establishing sustainable funding mechanisms to safeguard prevention, testing and care programmes is critical to reducing HIV transmission and mortality.

### OAC0603 How have policies influenced PrEP uptake? An analysis of 139 countries and 33 African countries

J. Tailor ^1^, K. Segal^1^, W. Nyagah^2^, M. Warren^1^

^1^AVAC, New York City, United States, ^2^AVAC, Nairobi, Kenya

**Background**: Oral PrEP is at varying stages of scale‐up globally, as longer‐acting PrEP products enter the market in limited supply. This analysis evaluates which policies significantly enable PrEP uptake to understand how to expand access to PrEP options.

**Methods**: Data on cumulative PrEP initiations (dependent variable) for 139 countries, as of September 2024, was collected from AVAC's Global PrEP Tracker. Data on the status of 54 policies (independent variable [IV]) related to HIV testing and prevention, structural changes and health systems across all 139 countries, as of July 2024, was collected from the HIV Policy Lab. Multiple and individual regressions, using RStudio (2024.12.0+467), assessed the significance of policies altogether and individually (*p* = 0.05). A separate analysis was done for 33 African countries. Correlation analyses of IVs assessed multicollinearity using the Pearson coefficient. Ridge regression analysis assessed if multicorrelated variables should be eliminated from the model to remove confounding in multiple regressions.

**Results**: The global analysis showed that eight policies on task‐shifting (1), decriminalizing same‐sex sex (3), decriminalizing HIV exposure (3) and HIV self‐testing (1) were significantly associated with PrEP uptake in individual regressions. Multiple regression showed only one policy on task‐shifting is significantly associated with PrEP uptake.

The analysis of African countries showed that four policies decriminalizing HIV exposure (2), prohibiting compulsory HIV testing (1) and loosening age restrictions on testing and treatment (1) were significantly associated with PrEP uptake in individual regressions. None were significantly associated with PrEP uptake in multiple regression.

The discrepancy in the individual and multiple regressions indicates multicollinear IVs. Correlation analyses confirmed that at least four pairs of policies are highly correlated in enabling PrEP uptake. Ridge regression results showed that these same variables were very strongly contributing to the predictive power of the model and cannot be eliminated.

**Conclusions**: Policies found to significantly enable PrEP uptake making access easier, reduce discrimination and align with user preferences. While the effect of individual policies is reduced when considering several altogether, they influence PrEP uptake both individually and systemically. Differences in policies that significantly enable PrEP uptake at global and regional levels imply that context affects which policies should be implemented.

### OAC0604 Evaluating global PrEP implementation progress over time: an analysis of the PrEP‐to‐Need ratio from 2018 to 2023 across 11 countries with high HIV incidence and/or burden

L. Sheira ^1^, C. Verde Hashim^2^, J. Tailor^3^, M. Prochazka Nunez^4^, H.‐M.A. Schmidt^4,5^

^1^University of California, San Francisco, School of Nursing, San Francisco, United States, ^2^AVAC, London, United Kingdom, ^3^AVAC, New York, United States, ^4^World Health Organization, Geneva, Switzerland, ^5^UNAIDS, Geneva, Switzerland

**Background**: Pre‐exposure prophylaxis (PrEP), which is highly effective at preventing HIV, could end the HIV epidemic if used at scale effectively and by those who need it. Despite market availability for at least 5−10 years globally, implementation has varied, with substantial differences between countries in terms of meeting in‐country PrEP need. Comprehensive data comparing countries over time can unlock critical insights in implementation facilitators and challenges.

**Methods**: Using UNAIDS data on HIV incidence and AVAC data on PrEP initiations (2018−2023), we calculated the PrEP‐to‐Need ratio (PNR) among all countries with available data (higher = greater PrEP coverage than HIV incidence). We categorized the PNR as leading (> 5), growing (1.00−4.99) and emerging (< 1.00) based on prior work by AVAC. Among countries with high PrEP volumes (initiations > 100,000 cumulative) in 2023, we compared the PNR ratio over time by PNR category.

**Results**: Of 111 countries with PrEP‐to‐need data available, 11 were high PrEP‐volume countries: six were leading (Kenya, Lesotho, Malawi, Uganda, Zambia and Zimbabwe) and five were growing (Brazil, Mozambique, Nigeria, South Africa, Tanzania); none were emerging. While HIV incidence was stable or decreasing across all countries over time, increases in PrEP initiations drove the PNR. Transition to leading took an average of 4.4 years; for growing countries, average time from emerging to growing was 3.8 years.

**Conclusions**: Leading countries were characterized by consistent and sustained increases in PrEP initiations per year. Countries in the growing category must increase their PrEP delivery substantially and among populations at substantial HIV acquisition risk to become leading. Targeting a minimum of five PrEP initiations among those with PrEP need per projected incident HIV case will promote higher PNR. Data in aggregate form may mask epidemics in key populations (KPs); specific, disaggregated coverage data for key populations needed to further characterize progress in meeting key PrEP goals among KPs.[Fig jia226518-fig-0018]

**Figure 1 jia226518-fig-0018:**
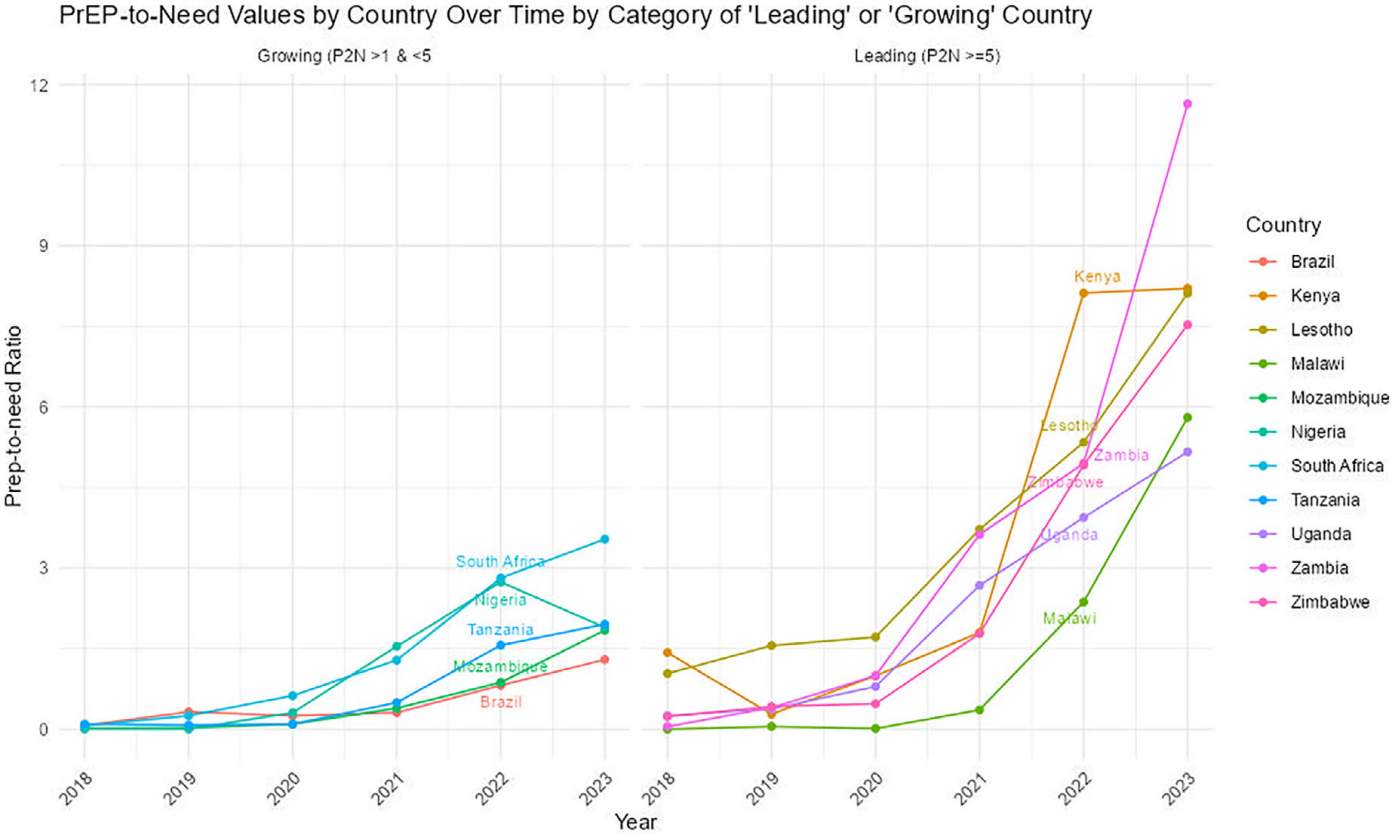
OAC0604

### OAC0605 Viral load suppression at first viral load after shifting from 6 to 3 months post‐ART initiation: a retrospective analysis of early outcomes in the South African public sector HIV programme

K. Rees ^1,2^, B. Mugisa^3^, C. O'Connor^1^, N. Davies^1^, L.S. Wilkinson^4,5^

^1^Anova Health Institute, Johannesburg, South Africa, ^2^University of the Witwatersrand, Department of Community Health, School of Public Health, Johannesburg, South Africa, ^3^USAID, Pretoria, South Africa, ^4^International AIDS Society, Geneva, Switzerland, ^5^University of Cape Town, Centre for Infectious Epidemiology and Research, Cape Town, South Africa

**Background**: In April 2023, South Africa updated anti‐retroviral (ART) guidelines to advance the first viral load (VL) from 6 to 3 months post‐initiation. This aimed to leverage dolutegravir's faster suppression rates and enable earlier enrolment into differentiated service delivery (DSD) models and adherence support. We evaluated the impact of this guideline change on VL suppression rates, and DSD enrolment and timing in four South African districts.

**Methods**: We analysed ART initiations May−December 2023, excluding those recorded as deceased, transferred out or with a baseline VL (suggesting prior treatment and/or re‐engagement) but including those who may have disengaged during follow‐up (168 days following guideline‐mandated first VL timing). VL< 200 copies/ml, VL suppression (< 50 copies/ml) and DSD enrolment were analysed using logistic regression. DSD enrolment timing was assessed using a Cox proportional hazards model, adjusting for age, gender and district.

**Results**: Among 26,091 individuals initiating ART with a VL, 65.1% achieved VL suppression and 87.8% had VL< 200 copies/ml with a 3‐month VL, compared to 66.9% and 86.2% with a 6‐month VL. Adjusted analysis showed that clients with VLs taken at 6 months had 13% higher odds of suppression (*p*‐value < 0.01) and 41% lower odds of DSD enrolment compared to those at 3 months. Median time to enrolment was 162 days for clients with suppressed VLs taken at 3 months compared to 252 days for 6 months.

**Conclusions**: Advancing the first VL to 3 months enabled earlier DSD enrolment for suppressed ART initiators, supporting retention efforts. Although suppression rates were slightly lower at 3 compared to 6 months, earlier VL also enables expedited identification of clients who could benefit from targeted adherence support and require follow‐up testing. Maximizing the benefits of an earlier VL requires promptly offering DSD or multi‐month dispensing to all suppressed clients and providing proactive, early support for unsuppressed clients.[Table jia226518-tbl-0007]

**Table 1 jia226518-tbl-0007:** OAC0605: Viral suppression and DSD enrolment by VL timing

Category	Early (29–77 days)	3 months (78–112 days)	3–6 months (113–161 days)	6 months (162–196 days)	Late (> 196 days)
**VL < 200 copies/ml (%)**	1671 (80.07%)	11,813 (**87.75** **%)**	3814 (83.33%)	3194 **(86.23%)**	1833 (81.07%)
Adjusted OR (*p*‐value, 95% CI)	0.57 (< 0.01, 0.51−0.64)	Ref	0.73 (< 0.01, 0.67−0.80)	0.93 (0.18, 0.83−1.03)	0.66 (< 0.01, 0.58−0.74)
**Suppressed VL < 50 copies/ml (%)**	1191 (57.07%)	8758 **(65.06%)**	2946 (64.37%)	2476 **(66.85%)**	1451 (64.18%)
Adjusted OR (*p*‐value, 95% CI)	0.71 (< 0.01, 0.64−.078)	Ref	0.99 (0.89, 0.93−1.07)	**1.13 (< 0.01, 1.05**−**1.22)**	1.02 (0.63, 0.93−1.13)
**Suppressed enrolled in DSD (%)**	473 (39.71%)	4322 (49.35%)	1109 (37.64%)	895 (36.15%)	412 (28.39%)
Adjusted OR (*p*‐value, 95% CI)	0.68 (< 0.01, 0.60–0.77)	Ref	0.63 (< 0.01, 0.58–0.69)	**0.59 (0.01, 0.53–0.64)**	0.42 (< 0.01, 0.37–0.48)
**Suppressed median time to DSD enrolment (days)**	143	**162**	196	**252**	306
Adjusted HR (*p*‐value, 95% CI)	1.12 (0.02, 1.02−1.24)	Ref	0.65 (< 0.01, 0.61−0.70)	0.44 (< 0.01, 0.40−0.47)	0.36 (< 0.01, 0.32−0.39)

### OAD0102 Mental health challenges and sexual health resource access among LGBTQ+ young people in Peru: findings from an online national survey

J.C. Jauregui ^1^, F. León‐Morris^2,3^, M. Reyes‐Diaz^2,3^, R. Nath^4^, A.B. Taylor^4^, K.A. Konda^2,5^

^1^University of California, Los Angeles, Social Welfare, Los Angeles, United States, ^2^Investigaciones Médicas en Salud, Lima, Peru, ^3^Universidad Peruana Cayetano Heredia, Center for Interdisciplinary Studies in Sexuality, AIDS and Society, Lima, Peru, ^4^The Trevor Project, West Hollywood, United States, ^5^Keck School of Medicine, University of Southern California, Population and Public Health Sciences, Los Angeles, United States

**Background**: The purpose of this study is to investigate how clinically significant levels of depression (PHQ‐2), anxiety (GAD‐2) and lifetime suicidal ideation are associated with access to sexual health resources among LGBTQ+ young people (ages 14−24) in Peru.

**Methods**: Data were drawn from a national mental health survey of LGBTQ+ young people in Peru (*n* = 4643) conducted from October to December 2022 with funding and collaboration from The Trevor Project (USA) and ethical approval from INMENSA (Peru). Logistic regression was used to assess the association between mental health indicators (depression, anxiety and lifetime suicidal ideation) and access to three sexual health resources: (1) pre‐exposure prophylaxis (PrEP); (2) HIV/STI treatment/consultation; and (3) condoms. Each outcome was dichotomized comparing: wanted but did not receive versus wanted and received resource. Adjusted odds ratios (aOR) were calculated, controlling for key socio‐demographic variables.

**Results**: Most of the 4643 respondents were 18 years or older (62.5%), 16.1% were transgender or non‐binary, 48.9% identified as bisexual and 28% as gay or lesbian. Most had clinically significant depression (58.2%), anxiety (54.6%) and 73.4% endorsed lifetime suicidal ideation. Some wanted each sexual health resource: PrEP 40.6%, HIV/STI treatment/consultation 47.7% and condoms 55.4%. In the PrEP model, individuals with a history of suicidal ideation were 43% less likely to access PrEP. In the HIV/STI treatment/consultation model, no mental health variables were significantly associated with access. In the condom access model, anxiety was associated with a 25% increase, and depression to a 25% decrease in the likelihood of condom access (Table 1).

**Table 1:** OAD0102: Models examining the associations between sexual health access (PrEP, STI/HIV treatment, condoms) and mental health indicators

Model 1: *n* = 1447, 17% accessed PrEP		Model 2: *n* = 1720, 30% accessed STI/HIV treatment or consultation		Model 3: *n* = 1986, 40% accessed condoms	
	aOR (95% CI)		aOR (95% CI)		aOR (95% CI)
Depressive symptoms (Ref: No)	1.06 (0.74−1.54)	Depressive symptoms (Ref: No)	1.11 (0.84, 1.46)	Depressive symptoms (Ref: No)	**0.75 (0.60**−**0.95)**
Symptoms of anxiety (Ref: No)	1.24 (0.87−1.78)	Symptoms of anxiety (Ref: No)	1.07 (0.82−1.40)	Symptoms of anxiety (Ref: No)	**1.25 (1.00**−**1.58)**
Lifetime suicide ideation (Ref: No)	**0.57 (0.39**−**0.83)**	Lifetime suicide ideation (Ref: No)	0.77 (0.57−1.03)	Lifetime suicide ideation (Ref: No)	0.90 (0.70−1.16)

*Note*: Bolded aOR and 95% CI reflects statistically significant associations (*p* < 0.05). All models adjusted for age, sex assigned at birth, gender, region of residence, migration status, education, employment and socio‐economic status. Depression was measured using the PHQ‐2 and anxiety using the GAD‐2; both measures were dichotomized, with a “yes” indicating a score of ≥3 (range: 0−6), which reflects clinically significant symptoms.

**Conclusions**: Our findings highlight mental health needs among LGBTQ+ Peruvians and their potential connection with sexual health resource access. Suicidal ideation was associated with impeded PrEP access, while anxiety and depression were associated with differential condom access. These results emphasize the need for integrated mental health and sexual health services to address the unique needs of LGBTQ+ young people, particularly those facing severe mental health challenges.

### OAD0103 Addressing alcohol use to improve mental health among men living with HIV (LWH): impact of the Kisoboka intervention on depressive symptoms among fisherfolk in Uganda

A.P. Miller ^1^, D. Tuhebwe^1,2^, M. Ediau^2^, R. Naigino^1,2^, A. Ancho^2^, K. Sileo^3^, N. Tumwesigye^2^, E. Reed^1^, J.A. Hahn^4^, R. Wanyenze^2^, S.M. Kiene^1^

^1^San Diego State University, San Diego, United States, ^2^Makerere University School of Public Health, Kampala, Uganda, ^3^Boston College, Boston, United States, ^4^University of California, San Francisco, San Francisco, United States

**Background**: Harmful alcohol use and depression are interrelated barriers to optimal ART adherence. Kisoboka, a multilevel intervention aimed at reducing alcohol use and improving HIV treatment outcomes through economic strengthening, unexpectedly reduced depressive symptoms. We examined whether its impact on depression varied by baseline alcohol use.

**Methods**: We conducted a pilot randomized controlled trial to evaluate Kisoboka. We enrolled fisherfolk men, aged 18−50, LWH, reporting suboptimal adherence and recent hazardous alcohol use, between January 2021 and March 2022. Linear generalized estimating equations (GEE) were built to examine potential moderation of intervention effect on depressive symptoms (measured using the CESD) over time (baseline, 3 and 6 months follow‐up) in adjusted models using a three‐way interaction term (time*arm*baseline AUDIT).

**Results**: Analysis of 160 participants and 466 observations revealed a significant three‐way interaction between continuous AUDIT score, time and study arm on depressive symptoms (B = −0.0970, 95% CI −0.1555, −0.0386, *p* = 0.001). To illustrate this effect (Figure 1), we plotted the probability of depressive symptoms over time for participants with higher (+1 SD) and lower (−1 SD) baseline AUDIT scores by study arm. Among those with higher AUDIT scores, the probability of depressive symptoms in the intervention decreased from 57% to 3% at 6 months, while it increased in the control arm from 37% to 66%. For participants with lower AUDIT scores, the probability of depressive symptoms declined in the intervention (27% to 2%) and slightly improved in the control arm (44% to 33%).

**Figure 1:**
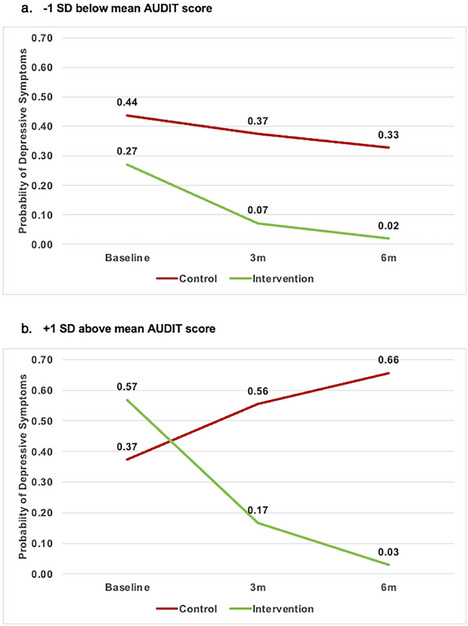
**OAD0103: a‐b. Probability of Depressive Symptoms by Time and Condition**.

**Conclusions**: The intervention's mental health benefits were most pronounced among those with higher baseline alcohol use. These findings suggest that addressing alcohol use may play a crucial role in improving mental health outcomes. Furthermore, the intervention's impact on depressive symptoms varied by alcohol use severity, highlighting the potential need for tailored approaches to maximize effectiveness across different risk profiles.

### OAD0104 The double burden: a cross‐sectional study of the intersection between mental health and access to HIV prevention services for female sex workers in internally displaced camps in Calabar, Nigeria

L. Efobi ^1,2^

^1^University of Nigeria, Clinical Pharmacy and Pharmacy Management, Nsukka, Nigeria, ^2^Person‐Centered HIV Research Team, Nsukka, Nigeria

**Background**: Female sex workers (FSWs) are vulnerable to HIV acquisition, occupational discrimination and mental health challenges. However, understanding the intersection of mental health and their access to HIV prevention services will guide policymakers in improving accessibility of FSWs in internally displaced persons (IDP) camps having a dual burden. This study assessed the relationship between mental health and accessibility of HIV prevention services for FSWs in IDP camps in Calabar, Nigeria.

**Methods: ​**A cross‐sectional study was conducted on 187 randomly selected FSWs in IDP camps in Calabar (December 2024−January 2025). A three‐part validated 17‐item questionnaire measuring mental health status, perceived discrimination and accessibility to HIV prevention services was used to collect data. Data analysis involved frequencies, cross‐tabulations and chi‐square tests using IBM SPSS (version‐27), with significance set at *p* < 0.05.

**Results**: Out of 187 FSWs, 108 participated in the study. Most participants, 25 (23.1%), were aged 25−34 and 39 (36.1%) had worked for > 6 years. There were 43 (39.8%), and 42 (38.9%), respondents who sometimes or often, respectively, felt sad, down or hopeless in the past month. Substance use was reported as the primary coping strategy for stress (*n* = 91, 84.3%). Occupational discrimination was prevalent, with 63.9% and 19.4% reporting frequent and occasional experiences, respectively. HIV testing (79 [73.1%]) and condom use (65 [60.2%]) were common, while PrEP access was low, 9 (8.3%). Among condom users, 50.8% sometimes felt sad, while 44.2% of non‐users often felt sad, linking mental health and access. Age influenced access to services (*p* = 0.001), with condom use highest in 25‐ to 34‐year‐olds (29.2%), but lowest in those < 18 years (10.8%). Likewise, HIV testing was more accessed by 25−34 years (27.8%) and < 18 years (26.6%), and least in > 45 years (8.9%). Respondents older than 45 years (83.3%) reported not accessing any HIV prevention services. Perceived discrimination correlated with poor access, especially among those with over 6 years’ experience; 83.3% reported no access (*p* = 0.001).

**Conclusions**: Mental health, perceived discrimination and access to HIV prevention services intersect in FSWs living in IDP camps, especially in older, more experienced populations, suggesting a need for age and experience‐specific interventions. Future research should investigate underlying factors contributing to low service utilization in older populations.

### OAD0105 Depression, suicidal ideation and HIV risk among young mothers in South Africa

A.P. Miller ^1^, Z. Essack^2^, D. Joseph Davey^3,4^, C. Groenewald^2^, Z. Petersen^2^, L.M. Filiatreau^5^, H. van Rooyen^6^

^1^San Diego State University, San Diego, United States, ^2^Human Sciences Research Council, Pretoria, South Africa, ^3^University of California, Los Angeles, Los Angeles, United States, ^4^University of Cape Town, Cape Town, South Africa, ^5^Washington University in St. Louis, St Louis, United States, ^6^University of Washington, Seattle, United States

**Background**: The burden of mental illness in African countries is rising, driven by poverty, unemployment and limited upward mobility. Many women face high rates of intimate partner violence (IPV), unplanned pregnancies and motherhood‐related stresses, exacerbating mental health challenges. Poor mental health can lead to reduced quality of life, disability, and, in severe cases, death. Depression and suicidal ideation are linked to high‐risk behaviours, including sexual activities that increase HIV risk. Despite these dynamics, few studies have examined mental health and HIV risk in African mothers.

**Methods**: This study examined associations between depression, suicidal ideation and HIV risk behaviours among pregnant and/or parenting young South African women. Data were collected from unemployed women aged 18–24 years as part of an evaluation in KwaZulu‐Natal (June–September 2018). The analysis included those reporting sexual activity in the past year. Baseline data were analysed using Poisson regression, with primary exposures of past year (i) prolonged sadness and (ii) suicidal ideation. The composite HIV risk outcome included transactional sex, partners with unknown HIV/STI status and substance‐influenced sex. Models adjusted for age, IPV and HIV serostatus.

**Results**: We analysed data from 1026 participants. Median age was 22 years (IQR: 21–23), with a mean of 1.1 children. Three in 10 reported ever drinking alcohol (29.7%). Past year IPV was reported by 9.1% and HIV prevalence was 8.7%. Two percent (*n* = 17) were currently pregnant, and most (97%) indicated their most recent pregnancy was unplanned. One‐third (34%) reported prolonged sadness, while 10% reported suicidal ideation. In unadjusted models, prolonged sadness and suicidal ideation in the past year were associated with increased composite HIV risk, with PRRs of 2.11 (95% CI: 1.75–2.52) and 1.99 (95% CI: 1.58–2.47), respectively. Adjusted models showed HIV risk PRRs of 1.79 (95% CI: 1.41–2.27) for sadness and 1.67 (95% CI: 1.24–2.27) for suicidal ideation.

**Conclusions**: Depression and suicidal ideation are critical challenges for unemployed pregnant or parenting young South African women. Antenatal, postnatal, well‐baby and HIV care offer crucial opportunities for mental health screening and referral, which can reduce HIV risk and improve long‐term health for both mothers and children.

### OAD0202 High stigma in one area strongly predicts high stigma in others: testing the idea of a “universal” approach to anti‐stigma intervention to improve healthcare for key populations

A. Adam ^1^, D. Callander^2,3^, D. Kirongo^2^, C. Kithinji^1^, A. Guadarrama^4^, K. Makobu^5^, L. Ledenski^2^, S. Dombojena^2^, M. Pastrana^4^, K. Khabala^2^, J.P. Ouamba^2^, I. Ciglenecki^4^, G. Mwendar^2^, P. Owira^6^

^1^Mombasa County Department of Health, Mombasa, Kenya, ^2^Médecins Sans Frontières, Mombasa, Kenya, ^3^University of New South Wales, Sydney, Australia, ^4^Médecins Sans Frontières, Geneva, Switzerland, ^5^Kenya Medical Research Institute, Kilifi, Kenya, ^6^International Centre for Reproductive Health, Mombasa, Kenya

**Background**: While traditional anti‐stigma intervention has used education around specific topics (e.g. sexuality), some contend this topical approach is ineffective for healthcare workers already overwhelmed with information. Instead, theorists argue for stigma as a universal concept best addressed through interventions promoting overall acceptance and equity. To explore the possibility of a universal anti‐stigma intervention, our study examined different stigmas among healthcare workers in the Kenyan city of Mombasa. Specifically, we tested the hypothesis that high stigma in one domain would predict high stigma in others.

**Methods**: A prospective cohort of healthcare workers from six public clinics in Mombasa was established (June 2023−December 2024). Every 3−6 months, participants completed a survey of attitudes towards stigmatized topics. Scale scores were standardized from 1 (low stigma) to 5 (high stigma), and a factor analysis with varimax rotation investigated potential stigma groupings. Multivariable regression analyses investigated associations between different stigmas while controlling potential confounders (i.e. age, education, social desirability).

**Results**: Over four waves, 925 data points were collected (*n* = 395). The factor analysis defined two stigma groups: experiences (i.e. mental health, abortion, early pregnancy, sexual violence; λ = 2.45) and populations (i.e. sex workers, people who use drugs, sexual and gender minorities; λ = 2.20). Strong two‐way associations were observed between all topics. For example, high sex work stigma predicted high sexual minority stigma (aOR = 6.7, 95% CI: 2.6−17.6), while stigma towards people who use drugs predicted gender minority stigma and vice versa (aOR = 4.4, 95% CI: 2.0−5.5). Even across factors, strong associations were observed: in a composite measure, high experience‐based stigma was associated with a 3.9 times greater likelihood of high population stigma (aOR = 3.9, 95% CI: 1.9−7.8).

**Conclusions**: Study results support our hypothesis, suggesting stigma can be understood as a “universal” experience. Anti‐stigma interventions should focus on promoting acceptance overall rather than engaging with specific topics, which offers exciting possibilities to improve healthcare for key populations and beyond.[Fig jia226518-fig-0020]

**Figure 1 jia226518-fig-0020:**
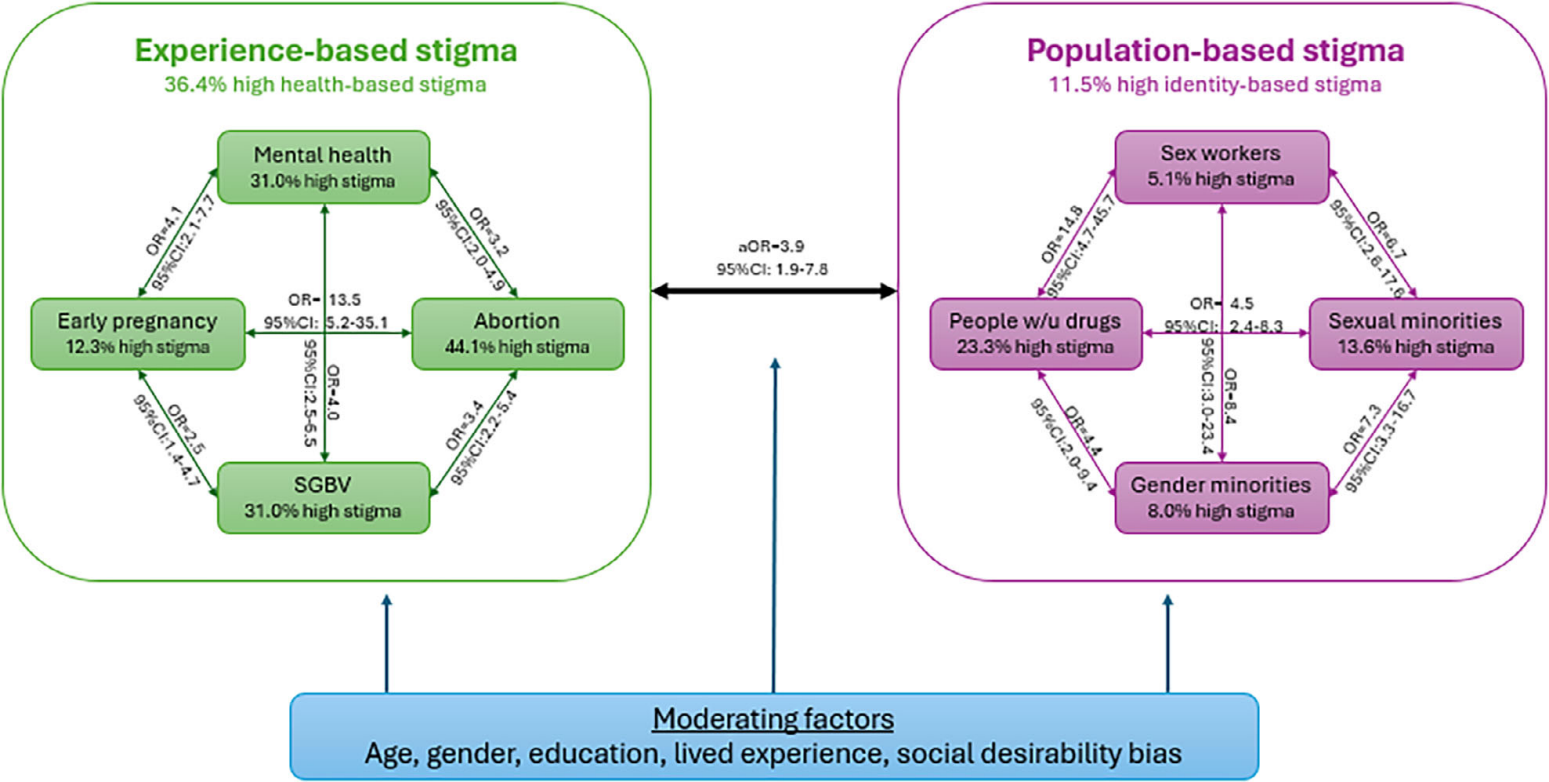
OAD0202

### OAD0203 Promoting equity for pregnant adolescents in research: ethics guidance from the PREPARE working group

R.V. Masese ^1^, H.C. Baron^1^, A.D. Lyerly^1^, S. Day^2^, The PREPARE Working Group

^1^University of North Carolina at Chapel Hill, School of Medicine, Department of Social Medicine, Center for Bioethics, Chapel Hill, United States, ^2^University of North Carolina at Chapel Hill, Department of Medicine, Division of Infectious Diseases, Chapel Hill, United States

**Background**: Exclusion of pregnant adolescents from clinical HIV research participation has led to significant evidence gaps in the safety, effectiveness, and uptake of HIV treatment and prevention strategies for this population. These gaps underscore the urgent need for ethics guidance to support the inclusion of pregnant adolescents in clinical HIV research. Inclusive research is essential to inform safe and effective treatment and prevention strategies and improve health outcomes for pregnant adolescents and their children.

**Description**: To address this urgent need, the PRomoting Equity for Pregnant Adolescents in REsearch (PREPARE) Working Group, an international and interdisciplinary team of 44 experts, has developed actionable ethics guidance for the equitable and responsible inclusion of pregnant adolescents in clinical HIV research. This guidance incorporates diverse expertise and perspectives from bioethicists, adolescent and HIV researchers, policy experts and adolescents with lived experience.

**Lessons learned**: The guidance comprises nine recommendations based on a reproductive justice framework, highlighting the ethical principles of respect, access and protection. Per the principle of respect: (1) pregnant adolescents should be central to decisions about research participation; (2) studies must address the full range of their reproductive options; (3) their lived experiences should inform the design, conduct and reporting of research.

Per the principle of access: (4) trials involving pregnant adults should be designed to include pregnant adolescents unless exclusion is strongly justifiable; (5) age alone should not determine inclusion; decisions should rely on scientific rationale, public health priorities and cultural context; (6) post‐trial access to therapeutics must be ensured.

Per the principle of protection: (7) exclusion harms must be considered during study reviews; (8) innovative study designs, such as phased inclusion, should be utilized where safety concerns would typically result in exclusion; (9) study design should consider what specific data are needed to determine safety among pregnant adolescents.

**Conclusions/Next steps**: Pregnant adolescents require earlier and intentional inclusion in research to ensure timely access to the benefits of advancements in HIV treatment and prevention. Adopting these recommendations will support their ethical inclusion in research, promote responsible study design, ensure access to appropriate therapies and improve health outcomes for both pregnant adolescents and their children.

### OAD0204 We fear the day this bill is passed: the criminalization of LGBTQ+ and its effect on public health efforts in Ghana

N.A. Acheampong ^1^, E.O. Ankrah^1^

^1^Ghana AIDS Commission, Research, Monitoring and Evaluation, Accra, Ghana

**Background**: The criminalization of lesbians, gay, bisexual, transgender and queer (LGBTQ+) individuals remains a global issue with discriminatory laws, stigma and violence affecting their wellbeing, health and human rights. Ghana is currently in the process of criminalizing LGBTQ+. A bill proscribing LGBTQ+‐related activities was proposed to the parliament of Ghana in 2021. The bill proposes to promote proper human sexual rights and family values whiles proscribing all LGBTQ+‐related activities. The bill as it stands now prohibits all forms of advocacy for LGBTQ+. Thus, making it illegal to provide HIV prevention services to LGBTQ+. The prevalence of HIV in Ghana is 1.3%, compared to 18.1% among men who have sex with men (MSM). We sought to investigate the effects of LGBTQ+ criminalization on HIV prevention and treatment efforts in Ghana.

**Description**: We elicited respondents’ perceptions of the effect the bill will have on MSM and HIV‐related activities in May 2024. We conducted 26 semi‐structured in‐depth qualitative interviews with peer social networks of MSM within Greater Accra Region. A thematic analytical framework was used to analyse audio recordings.

**Lessons learned**: The major findings indicated that MSM will be less willing to access HIV prevention services from drop‐in centres due to fear of being arrested. It was indicated that stigma and discrimination currently discourage the majority of MSM from accessing HIV prevention services. Further criminalization of LGBTQ+‐related activities will worsen the situation. Respondents also reported that the majority of MSM have gone underground out of fear of penalization due to their sexual behaviour. This poses a challenge for public health organizations to implement targeted intervention and outreach programmes for MSM.

**Conclusions/Next steps**: Laws criminalizing all forms of LGBTQ+ advocacy can fuel the HIV epidemic as they dissuade MSM from seeking treatment. Penalization of same‐sex intercourse also contributes to a cycle of stigma and discrimination. Such a hostile legal and social environment will exacerbate mental health issues among LGBTQ+ individuals, leading to higher rates of stress, anxiety and depression. The criminalization of LGBTQ+ in Ghana will significantly impact the country's HIV response.

### OAD0205 New HIV diagnoses, social determinants of health and systemic racism by US region 2013−2021

C. Garrett^1^, Z. Li^2^, H. Xia^1^, B. Olatosi ^3^, X. Li^1^, S. Qiao^1^

^1^University of South Carolina, Health Promotion, Education, and Behavior, Columbia, United States, ^2^Penn State, Geography, University Park, United States, ^3^University of South Carolina, Health Services Policy and Management, Columbia, United States

**Background**: The social determinants of health (SDOH) and systemic racism are drivers of health inequities but remain under investigated in relation to HIV acquisition across geographic regions. We aim to assess the effect of SDOH inequities and systemic racism on new HIV diagnosis rates across the five major regions of the United States.

**Methods**: Publicly available data (2013−2021) from AIDSVu and ACS were merged using unique, county‐level FIPS codes (i.e. geographic identifier). County‐level data were extracted and aggregated to the regional level. A generalized estimation equation model was run, within each region, to explore the associations between the SDOH, systemic racism and new HIV diagnoses across time. Variables included in the final model were age group among men; race/ethnicity; poverty; less than a high school degree; no insurance coverage; and measures of residential segregation: Gini, Delta, Spatial Proximity Index.

**Results**: Overall, 3013 counties across the United States were included in the analysis (i.e. Deep South = 712, South = 636, North = 237, West = 398, Midwest = 1030). Prior to the COVID‐19 pandemic, county‐level rates of new HIV diagnoses were highest in the Deep South, declining in the North and South, and rising in the West. New diagnoses fell in all regions during the pandemic (2019−2020), reflecting service interruptions, and have since began rising (2021), nearing pre‐pandemic levels. By region, counties with a higher proportion of men aged 35−54 (Deep South: IRR = 1.07, West: 1.06, Midwest: 1.14, North: 0.96; *p* ≤ 0.05) and Black populations (Deep South: 1.45, South: 1.46, North: 1.17, West: 1.49, Midwest: 2.62; *p* ≤ 0.001) were associated with higher rates of new HIV diagnoses. Systemic racism was found to be positively associated with new HIV diagnoses in all regions but the Midwest. Two interaction terms were found, Black x Delta (Deep South: 1.05, West: 1.28, North: 0.92; *p* ≤ 0.01) and Poverty x Gini (Deep South: 0.91, South: 0.89, Midwest: 0.65, North: 1.08; *p* ≤ 0.001).

**Conclusions**: These findings reflect regional patterns of interplay between SDOH, systemic racism and new HIV diagnoses. Overall, these findings inform regionally tailored public health efforts that address health disparities and systemic racism due to their effect on new HIV diagnoses.

### OAD0302 Community‐based participatory research analysis with transgender individuals as peer research associates in a qualitative study on female sex workers’ health and rights in Argentina

E. Panizoni ^1^, V. Zalazar^1^, N. Cardozo^1^, M. Duarte^1^, S. Fabian^1^, R. Acuña^1^, C. Serrao^1^, M. Loufty^2^, M. Romero^3^, G. Orellano^4^, M.I. Figueroa^1^, P. Cahn^1^, S. Walmsley^5^, A. Duran^6^, V. Fink^1^, I. Aristegui^1^, MAS por Nosotras Study Group

^1^Fundacion Huesped, Research Department, Buenos Aires, Argentina, ^2^University of Toronto, Women's College Hospital, Toronto, Canada, ^3^Asociación de Travestis, Transexuales y Transgénero de Argentina, Research Department, Buenos Aires, Argentina, ^4^Sindicato de Trabajadorxs Sexuales de Argentina, Buenos Aires, Argentina, ^5^University of Toronto, University Health Network, Toronto, Canada, ^6^Ministry of Health, Coordination of Sexual Health, HIV and Sexually Transmitted Infections, Buenos Aires, Argentina

**Background**: The DEPICT model is a collaborative, inclusive qualitative health research analysis approach integrating community and researcher perspectives to inform health research. This study aimed to describe the adaptation and benefits of the DEPICT model with the transgender community in the context of “MAS por Nosotras,” a project on the sexual and reproductive health of female sex workers with and without HIV in Argentina after COVID‐19 pandemic.

**Methods**: We adapted and followed the six sequential steps of the DEPICT model (Dynamic reading, Engaged codebook, Participatory coding, Inclusive reviewing, summarizing categories, Collaborative analysis, and Translating). We analysed 24 interviews conducted with policymakers and key actors at local and national levels to explore the perceptions of access to healthcare and financial aid during and after COVID‐19 pandemic using Monitoring of Results for Equity (MoRES) and Conservation of Resources Theory (COR) frameworks.

**Results**: Six steps were adapted and one added: 1. Training and organization: in methodology analysis of DEPICT for the social researchers (SR) and peer research associates (PRA), ensuring the approach is understood. We organized two pairs, one SR and one PRA for each. 2. Dynamic reading: team members reading the transcripts with an open mind, actively engaging, asking questions, identifying themes and linking ideas. 3. Engaged codebook selection: selecting the MoRES and COR frameworks 4. Participatory coding: analysing two/three daily interviews to complete the 24 transcripts. 5. Inclusive reviewing and summarizing categories: SR and a third researcher harmonizing summaries. 6. Collaborative analysing: results analysis and incorporation into policy briefs to amplify their impact. 7. Translating: Performing a Deliberative Dialogue to disseminate and translate the knowledge into concrete actions. Benefits included a collaborative learning environment, strengthening the defence of community values, empowering teamwork, facilitating implementation and improving research rigour.

**Conclusions**: Adapting the DEPICT model to the transgender community highlighted the potential of participatory approaches in addressing complex health and social challenges. Fostering collaboration between researchers and PRAs, this model enhanced analysis rigour while empowering the community. Incorporating a deliberative dialogue ensured research findings were translated into actionable policies, ensuring that the voices and needs of vulnerable populations were central to decision‐making processes.

### OAD0303 Unlocking success: recruitment and retention of CHWs for effective HIV prevention in West Virginia

H. Arnold ^1^, T.A. Young^1^, J. Lucas^1^

^1^Community Education Group, Lost City, United States

**Background**: The opioid crisis has increased HIV outbreaks from injection drug use in West Virginia. In response, the Community Health and Mobilization Prevention Program (CHAMPS) fostered a network recruitment model addressing the crisis in hard‐to‐reach areas. CHAMPS recruited individuals with histories of substance use and incarceration while serving socially and geographically isolated populations. This approach expanded access to education, resources and peer education, strengthening community resilience.

**Description**: CHAMPS was established in Washington, D.C., by the Community Education Group (CEG) to address health disparities faced by communities of colour. Training 150+ community health workers (CHWs), screening 10,000+ for HIV and 5000+ for HCV annually, achieving a 90% linkage and retention rate. CEG adapted CHAMPS to address underserved and geographically isolated Appalachian communities. By incentivizing CHAMPS graduates for referrals, CHAMPS recruited, trained and retained BIPOC individuals, former substance users and the formerly incarcerated. Notably, 35% of all CHAMPS graduates were recruited by earlier participants, highlighting sustainability.[Table jia226518-tbl-0009]

**Table 1 jia226518-tbl-0009:** OAD0303

Participants graduated:	185
% Participants graduated:	89%
% Grads w/history of substance use:	53%
% Grads identifying as female:	69%
% Grads identifying as BIPOC:	35%
% Grads w/history of incarceration:	41%

Recruitment efforts prioritize areas with the highest overdose mortality rates, strategically placing graduates in communities most vulnerable to HIV outbreaks. Utilizing graduates’ social networks as a recruitment strategy has facilitated high participation in outreach, with an ∼82% retention rate. Graduates complete a comprehensive 147‐hour CHW paid training programme, including 22+ hours of specialized HIV education, equipping them with the knowledge and skills to address the opioid crisis.

**Lessons learned**: CHAMPS successfully recruited 35% of graduates across four cohorts through alumni, prioritizing vulnerable individuals. CEG's model proved effective, with increased alumni referrals and strong graduate engagement in outreach within their already established but newly trained networks.

**Conclusions/Next steps**:

- Expand CHAMPS throughout Appalachia and areas with similar overdose and HIV risks.
- Leverage the CHAMPS workforce for future HIV interventions and outbreak response.
- Evaluate CHAMPS’ long‐term impact on HIV prevention and care in Appalachia.

### OAD0304 Breaking barriers: a community‐led policy to address structural determinants of HIV care in rural Ghana

N. Azebah ^1^

^1^Ejura Municipal Hospital, Midwifery, Kumasi, Ghana

**Background**: Structural determinants such as stigma, geographic inaccessibility and limited resources continue to impede equitable access to HIV prevention and care in rural Ghana. In Navrongo, an indigenous community in the Upper East Region, these barriers disproportionately affect adolescents, women and key populations. This policy intervention aimed to dismantle these barriers by integrating community voices into health system strengthening while fostering sustainable, inclusive HIV care practices.

**Description**: Between 2023 and 2024, this initiative leveraged a community‐driven model to adapt UNAIDS‐recommended frameworks into localized policy action. Key activities included:

1. Stakeholder Integration: Active involvement of people living with HIV, traditional leaders and healthcare seekers in policy design and implementation.
2. Capacity Building: Comprehensive training for healthcare workers on person‐first approaches, U = U strategies and tackling intersectional stigma.
3. Mobile Access: Deployment of mobile clinics that provided HIV testing, counselling and antiretroviral therapy (ART) initiation, reaching underserved households.
4. Cultural Alignment: Culturally tailored communication campaigns that emphasized the role of community cohesion in health outcomes.

**Lessons learned**: The intervention achieved measurable success:

- Uptake of Services: A 45% increase in HIV testing rates, with 98% of people living with HIV starting ART within 30 days of diagnosis.
- Stigma Mitigation: A 70% reduction in reported stigma among healthcare seekers, as assessed through community surveys.
- Community Empowerment: Traditional leaders and local organizations played pivotal roles in normalizing HIV discussions and sustaining intervention efforts.
- Policy Adaptability: The approach highlighted the value of flexible, locally informed policies that reflect specific community needs.

Challenges included limited financial resources and the need for scalable mental health integration.

**Conclusions/Next steps**: This initiative underscores the potential of community‐led, culturally sensitive policies to transform HIV prevention and care in rural settings. By addressing structural determinants, this approach aligns with UNAIDS’ 95‐95‐95 targets, bridging gaps in health equity for people living with HIV. Future plans focus on scaling this model to other underserved regions, expanding mental health services and increasing investment in mobile health delivery systems. These findings affirm the critical importance of collaborative, inclusive policymaking to advance global HIV care objectives.

### OAD0305 Community‐driven approaches to reducing HIV stigma and enhancing care

A. Alitsi ^1^

^1^Tangaza University, Social Science, Nairobi, Kenya

**Background**: In Nairobi's informal settlements, young people face significant challenges related to HIV stigma and access to care. This project aimed to explore community‐driven interventions to tackle these issues and improve wellbeing. The scope included a grassroots initiative from January 2022 to December 2023, focusing on adolescents and young adults (15−24 years). Objectives were to reduce HIV stigma, improve treatment adherence, and enhance community awareness and participation.

**Description**: From January 2022 to December 2023, we launched a grassroots HIV education and support programme in Nairobi's informal settlements. The initiative targeted adolescents and young adults (15−24 years) and involved peer education, training community health workers and collaborating with local organizations. Key activities included interactive workshops, personalized counselling sessions and distribution of educational materials. The programme aimed to reduce stigma, improve treatment adherence and enhance HIV knowledge.

**Lessons learned**: Peer education built trust, with over 150 workshops reaching about 3000 young people. Training local community health workers led to a 25% increase in HIV treatment adherence. Collaborative workshops with local organizations increased community participation by 40%, enhancing HIV knowledge and attitudes. Interventions reduced stigma by fostering open discussions and creating safe spaces, leading to a 30% decrease in self‐reported stigma.

**Conclusions/Next steps**: The findings highlight the significance of community‐driven approaches in addressing social and behavioural barriers to HIV prevention and care. The reduction in stigma, improved treatment adherence and increased community participation demonstrate the effectiveness of these interventions.

Future Implications:

- Leverage Peer Education: Continue involving young peer educators to build trust and share accurate information.
- Involve Community Health Workers: Train and support local health workers to ensure ongoing, personalized care and support.
- Enhance Local Partnerships: Collaborate with local organizations to maximize resources and broaden the programme's impact.
- Scale Up: Expand these initiatives to reach more informal settlements and other vulnerable communities.

Scaling up these community‐driven initiatives could further reduce stigma and improve health outcomes for young people in informal settlements, significantly contributing to the overall goal of HIV prevention and care. These findings underscore the importance of community involvement in achieving sustainable and impactful health interventions.

### OAD0402 Health disparities among migrant transgender women: results from TransCITAR cohort study in Argentina

I. Aristegui ^1^, E. Panizoni^1^, R. Caballero^1^, E. Frontini^1^, R. Acuña^1^, M.C. Trejo^1^, S. Fabian^1^, N. Cardozo^1^, C. Cesar^1^, V. Fink^1^, V. Zalazar^1^, M.I. Figueroa^1^, TransCITAR Study Group

^1^Fundacion Huesped, Research Department, Buenos Aires, Argentina

**Background**: Transgender woman (TGW) migrants faced increased health disparities, including stigma and limited healthcare access. Studies on migrants’ health in Latin America remain scarce. This study analysed psychosocial and clinical factors associated with migration (international and internal) in TGW participating in the TransCITAR cohort study in Argentina.

**Methods**: From September/2019 to December/2022, TGW enrolled in TransCITAR prospective cohort completed baseline data on socio‐demographics, HIV status, transgender‐related stigma (TIS), depression (SCL‐27 sub‐scale), suicide attempts, substance use (tobacco use, sexualized drug use [SDU], alcohol [AUDIT‐C]). Chi‐squared tests with studentized residuals and ANOVA with Tukey's post hoc test were used to compare groups (international, internal/non‐migrants).

**Results**: Among 423 TGW (Table 1), internal migrants comprised 46.80% (*n* = 198), international 31.21% (*n* = 132, 18.7% with permanent residency, 66.7% Argentine national ID) and non‐migrants 21.99% (*n* = 93). Internal migrants were younger (M = 31.03 ± 8.10 years) than international migrants (34.23 ± 9.16, *p* = 006). HIV prevalence was highest in international (53.0%) and internal migrants (42.7%; *p* < .001). Sex work prevalence was 68.7% for internal migrants, 53.8% in international migrants and 22.6% in non‐migrants (*p* < .001). Unstable housing was more frequent in migrants (both 50.5%) than non‐migrants (8.6%, *p* < .001). Depressive symptoms and suicide attempts were more frequent in internal migrants (*p* = .036, *p* = .013). Substance use, including tobacco (52.6%) and SDU (36.6%), was significantly higher among internal migrants (*p* < .001). Discrimination by security forces was more frequent among internal migrants (*p* = .003), and levels of transgender stigma in international migrants (*p* = .006).

**Table 1:** OAD0402: Descriptive statistics and comparison of socio‐demographic, psychosocial and clinical characteristics among migrants (international and internal) and non‐migrants

Characteristics	Total *n* = 423 (100%)	Non‐migrants *n* = 93 (21.99%)	International migrants *n* = 132 (31.21%)	Internal migrants *n* = 198 (46.80%)	*p*‐value
Financial aid support, yes	190 (45.0)	33 (33,5)^a^	41 (31.1)^a^	116 (58.9)^b^	**.000**
Gender identity discrimination for security forces, yes	95 (22.5)	15 (16.1)^a^	21 (15.9)^a^	59 (29.8)^b^	**.003**
Transgender Identity Stigma score, M (SD)	6.05 (7.37)	6.34 (6.88)^a,b^	4.39 (5.94)^b^	7.02 (8.25)^a^	**.006**
Depressive Symptoms Sub‐scale Score, M (SD)	0.55 (0.63)	0.54 (0.58)^a,b^	0.45 (0.54)^a^	0.63 (0.70)^b^	**.036**
Suicide attempts, lifetime, yes	128 (26.4)	30 (21.6)^a^	28 (20.9)^a^	70 (33.2)^b^	**.013**
Tobacco use, yes	206 (40.6)	53 (38.1)^a^	42 (31.3)^a^	111 (52.6)^b^	**.000**
Sexualized drug use (last month), yes	159 (36.6)	34 (27.4)^a^	32 (27.6)^a^	93 (47.9)^b^	**.000**
Alcohol use score total (last month), M (SD)	3.56 (2.87)	3.94 (3.00)^a^	3.57 (2.82)^a,b^	2.75 (2.52)^b^	**.004**

*Note*: ^a,b,c^ Categories that share the same letter do not have statistically significant differences in proportions within that row.

**Conclusions**: TGW migrants face significant health disparities, with international migrants showing high HIV prevalence and internal migrants exhibiting worse social and health outcomes. Tailored interventions are critical to improving the HIV continuum of care for international migrants.

### OAD0403 HIV transmission risk, sexual and mobility behaviours among men, women and female sex workers living with HIV in informal gold mining sites in Mali: the ANRS‐12392 – Sanu Gundo study

L. Sagaon‐Teyssier^1^, M.A. Guindo^2^, A. Kamissoko^2^, F. Traoré^2^, G. Maradan^3^, F. Cavaro^1^, F. Diallo^2^, Z. Diarra^2^, M. Mora^1^, M. Bourrelly^1^, M. Cissé^2^, B. Dembélé Keita^2^, B. Spire^1^, M. Fiorentino ^1^

^1^IRD, Aix Marseille Univ, Inserm, SESSTIM, Sciences Economiques & Sociales de la Santé & Traitement de l'Information Médicale, ISSPAM, Marseille, France, ^2^ARCAD Santé PLUS, Centre Intégré de Recherche, de Soins et d'Action Communautaire (CIRSAC), Bamako, Mali, ^3^ORS PACA, Observatoire régional de la santé Provence‐Alpes‐Côte d'Azur, Marseille, France

**Background**: In Mali, circular migration, sex work, geographic isolation and limited access to HIV care services and prevention information create a potential risk for HIV transmission in and from informal gold mining sites (IGMS). We aimed to compare vulnerabilities, mobility, behaviours and HIV transmission risk among men, female sex workers (FSWs) and non‐FSW‐women living with HIV at IGMS.

**Methods**: We used data from the ANRS‐SANU Gundo trial conducted in 2019−2022 at the Kofoulatiè and Diassa IGMS. Baseline characteristics of participants (newly HIV diagnosed), and ART adherence and HIV transmission risk 6 months after initiating ART were compared using Chi‐square tests.

**Results**: Of the 149 PLHIV, 34% were men, 26% were FSW and 40% were non‐FSW women. Men and non‐FSW‐women were more likely to report condomless sex with HIV‐negative/unknown‐status spouses at baseline (31%, 55% vs. 13% in FSW, *p* < 0.001) and to have no education (69%, 79% vs. 16%, *p* < 0.001). Non‐FSW‐women were more likely to have a precarious socio‐economic status (55% vs. 33% in men, 16% in FSW, *p* < 0.001) and to self‐perceive at risk of transmitting HIV (34% vs. 13%, 5%, *p* = 0.007). FSW reported 90 [60; 90] sexual partners in the past month (vs. 1 [0;1] in the other groups). They were more likely to be foreigners and non‐residents of IGMS (79% vs. 2% in men, 15% in non‐FSW women, *p* < 0.001), to report sex under alcohol or drugs (26% vs. 2%, 1%, *p* < 0.001), and condomless sex with HIV‐negative/unknown‐status occasional partners (18% vs. 3%, 2%, *p* = 0.004). Six months after initiating ART (*n* = 72), 36% of men and 30% of non‐FSW‐women were at risk of HIV transmission (i.e. uncontrolled viral load and condomless sex with any HIV‐negative/unknown‐status partner), compared to 18% of FSW (*p* = 0.05). ART adherence was similar across groups.

**Conclusions**: Our findings highlight the urgent need to strengthen and tailor HIV care and information campaigns in IGMS in Mali, not only for key populations such as FSW, where the risk of transmission may primarily stem from high mobility, multiple partners and substance use, but also for men and other women living with HIV, who are also at risk of transmission due to unprotected sex with their spouses and socio‐economic vulnerabilities.

### OAD0404 The perfect storm? Impact of climate change on HIV treatment access and adherence in Zimbabwe

R.W. Mukondwa ^1^, C. Christian^2^, K. Takarinda^1^, W. Mukuwapasi^3^, T. Makoni^3^, C. Aviles Guaman^2^, A. Ayer^2^, M. Hudson^2^, N. West^2^, K. Webb^1^, P. Shete^2^

^1^Organization for Public Health Interventions & Development, Programs, Harare, Zimbabwe, ^2^Center for Tuberculosis, University of California San Francisco, Health, California, United States, ^3^Zimbabwe National Network of People Living with HIV, SIE, Harare, Zimbabwe

**Background**: People living with HIV (PLHIV) face compounded challenges due to climate change, such as drought, extreme temperatures and water scarcity. These environmental stresses can interfere with HIV care adherence and exacerbate poor health outcomes. We explored the effects of climate change on HIV care and treatment adherence in 15 districts of Zimbabwe.

**Methods**: We conducted a mixed method study. Quantitative analyses utilized retrospective cross‐sectional data from client satisfaction surveys (CSS) among PLHIV (≥18 years) on antiretroviral therapy (ART) from November to December 2024. We summarized demographic characteristics, climate‐related disruptions and barriers to HIV care across 321 health facilities in 15 districts of Zimbabwe. We conducted three focus group discussions (FGDs) with recipients of HIV care. These were audio recorded, transcribed, translated into English, then uploaded to Dedoose for coding using deductive and inductive approaches.

**Results**: Among 896 PLHIV in ART care completing the CSS, 587 (66%) were female. Median age was 45 years (IQR: 36−52 years). Overall, 665 (74%) reported observed changes in climate in their communities over the past 5 years;75% (*n* = 497) of whom reported disruptions to HIV care due to climate change. Among those reporting disruptions to HIV care due to climate change, 432 (87%) reported adherence challenges related to lack of food, while 315 participants (63%) cited insufficient water access. Half of the participants identified transportation and financial barriers as critical issues affecting their ability to access HIV care;129 (26%) reported inability to safely store medication due to extreme temperatures. Qualitative findings revealed that climate change exacerbated pre‐existing vulnerabilities which impeded HIV care such as food insecurity, particularly, crop failures and livestock deaths, which disrupt household access to food and income required for accessing health facilities. Climate effects were cited as causes for taking HIV medications without adequate food or water, leading to side effects which decreased adherence.

**Conclusions**: Climate change creates and exacerbates a cascade of challenges, such as food insecurity and water shortages, that reduce adherence to care for PLHIV. These challenges require integrated climate adaptation and resilience strategies within routine HIV care programmes to improve health outcomes for PLHIV in climate vulnerable regions.

### OAD0405 Investigating the associations between weather, climate and HIV outcomes in sub‐Saharan Africa: a systematic review

M. Buczkowska ^1^, A. Trickey^2^, G. Charnley^3,4^, A. Gabot^1^, G. Hutchings^1^, C. Iwuji^5,6^, I. Kelman^1,7^

^1^University College London, London, United Kingdom, ^2^University of Bristol, Bristol, United Kingdom, ^3^Global Health Resilience Group, Barcelona Supercomputing Center ‐ Centro Nacional de Supercomputación, Barcelona, Spain, ^4^Imperial College London, London, United Kingdom, ^5^University of Sussex, Brighton, United Kingdom, ^6^Africa Health Research Institute, Durban, South Africa, ^7^University of Agder, Kristiansand, Norway

**Background**: The increasing frequency and intensity of extreme weather events in sub‐Saharan Africa pose unique challenges for public health. While the potential links between weather, climate and HIV outcomes have been hypothesized, evidence remains limited. This systematic review aimed to explore existing studies and identify gaps in understanding how weather and climate influence sexual behaviours, HIV transmission and HIV progression in sub‐Saharan Africa.

**Methods**: A systematic search of five databases was conducted to identify qualitative and quantitative studies examining weather and climate variables and HIV‐related measures in sub‐Saharan Africa. A narrative synthesis approach was used to summarize findings. Studies were assessed for quality, and descriptive trends were identified. A framework representing causal pathways between weather, climate and HIV outcomes was developed, based on the results from the qualitative studies.

**Results**: Twenty studies (14 quantitative, six qualitative) were included. Drought was the most frequently studied exposure and was associated with increased HIV prevalence, higher transactional sex rates and ART disruptions, especially in rural areas. Heavy rainfall and extreme heat were associated with food insecurity, migration and reduced healthcare access, further exacerbating HIV risks. Research gaps included limited longitudinal data, underrepresentation of children and key populations, and little focus on Western and Central Africa regions. Variability in weather and climate metrics and reliance on cross‐sectional designs limited the generalizability of findings.

**Conclusions**: Weather and climate might significantly impact HIV outcomes through indirect pathways such as food insecurity, migration and healthcare access. Vulnerable populations, including rural women and adolescents, face disproportionate risks. Future research should prioritize longitudinal studies, standardized metrics for weather and climate variables, and targeted investigations into underrepresented regions and populations. Integration of climate resilience strategies into HIV programmes, investment in data collection systems and policies addressing climate‐driven health vulnerabilities are critical.[Fig jia226518-fig-0021]

**Figure 1 jia226518-fig-0021:**
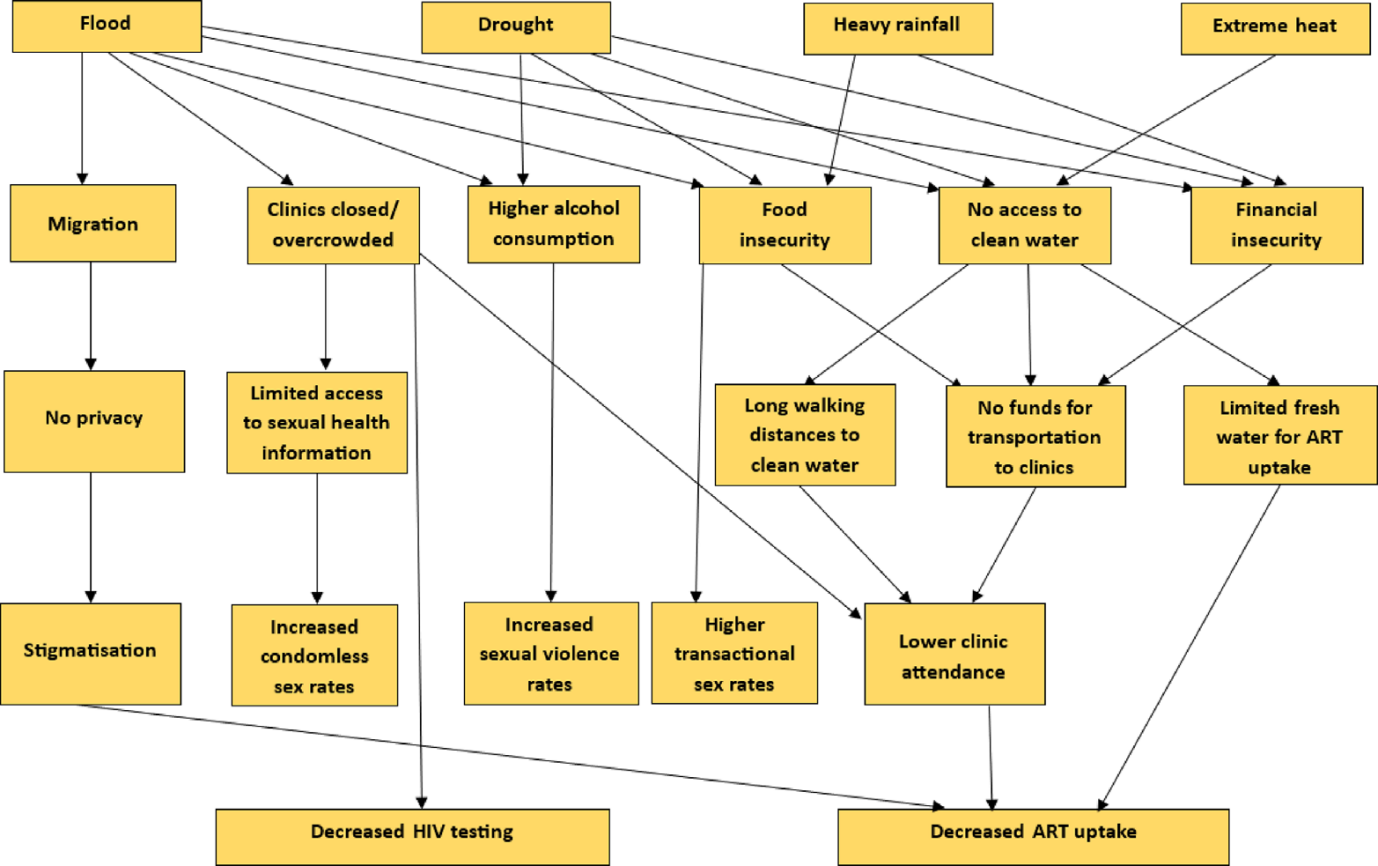
OAD0405

### OAD0502 HIV prevalence, risks and vulnerabilities of trans and gender diverse people in Saint Petersburg, Russia

M. Kandolsky ^1^

^1^Independent Researcher, Yerevan, Armenia

**Background**: Trans and gender diverse people (TGD) play a critical role in the context of the HIV epidemic, yet significant knowledge gaps persist. These gaps include limited regional diversity and a particular scarcity of data from Eastern Europe, with a predominant focus on trans feminine individuals, leaving data on trans masculine and other gender diverse people notably sparse. So far, no data on TGD living in Russia, country with the largest HIV epidemic in the region, has been available. This study represents the first exploration of Russian TGD individuals, focusing on Saint Petersburg, a city attracting TGD people from across the country.

**Methods**: We conducted a bio‐behavioural surveillance study using respondent‐driven sampling (*N* = 300). Behavioural data was collected through face‐to‐face structured interviews, and capillary blood HIV tests were conducted following WHO protocols.

**Results**: Transgender men (45.9%) and non‐binary individuals (32.4%) predominate within the group, while transgender women constitute only 14.6%, yet bear the entirety of HIV cases (HIV prevalence 14.1%) (95% CI: 0%−28.3%). Social transition is common, with 45.8% undergoing hormonal therapy. However, this poses health risks, as 60.0% lack medical supervision, and 45.6% use non‐pharmacy drugs. Overall, the study indicates low prevalence of risk behaviours among TGD individuals: needle sharing during hormonal injections is rare (0.6%), so as injection drug use (0.9%) and sexual activity (39.6% reported sex in the last 6 months). Yet, coverage by HIV prevention (11.5%) and testing (61.8% ever tested) services remains insufficient. Despite often concealing their identity, TGD individuals encounter various forms of discrimination and violence, with a significant proportion exhibiting signs of depression (63.5%). The newly enacted law prohibiting gender transitions had a significant negative impact on the TGD lives: 65.1% reported deterioration in their psychological wellbeing, 22.4% accelerated gender transition, 39.3% began to seriously consider emigration.

**Conclusions**: While the overall HIV prevalence among TGD individuals in Saint Petersburg does not suggest a concentrated epidemic (1.9%), transgender women remain at heightened risk of HIV acquisition. Inadequate coverage by HIV prevention and testing services coupled with negative social changes in the country services underscores the urgent need for targeted interventions addressing the unique vulnerabilities of this population.

### OAD0503 PrEP experiences and challenges among transgender women in the United States and Puerto Rico in the era of PrEP choice: findings from the ENCORE cohort

E. Cooney ^1^, T. Poteat^2^, A. Radix^3,4^, C. Beyrer^5^, J. Schneider^6^, M. Miller^7^, V. Vannappagari^8^, L. Ragone^8^, A. Guignard^9^, A. de Ruiter^10^, S. Reisner^11^, A. Wirtz^1^, ENCORE Study Group

^1^Johns Hopkins Bloomberg School of Public Health, Baltimore, United States, ^2^Duke University School of Nursing, Durham, United States, ^3^Callen‐Lorde Community Health Center, New York City, United States, ^4^Columbia University Mailman School of Public Health, Department of Epidemiology, New York City, United States, ^5^Duke Global Health Institute, Durham, United States, ^6^Emory University School of Medicine, Atlanta, United States, ^7^Trans Solutions Inc., Indianapolis, United States, ^8^ViiV Healthcare, Durham, United States, ^9^ViiV Healthcare, Wavre, Belgium, ^10^ViiV Healthcare, London, United Kingdom, ^11^University of Michigan School of Public Health, Ann Arbor, United States

**Background**: Real‐world data on PrEP choice and experience following the approval of long‐acting PrEP are limited. We sought to characterize the experiences of transgender women using PrEP in a large nationwide cohort.

**Methods**: From March 2023 to September 2024, we enrolled English‐ and Spanish‐speaking adult transgender women without HIV (laboratory‐confirmed) into the ENCORE cohort. Data were collected via self‐reported baseline surveys. Descriptive statistics characterized self‐rated PrEP experience (0−100 scale with 100 indicating completely positive experience), challenges taking PrEP and reasons for PrEP method selection. We assessed for differences by race, ethnicity, age, insurance, income and geographic region.

**Results**: We enrolled 2506 participants, 591 of whom had ever used PrEP (24%). At baseline, 439 used PrEP in the prior 6 months. Of these, 415 (95%) used daily oral, 15 (3%) received long‐acting injections (LAI) and 9 (2%) used both. Participants rated their PrEP experiences positively: mean (standard deviation): 87 (22); median (IQR): 100 (82−100). Ratings did not significantly differ by PrEP modality or socio‐demographic characteristics. Commonly reported pill challenges were: difficulty remembering daily dose (28%), side effects (25%) and assumptions about sexual behaviour from others (22%). Reported LAI challenges were: injection site pain and/or side effects (38%), required clinical visits (33%) and assumptions about sexual behaviour from others (21%). Black and Latina participants were more likely than non‐Hispanic White participants to report stigmatizing experiences related to their PrEP use including assumptions about their HIV status and number of partners and disapproval from partners. Top reasons for choosing oral over LAI were: using pills before LAI FDA‐approval (35%), healthcare provider recommendation (32%) and unaware of LAI (31%). Top reasons for choosing LAI over pills were: dosing frequency (79%), dislike pills (38%) and healthcare provider recommendation (21%). Black participants were more likely to cite concern that the effectiveness of long‐acting would decrease over time as a reason for choosing oral PrEP. Latina participants were more likely to cite greater confidence in pills as a reason for choosing oral PrEP.

**Conclusions**: Transgender women generally report positive PrEP experiences, but motivations and challenges may differ by race and ethnicity. Shared decision‐making interventions may support effective PrEP use.

### OAD0504 Stigma leads to substance use among MSM and transgender women living with HIV: insights from Vietnam

N.T. Minh ^1,2^, D. J. Colby^1^, A. Bao^1^, L.A. Do^3^, T.C. Le^1^, H.T. Tran^1^, B.Q. Luong^4^, P. Doan Thi Kim^5^, K. Do Quang^6^

^1^Center for Applied Research on Men and Community Health (CARMAH), Ho Chi Minh, Viet Nam, ^2^University of Medicine and Pharmacy at Ho Chi Minh City, Epidemiology, District 5, Viet Nam, ^3^Pham Ngoc Thach University of Medicine, Public Health, Ho Chi Minh, Viet Nam, ^4^HCMC Center for Disease Control, Epidemiology, Ho Chi Minh, Viet Nam, ^5^Can Tho Center for Disease Control, Epidemiology, Can Tho, Viet Nam, ^6^Galant Clinic, Ho Chi Minh, Viet Nam

**Background**: Previous research on stigma as a driver of substance use among men who have sex with men (MSM) and transgender women (TW) living with HIV has been limited in establishing causality. We aimed to evaluate the association between stigma experiences and the subsequent initiation of opiates and amphetamine‐type stimulants (methamphetamine, heroin and ecstasy) in a prospective cohort of MSM and TW with HIV in Vietnam.

**Methods**: A community‐based study was conducted in the Mekong Delta, Vietnam (2019–2022), involving MSM and TW with HIV who had no prior substance use. Five stigma dimensions—mockery, negative judgement, physical attack, sense of exclusion and emotional/mental trauma—were assessed at baseline, and substance use initiation was evaluated after a follow‐up period (median: 394 days). Univariable and multivariable Poisson models were used to estimate prevalence differences (PD), attributable risk (AR) and 95% confidence intervals (CIs).

**Results**: Among 504 participants, 76.0% were MSM and 24.0% were TW. By the end of the follow‐up, 30.1% had initiated substance use (21.8% methamphetamine, 16.5% ecstasy and 0.8% heroin). Overall, 66.8% of participants reported experiencing at least one form of stigma (57.7% sense of exclusion, 49.4% emotional/mental trauma, 47.4% mockery, 45.8% physical attack and 22.8% negative judgement). Physical attacks, sense of exclusion and emotional/mental trauma were significantly associated with an increased risk of substance use. Among MSM, stigma led to 14 new substance users per 100 annually (PD = 0.14 [95% CI: 0.08–0.19]; AR = 36.7% [24.8–47.1]). Among TW, it resulted in 18 new users per 100 annually (PD = 0.18 [0.07–0.30]; AR = 37.9% [16.0–54.1]).

**Conclusions**: Stigma is a critical social determinant of health. Beyond its psychosocial toll, stigma drives harmful risk behaviours that undermine treatment adherence, exacerbate HIV transmission risks and amplify health inequities among MSM and TW, necessitating the integration of stigma‐reduction strategies into clinical practice and public health policy.[Fig jia226518-fig-0022]

**Figure 1 jia226518-fig-0022:**
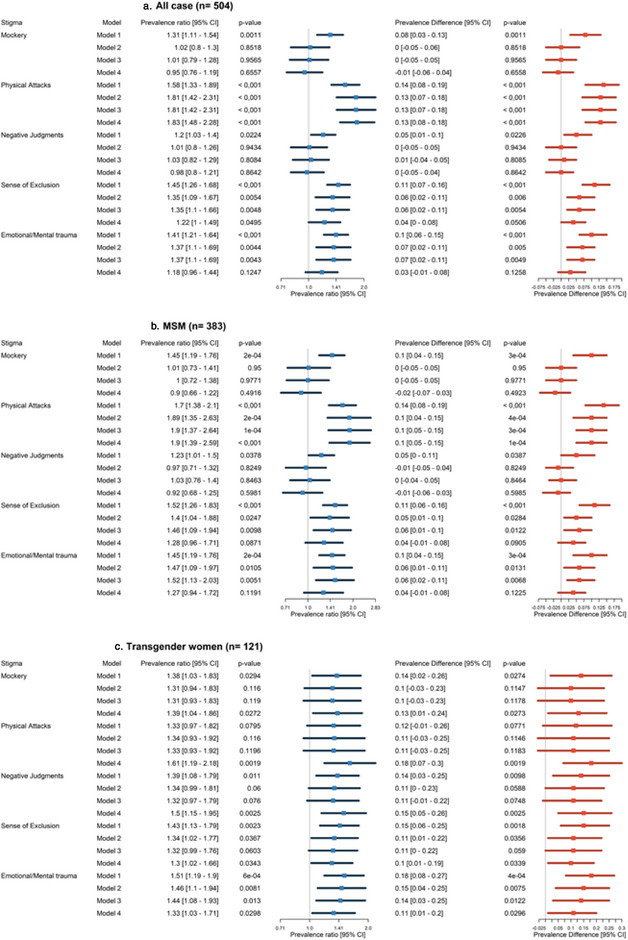
**OAD0504: The effect of stigma on the risk of substance use among MSM** (a) and Transgender Women (b) with HIV in Vietnam. Model 1: Univariable analysis; Model 2: Adjusted for age, education level, and the number of sexual partners in the past 12 months as potential confounders.

### OAD0505 Exposure to violence among transgender women living with HIV in France, engaged or not in sex work: results from the national ANRS‐14056 TRANS&VIH study

M. Fiorentino ^1^, A. Faye^1^, M. Mora^1^, R. Van Huizen^1^, M. Bourrelly^1^, G. Maradan^2^, C. Berenger^2^, F. Michard^3^, Y. Yazdanpanah^3^, A.F. Maresca^4,5^, E. Rouveix^4^, L. Balhan^6,7^, M. Costa^6,7^, D. Michels^7^, L. Blanquart^8^, G. Rincon^8^, B. Spire^1^, M. Annequin^1^, the ANRS‐Trans&HIV study group

^1^IRD, Aix Marseille Univ, Inserm, SESSTIM, Sciences Economiques & Sociales de la Santé & Traitement de l'Information Médicale, ISSPAM, Marseille, France, ^2^ORS PACA, Observatoire régional de la santé Provence‐Alpes‐Côte d'Azur, Marseille, France, ^3^Service de Maladies Infectieuses, Hôpital Bichat ‐ Claude‐Bernard, APHP, Paris, France, ^4^Service de Médecine Interne, UFR Paris Île‐de‐France Ouest, Hôpital Ambroise‐Pare, Boulogne‐Billancourt, France, ^5^Service de Maladies Infectieuses et Tropicales, Hôpital Avicenne, APHP, Bobigny, France, ^6^AIDES, Pantin, France, ^7^Community‐Based Research Laboratory, Coalition PLUS, Pantin, France, ^8^ACCEPTESS‐T, Paris, France

**Background**: Worldwide, stigma and violence, experienced by transgender women (TGW) and by sex workers (SWs), negatively impacting health and access to HIV care. This is the first national‐level study investigating typologies of recent violence experienced by TGW living with HIV (TGWHIV), engaged or not in sex work, in France.

**Methods**: The ANRS‐TRANS&HIV study was a national, cross‐sectional, retrospective survey conducted in 2020−2022 among 536 TGWHIV recruited from 36 HIV care centres. Typologies of recent violence exposure were identified using latent class analysis based on 24 violence‐related variables (by type and perpetrator), separately for TGWHIV‐SW and non‐sex workers (TGWHIV‐nSW). We used multinomial logistic regression to assess factors associated with TGWHIV‐SW typologies.

**Results**: Among the 501 participants with complete violence data, five violence typologies emerged: in TGWHIV‐SW, “clients/street violence” (17%), “multiple perpetrators violence” (involving clients, passersby, police, colleagues, partners, family and friends) (8%) and “low violence” (42%); in TGWHIV‐nSW, “multiple perpetrators violence” (3%) and “no violence” (30%). In TGWHIV‐SW, “clients/street violence” was associated with antiretroviral treatment (ART) interruption for ≥2 days in the previous month (aOR: 1.9 95% CI [1.0–3.4]) and stimulant consumption (2.3 [1.2–4.3]), compared to “low violence.” “Multiple perpetrators violence” was associated with a lack of moral support (2.9 [1.3–6.7]) and depression (4.2 [1.9–9.2]). Both typologies of violence were associated with inadequate housing conditions (2.3 [1.2–4.4]; 2.0 [0.9–4.5]), daily cannabis use (4.4 [1.8–10.9]; 3.1 [0.9–10.7]) and engaging in transactional sex in public or outdoor locations (4.0 [1.9–8.8]; 4.2 [1.4–12.8]).

**Conclusions**: Whether or not involved in sex work, TGWHIV face multidimensional violence in France. Among TGWHIV‐SW, this appears to be exacerbated by precarious housing and sex work conditions, with varying health implications. These include ART interruptions among those exposed to street violence in the context of sex work, and mental health issues among those subjected to violence by multiple perpetrators, including from their close social circle and the police. In a context of structural transphobia and sex work criminalization, ensuring equitable access to HIV care and mental health services for TGW is critical and inseparable from protecting their fundamental human rights and improving their socio‐economic conditions.

### OAD0602 AI‐powered preventive intervention for stigma and suicidal ideation in HIV self‐management: development, evaluation and user testing of the MARVIN chatbot's integrated mental health management module

S. Villanueva ^1,2^, Y. Ma^1,2^, S. Achiche^1^, A. Cadri^2^, B. Lebouché^2^

^1^Polytechnique Montreal, Biomedical Engineering, Montreal, Canada, ^2^Research Institute of McGill's University Health Center, Infectious Diseases and Immunity in Global Health (IDIGH) and Centre for Outcomes Research & Evaluation, Montreal, Canada

**Background**: Stigma and mental health challenges like anxiety and depression which are major concerns for people with HIV (PWH) can sometimes lead to self‐harm and suicidal ideation. Our AI‐powered MARVIN chatbot supports HIV self‐management by providing knowledge, assisting with medication adherence. To address extreme user intentions effectively, we developed and incorporated a preventive intervention module.

**Methods**: (1) MARVIN/ChatGPT Hybrid Module: Using the CO‐STAR framework (i.e. providing Context, Objective, Style, Tone, Audience and Response format), we prompt‐tuned ChatGPT to classify message intent into self‐harm, insult or non‐extreme (i.e. any other intent). We tested performance with three public hate speech datasets (hatespeechdata.com), MARVIN‐user conversations and a synthetic dataset (*N* = 1000/class), evaluating precision, recall, F1 score and overall accuracy. The algorithm was integrated into MARVIN. (2) Multidisciplinary Validation: Six testers (three Canadian PWH, a Chinese engineer, an Algerian engineer and a French doctor; all male, based in Canada) tested MARVIN for 2 hours through 14 conversational scenarios without sharing sensitive data and completed a two‐item questionnaire on clarity and satisfaction.

**Results**: With one‐shot prompting, the hybrid module attained 97.00% and 94.90% for recall on self‐harm and insult intent, respectively. Overall accuracy reached 95.57%, with the remaining metrics shown in the figure below. The MARVIN‐ChatGPT hybrid module successfully generated appropriate responses containing (1) emergency contact information for self‐harm intents; (2) messages guiding users to use stigma‐free expressions for insult messages; and (3) a response reviewed by a medical expert for a non‐extreme intent. All six male testers found MARVIN's responses to be clear and concise and were satisfied with the overall experience. However, one PWH participant suggested including links to additional resources.

**Conclusions**: Testing this anti‐stigma/suicidal ideation module within MARVIN demonstrated its ability to detect extreme intents and deliver concise responses, highlighting AI‐driven chatbots’ potential in fostering virtual stigma‐free self‐management and mental health support for PWH.[Fig jia226518-fig-0023]

**Figure 1 jia226518-fig-0023:**
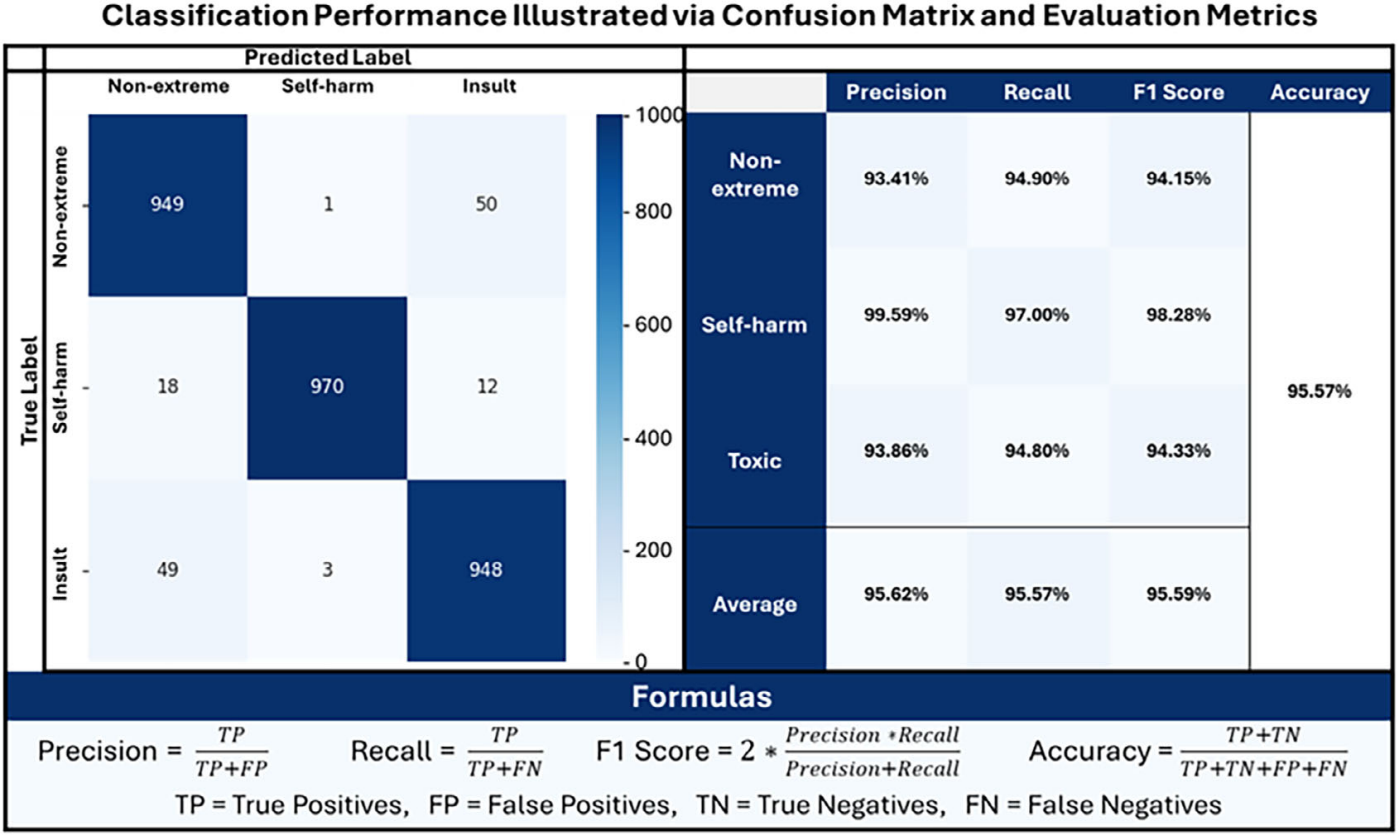
OAD0602

### OAD0603 “Superbees Awakened”—development of a digital game to enhance understanding of germline‐targeting vaccine trials among communities and site staff in South Africa and India

J. Mukherjee ^1^, S. ul Hadi^1^, P. Saha^1^, K. Goyal^1^, A. Baveja^2^, F. Gonsalves^2^, P. Sandbhor^3^, T. Aurora^2^, E. Landais^4^, H. Makkan^5^, S. Rawat^6^, Y.W. Machira^7^, K. Ondeng'e^7^, Z. Ciko^8^, A. Chauhan^2^

^1^International AIDS Vaccine Initiative Inc, Gurugram, India, ^2^Quicksand Design Studio, New Delhi, India, ^3^University of York, York, United Kingdom, ^4^International AIDS Vaccine Initiative, Neutralizing Antibody Centre, San Diego, United States, ^5^The Aurum Institute, Johannesburg, South Africa, ^6^The Humsafar Trust, Mumbai, India, ^7^International AIDS Vaccine Initiative Inc, Nairobi, Kenya, ^8^International AIDS Vaccine Initiative Inc, Cape Town, South Africa

**Background**: With advances in rational HIV vaccine design, discovery medicine vaccine trials (DMVTs) involve testing of complex scientific hypothesis, require intensive clinic visits and often include invasive sampling. Thus, it becomes critical for trial participants, participating communities and site staff to understand the scientific rationale behind these trials to enhance awareness, motivation and trust. To address this unmet need, IAVI partnered with Quicksand to develop digital games that explain germline targeting concepts using behavioural science, gamification and interactive digital design.

**Description**: We conducted 10 co‐creation workshops with HIV vaccine scientists (*n* = 5), clinical trial site staff (*n* = 10) and community representatives (*n* = 10) to (a) understand information needs; (b) identify metaphors; (c) develop sequential story line; (d) devise game play. Through immersive engagement and iterative prototyping, a 4‐module digital game “Superbees Awakened” was developed to (1) elaborate the human immune system and role of a research lab (Exploring the “Weza Forest” and “Forest Health Centre”); (2) explain non‐conventional sampling like large blood draws, leukapheresis and fine needle aspirations (Scan the large, filtered and focused samples); (3) identify conserved HIV epitopes and design whole protein or mRNA delivered immunogen (Spot the “Common Point”); and (4) explain B‐cell maturation, prime‐boost vaccine schedules and germline targeting (Train and target). Post‐game discussions enabled players to link game metaphors with HIV science.

**Lessons learned**:

- When scientific information is conveyed to communities through relatable stories and culturally relevant metaphors, it **facilitates self‐driven exploration of scientific concepts** leveraging indigenous knowledge as opposed to prescriptive top‐down information sharing.
- The self‐discovery of science is less intimidating and **leads to greater self‐confidence and higher retention of information**.
- The metaphors need to be carefully identified and vetted by scientific experts to avoid miscommunication, thus **balancing scientific accuracy and clarity of content**.
- Digital modules should **bring together variety of game types** (puzzles, action, adventure, skill‐based) rather than a singular experience to cater different learning styles.

**Conclusions/Next steps**: Digital gamification represents an impactful strategy for simpler, clearer and relatable science communication relevant to communities. Given the broad reach and high familiarity of digital technologies among youth, this game serves as a complementary tool to routine information, education and communication methodologies.

### OAD0604 Efficacy of a clinic‐integrated HIV prevention app “JomPrEP” among men who have sex with men in Malaysia: preliminary findings of a randomized control trial

S.H. Sujan ^1^, J.A. Wickersham^2^, E.E. Hui^3^, K. Paudel^1^, K. Gautam^1^, I. Azwa^3^, P. Phiphatkhunarnon^4^, R. Shrestha^1^

^1^University of Connecticut, Storrs, United States, ^2^Department of Medicine, Yale School of Medicine, New Haven, United States, ^3^Faculty of Medicine, University of Malaya, Kuala Lumpur, Malaysia, ^4^Love Foundation, Chiang Mai, Thailand

**Background**: Men who have sex with men (MSM) are disproportionately affected by HIV. In Malaysia, where MSM face high levels of stigma and discrimination, including in healthcare settings, mobile health platforms offer promising opportunities for advancing HIV prevention. Thus, to scale up HIV prevention endeavours among this vulnerable group, we created a clinic‐integrated HIV prevention app called “JomPrEP” which provides a virtual platform for them to engage in HIV prevention services. In collaboration with the local clinics in Malaysia, JomPrEP offers a number of HIV prevention (i.e. HIV testing and pre‐exposure prophylaxis [PrEP]) and other support services without having to interface with clinicians. This study aimed to measure the efficacy of the JomPrEP app to increase HIV testing and PrEP uptake among MSM in Malaysia.

**Methods**: We conducted a randomized controlled trial on 268 MSM (intervention group = 134, control group = 134) in Malaysia. Participants were recruited online from November 2023 to September 2024. Participants were randomly assigned to either access the JomPrEP app or receive standard healthcare services. Measures like demographics, HIV and STI testing history, PrEP uptake, sexual behaviour, drug and alcohol use, stigma, and depression were evaluated at baseline and 3‐, 6‐, and 9‐month periods.

**Results**: Participant's mean age was 28.8 (5.7) years. Overall, the intervention group showed a 26.8% increase in HIV testing and 5.9% increase in PrEP uptake compared to control group. Of the intervention participants, 43.3% initiated PrEP, with 36.6% opting for on‐demand PrEP and 6.0% choosing traditional PrEP. Interestingly, HIV testing had significantly increased over time in both groups (*F* (2,711) = 81.38, *p* < 0.001). Participants at 3 (OR = 16.83, 95% CI, 8.85–32.02) and 6 months (OR = 7.90, 95% CI, 4.13–15.12) showed higher odds of HIV testing than baseline. In addition, compared to the control group, participants in the intervention group had significantly higher odds of PrEP uptake at 3 months (OR = 2.32, 95% CI 1.00–5.37) and 6 months (OR = 2.99, 95% CI 1.26–7.13).

**Conclusions**: Our study demonstrated that the JomPrEP app is an effective tool for increasing HIV testing and PrEP uptake among MSM in Malaysia as compared to the standard of care.

### OAD0605 TWIIN: a digital assistant for enhancing health access among key populations

M. Malakhova ^1^, O. Marouniak^1^

^1^Alliance for Public Health, Kyiv, Ukraine

**Background**: TWIIN is an AI‐powered digital assistant designed to address barriers faced by key populations, such as people who use drugs (PWUD) and men who have sex with men (MSM). It provides 24/7, anonymous support for HIV prevention, mental health and harm reduction, aiming to reduce stigma and improve service access.

**Description**: Piloted in Ukraine, TWIIN integrates artificial intelligence with culturally relevant digital humans to provide tailored information and connect users to essential services. Focus areas include HIV prevention, harm reduction, sexual health and mental health. During the pilot, over 5000 unique users generated nearly 39,000 sessions. To maximize outreach, TWIIN utilized social media campaigns, NGO partnerships and collaborations with community representatives. Anonymity and accessibility were prioritized, especially in wartime conditions.1, 2

**Figure 1:**
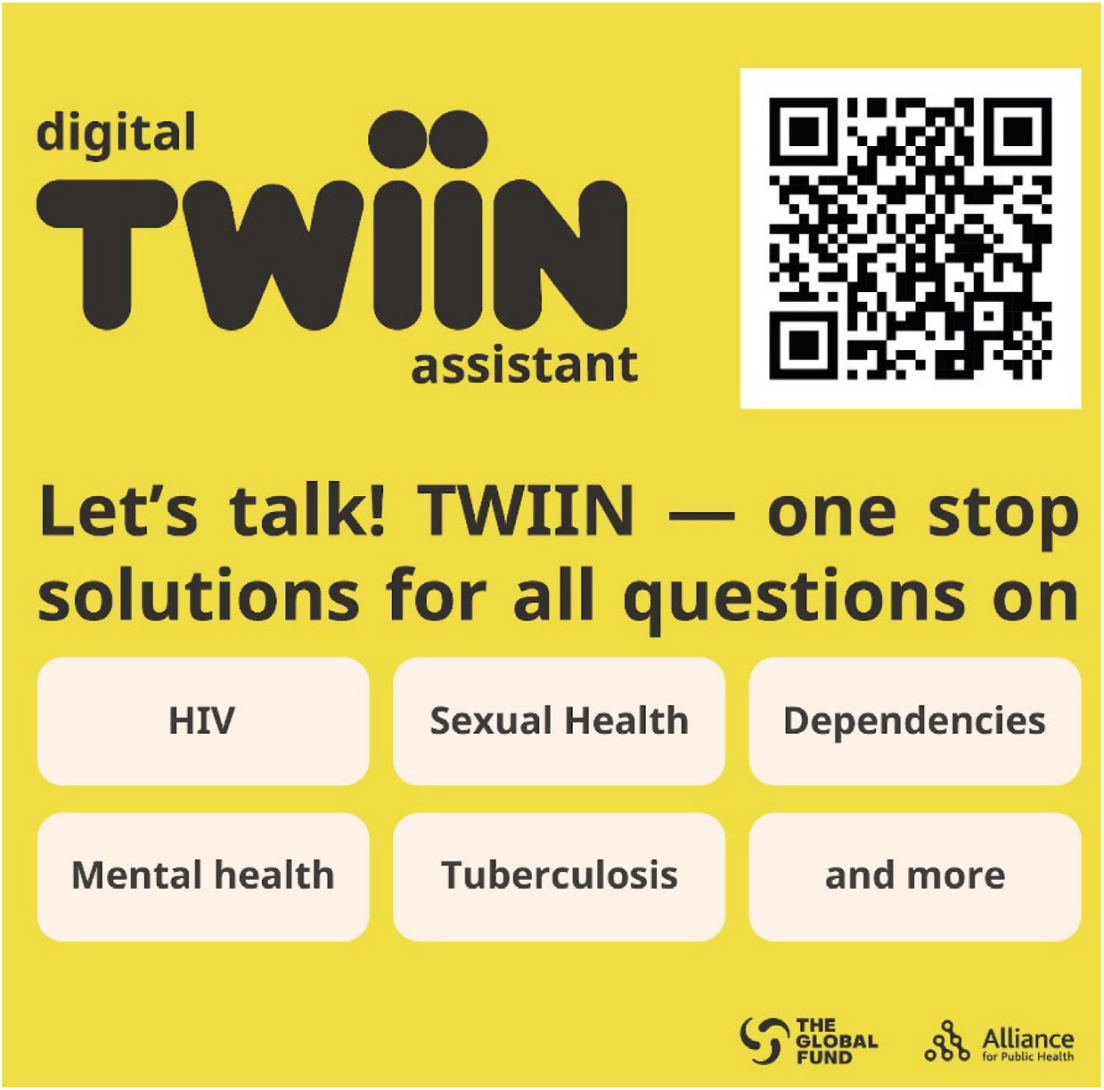
OAD0605

**Figure 2:**
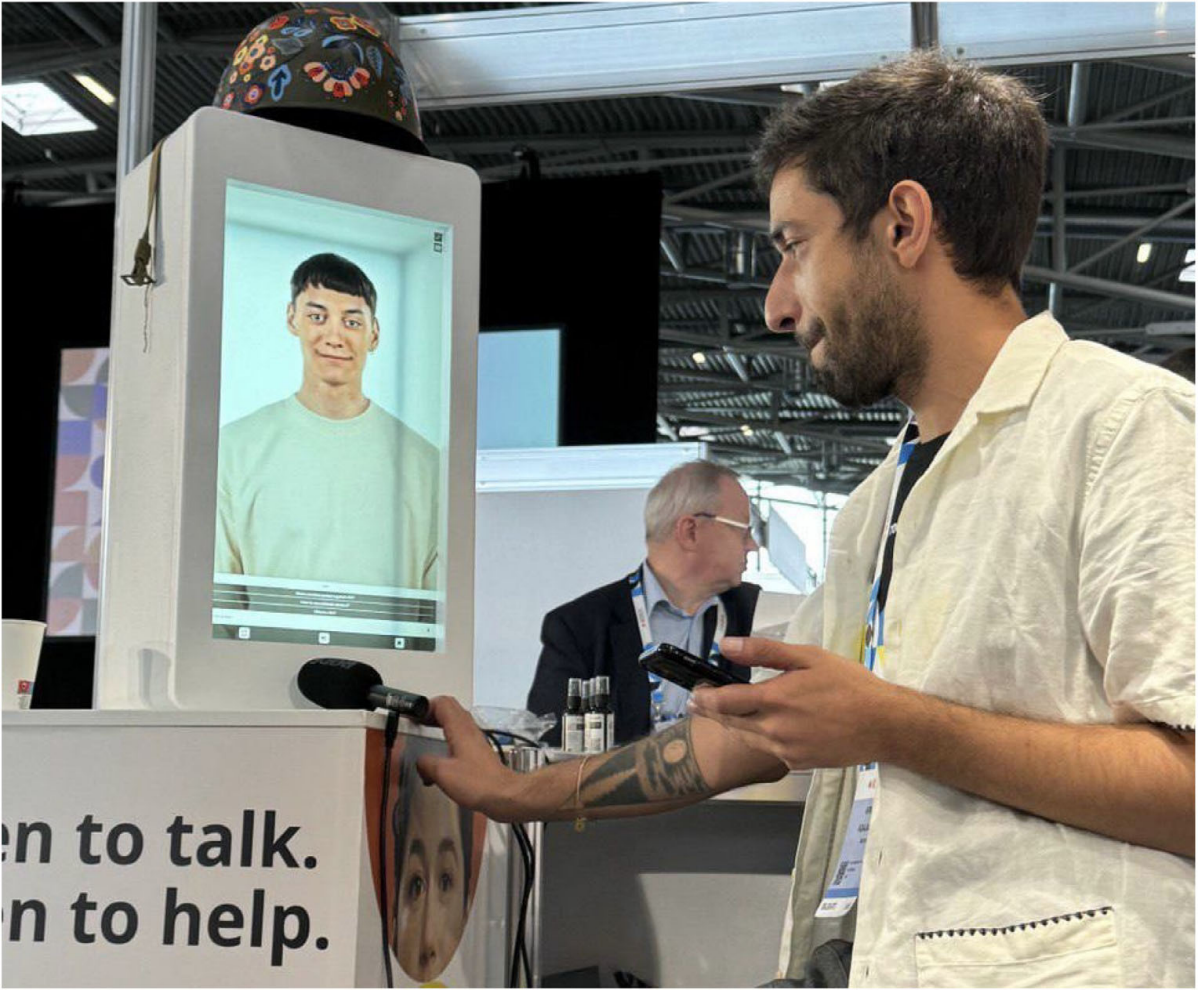
OAD0605

**Lessons learned**: Feedback showed high satisfaction, with 85% rating TWIIN as 4‐5/5 and 87% recommending it. Piloted targeting PWUD and MSM, we observed user experience from various perspectives. Onboarding over 400 doctors and 70 NGOs supported integration into existing services. Challenges included response delays and contacts presentation, which are being addressed through system improvements. The pilot emphasized the importance of cultural relevance, user anonymity and iterative feedback to refine the platform.

**Conclusions/Next steps**: TWIIN has shown great promise in expanding health access for marginalized populations, particularly in challenging settings. Plans include enhancing response times and developing additional modules. In 2025, TWIIN will launch in Moldova and Georgia, with localized adaptations to meet country needs. This scalable, digital health innovation offers a replicable model for addressing HIV prevention, mental health and harm reduction globally.

### OAE0102 Improving the health and human rights of PLHV and key populations in Myanmar: Community Feedback Mechanism project by Puu Paung Swann Saung Myanmar (2021−2024)

K.K. Win ^1^, E.T. San^2^, K. Taung^3^

^1^Puu Paung wann Saung Myanmar / MINA, Central Steering Committee Member, Yangon, Myanmar, ^2^Puu Paung wann Saung Myanmar / MINA, Project Officer, Yangon, Myanmar, ^3^Myanmar Interfaith Network on AIDS_MINA, Secretary, Yangon, Myanmar

**Background**: Myanmar established Community Feedback Mechanism (CFM) in 2014 by the leadership of Community Network Consortium on HIV which consists of (8) HIV National Networks. The CFM focused on issues related to PLHIV, KP and implemented through networks collaborating with target groups in townships. The PPSSM is one of the networks implementing CFM in six townships since 2021.

**Description**: CFM collects feedback related to health services, law and justice issues, workplace and socially related issues. The feedback was gathered through MyRights Application and then directed to relevant townships for further action. At the local level, local CFM committees, with the guidance and support of National AIDS Program (NAP), oversee the activities, verify the cases and decide how to proceed with concerns. Supports such as legal assistance, emergency support and counselling are provided as necessary to resolve these issues.

**Lessons learned**: Since 2021, a total of (449) cases were received via MyRights Application. The Key Population classification on the total cases of (*n* = 449), the cases were classified as 369 (82%) were PLHIV, 57 (13%) were FSW, 22 (5%) were MSM and 1 (0.22%) were PWID. Of which, 10 cases (2%) were invalided, and 439 cases (98%) were approved. Among the approved cases of (*n* = 439), 358 cases (82%) were resolved, and the rest were unresolved. The cases were identified as follows: Healthcare related—30 cases (7%), Law and Justice Related—20 (5%), Socially Related—358 (82%) and Workplace Related—31 cases (7%). The resolved cases were 358 (80%), unable to resolved case were 79 (18%) and 2 (0.4%) of cases were in process of case handling by CFM. During this period, a total of 57 (13%) cases were provided, 55 (12.5%) for Nutrition Support and 2 (0.5%) for Referral Transportation Allowance to health facility sites as the emergency support of CFM by PPSSM.

**Conclusions/Next steps**: Since its inception, the project has proven to be the most successful community‐led system which significantly impacted the development of successful communication strategies and monitoring of HIV‐related services. Many users have provided positive feedback and praised the service for its accessibility and the quality of information and guidance offered.

### OAE0103 Leaving no one behind: implementation of informal female sex workers intervention for fast‐tracking HIV case detection in Andhra Pradesh: a sustainable, scalable programme experience from India

J. Williams^1^, A. Vijayaraman ^1^, P. Venkateswara Rao^1^, B. Aparna^1^, K. Priya^1^, R. Shanmuganathan^1^, Y. Kameswara Prasad^2^

^1^The Voluntary Health Services, Chennai, India, ^2^Andhra Pradesh State AIDS Control Society, Andhra Pradesh, India

**Background**: In the rapidly changing sex work milieu, there has been a major shift in the traditional forms of soliciting sex by female sex workers resulting in emergence of more discreet spaces, working under network operators (NWOs) and are referred as informal female sex workers (iFSWs). Based on our implementation experience, the iFSWs are part‐time sex workers and are most often engaged as home makers or does petty work. The fact that these iFSWs are not covered by HIV helps them in seeking confidentiality and secrecy, but poses multiple risks and vulnerabilities, including HIV.

**Description**: We conducted community listening exercise that helped discover the characteristics and needs of iFSWs and the role of NWOs in reaching iFSWs. A comprehensive package of services were offered ranging from HIV/STI education, testing and treatment, condom distribution, TB screening and treatment, NCD services, mental health counselling, addressing violence faced by iFSWs, linkages with government departments for social protection, and so on. The primary goal is to improve the health and wellbeing of iFSWs and aid in achieving HIV epidemic control in the Andhra Pradesh state.

**Lessons learned**: During May 2022−September 2024, 4039 iFSWs were reached and all of them were provided HIV education. HIV testing was done for 66.94% (2704), of which 10.1% (274) were detected positive and 94.5% (259) were initiated on ART. The mean age of iFSWs is 29.3 (± 5.05) years, 79.6% (3216) of the iFSWs possess 1−2 years of experience in sex work. Around 93% (3768) were married and majority of them 87.2% (3521) were living with their spouse. Most of the iFSWs (93.9% [3795]) solicit sex through NWOs. Around 20.6% (833) migrate for sex work and majority of them (53.7%, 282) migrate for a week's time.

**Conclusions/Next steps**: Given the secretive nature of sex work and being outside of the radar of routine HIV interventions, the importance of implementing programmes targeting iFSWs through NWOs cannot be over‐emphasized. The iFSW intervention strategy to reach the unreached KPs at early detection of HIV can be sustainable and scalable by integrating it with the existing targeted intervention.

### OAE0104 Survival is not enough: enhancing mental health integration in key population HIV focused programming in Kalangala islands post Uganda's Anti Homosexuality Act 2023

E. Jjemba ^1^, H.D. Kayemba^2^, S. Kobugabe^1^, S.P. Ssonko^3^, C. Koote^4^

^1^Action for integrated for Sustainable Development (AISDA), Health, Kampala, Uganda, ^2^Action for integrated for Sustainable Development (AISDA), Prevention, Kampala, Uganda, ^3^Smart Children Africa, Monitoring and Evaluation, Kampala, Uganda, ^4^Action for integrated for Sustainable Development (AISDA), Care and Treatment, Kampala, Uganda

**Background**: The enactment of Uganda's Anti Homosexuality Act (AHA) in 2023 intensified stigma and discrimination among key populations (KPs) especially gay and other men who have sex with men and transgender. Although efforts in HIV treatment have improved survival rates, many KPs in Kalangala islands have continuously experienced profound mental health challenges including anxiety, depression, suicidal thoughts which undermine ART adherence and treatment outcomes. In response to this outcry, Action for Integrated Sustainable Development (AISDA) through its KP programme embarked on integrating mental health support to KPs to enhance ART adherence and mental wellbeing by addressing stigma and discrimination in the aftermath of AHA. This followed attendance of one of its staff at AIDS 2024 and gained insights on integrating mental health in KP programming.

**Description**: A self‐reporting questionnaire with 20 questions was designed and administered to KPs by trained social workers during home visits and clinic days to screen for mental health issues. Social workers assessed responses to specific questions to identify immediate risks. Scores on suicidal thoughts triggered immediate interventions employing safety planning approaches including referrals for specialized care. Recognizing geographical isolation of islands, tele‐mental health support was integrated through follow‐up calls and virtual counselling sessions. Regular home visits were conducted by social workers to provide holistic support to KPs including monthly intensive adherence counselling to non‐suppressing KPs, post disclosure counselling and psychosocial support.

**Lessons learned**: Mental health integration improves ART adherence and viral load suppression; adherence improved from 75% to 92% and viral load suppression increased by 15% since August−December 2024 among 288 KPs.

Tele‐mental health support enhances access to care in island settings. Eighty‐five percent of clients flagged for follow‐up utilized virtual sessions with 80% reporting satisfaction and reduced symptoms after engagement.

Peer‐led support groups create safe spaces and reduce stigma; self‐reported cases of discrimination reduced by 40% and participants expressed increased confidence in accessing care.

Involvement of community leaders in the project fostered acceptance in communities.

**Conclusions/Next steps**: Integration of mental health support for KPs post AHA 2023 has fostered purposeful living, enhanced mental wellbeing significantly improving HIV treatment outcomes, underscoring the need for holistic stigma‐free care.

### OAE0105 Making HIV care affordable: cost‐effectiveness of interventions for refugee women and girls in Nakivale refugee settlement, Southwestern Uganda

N. Zahara^1^, U. Muhumuza ^2^

^1^International University of Africa, Medicine and Surgery, Khartoum, Sudan, the, ^2^Mbarara University of Science and Technology, Medicine and Surgery, Mbarara, Uganda

**Background**: Refugee women in Nakivale face disproportionate risk of acquiring HIV due to the combination of social and structural challenges. High levels of poverty, stigma, limited access to healthcare services exacerbate their vulnerability. Despite the global progress in HIV prevention and care, interventions in refugee contexts fail to address unique needs of these populations, regarding affordability and accessibility. Resource‐constrained settings like Uganda lack evidence on cost‐effectiveness of localized strategies, hindering informed decision‐making and sustainable programme design. This research addresses gaps by evaluating costs, affordability and effectiveness of tailored HIV interventions in Nakivale, providing critical insights to guide resource allocation, policy formulation.

**Methods**: Cost‐effectiveness analysis was conducted between January and June 2024, focusing on three interventions: mobile HIV testing counselling services, women's economic empowerment programmes and community‐led stigma reduction campaigns. Cost data were collected from healthcare providers, implementing partners, local organizations, while effectiveness measures included increased HIV testing uptake, improved linkage to care and reductions in new acquisitions. Data were analysed using incremental cost‐effectiveness ratio (ICER) framework in Uganda Shillings (UGX) from community and provider perspectives. Affordability was assessed by comparing intervention costs to household and community income levels.

**Results**: Mobile HIV testing services were most cost‐effective, with an ICER of UGX 185,000 per additional HIV diagnosis compared to standard facility‐based testing. Women's economic empowerment programmes reduced new HIV acquisitions by 18% over 6 months, with an ICER of UGX 440,000 per acquisition averted. Community‐led stigma reduction campaigns significantly increased HIV testing uptake (by 34%) and linkage to care (by 27%), with an ICER of UGX 310,000 per additional client linked to care. Affordability assessments indicated that while these interventions are effective, their sustainability remains heavily reliant on external donor funding, as household income levels within the settlement are insufficient to cover implementation costs.

**Conclusions**: The study highlights that mobile HIV testing, economic empowerment, stigma reduction interventions are cost‐effective and impactful in reducing HIV‐related risks in Nakivale Refugee Settlement. Achieving the long‐term affordability requires the integration of these interventions into Uganda's health financing systems and continued support from development partners. These findings provide critical guidance for policymakers and implementers to design scalable, cost‐effective, locally sustainable HIV programmes in refugee contexts.

### OAE0202 Domestic resource mobilization for HIV in Africa: a comparative analysis of country policies and practices

R.M. Ochanda ^1^, A. Mwangomale^2^, A. Banda^3^, A. Ngwenya^4^, J. MacWilliam^5^, W. Chikanya^6^, S. Shilagwa^7^, S. Wambua^8^, K. Mutale^9^, I. Tandolkar^5^, S. Malunga^8^, M. Luba^10^, V. Adula^11^

^1^AVAC, Policy Advocacy, Ngong, Kenya, ^2^Sikika, Policy Advocacy, Dar es Salaam, Tanzania, the United Republic of, ^3^COMPASS, DRM, Lilongwe, Malawi, ^4^Pangaea Zimbabwe, COMPASS, Harare, Zimbabwe, ^5^AVAC, Policy Advocacy, New York, United States, ^6^ZiCHIRe, Policy Advocacy, Harare, Zimbabwe, ^7^Focus for the Future Generation, Policy Advocacy, Dar es Salaam, Tanzania, the United Republic of, ^8^Key Population Transnational Collaboration, Policy Advocacy, Nairobi, Kenya, ^9^Key Population Transnational Collaboration, Policy Advocacy, Lusaka, Zambia, ^10^AVAC, Policy Advocacy, Lilongwe, Malawi, ^11^Consolation East Africa, Nairobi, Kenya

**Background**: This research explored how seven African countries are adapting their domestic resource mobilization (DRM) policies and strategies for HIV/AIDS in response to potential declines in international funding. The study analysed the challenges faced, prioritized strategies and innovative approaches adopted.

**Methods**: This study examined DRM strategies for HIV/AIDS in seven African countries: Nigeria, Zambia, Ethiopia, Malawi, Kenya, Tanzania and Zimbabwe. Employing a mixed‐methods approach, it analysed secondary data, including academic literature, grey literature (policy documents and reports) and each country's DRM strategies. Thematic analysis identified key themes such as common trends, challenges, best practices and the impact of DRM on national HIV/AIDS outcomes. Country selection criteria included receiving support from PEPFAR or the Global Fund and having established DRM policies. Focus group discussions (FGDs) in select countries corroborated findings from the secondary data analysis.

**Results**: While international funding remains crucial for HIV responses, its reliance varies significantly. PEPFAR data reveals that only one country contributed over 60% of its HIV funding from internal sources in FY 2024, while others contributed between 1% and 15%. UNAIDS data shows international health assistance persistently plateaued at annualized rate of 1.2% since 2012. This highlights the urgent need for countries to prioritize self‐financing strategies for their health and HIV programmes.

Common DRM strategies found included public‐private partnerships, earmarked taxes, health insurance, community contributions, integrated financing mechanisms and sector‐wide approaches. Emerging innovations include service integration, leveraging retrieved illicit funds for health, social contracting, utilizing technology and adopting innovative financing mechanisms. Other strategies include concessionary loans, debt conversions and risk pooling.

Key weaknesses hindering effective DRM include constrained budgets due to limited fiscal space, significant human resource for health (HRH) gaps, mismanagement of funds and weak health systems. While some countries have dedicated HIV or health financing policies, others lack such frameworks.

**Conclusions**: This research underscores the critical need for specific policies and practices to strengthen DRM for HIV/AIDS in Africa. Its findings aim to guide policymakers and stakeholders in designing and implementing more effective and sustainable strategies, ultimately contributing to the global goal of ending the HIV/AIDS epidemic by 2030.

### OAE0203 From donor to domestic HIV financing in sub‐Saharan Africa: a review of transition models, challenges and success factors (2015−2024)

P. Agboola ^1^, B. Aderounmu^2^

^1^Sage and Enamel Foundation, Osogbo, Nigeria, ^2^John Hopkins Bloomberg School of Public Health, Baltimore, United States

**Background**: The transition from donor to domestic financing for HIV programmes in sub‐Saharan Africa is a critical challenge faced by many countries as they strive to sustain their HIV response amidst dwindling external support. This narrative review examines transition models, challenges and success factors from 2015 to 2024, providing insights into how countries can effectively manage this shift and ensure the continuity of essential services.

**Methods**: We conducted a narrative review of evidence for combined terms related to HIV financing, donor transition, domestic resource mobilization and sub‐Saharan African countries. Evidence published between February 2015 and September 2024 was included. Data reported in this research article were obtained from reports, literature in peer‐reviewed journals found in PubMed, PubMed Central, and ScienceDirect, grey literature, UNAIDS database and other data sources. The authors also snowball further data to gather information for this review.

**Results**: Forty‐two studies were included in this review. Our findings reveal diverse transition models employed by countries in the region. For instance, Zimbabwe established an AIDS Trust Fund through a tax mechanism that generated approximately $52.7 million for HIV programmes. Botswana utilized a debt conversion instrument through its National HIV/AIDS Prevention Support programme, generating $20 million to enhance prevention efforts. Côte d'Ivoire implemented a Debt2Health agreement that provided $27 million for treatment and prevention initiatives. Uganda has creatively leveraged government funding from other health sectors to maintain HIV services amid donor transitions. However, significant challenges persist, including the risk of service disruption for key populations and reliance on unpredictable domestic funding sources. Successful transitions are often characterized by strong political commitment, effective stakeholder engagement and the adoption of innovative financing strategies that leverage local resources.

**Conclusions**: This review highlights the complexity of transitioning from donor to domestic HIV financing in sub‐Saharan Africa. While some countries have made significant progress through innovative approaches and strong leadership, others continue to struggle with dependency on external funding. To ensure the sustainability of HIV programmes, it is crucial for countries to enhance domestic resource mobilization and develop comprehensive strategies that address identified challenges.

### OAE0302 Point‐of‐care community delivery of the advanced HIV disease care (AHD) package during door‐to‐door TB‐case finding

S. Misra^1^, T.P. Pita^1^, T. Thahanyane^2^, M. Kamele^2^, M. Keitseng^2^, A. Tshazi^1^, I. Ayakaka^2^, T. Madonsela^1^, S. Bosman^1^, T. Decroo^3^, F. Vanobberghen^4^, E. Vlieghe^5,6^, K. Reither^4,7^, L. Lynen^3^, A. van Heerden^8^, T. Gils ^3,5^

^1^Human Science Research Council, Pietermaritzburg, South Africa, ^2^SolidarMed, Butha Buthe, Lesotho, ^3^Institute of Tropical Medicine, Clinical Sciences, Antwerp, Belgium, ^4^Swiss Tropical and Public Health Institute, Allschwil, Switzerland, ^5^University of Antwerp, Global Health Institute, Antwerp, Belgium, ^6^University Hospital Antwerp, Antwerp, Belgium, ^7^University of Basel, Basel, Switzerland, ^8^University of the Witwatersrand, Johannesburg, South Africa

**Background**: People with HIV (PWH) with AHD (PWAHD) (re)presenting to care are at high mortality risk. Community delivery of the AHD care package allows early AHD detection and management. The package was provided to PWH during door‐to‐door TB screening in (semi)rural Lesotho and South Africa (SA) (clinicaltrials.gov:NCT05526885). We evaluated the burden of AHD and implementation of the package.

**Methods**: Between September 2022 and 2024, consenting adult PWH were offered point‐of‐care CD4‐testing (VISITECT until a batch recall in April 2023, PIMA after that) and if CD4≤200 cells/µl, serum cryptococcal antigen (CrAg) and urine TB lipoarabinommaman (TB‐LAM) testing. PWH (re)‐entering care (with TB symptoms in Lesotho) and those eligible by a triage algorithm were offered sputum Xpert MTB/RIF Ultra (Xpert). A PIMA CD4≤200 cells/µl or positive Xpert (among those with access to both, others have “unknown” AHD status) was considered AHD. VISITECT results were inaccurate and not presented. Treatment initiation included anti‐retroviral treatment (ART), cotrimoxazole if CD4≤200 cells/µl and TB preventive therapy (TPT; if new HIV positive or not previously received) and Xpert positives were referred for TB treatment. PWAHD were referred to their health facility and traced after 56 days.

**Results**: 20,024 people were enrolled; 65.1% in Lesotho and 34.9% in SA. HIV positives represented 22.7% (4536); 19.7% (2568) of Lesotho and 28.2% (1968) of SA participants (Table 1). One thousand nine hundred and seventeen (42.3%) had valid CD4 results following the VISITECT recall and PIMA supply delays. Among them, 49 (2.6%) had CD4≤200 cells/µl and 7 (0.4%) were Xpert positive. Fifty‐five (2.9%) had AHD. All 46 CrAg and 45 TB‐LAM tests performed were negative.

**Table 1:** OAE0302

	Total PWH (*n*, col%)		Unknown AHD status (*n*, % of PWH)		Evaluated for AHD (*n*, % of PWH)		AHD (% of evaluated)		no AHD (% of evaluated)	
	4536	100	2619	57.7	1917	42.3	55	2.9	1862	97.1
Sex										
Female	3269	72.1	1902	58.2	1367	41.8	23	1.7	1344	98.3
Male	1264	27.9	716	56.6	548	43.4	32	5.8	516	94.2
Ambiguous	3	0.1	1	33.3	2	66.7	0	0.0	2	100.0
HIV status										
Known HIV positive on ART	4453	98.2	2556	57.4	1897	42.6	51	2.7	1846	97.3
Known HIV positive not on ART	22	0.5	16	72.7	6	27.3	1	16.7	5	83.3
Newly diagnosed with HIV	61	1.3	47	77.0	14	23.0	3	21.4	11	78.6
Country										
Lesotho	2568	56.6	1592	62.0	976	38.0	12	1.2	964	98.8
South Africa	1968	43.4	1027	52.2	941	47.8	43	4.6	898	95.4

Among eligible PWAHD, 54/55 were on ART, 6/7 on TB treatment, 7/28 on cotrimoxazole and 1/8 on TPT on day 56.

**Conclusions**: AHD prevalence was 3% among PWH during community recruitment. Community implementation of the AHD care package is conditional on the availability of appropriate CD4 tests. Prophylactic treatment initiation needs improvement.

### OAE0303 Reducing mortality and sustaining gains in advanced HIV disease management: a comparative analysis of transitioning support to the Ministry of Health in Malawi

T. Maphosa ^1^, L. Denoeud^2^, L. Kalitera^1^, L. Chilikutali^1^, E. Matiya^3^, J. Songo^4^, A. Mayi^2^, R. Nyirenda^5^, R. Machekano^2^, A. Tiam^2^

^1^Elizabeth Glaser Pediatric AIDS Foundation, Research, Lilongwe, Malawi, ^2^Elizabeth Glaser Pediatric AIDS Foundation, Research, Washington, DC, United States, ^3^Elizabeth Glaser Pediatric AIDS Foundation, Programmes, Lilongwe, Malawi, ^4^Elizabeth Glaser Pediatric AIDS Foundation, Strategic Information Systems, Lilongwe, Malawi, ^5^Ministry of Health, Department of HIV and AIDS, Lilongwe, Malawi

**Background**: To enhance sustainability and improve outcomes, the responsibility for advanced HIV disease (AHD) services transitioned from a project‐based approach to the Ministry of Health (MoH). This study evaluated the impact of this transition on AHD screening, CD4 testing uptake, tuberculosis (TB), cryptococcal meningitis (CM) management and 12‐month survival.

**Methods**: A retrospective cohort study was conducted at 22 health facilities during pre‐transition (June–December 2021) and post‐transition (April–September 2023). HIV client's records were reviewed to capture 12‐month follow‐up data. Inclusion criteria included individuals aged > 5 years with CD4 < 200 cells/mm^3^, WHO Stage 3/4 disease, newly diagnosed HIV, ART initiation < 12 months or ART instability > 12 months, as well as all children < 5 years initiating ART. Outcomes analysed included WHO staging, CD4 coverage, ART experience, TB and CM screening, and mortality. Statistical analyses included Cox proportional hazards modelling and competing risk regression.

**Results**: Among the 1042 clients enrolled (pre‐transition: *N* = 553; post‐transition: *N* = 489), 49.7% (*N* = 275) and 52.7% (*N* = 258) were female, respectively. The median age was 38 years (IQR: 30–46) pre‐transition and 40 years (IQR: 32–48) post‐transition. Hospital‐based care increased post‐transition (55.7% vs. 71.2%; *p* = 0.001). Advanced WHO Stage 3/4 presentations rose (47.6% vs. 65.3%; *p* = 0.001), as did ART‐experienced patients (24.2% vs. 44.6%; *p* = 0.001), while CD4 testing coverage declined (80.6% vs. 45.9%; *p* = 0.001). TB screening rates remained high (97.3% vs. 98.2%; *p* = 0.528), with increases in TB diagnoses (29.5% vs. 39.2%; *p* = 0.005) and urine LAM positivity (30.8% vs. 44.1%; *p* = 0.001). Mortality at 12 months significantly declined (9.4% vs. 5.5%; HR = 0.59, 95% CI: 0.37–0.94, *p* = 0.024)—see Table 1.

**Table 1:** OAE0303: Treatment outcomes and comparison of survival function between pre‐ and post‐transition periods

	Phase of AHD programme implementation	
	Pre‐transition	Post‐transition
***N***	553 (53.0%)	491 (47.0%)

	HR^*^(1)	95% CI	*p*‐value	HR^**^(2)	95% CI	*p*‐value
**Pre‐transition**	1.00			1.00		
**Post‐transition**	0.59	[0.37−0.94]	0.026	0.58	[0.36−0.93]	0.024

* (1) Cox regression.

** (2) Fine and Gray competing risk regression.

**Conclusions**: The successful transition of AHD project activities saw sustained critical outcomes and reduced mortality but highlighted gaps in CD4 testing. This approach must be used to scale up the intervention beyond these sites.

### OAE0304 Why are people living with HIV not prescribed TB preventive treatment at ART initiation? Insights from a cohort study

L. Steiner ^1^, K. Shearer^1^, L. Genade^2^, A. Pattamukkil^2^, B.A.S. Nonyane^3^, J.E. Golub^1^, L. Lebina^2^, N. Martinson^2^, C.J. Hoffmann^1^

^1^Johns Hopkins University, Center for Tuberculosis Research, Baltimore, United States, ^2^Perinatal HIV Research Unit (PHRU), University of the Witwatersrand, Johannesburg, South Africa, ^3^Johns Hopkins Bloomberg School of Public Health, Department of International Health, Baltimore, United States

**Background**: High rates of tuberculosis (TB) preventive treatment (TPT), especially at antiretroviral therapy (ART) initiation among people living with HIV (PWH), is critical for progress towards TB elimination. We characterized reasons for non‐prescription of TPT at ART initiation among PWH in the North West and Free State provinces of South Africa.

**Methods**: This study was nested within a cluster‐randomized trial conducted in 36 public‐sector primary health clinics. Medical records were abstracted for all adult (18+) PWH who had an HIV care‐related visit over a 1‐year implementation period. This analysis was limited to PWH who initiated on ART during the implementation period (between September 2021 and August 2023), including those re‐initiating treatment. Prescribing of TPT within 12 months of ART initiation and reasons for non‐prescribing TPT, as documented in the clinical file, are presented with chi‐square analysis for sex, age and province differences.

**Results**: Clinic records of 5004 PWH initiating ART were abstracted; 63% were female and the median (interquartile range [IQR]) age was 36 years (29, 44). Overall, 3651 PWH (73%) were prescribed TPT up to 12 months after ART initiation; 86% initiated TPT on the same day as ART. TPT prescribing differed by province (70% compared to 77%, *p* < 0.001) but not by sex or age. Among the 1335 (27%) PWH who were not prescribed TPT, complete data for the ART initiation visit was available for 1124 (84%). Nine hundred and forty (84% of those who did not receive TPT) lacked a clear reason for non‐prescribing of TPT; 82 (7%) had a TB investigation recorded, 76 (7%) were on TB treatment or completed TB treatment in the last 12 months, 6 (0.5%) completed TPT in the past 12 months and 1 (0.1%) evidence of a contraindication (liver disease, heavy alcohol use or hypersensitivity to INH). Nineteen (2%) additional patients had at least one TB symptom recorded without documentation of a TB investigation.

**Conclusions**: Approximately one‐quarter of PWH initiating or re‐initiating ART were not prescribed TPT with the majority not prescribed TPT appearing to meet South African TPT eligibility criteria. There remains a significant gap of people eligible, but not receiving TPT.

### OAE0305 Characteristics of HIV‐exposed infants testing positive on the Early Infant Diagnosis Programme, in Bunyoro, mid‐western Uganda, a retrospective cohort review from April 2021 to September 2024

N. Matovu ^1^

^1^Baylor Foundation Uganda, Medical & Psychosocial, Kampala, Uganda

**Background**: Early Infant Diagnosis (EID) of HIV is crucial for timely identification and antiretroviral therapy (ART) initiation among HIV‐exposed infants (HEIs) who seroconvert. Bunyoro registered a decline in vertical transmission of HIV (VT) from 2.3% in April 2021 to 1.8% in September 2024. However, HEIs still seroconvert on the EID programme, undermining efforts towards zero new infections by 2030. This study aimed to characterize HEIs who seroconvert on the EID programme in Bunyoro.

**Description**: A retrospective audit of HEI clinical charts from 91 health facilities assessed factors associated with VT using a positive infant audit tool. Ninety‐two percent (239/260) seroconverted at first DNA PCR (average age 7.1 months). The majority were from facility sick entry points, mother‐baby care points (MBC), community HIV testing and Expanded Program on Immunization (EPI) at 34% (88), 28% (74), 19% (49) and 15% (39), respectively. Maternal average age was 26 years (47% adolescent girls and young women [AGYW] [16−41 years]). Overall non‐ANC attendance was 47% (122/260), 65% (168/260) knew their HIV status during pregnancy, of whom 69% (116/168) either tested HIV positive before pregnancy or during first ANC, 31% (52/168) were HIV negative during ANC and seroconverted late in pregnancy or while breastfeeding (BF). HIV disclosure rates to spouses among pregnant women living with HIV stood at 49% (74/116). Despite a high ART initiation rate of 97% (253/260), 73% (185/253) initiated ART late (after second trimester), while 3% (7/260) declined ART due to non‐disclosure. Interruption in treatment (IIT) among the 116 mothers on ART before delivery was 81% (94), and 65% (168/260) of mothers had non‐skilled delivery.

**Lessons learned**: Facility sick entry points, MBC points, community HIV testing and EPI (96%) were key areas for identifying HIV‐positive infants, highlighting the need for strengthened EID/EPI integration. Late ART initiation (73%), IIT (81%) and low disclosure rates (49%) suggest substantial gaps in early ANC interventions. Seroconversion during BF (31%) requires intensified maternal retesting and PrEP integration.

**Conclusions/Next steps**: Strengthening EPI/EID integration, EID testing by 2 months and scaling up AGYW programming, targeted community‐based mobilization are some approaches that can improve timely prevention of VT and expand PrEP uptake.

### OAE0402 Towards elimination of HIV, syphilis and HBV mother‐to‐child transmission in The Gambia and Burkina Faso: the triple elimination model of mother‐to‐child transmission (TRI‐MOM) programme

A.E. Jallow ^1^, E. Vo‐Quang^2^, K. Jammeh^3^, B. Dibba^3^, G. Ndow^3,4^, A.E. Jallow^1^, E. Vo‐Quang^1,2^, J. Kitabu^2^, D. Bakary^2^, S. Drammeh^1^, L. Perieres^3^, U. D'Alessandro^1^, S. Boyer^3^, M. Lemoine^4^, G. Ndow^1,4^

^1^Medical Research Unit, The Gambia at London School of Hygiene and Tropical Medicine, Disease Control and Elimination, Fajara, Gambia, the, ^2^Univ Paris Est Créteil, INSERM, IMRB, Créteil, Paris, France, ^3^Medical Research Unit, The Gambia at London School of Hygiene and Tropical Medicine, Fajara, Gambia, the, ^4^Department of Metabolism, Digestion and Reproduction, Division of Digestive Diseases, Imperial College London, London, United Kingdom

**Background**: West Africa faces significant challenges with mother‐to‐child transmission (MTCT) of HIV, syphilis and hepatitis B virus (HBV) infections. Despite this burden, MTCT of these infections can be effectively prevented with straightforward interventions. The World Health Organization (WHO) has advocated for a “triple elimination” model, integrating the prevention of these infections into maternal care services to accelerate their control and elimination. The TRI‐MOM project aims to evaluate the feasibility of integrating the triple elimination model in two West African countries.

**Methods**: In The Gambia, the triple elimination strategy was deployed across four healthcare sites representing different levels of care. First, we conducted training for healthcare workers (HCWs) on the three infections to enhance awareness and capacity. Then, we offered free routine antenatal screening for the three infections using rapid diagnostic tests. Pregnant women testing positive for any of the three infections received appropriate treatment or prophylaxis to prevent MTCT. Data on MTCT outcomes and cost‐effectiveness were collected and analysed to assess the strategy's impact.

**Results**: Between January and March 2024, HCWs in government facilities were trained. Simplified screening and treatment/prophylaxis SOPs were developed and adapted per health facility. Between 18th March and 23rd December 2024, 6301 pregnant women were screened for the three infections. Overall, 99% of all pregnant women accepted screening.

Among these, 267 women (4.23%) tested positive for at least one infection: 75 had HIV mono‐infection, 173 had HBV mono‐infections, 14 had syphilis mono‐infection, 4 had HIV/HBV co‐infection and 1 had HIV/syphilis co‐infection. All HIV‐positive women were started on antiretroviral therapy (ART) following the “test‐and‐treat” approach. All syphilis‐positive women accepted treatment with benzathine penicillin, with no adverse reactions reported. Of 173 HBV mono‐infected women, 45 received TDF prophylaxis either based on high viral load (> 200,000 IU/ml) or based on a test‐and‐prophylax protocol.

**Conclusions**: The TRI‐MOM project demonstrates that integrating triple screening for HIV, syphilis and HBV into maternal care is both feasible and acceptable. Training of government HCWs, counselling of women and availability of rapid diagnostic tools have enabled seamless implementation. This approach can improve maternal and neonatal health outcomes and promote elimination of MTCT of these infections.

### OAE0403 Leveraging community‐based viral load sample collection to close treatment gaps among paediatrics and adolescents on ART in Zambia

R. Kanyinji^1^, R. Mwilu^1^, A. Mulenga^1^, E.K. Nyundu ^1^, B. Walusiku‐Mwewa^2^, G. Chanda^3^

^1^Access to Health Zambia, USAID Empowered Children and Adolescent Program I, Lusaka, Zambia, ^2^Access to Health Zambia, Lusaka, Zambia, ^3^USAID/Zambia, Lusaka, Zambia

**Background**: Despite significant global efforts, paediatrics and adolescents living with HIV continue to experience wide treatment gaps globally. In Zambia, paediatric and adolescent viral load (VL) coverage and suppression in 2021 were at 74% and 91%, respectively, falling short of the UNAIDS 95‐95‐95 targets. These gaps are exacerbated by barriers such as distance to health facilities, stigma, and limited paediatric and adolescent‐focused interventions. To close the treatment gap, the USAID Empowered Children and Adolescents Program I (ECAP I) partnered with PEPFAR clinical teams to implement a paediatric and adolescent‐focused surge.

**Description**: The surge focused on training, coaching and mentorship to 493 community health workers to conduct ethical index HIV testing and ensuring initiation on ART for paediatrics and adolescents newly diagnosed with HIV. Community VL sample collection was integrated and implemented across 100 health facilities in 13 high HIV burden districts in Zambia. At each health facility setting, a multidisciplinary team of clinicians, social workers and community health workers jointly conducted community VL sample collection focusing on paediatrics and adolescents on ART who were due for VL testing as well as those who were in “hard‐to‐reach” areas due to distance to the health facility. On average, the project covered about 5.4% of over 10,200 paediatrics and adolescents on ART who were eligible for VL testing each year from 2020 to 2024, for whom VL sample was collected, and result documented on file.

**Lessons learned**: The initiative has contributed to improvement in VL coverage for paediatrics and adolescents on ART from 86.4% in October 2021 to 95.6% in September 2024, while VL suppression was sustained at 96.9% from 87.0%, during the same period. Qualitative insights revealed that joint implementation of clinical and OVC programme interventions by multi‐disciplinary teams contributes to improving the health and wellbeing of marginalized priority sub‐populations.

**Conclusions/Next steps**: This intervention demonstrated the potential to improve VL coverage and suppression among paediatrics and adolescents, reducing persistent treatment gaps. Scaling this model to additional regions and integrating it with digital health solutions could further enhance its impact.

### OAE0404 Resilient HIV care in crisis: transforming access through drugs dispensing points in Haiti

D. Dorestan ^1^, B. Shaw^2^, N.R. Labbe^1^, M.C. Alcide Jean‐Pierre^1^

^1^Georgetown University, Center for Global Health Practice and Impact, Port au Prince, Haiti, ^2^Georgetown University, Center for Global Health Practice and Impact, Washington, United States

**Background**: Chronic and recent crises in Haiti, including insecurity, lockdowns, fuel scarcity and internally displaced populations, have severely impacted healthcare workers and disrupted HIV care. In response, the Georgetown University Center for Global Health Practice and Impact launched the Drugs Dispensing Points (DDP) project to improve treatment access. Haiti remains the Caribbean country most affected by HIV, with approximately 120,000 people living with HIV (PLHIV) on antiretroviral therapy (ART). This study highlights the context, challenges, implementation and results of the DDP strategy.

**Description**: Using Human‐Centred Design (HCD), the implementation team collaborated with the Ministry of Health (MoH), eight implementing partners and 261 stakeholders, including 108 PLHIV clients, to identify 159 strategic DDP locations. A DDP Priority Scale and a seven‐step Team Cycle Strike guided implementation. Selected sites underwent rigorous evaluation, and were equipped with trained staff, confidentiality measures and digital tools. Medication dispensation is monitored via the National Dispensing Interface Platform (nDIP), a web and mobile‐based ecosystem developed with SOLUTIONS SA. nDIP connects 167 treatment sites and 57 decentralized DDPs, offering real‐time updates on prescriptions, clinical statuses and service management. Over 100 treatment providers were trained on client referrals to DDPs, updating statuses, and ensuring seamless care coordination to maintain robust adherence and information flow within Haiti's HIV care network.

**Lessons learned**: Customization: Tailored DDP services through four organizational models—private pharmacies, PLHIV associations, community health centres and community‐based institutions—ensured local adaptability. Collaboration: Partnerships with MoH and stakeholders were key to identifying and establishing DDPs. Technology: nDIP enabled efficient medication management and real‐time care coordination. Training: Comprehensive provider training supported effective DDP operations and client adherence. Crisis Response: The DDP model proved resilient, with increased acceptance during crises.

**Conclusions/Next steps**: The DDP model has improved ART access for over 10,000 clients, achieving a 98% viral suppression rate. By integrating HCD, leveraging technology and fostering collaboration, the model reduces stigma, enhances care retention and adapts to Haiti's challenges. Continued investment will advance Haiti towards UNAIDS’ 95‐95‐95 targets and eliminate HIV as a public health threat.

### OAE0405 Unlocking access to advanced HIV disease care: lessons from Lesotho's phased strategy

T. Sefuthi ^1^, M. Milaham^2^, T. Tarumbiswa^3^, M. Thulo^4^, M. Tsotako^5^, M. Molapo^5^, S. Morebotsane^4^, N. Marake^3^, I. Amamilo^1^, L. Ntainyane^3^, M. Mohoanyane^3^, M. Dupreez^3^, E. Mandara^2^

^1^Clinton Health Access Initiative, HIV Access Program, Boston, United States, ^2^Clinton Health Access Initiative, Maseru, Lesotho, ^3^Ministry of Health, Maseru, Lesotho, ^4^Elizabeth Glaser Pediatric AIDS Foundation, Maseru, Lesotho, ^5^Baylor College of Medicine Children's Foundation, Maseru, Lesotho

**Background**: Advanced HIV disease (AHD) is a leading cause of death in Lesotho, with a prevalence of 14.5% among people living with HIV (PLHIV). Recognizing the severe socio‐economic toll of AHD on individuals and the health system, Lesotho prioritized a robust public health response. With support from CHAI, the Ministry of Health (MoH) introduced a comprehensive AHD treatment and management programme, achieving nationwide coverage by 2024 through a phased implementation approach.

**Description**: In 2019, CHAI, the Ministry of Health (MoH) and other development agencies developed national AHD management guidelines, the first‐ever national guidance dedicated to AHD. The MoH selected a hub and spoke model to deploy the care package. Hubs (district hospitals) provide screening, pre‐emptive therapy and treatment for all conditions, while spokes (health centres) screen and treat some opportunistic infections. In 2021, MoH introduced the AHD care package in all 18 hubs, referred to as phase I sites. Key enablers of rollout include implementation planning with MoH and partners, quantification and procurement of AHD focal commodities, healthcare worker training and programme monitoring. Applying this phased approach, MoH increased access to AHD services from 7% in 2021 to 31% and finally achieved 100% at all healthcare levels in 2024.

**Lessons learned**: The scale‐up of AHD management has been successful, but challenges remain in optimizing programme monitoring, increasing screening and treatment for diseases like cryptococcal meningitis and strengthening the AHD supply chain. A phased implementation approach has helped address these challenges given technical assistance resource constraints experienced during supportive supervisory visits. To further address these challenges, the government will leverage continuous quality improvement initiatives and conduct data quality assessments to enhance service delivery and data quality in 2025.

**Conclusions/Next steps**: A phased approach has enabled Lesotho to achieve nationwide AHD service coverage despite resource constraints. This underscores the government's commitment to reducing AHD‐related morbidity and mortality. Moving forward, sustained focus on high‐quality, integrated service delivery will be critical to reducing AIDS‐related deaths and securing long‐term programme success and impact.

### OAE0502 Outcomes from a community‐led monitoring (CLM) intervention in Malawi and South Africa

G. Oberth^1^, K. Lauer^2^, J. Bozinovski^3^, D. Mseu^4^, H. Kachepatsonga^4^, M. Soboyisi^5^, M. de Vos^6^, D. Mnkandla^5^, S. Baptiste ^7^

^1^University of Cape Town, Center for Social Science Research, Cape Town, South Africa, ^2^International Treatment Preparedness Coalition (ITPC), Seattle, United States, ^3^International Treatment Preparedness Coalition (ITPC), Lilongwe, Malawi, ^4^Malawi Network of Religious Leaders Leaving with or Personally Affected by HIV (MANERELA+), Lilongwe, Malawi, ^5^Networking HIV/AIDS Community of South Africa (NACOSA), Johannesburg, South Africa, ^6^Networking HIV/AIDS Community of South Africa (NACOSA), Cape Town, South Africa, ^7^International Treatment Preparedness Coalition (ITPC), Johannesburg, South Africa

**Background**: Since 2020, more than $100 million has been invested in community‐led monitoring (CLM), globally. To date, CLM research largely centres on the identification of issues with HIV services and barriers to access. Few studies show how CLM leads to concrete changes in service uptake or health outcomes. Rigorous evaluation methods are needed to demonstrate the effectiveness of the intervention.

**Methods**: Community‐led organizations systematically collected data on HIV prevention and treatment services from 33 health facilities in Malawi and South Africa between November 2020 and October 2024 (4 years). They routinely fed this data back to decision‐makers to inform service improvements. At the end of the project, we compared outcomes at the CLM sites with the other health facilities in the focus districts by analysing DHIS2. We also investigated whether more intensive CLM was associated with improved results. We calculated odds ratios (ORs) to determine the effect of the CLM intervention.

**Results**: Recipients of care at CLM sites were more likely to initiate pre‐exposure prophylaxis compared to other facilities (1.32 OR 95% CI 1.27−1.38). Pregnant women at CLM sites were twice as likely to deliver in the health facility, reducing the risk of vertical transmission (1.99 OR 95% CI 1.51−2.62). The CLM sites were also more likely than non‐CLM sites to find and diagnose adolescent girls and young women (AGYW) living with HIV (1.46 OR 95% CI 1.28−1.66). As a result, the cost to diagnose one AGYW living with HIV was $2852 at the CLM sites compared to $4154 at the non‐CLM sites. More intensive CLM was associated with improved service coverage and health outcomes. At CLM sites with additional resources for community scorecards, community mobilization and community dialogues, people living with HIV were six times more likely to access treatment through a differentiated service delivery model (6.79 OR 95% CI 6.04−7.63). People at the intensive CLM sites were twice as likely to be virally suppressed compared to the standard CLM intervention (2.34 OR 95% CI 2.16−2.54).

**Conclusions**: It is possible to evaluate CLM interventions using rigorous scientific methods. Identifying a control group can be helpful to demonstrate the effectiveness of CLM.

### OAE0503 From barriers to breakthroughs: the impact of community‐led monitoring on healthcare access in Liberia

B. Gibson ^1^, T.F. Musa^2^, J. Godoe^2^, M. Dahn^1^, E.M. Dioukhane^3^, M. Tegli^1^, J. Flomo^4^, A. Jaygbeh^1^, P. George^1^, T. Ahmad^1^

^1^Plan International, HIV/TB Program, Monrovia, Liberia, ^2^Liberia Network of People Living with HIV, Program, Monrovia‐, Liberia, ^3^Plan International, HIV/TB Program, Toronto, Canada, ^4^National AIDS Control Program, HIV, Sinkor, Liberia

**Background**: The Liberia Network of People Living with HIV (LibNeP+) with funding from the Global fund has made significant strides in advancing Community‐Led Monitoring (CLM) to enhance accountability, equity and quality in healthcare delivery. With technical assistance from Community‐Led Accountability Working Group (CLAW) consortium, LibNeP+ piloted the CLM model in five counties—monitoring 28 sites, including 14 health facilities, 2 drop‐in centres and 12 populated areas. This pilot aimed to identify systemic barriers, enhance service delivery and empower communities to advocate for people‐centred solutions.

**Methods**: The 6‐month pilot (July–December 2023) engaged 14 peer monitors to collect and analyse data from facility managers, nurses, people living with HIV (PLHIV), key populations (KPs), and pregnant women using qualitative and quantitative tools. We conducted comprehensive training to 22 member organizations, including county supervisors, key population‐led and PLHIV‐led organization, and LibNeP+ staff, with support from the National AIDS Control Program (NACP). Training topics included CLM setup, data analysis and evidence‐based advocacy to enhance CLM efforts by involving more community‐based organizations. Data collection included interviews with facility managers, ART clinicians and recipients of care. Findings informed targeted interventions through rapid feedback loops with healthcare providers and decision‐makers.

**Results**: The pilot revealed critical gaps in healthcare delivery and demonstrated the transformative impact of CLM interventions.

Metric Baseline (Pre‐Intervention Post‐Intervention Change)[Table jia226518-tbl-0013]

**Table 1 jia226518-tbl-0013:** OAE0503

Metric	Baseline (pre‐intervention)	Post‐intervention	Change
Average wait time (hours)	3.9	2.5	↓ 36%
Queue length	22	15	↓ 32%
PrEP awareness	67%	85%	↑ 18%
PrEP uptake (% offered PrEP)	50%	70%	↑ 20%
Stock‐outs	13%	5%	↓ 62%
ART refill periods (% offering 3−6 months)	50%	80%	↑ 30%
KP‐specific services available	67%	85%	↑ 18%
Client satisfaction	85%	92%	↑ 7%

**Conclusions**: The CLM pilot in Liberia demonstrated the impact of community‐driven approaches in addressing systemic healthcare barriers. Peer monitors fostered trust and provided actionable insights, leading improve quality of care. Building on its success, we plan to scale CLM nationwide. Regular data‐sharing forums with key decision‐makers will sustain momentum and drive systemic improvements, positioning CLM as a replicable model for equitable, community‐centred healthcare transformation.

### OAE0504 Advancing HIV service quality through community‐led monitoring system in Chiang Mai, Thailand

S. Sittikarn ^1^, C. Wongwaiphanit^1^, P. Patpeerapong^2^, L. Mahawan^3^, S. Sangtong^3^, K. Khomkaew^3^, P. Palee^3^, P. Chaisopa^3^, K. Singchaichit^3^, S. Janla^3^, N. Suwanphatthana^3^, K. Sirisup^4^, S. Santayakul^4^

^1^CAREMAT Foundation, Chiangmai, Thailand, ^2^MPLUS Foundation, Chiangmai, Thailand, ^3^Chiang Mai Community‐Led Monitoring Committee, Chiangmai, Thailand, ^4^FHI 360, USAID EpiC project, Bangkok, Thailand

**Background**: People‐centred HIV services that respond to the needs of clients necessitate feedback mechanisms such as community‐led monitoring (CLM) where stigmatized population groups can voice their concerns and perspectives on how to improve HIV testing, treatment and pre‐exposure prophylaxis (PrEP).

**Description**: A group of civil society and key population (including people‐living‐with‐HIV [PLHIV]) representatives in Chiang Mai, Thailand launched a people‐centred CLM system in January 2022. This CLM aims to improve HIV service delivery by collecting and analysing data on service quality and clients’ satisfaction through structured feedback tools, surveys and interviews with clients to identify gaps and formulate improvement plans. This initiative involved 17 committee members and 21 hospitals collaborating to enhance service quality per the evolving needs of key populations.

**Lessons learned**: Data collection was conducted during March 2022−November 2024 and yielded 3425 responses from 21 hospitals across Chiang Mai.[Table jia226518-tbl-0014]

**Table 1 jia226518-tbl-0014:** OAE0504

CLM results	Outcomes
Clients reported low or no access to PrEP in many hospitals. (2022)	Expansion of PrEP facilities from 13 to 24 hospitals across all 24 districts. (2023)
PLHIV felt that multi‐month dispensing (MMD) was not offered to them by providers. (2022)	Provincial Health Office‐led strategy to scale up 6‐month MMD at 24 hospitals. (2024)
PLHIV reported that U = U was not integrated in counselling. (2023)	Provincial Health Office launched a comprehensive U = U campaign. (2024)

In addition to CLM findings, the structured feedback tools were formalized into standard operating procedures (SOPs) for data collection and analysis and subsequently scaled up to three other provinces: Chiang Rai, Phitsanulok and Ubonratchathani.

**Conclusions/Next steps**: CLM is vital in creating a participatory environment where key populations monitor and review HIV services. Through the implementation in Chiang Mai during March 2022−November 2024, CLM improved PrEP service availability to cover all districts in the province, reduced travel burden for ART refills for clients living with HIV and increased awareness of U = U (Undetectable = Untransmittable) among the public, key population communities and service providers. With standardized tools and community leadership, CLM proves to be an effective and scalable process to enhance HIV service quality in multiple provinces.

### OAE0505 Accessibility of HIV services for key and vulnerable populations (KVPs) in criminalized contexts: findings from KVP‐led community monitoring in Dar es Salaam

P. Liberatus ^1^, M. Mutongore^1^, C. Peter^1^, Z. Mansoor^1^, A. Nicholaus^1^, M. Milanga^2^, E. Lankiewicz^3^

^1^The Key and Vulnerable Populations Forum Tanzania, Dar es Salaam, Tanzania, the United Republic of, ^2^Health GAP, Nairobi, Kenya, ^3^amfAR, The Foundation for AIDS Research, Washington, DC, United States

**Background**: Ending the HIV epidemic will require closing gaps for most at‐risk populations. In contexts where key and vulnerable populations (KVPs) are stigmatized and criminalized such as Tanzania, this will require continuous availability of friendly services. However, accurate assessments of the state of healthcare for KVPs are challenging in these contexts. This project aimed to fill this gap with monitoring of service accessibility by and for KVPs.

**Description**: The Key and Vulnerable Populations (KVPs) Forum in Tanzania spent 2 weeks collecting community‐led monitoring data among KVPs in Dar es Salaam in September 2024. An electronic data collection system was used, with data collected by key populations in Kiswahili at community hot spots. One thousand three hundred and forty‐three KVPs consented to interviews (336 transgender individuals, 314 sex workers, 298 people who use drugs, 194 men who have sex with men [GBMSM], and 201 adolescent girls and young women [AGYWs]).

**Lessons learned**: These data reveal that despite promises of non‐discriminatory healthcare provision, services, especially at public health facilities, remain discriminatory and inaccessible to many. More than half (54%, *n* = 191) of KPs surveyed who use public facilities had been refused services in the last year, including 80% (*n* = 41) of GBMSM and 75% (*n* = 106) of transgender people. Many fear arrest when seeking services (Figure 1). More than half (53%, *n* = 159) of KVPs felt very unsafe or unsafe at facilities. Lack of safety at facilities extended beyond criminalized populations to AGYWs as well, with 57% (*n* = 30) of AGYWs using public health facilities reporting feeling unsafe.

**Figure 1:**
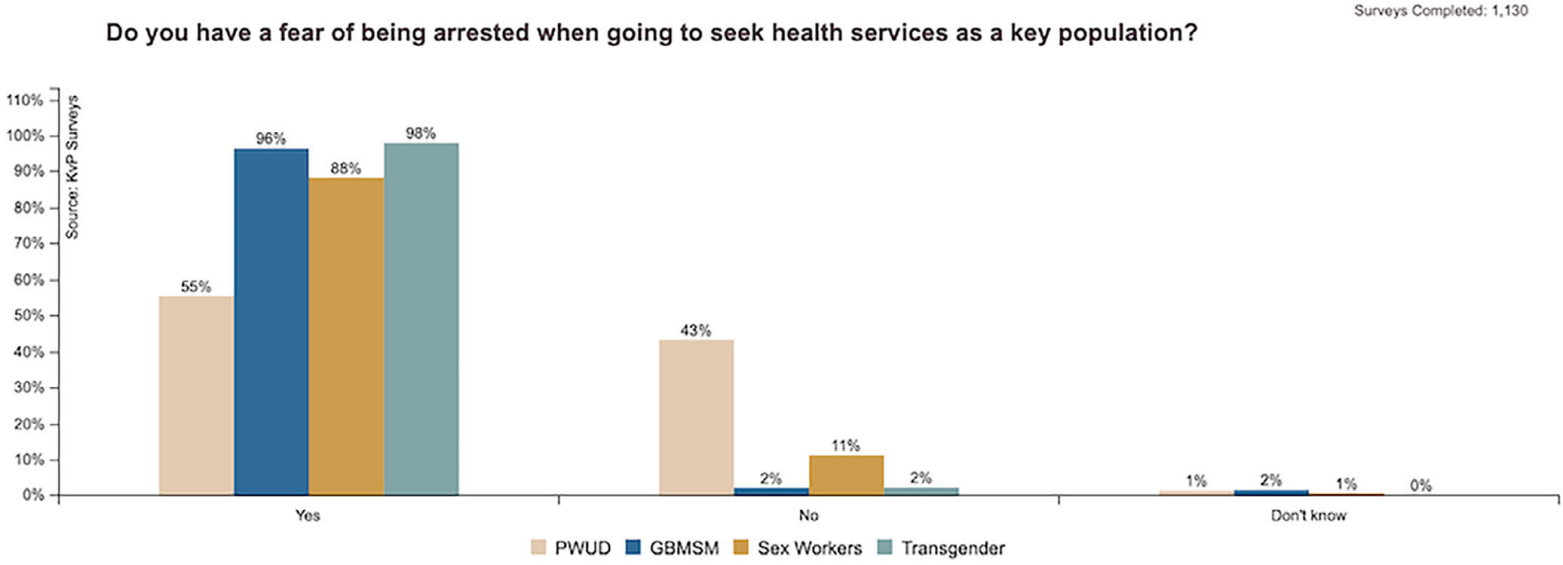
OAE0505

**Conclusions/Next steps**: These data represent one of the largest samples of KVPs in Tanzania, pointing to the importance of data collection for vulnerable populations by vulnerable populations. As international funders elevate sustainability conversations where policies fail to protect human rights, programmes like KVP‐led CLM are critical to ensuring we have accurate pictures of the lived realities of HIV service accessibility.

### OAE0602 Enhancing data quality for differentiated service delivery: lessons from Zimbabwe's multiregional DSD review

T. Matare ^1^, P. Mujuru^2^, C. Gwanzura^1^, C. Mupanguri^1^, T. Apollo^1^

^1^Ministry of Health and Child Care (MOHCC), HIV, Harare, Zimbabwe, ^2^Zimbabwe Technical Assistance, Training and Education Center (Zim‐TTECH), HIV Care and Treatment, Harare, Zimbabwe

**Background**: Differentiated service delivery (DSD) is a client‐centred approach that simplifies and adapts HIV services to reflect clients’ preferences and expectations while reducing unnecessary burdens on the health system. Zimbabwe started implementing DSD models for ART in 2017 and has been conducting DSD reviews annually since 2018. They assess implementation progress and peer‐learning opportunities to share best practices, challenges and innovations. The 2024 DSD review was conducted in July–August 2024.

**Description**: A total of 148 facilities in 30 districts from four provinces participated. Data was collected using Survey CTO and analysed using PowerBI. A facility survey and individual patient survey tools were used. Data was abstracted from 4630 patient files for recipients of care (RoCs) in the 12‐, 24‐ and 36‐months ART cohorts. Data was collected from paper‐based tools and/electronic medical records for services received across the HIV continuum of care for HIV testing, treatment, management of high viral load and integration with tuberculosis, family planning, mental health, opportunistic infections and non‐communicable diseases.

**Lessons learned**: As Zimbabwe nears epidemic control, it is imperative that available data sources are robust, as we provide client‐centred services, for better data‐driven decisions. However, the review showed incompleteness on some key patient variables such as DSD model (22%), date of birth (4%), date of HIV diagnosis (4%), sex (1%) and date of ART initiation (1%). This is despite having standard operating procedure for recording and reporting. Zimbabwe intensified the development of a module for DSD models with validation rules and subsequent data visualizations within electronic health record (EHR) with support from PEPFAR. The module was refined after several validations and deployed to 795 facilities. Zimbabwe plans to decommission all paper tools, inter‐connect DHIS 2 with EHR and eventually utilize EHR to do DSD reviews.

**Conclusions/Next steps**: The review provided an opportunity for assessing data quality. We learnt that data quality was sub‐optimal on some variables. Electronic medical records provide an opportunity for improving data quality. However, there is a need for governments and implementing partners to tackle challenges around connectivity, power, hardware, software development and human resources for health capacity building to sustain the system for better data quality.

### OAE0603 Decentralizing HIV care: blockchain solutions for client data integrity and pharmaceutical supply chain resilience

P.C. Rupasinghe ^1^, N.G. Abdelrahman^2^, H.M.E. Mahgoub^3^, S. Asaad^4^

^1^Icahn School of Medicine at Mount Sinai, Public health‐ Epidemiology, New York, United States, ^2^Michigan State University, Geriatric Medicine, East Lansing, United States, ^3^Michigan State University, Tele‐Geriatric Research Fellowship, Jeddah, Saudi Arabia, ^4^Michigan State University, Tele‐Geriatric Research Fellowship, Houston, United States

**Background**: Blockchain technology is revolutionizing HIV care by addressing critical challenges in data management and pharmaceutical supply chains. Its decentralized, tamper‐proof architecture offers solutions to fragmented systems, privacy risks and inefficiencies that undermine global treatment equity.

**Description**: Blockchain technology has demonstrated transformative potential across HIV care delivery, with applications spanning client data management and pharmaceutical supply chains. In Kakamega, Kenya, the Immunify.Life initiative replaced paper‐based systems with blockchain‐enabled real‐time data tracking, improving treatment adherence by 25% among non‐adherent clients over 12 months. Parallel efforts in Houston, Texas, utilized blockchain platforms for pre‐exposure prophylaxis (PrEP) access, achieving 83% user trust and reducing administrative errors by 40% through secure, decentralized health records (Yue et al., 2022). Beyond clients care, blockchain strengthened supply chain resilience: in Malawi, tracking of ARV drugs reduced clinic stock outs from 25% to 12% and lowered counterfeit drug rates by 35% across 150 clinics (Gavi, 2023), while a Ghanaian pilot cut HIV test kit delivery delays by 50% in rural areas through blockchain‐monitored shipments. These geographically diverse implementations highlight blockchain's dual capacity to enhance care coordination and safeguard medical supply chains, addressing systemic inefficiencies in both high‐ and low‐resource settings.

**Lessons learned**: Blockchain enhances data security, interoperability and supply chain transparency. Key successes include adherence gains (25% in Kenya), error reduction (40% in the United States) and counterfeit drug mitigation (35% in Malawi). Challenges such as infrastructure gaps (e.g. rural internet access) and regulatory complexities (e.g. HIPAA compliance) demand hybrid models and public‐private partnerships for scalability.

**Conclusions/Next steps**: Blockchain's success in improving client adherence, reducing counterfeit drugs and streamlining supply chains underscores its potential to revolutionize HIV programmes globally. However, scaling these innovations requires addressing infrastructure gaps (e.g. internet access in rural areas), regulatory alignment (e.g. HIPAA compliance) and stakeholder training. Future efforts should prioritize hybrid blockchain models to balance transparency with compliance, foster cross‐sector collaboration to expand solutions, and explore broader applications in chronic disease management and cross‐border health data sharing. By integrating blockchain with emerging technologies like AI, healthcare systems can unlock predictive analytics for supply chain optimization and personalized client care, positioning blockchain as a cornerstone of equitable, client‐centred healthcare worldwide.

### OAE0604 Innovative use of mobile digital chest‐X‐ray equipped with artificial intelligence to improve TB diagnosis among people living with HIV at primary health centres (PHCs) in Lagos, Nigeria

O. Udunze ^1^, A. Agbaje^2^, D. Olugbenga^1^, E. Ajayi^1^, D. Sokoya^3^, E. Ubochioma^4^, A. Omolaja^5^, B. Odume^6^, T. Osatuyi^7^, E. Chukwu^8^

^1^Institute of Human Virology Nigeria, Strategic Information (S.I), Lagos, Nigeria, ^2^Institute of Human Virology Nigeria, Lagos, Nigeria, ^3^Lagos State Ministry of Health, STBLCP, Lagos, Nigeria, ^4^National Tuberculosis and Leprosy Control Programme, Program, Abuja, Nigeria, ^5^Grant Management Unit, Lagos State Ministry of Health, Lagos, Nigeria, ^6^KNCV Nigeria, Executive Director, Abuja, Nigeria, ^7^KNCV Nigeria, Project Coordinator, Abuja, Nigeria, ^8^KNCV Nigeria, Regional Project Coordinator, Abuja, Nigeria

**Background**: Deployment of mobile digital chest X‐ray (CXR) vans equipped with artificial intelligence (CAD4TB) is a joint investment of the State Government and Global Fund to complement other interventions in TB programme, to find and treat the missing persons with TB. The CXR intervention will further increase access to free TB screening and treatment as part of the global strategy to end TB. Three mobile CXR vans, equipped with artificial intelligence (CAD4TB), were used for this activity. This provided an opportunity for adequate TB/HIV integration and collaboration at the PHCs. The objective of this study is to highlight the effectiveness of TB/HIV service integration at the primary healthcare centres, characterized with client referrals to other designated diagnostics centres for evaluations and further follow‐up examinations.

**Description**: The use of the mobile digital CXR at the PHCs for active TB case finding was conceptualized and conducted from January to December 2024 to address the existing 32% TB notification gaps in Lagos Nigeria. This, however, provided an opportunity to adequately screen PLHIV for TB at PHCs. DHIS2 was used to determine the PLHIV currently on treatment across eight flagship sites (PHCs) in the state, following facility assessment and advocacy visit. TB facility cascade management team meeting was conducted and CXR mobile vans were deployed. WHO 3B TB screening algorithm was adopted. Specimens were collected and sent for GeneXpert test.

**Lessons learned**: For the AI‐supported TB screening among PLHIV, the average TB yield as calculated is 23%, NNS and NNT calculated were 24 and 4, respectively, given a better yield compared to the conventional TB screening (symptom screening at 8% TB yield, 123 NNS and 12 NNT calculated, respectively).[Table jia226518-tbl-0015]

**Table 1 jia226518-tbl-0015:** OAE0604

Type of TB screening (screening with AI‐supported CXR vs. symptom screening)	No. of PLHIV screened for TB with AI‐supported CXR mobile machine	No. of TB presumptive among PLHIV	No. of TB presumptive clinically evaluated	No. diagnosed with TB	No. linked to care	% TB yield	No. needed to screen (NNS)	No. needed to test (NNT)
TB screening using AI‐supported CXR at PHCs	1128	203	203	47	47	23%	24	4
Symptom screening at other secondary facilities	7898	789	789	64	57	8%	123	12

*Note*: Comparative quarterly data showing the TB cascade for both AI‐supported and non‐AI‐supported screening activities.

**Conclusions/Next steps**: Artificial intelligence‐powered mobile digital CXR has demonstrated efficiency in TB diagnosis among PLHIV while fostering adequate integration of TB/HIV. Therefore, the expansion of mobile digital CXR equipped with AI will further strengthen this collaboration and improve the quality of care among PLHIV in Nigeria.

### OAE0605 Ethical concerns and suggested mitigations surrounding access to and use of SMS and social media data in sentiment analysis, for HIV prevention among young women in Western Kenya

A. Ochiel ^1^, Z. Kwena^1^, K. Oware^1^, P. Ochwal^1^, L. Adiema^1^, M. Hewa^1^, J. Odoyo^1^, B. Rono^1^, L. Garrison^2^, E. Bukusi^3^, J. Haberer^2,4^

^1^Kenya Medical Research Institute, CMR‐RCTP, Kisumu, Kenya, ^2^Massachusetts General Hospital, Obstetrics and Gynecology, Boston, United States, ^3^Kenya Medical Research Institute, Center for Microbiology Research, Nairobi, Kenya, ^4^Harvard Medical School, Obstetrics and Gynecology, Boston, United States

**Background**: As artificial intelligence (AI) technology gains popularity, innovative smart phone apps based on predictive algorithms are emerging. Moreover, the public is increasingly using these apps to acquire new knowledge, solve problems, and trade goods and services. Given the vast amounts of inter‐personal communication via SMS and social media, AI‐driven apps could analyse these data to predict individual behaviours and health‐related risks. We sought to explore the ethical concerns and suggested mitigations in accessing young women's phone data for the development of an AI‐based tool to assess HIV prevention needs.

**Methods**: Between February and April 2024, we conducted in‐depth interviews (IDIs) with 32 young women aged 18−24 who sought sexual and reproductive health services from four public facilities. Half of them were willing to share their SMS and social media messages for HIV prevention analysis. Additionally, two focus group discussions were held with 11 bioethicists. Ethical concerns were explored through questions covering privacy, confidentiality, autonomy, beneficence, non‐maleficence, justice and power. Data were thematically analysed using a deductive approach.

**Results**: Both groups expressed concerns about likelihood of privacy intrusion, risks of accessing third‐party and deleted messages, potential for embarrassment, confidentiality breaches and data leaks during algorithm development. Bioethicists were worried about practicality of obtaining true informed consent due to the complexity of sentiment analysis.

Young women suggested strict data security measures, such as de‐identifying data and involving non‐Kenyans in data handling. Bioethicists stressed the importance of carefully developing consent forms through iterative participation to obtain truly informed consent from participants. Some suggested exploring the feasibility of using public social media data, instead of personal data, to develop the AI‐based HIV risk assessment tool prototype. Bioethicists recommended ethical bodies to define guidelines and legal thresholds for allowable confidentiality breaches in cases involving the discovery of criminality.

**Conclusions**: These findings offer data access insights for researchers designing AI‐driven prediction tools for HIV prevention and other health‐related behaviours: to help with upholding critical ethical principles including privacy, confidentiality and non‐maleficence. Ethical review boards should define minimum standards for participants’ protection in research employing sentiment analysis, as it is crucial for the proposed app and similar future tools.

## LATE BREAKING ABSTRACTS

### OAA0106LB Defective HIV‐1 proviruses recombine to restore infectious virus: a new barrier to cure

H. Imamichi ^1^, T. Imamichi^2^, F. Scrimieri^2^, B.T. Sherman^2^, W. Chang^2^, H.C. Lane^1^

^1^National Institute of Allergy and Infectious Diseases, Bethesda, United States, ^2^Frederick National Laboratory for Cancer Research, Frederick, United States

**Background**: Recent studies have shown that “defective” HIV‐1 proviruses are capable of transcription and translation into proteins. This study was designed to directly investigate whether these “defective” proviruses can recombine to regenerate replication‐competent virus.

**Methods**: We established a model system using two defective HIV‐1 proviruses derived from a person with HIV (PWH), each containing distinct large internal deletions: a 3'‐deleted provirus with a 3.8 kb internal deletion missing the vif, vpr, vpu, tat, rev, env and nef regions, and a 5'‐deleted provirus with a 3.3 kb deletion missing the gag and pol regions. Notably, the precursor frequency of full‐length intact HIV‐1 proviruses in the PWH was 1 in 697 sequences (∼0.1%), based on the analysis of autopsy tissue specimens. Primary CD4^+^ T cells from HIV‐negative donors were co‐transfected with pNL4‐3 plasmids containing synthesized inserts representing the 3′‐ or 5′‐deleted HIV‐1 proviruses. Viral production was assessed by measuring p24 antigen levels and examining viral particle morphology via transmission electron microscopy (TEM). HIV‐1 infectivity was evaluated through four rounds of in vitro passaging. Single‐genome amplification (SGA) and full‐length HIV‐1 sequencing were used to characterize recombination events.

**Results**: We demonstrate for the first time that two defective HIV‐1 proviruses can recombine to regenerate a fully replication‐competent virus. Co‐transfection resulted in robust p24 production (1100 ng/ml at day 7 post‐transfection) and successful infection of primary CD4^+^ T cells (9900 ng/ml at day 7 post‐infection). Sequence analysis revealed recombination events restoring intact open reading frames and correcting deleterious large internal deletions. Critically, the emergent viruses replicated with kinetics comparable to wild‐type HIV‐1. These findings show that defective proviruses, once thought to be biologically inert “dead‐end” products, can actively contribute to viral persistence and reservoir replenishment.

**Conclusions**: Recombination between defective HIV‐1 proviruses represents a novel and active mechanism of viral evolution, with direct implications for cure strategies, and may help explain the source of delayed viral rebound in some rare cases following ART interruption. Strategies for HIV‐1 eradication must consider the potential for defective genomes to recombine and regenerate infectious virus, posing a new and significant barrier to achieving durable remission.

### OAA0306LB Cross‐clade HIV‐1 neutralizing antibodies with characteristics of bNAbs in paediatric populations of humans and rhesus macaques

R. Tuck^1^, A. Nelson^2^, Y. Chen^1^, S. Holmes^1^, S. Rohr^1^, M. Berry^1^, A. Eaton^1^, R.J. Edwards^1^, K. Mansouri^1^, T. Spence^1^, M. Clark^1^, D.N. Chaturbhuj^3^, P.J. Klasse^3^, M.S. Seaman^4^, D.C. Montefiori^1^, K. Wiehe^1^, G. Fouda^2^, W. Williams ^1^

^1^Duke University School of Medicine, Duke Human Vaccine Institute, Durham, United States, ^2^Weill Cornell Medicine, Pediatrics, New York, United States, ^3^Weill Cornell Medicine, Microbiology and Immunology, New York, United States, ^4^Harvard Medical School, Beth Israel Deaconess Medical Center, Cambridge, United States

**Background**: Induction of HIV‐1 envelope (Env)‐reactive broadly neutralizing antibodies (bNAbs) is a primary goal of a prophylactic HIV‐1 vaccine. There is a dearth of bNAbs from paediatric populations that may be used to inform immunogen design for their induction and maturation in infants and children through vaccination.

**Methods**: We assessed HIV‐1 neutralization breadth in 13 young rhesus macaques (RMs) following clade C SHIV infection during infancy and 47 children with perinatally acquired clade B HIV‐1. We isolated Env SOSIP‐trimer reactive B cells from blood of representative subjects with broad and potent responses. Recombinant monoclonal (m) Abs with paired variable heavy (V_H_) and light (V_L_) genes per B cell were functionally characterized and visualized for Env‐interaction via negative stain electron microscopy.

**Results**: Of 13 SHIV‐infected RMs, 62% developed plasma heterologous HIV‐1 NAbs. We isolated two clonally related mAbs (DH1518.1 and DH1518.2) that used V_H_4‐NL‐22 (17 amino acid [aa] complementarity‐determining‐region‐3/HCDR3) and V_K_2‐X from one RM at month 14 post‐infection. The DH1518 mAbs neutralized ∼13% of 119 difficult‐to‐neutralize/tier 2 multiclade HIV‐1 strains (Geometric mean, IC50: ∼8−11 µg/ml) and mapped to a discontinuous epitope partially occluded by glycans and span V2‐apex, V3‐glycan and CD4‐binding site (BS) bNAb epitopes. Additionally, 34% of children with HIV‐1 showed heterologous tier 2 HIV‐1 NAbs. We isolated two mAbs, DH1668 and DH1669, from B cells sampled at 3.5 years‐of‐age of a child who developed neutralization breadth at 1 year‐of‐age. The mAbs DH1668 used V_H_3‐21 (28aa‐long HCDR3) and V_K_1‐33, and DH1669 used V_H_3‐33 (5aa‐long HCDR3) and V_L_4‐69, and mapped to CD4BS and V3‐glycan bNAb epitopes, respectively. Compared to DH1668, DH1669 had maximal neutralization activity against 37% (11/30) of multiclade isolates (Geometric mean, IC50: 0.25 µg/ml), and neutralized 40% (4/10) of breastmilk‐derived clade C isolates. All isolated mAbs were derived from subdominant B cell subsets in subjects studied.

**Conclusions**: We defined paediatric cross‐clade HIV‐1 NAbs that target bNAb epitopes, thus implicating that they may be matured over time with appropriate vaccine immunogens. Ongoing studies will identify additional NAbs in infant RMs and humans to inform immunogen design for early life prophylactic bNAb‐inducing HIV‐1 vaccines—a potential strategy for ending the HIV‐1 pandemic.

### OAA0406LB Increased systemic inflammation and plasmacytoid dendritic cells in people living with HIV with history of cured pulmonary TB

M. Ward^1^, Y. Joseph^2^, D. Lespinasse^2^, P. Zumbo^1^, A. Apollon^2^, E. Dumont^2^, D. Fitzgerald^1^, J. Pape^1,2^, K. Dupnik ^1^

^1^Weill Cornell Medicine, New York, United States, ^2^GHESKIO, Port au Prince, Haiti

**Background**: In Haiti, people living with HIV who have a history of TB have increased all‐cause mortality despite virologic suppression and TB cure (Joseph, 2021). Our objective was to assess for the post‐TB immunologic profile in people living with HIV. We hypothesized that people with history of TB have enduring elevated inflammatory cells and cytokines which contribute to worse outcomes after TB.

**Methods**: Peripheral blood mononuclear cells were collected from people living with HIV who participated in an observational study on TB recurrence at GHESKIO Centers in Port au Prince, Haiti between 2019 and 2023. We completed Cellular Indexing of Transcriptomes and Epitopes by Sequencing (CITE‐Seq) of peripheral blood mononuclear cells (PBMCs) of people living with HIV with (*n* = 6) or without (*n* = 3) TB history. Analyses of CITE‐Seq data included differential gene expression, principal component analyses and pathway analyses. We completed confirmatory dendritic cell subtyping with flow cytometry and plasma cytokine quantification using an 18‐plex Th1/Th2 panel on an expanded group of people (*n* = 29).

**Results**: There were more than 40 differentially expressed genes in the T lymphocyte, B lymphocyte, natural killer cell and dendritic cell populations. In each of these immune subtypes, the Hallmark gene set “regulated by NF‐kB in response to TNF” was over‐represented. In the dendritic cell subset, there was over‐representation of pathways important for response to TB: response to hydrogen peroxide, oxidative stress and reactive oxygen species in the 68 differentially expressed genes. In the expanded cohort of 29 people with HIV, there was a larger percentage of plasmacytoid dendritic cells by flow cytometry and increased plasma IL‐6, IL‐12p70, IL‐15, IL‐2, IFN‐alpha and TNF in the TB history group (*n* = 18) compared to people with no history of TB (*n* = 11). These findings remained statistically significant when assessed with Mann‐Whitney non‐parametric tests and with tobit logistic regression to account for left censoring with and without sex included as a covariate.

**Conclusions**: A pro‐inflammatory milieu and immune cell gene expression changes mediated by TNF persist after TB cure for people living with HIV. If the differences are preexisting risk factors or establish during the natural history of HIV and TB is still to be determined.

### OAA0506LB Impact of AGT103‐T on the HIV proviral reservoir: insights from a modified IPDA in a phase 1 study

G. Canepa ^1^, L. Xiao^1^, A. Jain^2^, M.‐L. Liou^1^

^1^American Gene Technologies, Discovery, Rockville, United States, ^2^American Gene Technologies, Rockville, United States

**Background**: HIV cure research is challenged by the persistence of the replication‐competent proviral reservoir. Our hypothesis is that AGT103‐T, a gene therapy designed to protect and boost HIV‐specific immune cells, reduces this reservoir. To accurately assess this effect, we developed a modified Intact Proviral DNA Assay (IPDA) that distinguishes HIV sequences from the AGT103 lentiviral vector.

**Methods**: In our FDA‐approved Phase 1 study, HIV‐positive subjects received AGT103‐T infusions. Longitudinal peripheral blood samples were collected before and after infusion, including during analytical treatment interruptions (ATIs) that allowed the evaluation of viral rebound dynamics in a clinical setting. Our modified IPDA, using primers and probes targeting conserved HIV regions absent in the AGT103 lentiviral vector, quantified both intact and defective proviral DNA in CD4^+^ T cells.

**Results**: In all evaluable subjects (*n* = 5), our modified IPDA demonstrated a significant reduction in intact proviral DNA following AGT103‐T treatment. For example, one subject's baseline intact proviral level of approximately 1664 copies per million CD4^+^ T cells decreased to undetectable levels by 500 days post‐infusion, with suppression sustained for up to 2 years. Notably, while intact proviral DNA declined markedly, defective proviral DNA levels remained unchanged throughout the study. All subjects underwent ATI, which provided valuable insights into the suppression of viral rebound.

**Conclusions**: These early findings suggest that AGT103‐T therapy may reduce the replication‐competent HIV reservoir in people living with HIV. While promising, these results should be interpreted with caution given the study's limitations (small sample size and variability in ATI timing among participants). Additional trials with larger populations and optimized ATI timing are necessary to further evaluate the clinical efficacy of this immune‐regulatory strategy.

### OAB0105LB Promising new therapeutic option for HIV—next‐generation HIV‐1 investigational protease cleavage site (maturation) inhibitor, HRF‐10071—phase 2 results

N. Kumarasamy ^1^, B. Parthasaradhi Reddy^2^, A. Panduranga Reddy^2^, K. Ratnakar Reddy^2^, D. Shubhadeep Sinha^2^, S. Sreenivasa Chary^2^, P. Thakur^2^, D. Shashidhar Reddy^2^, T. Sravan^2^, S.K. Tripathi^3^

^1^VHS Infectious Diseases Medical Centre, CART Clinical Research Site, Chennai, India, ^2^HETERO LABS LIMITED, Hyderabad, India, ^3^Jagannath Gupta Institute of Medical Sciences & Hospital, Clinical Pharmacology, Kolkata, India

**Background**: HRF‐10071 is a new generation protease cleavage site inhibitors that inhibit viral maturation by blocking cleavage of the Gag capsid (CA) precursor, CA‐SP1, to mature CA protein. Dose escalating studies (single ascending dose and multiple ascending dose) were evaluated in healthy adult human participants. HRF‐10071 exposures (C_max_ and AUC) were dose proportional (linear) from 20 to 160 mg and found to be safe and well tolerable. Here, we report the outcome of the Phase 2 Proof of Concept study carried out among PLHIV.

**Methods**: Phase 2 (Proof of Concept) study conducted at 15 sites across India. This study was a double‐blind, parallel, placebo‐controlled study, which evaluated antiviral effect, safety and tolerability of HRF‐10071 20, 30, 40 and 80 mg dose levels over 14 days of treatment period.

**Results**: Overall, 30 people (25 males and 5 females) with baseline BMI of 22.4−25.3 kg/m^2^, median viral load of 31,923–89,632 copies/ml and CD4 count of 397−495 cells/mm^3^ enrolled. HIV RNA levels decreased significantly from Day 7 onwards in all dose levels. HRF‐10071 doses 20, 30, 40 and 80 mg have shown −0.98, −1.56, −1.84 and −1.76 HIV RNA Log10 change from baseline to Day 14, respectively. The change in HIV RNA levels was statistically significant compared with placebo. Pharmacokinetic profile in study participants is similar to that of healthy people (dose proportional) with steady state reaching between 7 and 10 days on QD regimen. Overall, 10 adverse events (nine grade 1 and one grade 2) reported in this study were not related to tested compound. No grade 3 or 4 or severe AEs reported. No patient discontinued due to AE/SAE.

**Conclusions**: HRF‐10071 is a novel potent protease cleavage site inhibitor. Study results have shown significant viral load reductions. This could be a future treatment option for people living with HIV along with other anti‐retroviral agents.[Fig jia226518-fig-0027]

**Figure 1 jia226518-fig-0027:**
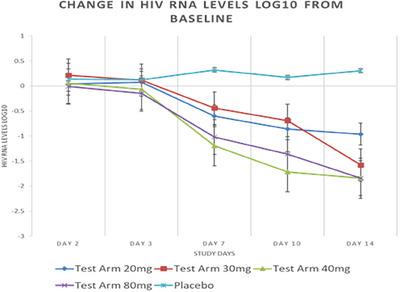
OAB0105LB

### OAB0106LB Systematic review and meta‐analysis of the efficacy of intermittent antiretroviral therapy dosing: a crisis response to the sudden cuts in USAID and PEPFAR funding

A. Hill^1^, C. Fairhead ^2^, S. Manalu^3^, F. Venter^4^, S. Collins^5^, R. Landman^6^, A. Pozniak^7^, E. Martinez^8,9^

^1^University of Liverpool, Department of Pharmacology and Therapeutics, Liverpool, United Kingdom, ^2^Royal Free Hospital, Acute Medicine, London, United Kingdom, ^3^Universitas Indonesia, Oxford University Clinical Research Unit Indonesia, Faculty of Medicine, Jakarta, Indonesia, ^4^Wits Ezintsha, University of the Witwatersrand Faculty of Health Sciences, Johannesburg, South Africa, ^5^HIV i‐Base, London, United Kingdom, ^6^Assistance Publique – Hôpitaux de Paris, Hospital Bichat service des maladies infectieuses et tropicales, Paris, France, ^7^Chelsea and Westminster Hospital NHS Foundation Trust, London, United Kingdom, ^8^Hospital Clínic de Barcelona, Barcelona, Spain, ^9^Instituto de Salud Carlos III, CIBER de Enfermedades Infecciosas (CIBERINFEC), Madrid, Spain

**Background**: Antiretroviral therapy (ART) shortages due to sudden, severe USAID and PEPFAR funding cuts are predicted to cause millions of HIV‐related deaths and new acquisitions. We assessed the efficacy of intermittent ART dosing, which could extend supplies and mitigate this crisis.

**Methods**: We systematically reviewed PubMed, MEDLINE and ClinicalTrials.Gov. Randomized controlled trials (RCTs) comparing intermittent triple ART taken 3–6 days‐per‐week versus daily ART in people with HIV‐1 of any age were included. Studies with high risk of bias were excluded. Forty‐eight‐week efficacy (HIV viral load [VL] > 50 copies/ml) was meta‐analysed. Highly sensitive VL; treatment‐emergent drug resistance and inflammation (interleukin‐6, d‐dimer and highly sensitive C‐reactive protein) were descriptively analysed. This was supplemented by a rapid scoping review of observational studies.

**Results**: Seven RCTs were included; three evaluated INSTI‐inclusive regimens. In total, 1286 virologically supressed non‐pregnant people with HIV without hepatitis B, with high CD4 counts and no virologic failure or drug resistance history, were included. RCTs investigated 3, 4 and 5 days‐per‐week intermittent ART. There was no difference in efficacy (risk difference −0.00; 95% CI −0.02 to 0.02; p = 0.86): overall 21/643 (3%) experienced HIV RNA > 50 copies/ml with intermittent ART versus 22/643 (3%) with daily ART. Viraemia was typically low level, with re‐suppression after adherence counselling and/or return to daily dosing. Where evaluated, there was no difference in highly sensitive VL or inflammation markers, and satisfaction with intermittent dosing was high. Overall, 9/14 (64%) successfully genotyped participants with VL > 50 copies/ml had treatment‐emergent resistance with intermittent ART versus 12/16 (75%) with daily ART, predominantly in lower‐genetic‐barrier regimens. Five observational studies of 3–6 days‐per‐week triple ART were identified; HIV VL > 50 copies/ml occurred in 6/374 (2%) participants at 48 weeks.

**Conclusions**: For countries with urgent drug shortages, evidence supports intermittent dosing 4−5 days‐per‐week to extend ART supplies, with clinical benefits for people with HIV and continued population benefits of minimizing HIV transmission.[Table jia226518-tbl-0018]

**Table 1 jia226518-tbl-0018:** OAB0106LB: Summary of efficacy (assessed through HIV RNA > 50 copies/ml at 48 weeks) and treatment‐emergent drug resistance in randomized controlled trials of intermittent versus daily triple antiretroviral therapy

RCT	Strategy	ART regimen	HIV RNA > 50 copies/ml; intermittent, *n*/*N* (%)	HIV RNA > 50 copies/ml; daily, *n*/*N* (%)	Resistance**; intermittent, *n*/*N*	Resistance**; daily, *n*/*N*
BREATHER	5 on, 2 off	NNRTI	6/99 (6%)	7/100 (7%)	2/3	5/6
Reynolds et al.***	5 on, 2 off	NNRTI	5/57 (9%)	9/56 (16%)	4/4	5/7
Sun et al.	5 on, 2 off	B/F/TAF	0/30 (0%)	1/31 (3%)	NA	–
QUATUOR	4 on, 3 off	Triple ART	6/318 (2%)	4/318 (1%)	3/6	2/3
ATAD	Alternate	NNRTI	3/99 (3%)	1/98 (1%)	0/1	–
A‐TRI‐WEEK	3 on, 4 off	NNRTI	0/30 (0%)	0/30 (0%)	NA	NA
BETAF‐RED	3 on, 4 off	B/F/TAF	1/10 (10%)	0/9 (0%)	–	NA

Abbreviations: ART, antiretroviral therapy; NNRTI, non‐nucleoside reverse transcriptase inhibitor; B/F/TAF, bictegravir/emtricitabine/tenofovir alafenamide; NA, not applicable; −, not reported.

*When 4 or 5 days per week dosing is employed, continuous ART is given for these days and then no ART for the rest of the week. When 3 days‐per‐week dosing is used, the days off ART are not continuous but are instead alternate.

**Resistance = treatment‐emergent drug resistance. Resistance n/N = number of individuals with mutations conferring resistance to study drugs/number of reported successfully genotyped individuals with HIV RNA > 50 copies/ml.

*** Reynolds et al.: The primary endpoint was changed during the study from < 50 copies/ml to < 400 copies/ml to eliminate the inclusion of low‐level viral blips.

### OAB0206LB A5391: a randomized multicentre 3‐arm controlled trial for people with obesity on integrase inhibitors and tenofovir alafenamide switching to doravirine, with or without tenofovir disoproxil fumarate (The DO‐IT Trial)

J. Koethe ^1^, J. Lake^2^, A. Kantor^3^, L. Smeaton^3^, K. Erlandson^4^, L. Moran^5^, P. Belaunzaran‐Zamudio^6^, A. Landay^7^, P. Debroy^2^, J. Bennet^8^, J. O'Halloran^9^, W. Min Han^10^, O. Alli^6^, M. Leonard^1^, R. Gulick^11^, for the ACTG 5391 (Do IT) Trial Team

^1^Vanderbilt University Medical Center, Nashville, United States, ^2^University of Texas Health Science Center at Houston, Houston, United States, ^3^Harvard T.H. Chan School of Public Health, Boston, United States, ^4^University of Colorado, Aurora, United States, ^5^ACTG Network Coordinating Center, Bethesda, United States, ^6^National Institutes of Health, Bethesda, United States, ^7^University of Texas Medical Branch at Galveston, Galveston, United States, ^8^University of the Witwatersrand, Johannesburg, South Africa, ^9^St. Vincent's University Hospital, Dublin, Ireland, ^10^HIV‐NAT, Thai Red Cross AIDS Research Centre, Bangkok, Thailand, ^11^Weill Cornell Medical College, New York, United States

**Background**: Excessive weight gain has been associated with integrase inhibitors (INSTI) and tenofovir alafenamide (TAF). ACTG A5391 evaluated whether switching to doravirine (DOR; a non‐nucleoside reverse transcriptase inhibitor) with or without a switch to tenofovir disoproxil fumarate (TDF) would alter weight in people with HIV and obesity on INSTI+TAF regimens.

**Methods**: A phase IV, 48‐week, 3‐arm, open‐label, multicentre randomized trial to assess whether switch from an INSTI (bictegravir, dolutegravir or raltegravir) with TAF/emtricitabine (TAF/FTC) to either DOR+TAF/FTC or DOR+TDF/FTC results in a ≥5% point between‐group difference in body weight change (a clinically relevant effect), compared to continuing current INSTI+TAF/FTC. We used linear regression, adjusting for sex and race, to estimate treatment effects.

**Results**: One hundred and forty‐five participants in the United States were randomized to change to DOR+TAF/FTC (*n* = 47), DOR+TDF/FTC (*n* = 49) or continue current treatment (*n* = 49). At entry, median age was 49 years; BMI 34.9 kg/m^2^; time on INSTI+TAF/FTC 3.4 years; 49% were female, 53% Black and 18% Hispanic/Latino. Estimated mean change in weight at 48 weeks was −0.47% (95% confidence interval [CI]: −2.09, 1.14) for DOR+TAF/FTC, −2.73% (−4.22, −1.23) for DOR+TDF/FTC and −1.84% (−3.37, −0.30) for continued INSTI+TAF/FTC. The DOR versus INSTI (both with TAF/FTC) estimated mean difference was +1.36% points (*p* = 0.23), and the DOR+TDF/FTC versus INSTI+TAF/FTC estimated mean difference was −0.89% points (*p* = 0.41; Figure 1). Weight treatment difference 97.5% CI limits were < 5% points. There was no evidence that treatment differences varied by subgroups, including by race or sex. For hip bone mineral density, estimated mean differences were +0.78% points (*p* = 0.19) and −0.64% points (*p* = 0.27), respectively. The observed median change in creatinine clearance was 4.9% in DOR+TAF/FTC, 7.4% in DOR+TDF/FTC and 0.4% in INSTI+TAF/FTC.

**Figure 1:**
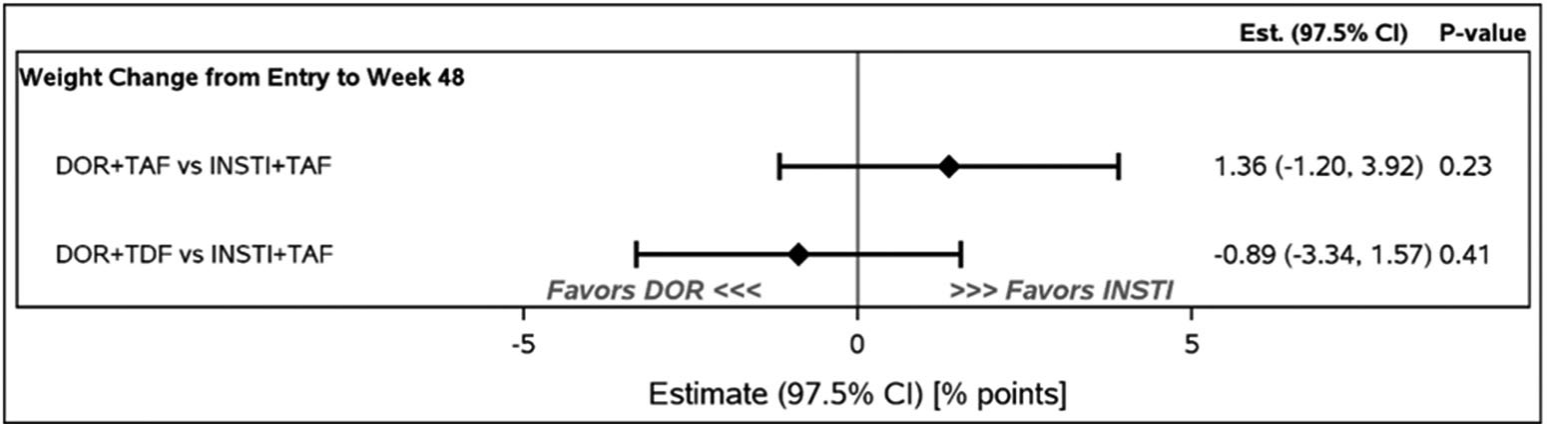
**OAB0206LB: Treatment difference in body weight change between DOR and INSTI arms at 48 weeks**.

**Conclusions**: In people with HIV and obesity, switching from an INSTI+TAF/FTC regimen to doravirine/FTC with either TAF or TDF did not result in substantial weight change at 1 year, including among women and Blacks.

### OAB0306LB PedMAb1 trial: safety of two broadly neutralizing antibodies, CAP256V2LS and VRC07‐523LS, administered singly or concurrently at birth and 3 months to breastfeeding HIV‐1‐exposed neonates and infants born without HIV

G. Scarlatti ^1^, E. Clarence^2^, T. Ramraj^2^, L. Naidoo^2^, B. Daniels^2^, T. Chetty^2^, R. Dassaye^2^, N.K. Ngandu^2^, S. Buthelezi^2^, G. Mjwara^2^, M. Reddy^2^, Q. September^2^, T. Reddy^2^, Q. Ndlangamandla^2^, N. Jeenarain^2^, S. Ganesh^2^, A. Ramjeith^2^, Y. Cazaubon^3^, J.P. Moles^3^, P. Van de Perre^3^, T. Tylleskär^4^, P.L. Moore^5,6,7^, S. Balla^5,6^, N.N. Mkhize^5,6^, C. Crowther^5,6^, M. Tolazzi^1^, S. Dispinseri^1^, A. Goga^2,8^, on behalf of the PedMAb1 clinical trial team

^1^IRCCS Ospedale San Raffaele srl, Viral Evolution and Transmission Unit, Milan, Italy, ^2^South African Medical Research Council, Durban, South Africa, ^3^INSERM, University Montpellier, Montpellier, France, ^4^University of Bergen, Bergen, Norway, ^5^University of the Witwatersrand, SAMRC Antibody Immunity Research Unit, School of Pathology, Faculty of Health Sciences, Johannesburg, South Africa, ^6^National Institute for Communicable Diseases, Centre for HIV and STIs, division of the National Health Laboratory Service, Johannesburg, South Africa, ^7^University of KwaZulu‐Natal, Centre for the AIDS Programme of Research in South Africa (CAPRISA), Durban, South Africa, ^8^University of Pretoria, Department of Paediatrics and Child Health, Pretoria, South Africa

**Background**: PedMAb aims to develop a promising strategy using broadly neutralizing antibodies (bNAbs) to interrupt postnatal HIV transmission via breastmilk, which still contributes significantly to paediatric HIV infections in high HIV‐prevalence settings. We report on safety and pharmacokinetic (PK) data of two anti‐HIV‐1 bNAbs, CAP256V2LS and VRC07‐523LS, administered concurrently to neonates for the first time at delivery and 3 months.

**Methods**: Forty HIV‐exposed infants born without HIV (HEI) received escalating doses of either CAP256V2LS (arms 1, 2, 3: 5, 10 or 20 mg/kg) or VRC07‐523LS (arms 4, 5: 20 or 30 mg/kg) subcutaneously (SC), within 96 hours from birth. Following a safety assessment and PK population analysis, eight infants (arm 6/6b) received a fixed dose of CAP256V2LS (60 mg) and VRC07‐523LS (90 mg) within 96 hours (dose 1) and 120 mg of either bNAb at 3 months (dose 2). Infants were observed for 2–4 hours or 1 hour after each dose in arm 6/6b, and seen at days 1, 3, 14 and 28, then monthly until end of follow‐up. Internal and external safety committees reviewed safety and PK data monthly and biannually, respectively.

**Results**: Reactogenicities at 4 hours and over the first 3 days post‐dose were observed in 5/24 and 7/16 infants receiving CAP256V2LS or VRC07‐523LS, respectively; and in arm 6/6b in 4/8 and 1/8 infants after dose 1 and 2, respectively. One hundred and seventeen adverse events (AEs) were reported in 24 infants receiving CAP256V2LS, 82 in 16 infants receiving VRC07‐523LS; 43 and 54 AEs were reported in eight infants of arm 6/6b after dose 1 and 2, respectively. Overall, AEs were grade 1 or 2, except for five grade 3 and two grade 4. In arm 6/6b, possibly related AEs were low absolute neutrophil count (*n* = 5) and anaemia (*n* = 1), and one probably related case of irritability. All infants were clinically well, and AEs resolved spontaneously.

**Conclusions**: CAP256V2LS and VRC07‐523LS are safe when administered alone and in combination to HEI. Phase 2/3 trials are needed to investigate the efficacy of bNAbs to interrupt vertical HIV transmission, thus increasing the armamentarium against postnatal HIV transmission and creating an HIV‐free paediatric population. Progress has been hampered by current global development.

### OAB0406LB Foetal growth in pregnant women living with HIV: longitudinal analysis of serial ultrasound measurements in South Africa

D.N.K. Darji ^1^, I. INTERBIO‐21st Consortium^2,3^, S. Norris^4^, E. Ohuma^5^, J. Hemelaar^1^

^1^University of Oxford, Nuffield Department of Population Health, Oxford, United Kingdom, ^2^Oxford Maternal & Perinatal Health Institute, Green Templeton College, Oxford, United Kingdom, ^3^University of Oxford, Nuffield Department of Women's & Reproductive Health, Oxford, United Kingdom, ^4^University of Witwatersrand, MRC‐Wits Developmental Pathways for Health Research Unit, Johannesburg, South Africa, ^5^London School of Tropical Medicine and Hygiene, c Centre for Maternal, Adolescent, Reproductive, and Child Health, London, United Kingdom

**Background**: Pregnant women with HIV (PWHIV) are at increased risk of delivering babies who are small for gestational age (SGA). We aimed to determine the antenatal foetal growth trajectories of pregnant women with HIV (PWHIV), compared to HIV‐negative women.

**Methods**: In a prospective pregnancy cohort study in Soweto, South Africa, in 2013–2016, serial ultrasound measurements of head circumference (HC), biparietal diameter (BPD), abdominal circumference (AC) and femur length (FL). Multivariable linear mixed effects models were used to estimate differences in mean foetal growth measures and mean foetal growth velocity increments according to maternal HIV status. Z‐scores and centiles for all parameters were calculated according to the INTERGROWTH‐21st standards for foetal growth. Multivariable mixed effects logistic regression was used to examine the association of maternal HIV acquisition with in‐utero SGA and very SGA.

**Results**: Ultrasound measurements of 228 PWHIV receiving ART and 384 HIV‐negative pregnant women, with a median of five antenatal ultrasound scans per women, were analysed. There were no significant differences in mean foetal growth measures and growth velocity increments between foetuses of PWHIV and HIV‐negative women. The prevalence of SGA ranged between 14.2−26.4% for PWHIV and 18.5−24.1% for HIV‐negative pregnant women during different gestation windows. There was no significant association between maternal HIV acquisition and in‐utero SGA or VSGA.

**Conclusions**: Maternal HIV acquisition treated with ART was not associated with altered foetal growth, foetal growth velocity or in‐utero SGA, compared to HIV‐negative women. Our findings support international clinical guidelines recommending ART for PWHIV to improve maternal health and reduce vertical HIV transmission.1, 2

**Figure 1:**
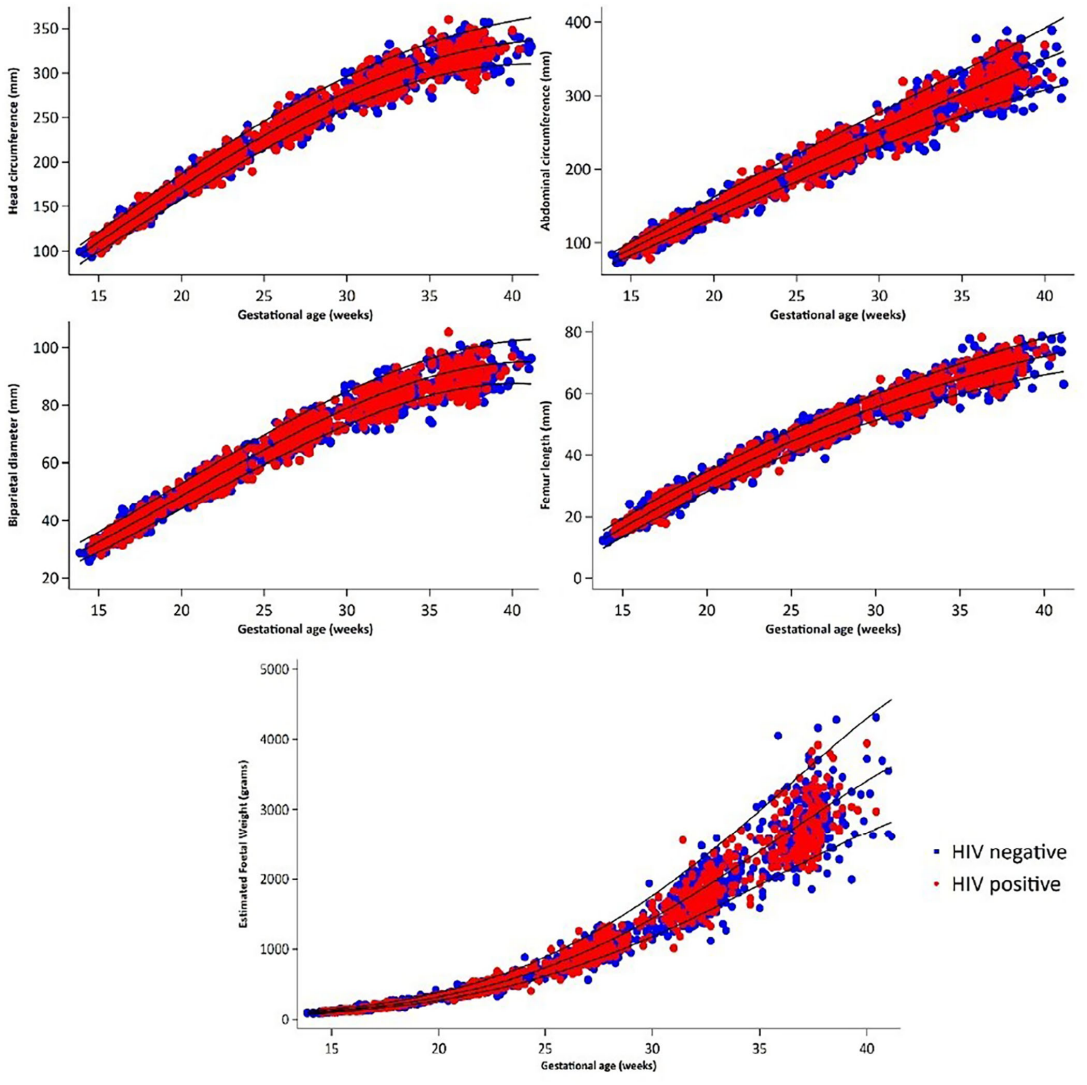
OAB0406LB

**Figure 2:**
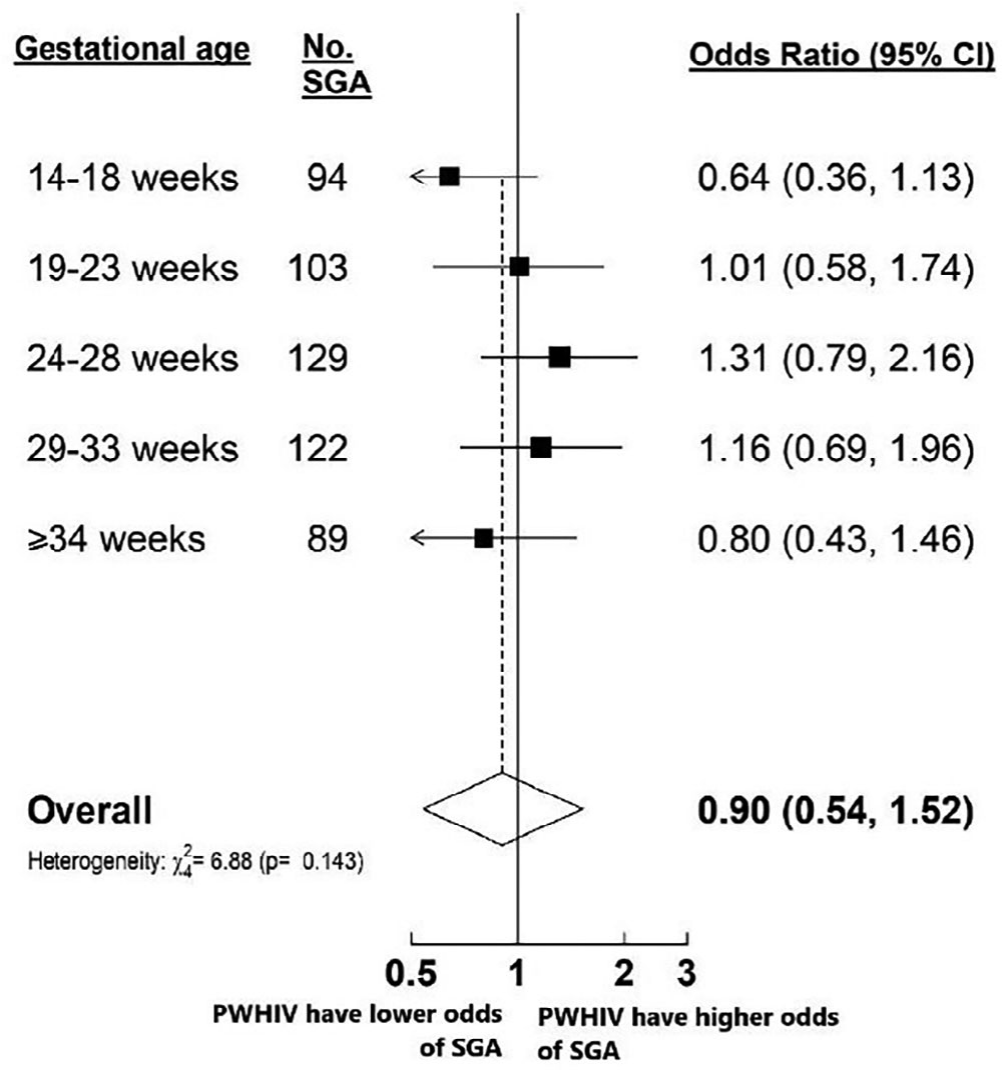
OAB0406LB

### OAB0506LB Inpatient initiation of TB preventive therapy with 1 month of isoniazid and rifapentine for adults with advanced HIV disease and cryptococcal meningitis: results of the IMPROVE randomized controlled trial

J. Ellis ^1,2^, G. Hale^2^, L.J. Nsangi^2^, A. Wele^3^, E. Kigozi^4^, J. Gakuru^2^, E. Kagimu^2^, T. Mugabi^2^, S. Namombwe^2^, S. Kimuda^2^, F. Ssekindi^2^, J.F. Ndyetukira^2^, A. Sadiq^2^, A. Tukundane^2^, W. Bakka^2^, T.S. Harrison^5,6^, E. Mande^2^, C. Muzoora^4^, D.A.J. Moore^1^, D.B. Meya^2,7^, K. Fielding^8^, D.R. Boulware^7^, J.N. Jarvis^1^

^1^London School of Hygiene and Tropical Medicine (LSHTM), Clinical Research Department, London, United Kingdom, ^2^Infectious Diseases Institute, Kampala, Uganda, ^3^University of Minnesota, Division of Biostatistics and Health Data Science, School of Public Health, Minneapolis, United States, ^4^Mbarara University of Science and Technology, Department of Medicine, Mbarara, Uganda, ^5^City St George's University of London, Institute for Infection and Immunity, London, United Kingdom, ^6^University of Exeter, MRC Centre for Medical Mycology, Exeter, United Kingdom, ^7^University of Minnesota, Division of Infectious Diseases and International Medicine, Department of Medicine, Minneapolis, United States, ^8^London School of Hygiene and Tropical Medicine (LSHTM), Department of Infectious Disease Epidemiology and International Health, London, United Kingdom

**Background**: Tuberculosis preventive therapy (TPT) coverage for persons with advanced HIV disease is poor. Due to advanced immunosuppression, adults with cryptococcal meningitis have 12‐month mortality following hospital discharge up to 78%; tuberculosis (TB) is common and preventable, and likely contributes to poor patient outcomes. Innovative delivery strategies to increase TPT provision and uptake are needed for this high‐risk population.

**Methods**: In this phase‐3, multi‐site, open‐label, non‐inferiority randomized controlled strategy trial, we randomized adults with HIV‐associated cryptococcal meningitis and no evidence of active TB disease in a 1:1 ratio to inpatient initiation of 1HP (1 month of daily rifapentine plus isoniazid) prior to hospital discharge, or outpatient initiation at 6 weeks (standard of care). The primary end point was TB disease‐free survival and 1HP treatment completion at 18 weeks, based on a 15% non‐inferiority margin and one‐sided 95% confidence interval (CI).

**Results**: Of 205 randomized participants, 69.9% (72/103) with inpatient 1HP initiation had TB disease‐free survival and 1HP treatment completion at 18 weeks versus 61.8% (63/102) in the outpatient 1HP arm (adjusted risk difference 7.1% 95% CI −5.9% to 20.1%). No significant differences existed in any secondary endpoints when comparing inpatient versus outpatient 1HP initiation, including: grade 3 or higher and serious adverse events (risk difference −2.4% 95% CI −15.8% to 11.0%); drug‐induced liver injury (risk difference 0.4% 95% CI −7.9% to 8.7%); incident TB disease (risk difference −5.4%, 95% CI −13.4% to 2.6%); and 18‐week survival (adjusted risk difference 1.9%, 95% CI −8.0% to 11.8%).

**Conclusions**: 1HP initiation prior to hospital discharge was non‐inferior to outpatient initiation among adults with advanced HIV disease and cryptococcosis. These data indicate that following the exclusion of active TB disease, inpatient 1HP initiation is feasible and safe.[Fig jia226518-fig-0032]

**Figure 1 jia226518-fig-0032:**
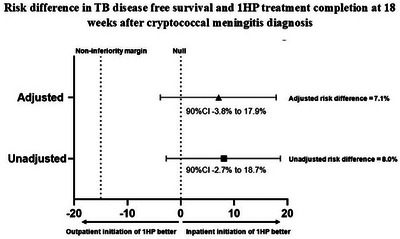
OAB0506LB

### OAC0106LB PrEP provision in bars versus primary care: a cluster RCT among 780 female bar workers in Dar es Salaam, Tanzania

W. Akyoo ^1,2^, V.K. Nguyen^2,3^, H. Goymann^2^, D. Kamori^4^, I. Mosha^1^, A.J. Njwayo^5^, A. Jahn^2^, M. Gandhi^6^, H. Okochi^6^, J. Knox^7,8,9^, J.J. Chebet^10^, D. Spiegelman^11^, D. Barnhart^12^, G. Harling^13,14,15^, R. Mpembeni^16^, T. Bärnighausen^2,14,17^

^1^Muhimbili University of Health and Allied Sciences, School of Public Health and Social Sciences, Department of Behavioural Sciences, Dar es Salaam, Tanzania, the United Republic of, ^2^Heidelberg University, University Hospital and Medical Faculty, Institute of Global Health, Heidelberg, Germany, ^3^School of Public Health, Imperial College London, Department of Infectious Diseases Epidemiology, London, United Kingdom, ^4^Muhimbili University of Health and Allied Sciences, Department of Microbiology and Immunology, Dar es Salaam, Tanzania, the United Republic of, ^5^Ubungo Municipal Council, Dar es Salaam, Tanzania, the United Republic of, ^6^University of California, San Francisco (UCSF), Hair Analytical Laboratory, Division of HIV, Infectious Diseases, and Global Medicine, Department of Medicine, San Francisco, United States, ^7^Columbia University Irving Medical Center, Department of Psychiatry, New York, United States, ^8^New York State Psychiatric Institute and Columbia University, HIV Center for Clinical and Behavioral Studies, New York, United States, ^9^Columbia University Mailman School of Public Health, Department of Sociomedical Sciences, New York, United States, ^10^University of Arizona, Department of Health Promotion Sciences, Mel and Enid Zuckerman College of Public Health, Tucson, United States, ^11^Yale School of Public Health, Center for Methods in Implementation and Prevention Science (CMIPS), Department of Biostatistics, New Haven, United States, ^12^Laterite, Nairobi, Kenya, ^13^University College London, Institute for Global Health, London, United Kingdom, ^14^Africa Health Research Institute (AHRI), Somhkele and Durban, South Africa, ^15^University of the Witwatersrand, MRC/Wits Rural Public Health and Health Transitions Research Unit (Agincourt), School of Public Health, Faculty of Health Science, Johannesburg, South Africa, ^16^Muhimbili University of Health and Allied Sciences, School of Public Health and Social Sciences, Department of Epidemiology and Biostatistics, Dar es Salaam, Tanzania, the United Republic of, ^17^Harvard University, Harvard T H Chan School of Public Health, Boston MA, United States

**Background**: In many countries in Africa, adolescent girls and young women (AGYW) are disproportionally affected by HIV and may thus benefit from HIV pre‐exposure prophylaxis (PrEP). Female bar workers (FBWs) are AGYW who are at particularly high risk of HIV acquisition, because they often engage in transactional sex. However, FBWs commonly lack easy access to PrEP because of their work schedules, high mobility and stigma. We report primary outcomes from a cluster‐randomized controlled trial (cRCT) of bar‐based PrEP versus the standard‐of‐care, which is primary care‐based PrEP for FBWs in Dar es Salaam, Tanzania.

**Methods**: One hundred and twelve bars were cluster‐randomized (cR1) to receive PrEP promotion, eligibility screening, initiation and monthly refills in the bars versus PrEP promotion in the bars but all other PrEP functions in primary care clinics, the current standard‐of‐care in Tanzania. All FBWs who initiated PrEP were further individually randomized (R2) to receive versus not receive adherence support through a PrEP champion. We followed up with trial participants monthly over 6 months, including urine‐based tenofovir disoproxil fumarate (TDF) testing at months 2 and 6. We report the proportion of FBWs who took up, adhered to, were lost to follow‐up and dropped out of PrEP in each trial arm. We used modified‐Poisson regression to calculate risk ratios (RR) and 95% confidence intervals (CI). The trial was registered with the German Clinical Trials Register (DRKS00018101).

**Results**: Of the 1017 FBWs in the participating bars, 780 agreed to be screened and 439 were eligible and consented to participate in the trial. When offered PrEP, initiation was 75% in the bar‐based versus 27% in the primary care‐based arm (RR 2.79, 95% CI: 2.26−3.47). Adherence, loss to follow‐up and drop‐out were not significantly different between the trial arms over the follow‐up period of 6 months.

**Conclusions**: Bar‐based PrEP provision for FBWs substantially boosted PrEP uptake. Once FBWs received care, their adherence and retention did not differ between the trial arms. Offering PrEP in bars, and similar workplaces, should be considered as a policy in Tanzania and other countries in Africa to increase PrEP uptake among key populations in the HIV response.

### OAC0206LB Evaluating CAB‐LA concentration and breastmilk transfer in postpartum PrEP: results from the Tshireletso PK substudy

M. Yoseph ^1^, J. Momper^2^, E. Shava^1^, B. Oguttu^1^, K. Shambira^1^, S. Silarszka^3^, T. Mohammed^1^, S. Moyo^1^, J. Makhema^1^, G. Masheto^1^, C. Morroni^1,4^, S. Lockman^1,5^, R. Shapiro^1,6^, E. Capparelli^2^, R. Zash^1,7^

^1^Botswana Harvard Health Partnership, Gaborone, Botswana, ^2^University of California San Diego, San Diego, United States, ^3^Harvard University, Boston, United States, ^4^University of Edinburgh, Edinburgh, United Kingdom, ^5^Brigham and Women's Hospital, Boston, United States, ^6^Harvard TH Chan School of Public Health, Boston, United States, ^7^Beth Israel Deaconess Medical Center, Boston, United States

**Background**: Initiating long‐acting cabotegravir (CAB‐LA) for pre‐exposure prophylaxis (PrEP) on the maternity ward after delivery is an efficient strategy to engage young women and prevent HIV in mothers and breastfeeding infants. However, there is a paucity of data on the pharmacokinetics (PK) of CAB‐LA started immediately post‐partum and infant exposure through breastfeeding.

**Methods**: “Tshireletso” evaluated the implementation and safety of CAB‐LA PrEP within 14 days of delivery. CAB‐LA was injected IM at enrolment, 1 month, then every 2 months. Samples were collected just before the 1‐month injection (1m), 1 week later (1m+1wk), just before the 5‐month injection (5m) and 1 week later (5m+1m). Plasma and whole breastmilk concentrations of CAB were measured using a liquid chromatography‐tandem mass spectrometry (LC‐MS/MS) assay. Relative infant dose (RID) (estimated infant dose via breastmilk [mg/kg/day]/adult dose [mg/kg/day]) was calculated using average milk intake of 150 ml/kg/day, observed breastmilk concentrations, adult IM CAB dose (600 mg q56d) and measured maternal and infant weights.

**Results**: Data from 27 lactating women and their breastfed infants with all specimens from ≥two time points were included. Median maternal plasma, breastmilk and infant plasma CAB concentrations at 1m, 1 m+1wk, 5m and 5 m+1wk were highly variable and increased over time (Table 1). Seventy‐four percent of maternal CAB concentrations were > 4x PA‐IC90 (664 ng/ml) at 1m, and all but one (from 1m+1wk) exceeded this threshold at all other time points. Median (IQR) breastmilk to plasma ratio of CAB was 1.4% (1.1%, 1.9%). Infant CAB concentrations were greater than 1x PA‐IC90 (166 ng/ml) in 13.7% of samples. The overall median (IQR) RID was 2.5% (1.6%, 4.5%).

**Table 1:** OAC0206LB: Cabotegravir concentrations in maternal, infant and breastmilk samples over time after initiation of maternal CAB‐LA injections for PrEP

	1 month (trough) median (range)	1 month ± 1 week (peak) median (range)	5 month (trough) median (range)	5 month ± 1 week (peak) median (range)
Maternal plasma CAB concentration (ng/ml)	941 (322, 6980)	1980 (580, 9820)	1875 (1030, 3050)	2480 (1800, 7080)
Breastmilk CAB concentration (ng/ml) *Quantitative limit (QL) for breastmilk = < 0.977 ng/ml	14.6 (2.5, 89)	29.8 (6.7, 143)	27.4 (10.8, 129)	36.1 (17.2, 108)
Infant plasma CAB concentration (ng/ml) *Quantitative limit (QL) for plasma ≤39 ng/ml	33.2 (16 below QL, 638)	69.2 (5 below QL, 933)	85.3 (5 below QL, 226)	92.1 (1 below QL, 365)
Relative infant dose of CAB (% of maternal dose, mg/kg/day)	1.6% (0.2%, 6.3%)	2.8% (0.6%, 10.1%)	2.2% (1.1%, 9.5%)	3.3% (1.7%, 7.7%)

**Conclusions**: CAB reached protective levels within 1 week after the second dose in nearly all breastfeeding mothers. Breastmilk concentrations of CAB‐LA were low, with estimated median RID below the < 10% threshold, and all infant levels less than 1000 ng/ml. These findings support the safety of CAB‐LA use during breastfeeding, reinforcing its potential as a viable PrEP strategy in the postpartum period.

### OAC0406LB The impact of long‐acting lenacapavir on adolescent girls and young women in Kenya: a mathematical modelling study

S. B. Klein ^1^, C. L. Hathaway^1,2^, J. Heitner^1^, M. Onono^3^, N.R. Mugo^4,5^, R. V. Barnabas^1,6,7^

^1^Massachusetts General Hospital, Division of Infectious Diseases, Boston, United States, ^2^University of Washington, School of Medicine, Seattle, United States, ^3^Kenya Medical Research Institute, Center for Microbiology Research, Kisumu, Kenya, ^4^University of Washington, Department of Global Health, Seattle, United States, ^5^Kenya Medical Research Institute, Center for Clinical Research, Nairobi, Kenya, ^6^Harvard Medical School, Department of Medicine, Boston, United States, ^7^Harvard School of Public Health, Department of Epidemiology, Boston, United States

**Background**: Current oral pre‐exposure prophylaxis (PrEP) for HIV has not achieved its full potential, largely due to the challenges with adherence and consistent use. To improve uptake and effectiveness, alternative delivery methods are needed. Lenacapavir, a long‐acting injectable administered twice yearly, offers a promising solution. We used modelling to assess the potential impact of lenacapavir on HIV outcomes among high‐risk adolescent girls and young women (AGYW) in Kenya.

**Methods**: We used a deterministic transmission‐dynamic compartmental model of HIV infection calibrated to Kenya to simulate PrEP implementation scenarios. Oral PrEP, introduced in 2018, was assumed to be used exclusively by HIV‐negative girls aged 15–24 at high risk (≥5 partners/year) reaching 4.6% coverage by 2025. Starting in 2026, we assumed a national switch in Kenya to long‐acting injectable lenacapavir with five possible trajectories: (1) maintaining 2025 coverage levels, or increasing to (2) 15%, (3) 30%, (4) 45%, or (5) 57% coverage by 2030. We also modelled a comparator scenario in which there is no switch to lenacapavir and no scale‐up beyond 2025 oral PrEP coverage levels. Oral PrEP was assumed to be 75% effective and lenacapavir 100% effective in preventing HIV infection. We assessed the median and interquartile range (IQR) of new HIV cases and cumulative cases averted over 30 years across the 25 best‐fitting parameter sets.

**Results**: The greatest impact of lenacapavir among AGYW was observed at higher coverage levels. At 57% coverage, lenacapavir was estimated to avert 11,904.93 (9293.73−17,027.33) new HIV cases, representing an 18.82% (18.34%–19.62%) reduction compared to oral PrEP. However, switching to lenacapavir alone without scaling up coverage had minimal effect—at 4.6% coverage, the percent reduction in HIV acquisition was only 0.58% (0.57%–0.61%). A modest increase to 15% coverage averted 2974.52 (2322.97−4274.46) HIV cases, while scaling to 30% averted approximately 6445.65 (5036.58−9248.84) cases.

**Conclusions**: These results emphasize the importance of not only transitioning to long‐acting PrEP but also expanding coverage to achieve meaningful reductions in HIV incidence among AGYW. Lenacapavir has the potential to significantly reduce the burden of HIV among adolescent girls and young women if coverage can be effectively scaled up.

### OAD0106LB Effectiveness of the TENDAI integrated therapy for depression and adherence to HIV medication delivered by lay adherence counsellors in Zimbabwe: results from a randomized controlled trial

T. Bere ^1^, C. O'Cleirigh^2^, K. Goldsmith^3^, P. Nyamayaro^1^, R. Jopling^4^, S. Cross^3^, S. Marquez^2^, P. Ganguli^4^, A. Stanton^5^, D. Gudyanga^1^, J. Lee^2^, D. Chibanda^1^, S. Safren^6^, B. Barrett^4^, W. Mangezi^1^, M. Abas^7^

^1^University of Zimbabwe, Mental Health Department, Harare, Zimbabwe, ^2^Massachusetts General Hospital, Behavioral Medicine Program, Department of Psychiatry, Boston, United States, ^3^King's College London, Department of Biostatistics & Health Informatics, London, United Kingdom, ^4^King's College London, Health Service and Population Research Department, London, United Kingdom, ^5^Boston University, Dept. of Psychological & Brain Sciences, Boston, United States, ^6^University of Miami, Center for HIV and Research on Mental Health, Miami, United States, ^7^King's College London, Centre for Global Mental Health, London, United Kingdom

**Background**: Depression increases the risk that people will have poor adherence to antiretroviral therapy (ART) and HIV viral non‐suppression. It is unknown if psychological interventions delivered by lay counsellors can improve mental health and HIV outcomes. We conducted a 2‐arm parallel‐group randomized controlled trial testing whether the TENDAI psychological intervention was effective for addressing viral non‐suppression, depression and ART adherence.

**Methods**: Two hundred and eighty adults living with HIV aged ≥18 years with probable depression (Patient Health Questionnaire, PHQ‐9, ≥10) and viral non‐suppression (≥1000 HIV copies per ml) were recruited from rural and urban clinics in Zimbabwe and randomized. People in the intervention arm received TENDAI, a culturally adapted 6‐session problem‐solving and motivational intervention, combining depression treatment with focus on adherence, delivered by lay adherence counsellors. People in the control arm received enhanced usual care (EUC). TENDAI versus EUC comparisons were estimated using logistic regression (viral suppression), linear mixed‐effects models (PHQ‐9 and Wilson self‐report adherence) and generalized estimating equations (GEE) logistic regression (second‐line ART use, a post hoc outcome). Cost‐effectiveness was estimated using quality‐adjusted life‐years (QALYs) derived from the EQ‐5D‐5L.

**Results**: Retention at 12 months was 91%. PHQ‐9 depression scores were 4.3 points lower in the TENDAI arm compared to EUC at 4 months (95% CI −5.4, −3.2, *p* < 0.001) and 2.2 points lower at 12 months (95% CI −3.3, −1.2, *p* < 0.001). Self‐reported adherence was significantly higher in the TENDAI arm at 4 months (mean difference 5.2 points, 95% CI 2.5, 7.9, *p* < 0.01) and at 12 months (mean difference 4.2 points, 95% CI 1.2, 7.2, *p* < 0.01). There was no significant difference in viral suppression between TENDAI and EUC arms. However, TENDAI participants had 70% lower odds of being prescribed second‐line ART at 12 months (OR 0.3, 95% CI 0.1, 0.7, *p* < 0.01). QALYs were significantly higher, and costs lower, in the TENDAI arm, providing evidence of cost‐effectiveness.

**Conclusions**: This is the first trial demonstrating that an intervention delivered by lay adherence counsellors achieved sustained improvements in depression symptoms and self‐reported adherence. People receiving TENDAI were more likely to remain on first‐line ART. These promising results, including favourable cost‐effectiveness, support implementing TENDAI in routine HIV care in low‐resource settings.

### OAD0206LB Redressing resource inequities and advancing the right to health: a rights‐based approach to HIV, TB and STI service access in South Africa

M. Mbasa ^1^

^1^Civil Society Forum, People Living with HIV, Johannesburg, South Africa

**Background**: Section 27(1) of the South African Constitution guarantees every person the right to access healthcare services, including reproductive health services. Yet, systemic inequities, especially in public health financing, supply chain continuity and service delivery persist as structural barriers. With 87% of the population reliant on an underfunded public system that receives less than half of the national health budget, rights violations are entrenched. This abstract presents lessons from community and civil society‐led initiatives advocating for equitable access to HIV, TB and STI services through a human rights‐based lens.

**Description**: This analysis draws on qualitative inputs from multisectoral advocacy meetings, service delivery audits and structured community dialogues conducted between 2022 and 2024 across five under‐resourced districts in South Africa. Facilitated by coalitions of civil society, healthcare users and frontline providers, the dialogues explored gaps in supply chains, viral load monitoring, medicine stockouts and systemic delays in clinical service delivery. These findings were triangulated with public expenditure data and civil society‐led shadow budget reviews, emphasizing youth, key populations and people living with HIV.

**Lessons learned**: Persistent underfunding and fragmented planning in public health have undermined HIV/TB/STI service delivery, particularly affecting the continuity of ARV supply and viral load monitoring. Disparities in healthcare financing where 51% of expenditure benefits only 14% of the population have deepened inequality. Community‐led monitoring exposed critical data gaps, while civil society advocacy pushed for greater accountability in budget allocation. Deprivation‐linked stigma further alienates vulnerable groups from accessing care. Yet, community organizations remain underfunded, threatening the sustainability of their work.

**Conclusions/Next steps**: A human rights‐based health response must include structural reforms to financing, improved transparency, equitable resource distribution and integration of deprived communities into planning processes. Scaling community‐led accountability, policy advocacy and investment in grassroots actors are essential to make rights to health not only aspirational but real. Public‐private parity in resource allocation must be central to closing the treatment gap.

### OAD0306LB Enhancing PrEP uptake through stigma reduction: results from the PrEPUp! Quality improvement collaborative in Malawi

S. Likumbo ^1^, E. Moses^2^, M. Enock^2^, G. Kawalazira^3^, Y. Kamgwira^4^, D. Kasale^4^, R. Birchard^5^, D. Hoege^6^, S. Allinder^7^, C. Holmes^7^, J. Murungu^8^, B. Agins^9^

^1^Blatyre District Health Office, Blantyre, Malawi, ^2^HEALTHQUAL, University of Carolina San Francisco, Lilongwe, Malawi, ^3^Blantyre District Health Office, Blantyre, Malawi, ^4^Malawi National Aids Commission, Blantyre, Malawi, ^5^HEALTHQUAL, University of Carolina San Francisco, San Francisco, United States, ^6^Georgetown University, Centre for Innovation in Global Health, New York, United States, ^7^Georgetown University, Centre for Innovation in Global Health, Washington DC, United States, ^8^HEALTHQUAL, University of Carolina San Francisco, Harare, Zimbabwe, ^9^HEALTHQUAL, University of Carolina San Francisco, New York, United States

**Background**: While stigma towards people living with HIV (PLHIV) is well documented, limited research addresses stigma associated with pre‐exposure prophylaxis (PrEP). As part of a quality improvement collaborative (QIC) within the Blantyre Prevention Strategy, PrEPUp! implemented provider and client assessments of PrEP‐related stigma and identified drivers of low uptake to inform targeted quality improvement (QI) interventions.

**Methods**: An 11‐item standardized tool, developed by Blantyre Department of Health and UCSF HEALTHQUAL, assessed domains including privacy, respect, clinic environment, fear of HIV labelling and PrEP decision‐making. Sample sizes for nine public facilities were calculated based on outpatient volume, with systematic sampling used to select participants. Hospital ombudsmen and health surveillance assistants administered surveys using REDCAP. QI coaching facilitated data use for design of stigma‐reduction strategies.

**Results**: Between June and December 2024, 797 clients (64% of projected sample) completed the survey. Most clients reported comfort discussing PrEP with providers (91%) and no discrimination from providers or staff (91%). However, 89% were counselled to be monogamous, 22% had to initiate PrEP discussions themselves and 17% feared stigma associated with PrEP use. All clients described clinics as welcoming and providers as respectful; nevertheless, 10% reported receiving inadequate health information. Significant variation was observed across facilities related to routine offering of PrEP.

**Conclusions**: Findings reveal persistent stigma and inconsistent PrEP service delivery in Blantyre. While many facilities offer supportive environments, many patients are required to initiate PrEP discussions and monogamy is promoted, reflecting ongoing need for provider sensitization to enhance patient engagement. Interventions are needed to standardize offering of PrEP within public health facilities; stigma‐related barriers need to be addressed. Embedding stigma reduction within QI processes offers a practical approach to reducing stigma and strengthening PrEP uptake.

### OAD0406LB The intersection of climate change and health: investigating the association of drought and HIV prevalence in Kenya

G. Kangogo ^1^, T. Loux^2^, K. Li^2^, M. Teni^2^, K. Trout^1^, E. Shacham^3^

^1^University of Missouri, Department of Health Sciences, Columbia, United States, ^2^Saint Louis University, Department of Epidemiology and Biostatistics, Saint Louis, United States, ^3^Saint Louis University, Department of Behavioral Science and Health Equity, Saint Louis, United States

**Background**: The intersection of climate change and public health, including the impacts of droughts, floods, high temperatures and migration, is a critical yet underexplored area, particularly in the context of HIV/AIDS. Limited findings have associated droughts with adverse health outcomes and disruptions to public health programmes. The aim of this study was to examine the relationship between climate‐induced droughts and HIV prevalence in Kenya.

**Methods**: This study utilized nationally representative data including biomarker testing for participants from the 2019 Kenya Population‐Based HIV Impact Assessment (KENPHIA) survey. Drought conditions were quantified using precipitation data from the Climate Hazards Group InfraRed Precipitation with Station Data (CHIRPS). The association between drought and HIV prevalence as measured by biomarker testing was analysed using weighted binary logistic regression models, accounting for the complex survey design and weights.

**Results**: The analysis included 31,032 participants examining socio‐demographic factors, sexual behaviours and climate variables. Nearly half of the respondents (47.2%) had experienced drought. The majority of participants who tested positive for HIV (58.2%) resided in drought‐affected areas compared to those in non‐drought areas. Those who resided in drought‐affected areas had significantly higher odds of testing positive compared to those who had not experienced drought. High‐risk sexual behaviours such as transactional sex and intergenerational partnerships were significantly associated with HIV prevalence. Lower educational attainment and food insecurity were significantly associated with increased odds of testing positive for HIV.

**Conclusions**: We found that participants who had experienced droughts were more likely to test positive for HIV. This may be because droughts exacerbate socio‐economic vulnerabilities, such as food insecurity and migration, which can increase high‐risk behaviours and reduce access to healthcare services. In light of this, these findings underscore the compounded risks of climate change on public health, emphasizing the need for integrated strategies that address both climate adaptation and HIV prevention.

### OAD0506LB Mpox awareness and access barriers among transgender and intersex communities in Kenya

C. Njuguna ^1^

^1^ALISON, Nairobi, Kenya

**Background**: ​In early ​2025, Kenya confirmed ​Mpox cases across multiple countries. Mpox is rarely discussed in the context of gender diverse communities. ​Transgender and intersex ​communities already facing systemic stigma and ​healthcare discrimination were potentially at high risk due to overlapping vulnerabilities and limited access to accurate health information. This ​project is aimed to assess Mpox knowledge and health‐seeking ​behaviour among these populations.

**Description**: Around February−March 2025, series of grassroot‐led Mpox sensitization session were conducted in Nairobi's informal settlements targeting transgender and the intersex persons. Activities included peer‐led group discussions, one on one outreach both in person and digital platforms. Special attention was given to correcting misinformation, recognizing Mpox symptoms and how ​different they are to the other health conditions and addressing barriers ​to seeking ​care particularly fear of ​judgement and prior negative experiences in health facilities.

**Lessons learned**: After the surveys, it was revealed that 85% of the participants had never received Mpox‐related information and many confused with other skin‐related conditions common among people living with HIV. Intersex too expressed heightened fear due to social invisibility and legal misrecognition despite Kenya's 2022 legal recognition of intersex as a gender. Peer‐led education improved trust significantly, knowledge uptake and willingness to discuss symptoms openly. However, access to Mpox screening and referrals through county‐level outreaches remains limited and were often perceived as unsafe due to integration with general services.

**Conclusions/Next steps**: Community‐specific Mpox interventions must be integrated into national health responses using rights based and inclusive frameworks. ​Health workers need training on gender diversity to ​reduce stigma in service provision. Future steps include scaling up safe space outreaches, decentralizing ​Mpox information materials and including the transgender and intersex voices in preparedness planning and surveillance efforts.

### OAD0606LB Co‐designing trust: community voices in building an AI companion for HIV prevention

S. Cooper^1^, S. Frade^2^, N. Maricich^2^, R. Mendonca^1^, S. Morris^1^, C. Govathson^3^, A. Spyrelis^4^, S. Stafford^4^, P. Matambanadzo^5^, S.T. Chabata^5^, S. Musemburi^5^, B. Chingombe ^5^

^1^Audere, Seattle, United States, ^2^Audere Africa, Johannesburg, South Africa, ^3^HE2RO Indlela, Johannesburg, South Africa, ^4^Shout‐It‐Now, Johannesburg, South Africa, ^5^CeSHHAR, Harare, Zimbabwe

**Background**: Adolescent girls and young women (AGYW), female sex workers (FSWs) and individuals who sell sex but do not self‐identify as sex workers (i.e. on the edge of sex work [EoSW]) in southern Africa face persistent barriers to HIV prevention and care. Digital health solutions using artificial intelligence (AI) offer promise, but their effectiveness depends on user trust, relevance and meaningful engagement. This abstract shares formative and summative findings from the participatory design and testing of the “Self‐Care from Anywhere” (SCFA) Companion—an AI‐powered conversational tool co‐created with AGYW, FSW and EoSW to support HIV prevention and care, and explores how a priority populations’–driven design process contributed to an accessible, trustworthy and engaging tool.

**Description**: In South Africa, co‐design included structured focus groups with AGYW during a workshop, followed by informal conversations and mini‐feedback sessions at mobile service points, such as clinics. These engagements validated concepts and informed refinements to content, tone and features. Insights from both phases guided iterative usability testing. Summative methods included structured observations and usability surveys with 90 AGYW aged 15–24. In Zimbabwe, a 2‐day workshop worked with FSWs, EoSW, clinicians, peer microplanners and government officials to inform the early stage design for EoSW and FSWs. At both sites, participants shaped chatbot tone, content, language and privacy features.

**Lessons learned**: Co‐design through interactive workshops and real‐time prototype engagement led to significant improvements in design and functionality. In South Africa, repeated feedback loops refined tone, navigation and privacy features (e.g. emojis, fake names), contributing to a System Usability Scale score of 88.1. In Zimbabwe, early engagement identified core needs like local language support and stigma‐free referrals. Importantly, co‐design extended beyond initial development. Continuous monitoring and updates based on user interactions continue to enhance usability and trust. This ongoing co‐design approach now includes live demos, embedded feedback capture and localized content updates as standard practice.

**Conclusions/Next steps**: Sustained, inclusive co‐design is essential for digital tools that resonate. There is strong demand for trusted, context‐aware AI companions supporting HIV and SRH. Next steps include ongoing feedback loops, engagement tracking, regional language support and adapting workflows to diverse contexts.

### OAE0106LB Empowering young female sex workers in coastal Kenya: a community‐led approach to HIV prevention through peer education and digital reminders (2023–2024)

R. Gachiri ^1^, S. Kamiri Thuo^2^

^1^KANCO, Programs, Nairobi, Kenya, ^2^Kenya Red Cross Society, Global Fund Program Unit, Nairobi, Kenya

**Background**: In Kenya's coastal counties of Mombasa, Kwale and Kilifi, young female sex workers (YFSWs) are at high risk for HIV/AIDS due to stigma, economic instability and limited healthcare access. In 2023, a strategic initiative integrated digital health systems with peer‐led programmes. The new platform includes a calendar for quarterly screening reminders and facilitates ongoing peer education, aiming to improve healthcare access and continuity for high‐risk groups.

**Description**: The initiative helped young female sex workers (YFSWs) access HIV prevention services through a digital platform that sent quarterly screening reminders, addressing forgetfulness and logistical barriers. Peer educators provided ongoing education via messaging apps on topics like condom use and PrEP, fostering trust and engagement. Support networks allowed YFSWs to connect and share experiences, empowering them in managing their health and promoting sustainable health‐seeking behaviours.

**Lessons learned**: The integration of digital tools with peer‐led HIV prevention strategies has significantly benefited young female sex workers (YFSWs) in Kenya's coastal counties, particularly Mombasa, Kwale and Kilifi. By the end of the programme, over 78% of YFSWs accessed HIV testing regularly, and 60% reported consistent condom use. A digital reminder system improved health screening adherence, with peer educators using WhatsApp and Facebook to provide culturally relevant education, leading to an 80% increase in knowledge about HIV prevention. However, challenges in digital access remained, as only 80% had reliable mobile data, despite 98% owning phones. Collaboration with local health systems was crucial, resulting in 85% of YFSWs receiving effective referrals to clinics for follow‐up care, highlighting the effectiveness of combining digital tools with health services.

**Conclusions/Next steps**: This peer‐led, digitally enabled approach has improved HIV prevention among young female sex workers by enhancing service continuity and community engagement. Mobile reminders and peer education have increased access to health services and reduced stigma. Future goals include scaling the model, improving digital access and expanding engagement in various spaces. Real‐time data systems will aid responsive programming. With ongoing funding, this model is a cost‐effective strategy for equitable, community‐led HIV prevention, contributing to efforts to end AIDS as a public health threat.

### OAE0204LB Assessing the impact of USG funding cuts on Zambia's HIV programming: a retrospective review of PrEP and VMMC uptake (2024–2025)

M. Silondwa ^1^, B. Longwe^1^, C. Phiri^2^, M. Mulenga^1^, A. Mwamelo^1^, J. Drakes^3^, D. Resar^3^, P. Haimbe^1^, H. Shakwelele^1^, L. Mulenga^2^

^1^Clinton Health Access Initiative, Lusaka, Zambia, ^2^Ministry of Health, Lusaka, Zambia, ^3^Clinton Health Access Initiative, Boston, United States

**Background**: Zambia has made strides in reducing the HIV burden with incidence having declined from 0.61% in 2016 to 0.31% in 2021 among the general population aged 15–49 years (ZAMPHIA). Zambia's commitment to curb HIV has seen rollout of programmes such as voluntarily medical male circumcision (VMMC), oral and injectable pre‐exposure prophylaxis (PrEP). These programmes have expanded rapidly largely due to the support from the United States Government (USG). This analysis assesses the impact of USG funding disruptions on PrEP and VMMC in Zambia.

**Description**: A retrospective analysis of secondary data from DHIS2, the Ministry of Health's national data system, was conducted to assess uptake in oral PrEP and VMMC services, comparing Q1 2024 to Q1 2025. The analysis focused on quarterly changes in uptake across geographies with cross‐tabulations and trend analysis used to identify performance implementation gaps and emerging patterns.

**Lessons learned**: The HIV treatment programme showed stability compared to the sharp declines in prevention. Between January and March 2024 and the same period in 2025, the number of individuals on ART declined marginally by just 0.5% (from 3,863,872 to 3,844,998). In contrast, oral PrEP initiations dropped from 71,608 in Q1 2024 to 28,230 in Q1 2025, a 60.6% decrease. This performance was 51% below the quarterly target of 58,120. VMMC services saw a similar drop from 107,034 individuals in Q1 2024 to 60,546 in Q1 2025, a 43.4% decline, achieving just 49.5% of the quarterly target of 144,589.

**Conclusions/Next steps**: USG funding cuts have disrupted Zambia's HIV prevention programmes, particularly in USAID‐supported provinces, leading to reduced uptake of PrEP and VMMC, threatening broader epidemic control. Urgent action is needed to assess the full impact of the disruptions to inform government‐led efforts to sustain services through domestic resource mobilization, reprioritization to ensure continuity and robust monitoring systems to build resilience against external shocks.[Fig jia226518-fig-0034]

**Figure 1 jia226518-fig-0034:**
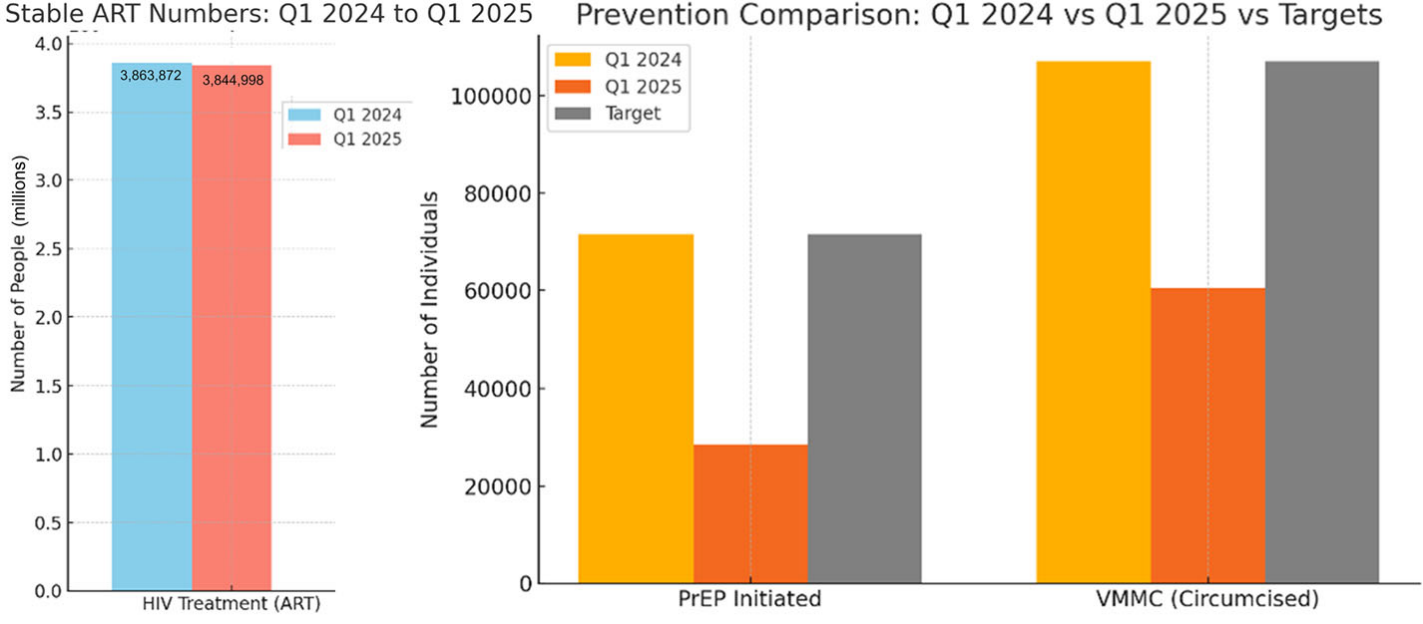
OAE0204LB

### OAE0205LB From vertical to domestic: transition readiness of national HIV responses in 14 countries in Africa

S. Kilonzo ^1^, The HIV Leadership Forum‐ a community of practice of Director Generals of AIDS Commissions

^1^Yemaya Health Group, Nairobi, Kenya

**Background**: Following the halt of U.S. government funding in January 2025, the HIV Leadership Forum—comprising Director Generals of 40 national AIDS commissions—conducted a rapid transition readiness survey across 24 sub‐Saharan African countries to gauge service continuity, systems resilience and available transition assets. Fourteen countries (58%) responded by mid‐April, via a 46‐indicator Microsoft‐Forms assessment, analysed descriptively in Excel.

**Description**: Service‐delivery: All countries reported operational public‐health facilities and governments quickly integrated antiretroviral (ART) dispensing into OPD departments. PMTCT services remained functional in 83% of nations. However, 75% of NGO/community sites closed, disrupting 90% of prevention programmes.

Systems: All countries reported data interruptions in facility and national health information tied to NGO‐managed servers or licenses. ART client transfers occasionally occurred without records. Laboratory networks faced similar vulnerabilities.

Commodities: While 65% of countries had 9‐month ARV and testing commodity stocks, half held < 6 months of prevention‐specific ARVs or condoms. Supply chain disruptions varied, though eight countries indicated internal distribution breakdowns.

Human resources: Donor‐funded staff attrition totalled 123,668 across 12 countries, including 60% frontline clinical workers (nurses, doctors, lab staff) and 44,502 community volunteers. Contractual complexities hindered retention efforts.

Governance: Governments issued service‐continuity directives, repurposing HIV clinics and initiating domestic funding. Countries with partial control over procurement, logistics and data systems experienced milder disruptions.

Transition assets identified: Embedding HIV services in universal health coverage schemes, leveraging private/faith facilities for co‐payment models, expanding data interoperability, aligning community‐social services and strengthening AIDS commissions stewardship.

**Lessons learned**: Decades of significant HIV systems investments collapsed, underscoring the fragility of donor‐dependent structures and the protective role of integrated national platforms. Public facilities struggle with sudden ART client influxes amid system disruptions. The attrition of 60% frontline health workers risks care quality, while prevention collapses demand urgent cross‐sector models targeting at‐risk populations. Reported commodity stocks are residual from pre‐January shipments, thus, urgent domestic investments are needed.

**Conclusions/Next steps**: Governments demonstrated rapid adaptation and now transition stewardship is required. Immediate priorities include: ecosystem audits to resolve client data gaps; revised commodity forecasts reflecting new client distributions; redesigned, cross‐sector prevention models; partnerships with PLHIV networks and private sector; workable HR retention solutions; and sustained political advocacy for domestic resources.

### OAE0306LB Examining unmet PrEP need and PrEP equity among young people using pooled data from three African countries, October 2023−September 2024

S. Wallach ^1,2^, W. Reidy^1,2^, R. Fayorsey^2^, J. Franks^2,3^, S. Saito^1,2^

^1^Mailman School of Public Health, Columbia University, Department of Epidemiology, New York City, United States, ^2^ICAP ‐ Columbia University, New York City, United States, ^3^Mailman School of Public Health, Columbia University, Department of Sociomedical Sciences, New York City, United States

**Background**: HIV disproportionately affects young women (YW) compared to men (YM) ages 15–24 in eastern and southern Africa (ESA), with YW incidence two to four times that of YM. To examine HIV pre‐exposure prophylaxis (PrEP) access relative to need for young people, we measured PrEP‐to‐Need (PnRs) and PrEP Equity Ratios (PER) pooled across three countries in ESA.

**Methods**: We used aggregate data from 345 ICAP‐supported facilities, October 2023−September 2024. PnR numerators reflect PrEP access and denominators approximate PrEP need; lower PnRs indicate more unmet need. PERs assess equity of PrEP access relative to need between groups, with PER = 1 indicating equity. PnRs were operationalized using PEPFAR monitoring and reporting indicators as [annual PrEP_NEW (new PrEP initiations) + Q1 PrEP_CT (PrEP continuations)]/annual HTS_TST_POS (new HIV diagnoses) disaggregated by sex and age. PERs were PnRs for YW divided by YM and 20–24 divided by 15–19.

**Results**: YW PnRs (15−19: 6.73; 20–24: 3.63) were lower than YM's (15−19: 12.36; 20–24: 9.75) suggesting YW had less PrEP access relative to need. The sex PER = 0.42, demonstrating a 58% gap in PrEP access relative to need for YW compared to YM. PnRs were lower for 20‐ to 24‐year‐olds compared to 15‐ to 19‐year‐olds, suggesting less PrEP access relative to need in this older group. The age PER = 0.63, demonstrating a 37% gap in PrEP access relative to need for 20‐ to 24‐year‐olds compared to 15‐ to 19‐year‐olds. While YW PrEP access (PnR numerator) was greater for each age group, the larger YM PnRs were driven by lower new HIV diagnoses (denominator). Larger 15–19 PnRs were also driven by lower new diagnoses.

**Conclusions**: In this pooled data, YW have 58% less PrEP access relative to need than YM, and 20‐ to 24‐year‐olds had 37% less PrEP access relative to need than 15‐ to 19‐year‐olds. We recommend increased efforts to provide PrEP for YW and 20‐ to 24‐year‐olds. PnRs and PERs are important metrics for evaluating PrEP scale‐up.[Fig jia226518-fig-0035]

**Figure 1 jia226518-fig-0035:**
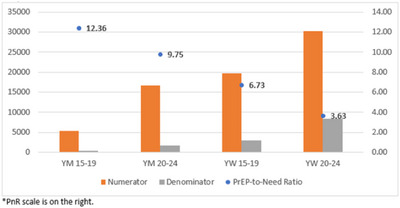
**OAE0306LB: PrEP‐to‐Need Ratios and PrEP‐to‐Need Ratio Numerators and Denominators for Young People in a Pooled Dataset from Three African Countries, 2024**.

### OAE0406LB Investigating adolescent‐friendly HIV prevention platforms as novel spaces for obesity interventions

C. Pike ^1^, C. Bondarchuk^2^, K. Lebelo^1^, E. Rousseau^1^, P. Macdonald^1^, P. Mapukata^1^, L.‐G. Bekker^1^, N. Chandiwana^1^

^1^Desmond Tutu Health Foundation, Woodstock, South Africa, ^2^Harvard Medical School, Boston, United States

**Background**: South Africa has built strong youth‐focused HIV prevention platforms through sustained investment. In contrast, research and services addressing obesity remain limited despite rapidly rising rates, particularly among adolescent girls and young women. This overlap presents an opportunity to leverage HIV prevention platforms for integrated interventions addressing both HIV and obesity. We describe the baseline prevalence of obesity in a real‐world PrEP programme for young people and evaluate the short‐term impact of long‐acting cabotegravir (CAB‐LA) on body mass index (BMI).

**Methods**: PrEPared to Choose (PtC) is a phase 3b study nested within the FASTPREP implementation study in Cape Town, South Africa. It evaluates uptake of oral pre‐exposure prophylaxis (tenofovir disoproxil fumarate/emtricitabine), the dapivirine vaginal ring, and CAB‐LA among AGYW aged 15–29 years and their male partners. At enrolment, participants selected one of the available PrEP options. BMI was measured at baseline to guide CAB‐LA needle selection (21G, 2‐inch for BMI ≥30; 23G, 1.5‐inch for BMI < 30). BMI was categorized using WHO definitions and described by age, sex and PrEP choice. Among participants who received ≥2 CAB‐LA doses, mean BMI change over 7 months was calculated. Multivariate regression was used to assess associations between baseline BMI and PrEP choice, and to evaluate BMI change over time.

**Results**: Over half of participants (56%, 657/1164) were overweight or had obesity at baseline (BMI ≥25 kg/m^2^), including 30% classified as having obesity (BMI ≥30 kg/m^2^). Among those with elevated BMI, 76% were female and 81% were under 29 years of age. The proportion of female participants increased with obesity severity: 74% in class I, 87% in class II and 93% in class III. Baseline BMI was not associated with PrEP product choice (aOR 1.23; 95% CI: 0.76–2.01). Among 612 participants who received at least two CAB‐LA doses, mean BMI decreased slightly over 7 months (mean change −0.021; 95% CI: −0.034 to −0.009; *p* = 0.001), while no meaningful change was observed in those who received fewer or no doses.

**Conclusions**: Short‐term CAB‐LA use was not associated with weight gain. The high baseline burden of obesity underscores the opportunity to integrate obesity care into HIV prevention platforms.

### OAE0606LB Building a safe, accurate AI companion: lessons from the field

S. Morris ^1^, N. Maricich^2^, S. Frade^2^, R. Mendonca^1^, S. Cooper^1^, A. Spyrelis^3^, S. Stafford^3^, D. Simpson‐Cupido^4^, D. Jaravani^4^, N. Moremi^4^

^1^Audere, Seattle, United States, ^2^Audere Africa, Johannesburg, South Africa, ^3^Shout‐It‐Now, Johannesburg, South Africa, ^4^CHAPS, Johannesburg, South Africa

**Background**: “Self‐Care from Anywhere” (SCFA), a multimodal AI tool including large language models (LLMs) and computer vision, is now in the hands of adolescent girls and young women (AGYW) in South Africa. This marks a milestone in a region where stigma, multilevel barriers and high HIV burden challenge traditional prevention strategies. As similar tools emerge, early field learnings from the SCFA Companion offer valuable insights into performance and value of AI as an efficient way to provide services in an era of drastically shrinking resources for HIV prevention.

**Description**: SCFA delivers private, on‐demand HIV support to AGYW through an AI‐powered WhatsApp chatbot that conducts risk assessments and provides tailored education, self‐testing guidance, relationship advice, and seamless connection to real clinicians—reducing stigma and closing access gaps. A clinician‐facing portal supports care with AI‐verified test results, and outreach tools. The platform was developed through cross‐organizational collaboration and supported by human‐in‐the‐loop review, and is designed to be scalable, customizable and interoperable with existing digital systems. Evaluation areas include youth engagement and priority topics; clinical accuracy and local relevance of LLM outputs; AI‐generated, data‐rich handoffs to clinicians when needed; clinical support utility; the feasibility of using structured data for ML‐based HIV risk models; and early cost‐per‐interaction analysis.

**Lessons learned**: Preliminary results show that built‐in mechanisms effectively limited AI‐generated inaccuracies, though hallucination rate data are still under analysis. Safety triggers were successful in identifying and addressing potentially harmful content. RAG functionality and the inclusion of slang understanding enhanced responsiveness and contributed to contextual accuracy, though further refinement is ongoing. Third, clinician feedback highlighted that AI‐generated summaries and test verification improved triage, though handoff processes between the chatbot and clinician will benefit from further streamlining, as many client requests for a human could have been handled by the chatbot. Ongoing costs are outweighed by benefits of user trust, interaction quality that provides greater access to care.

**Conclusions/Next steps**: This early deployment underscores the promise of AI tools like the SCFA Companion as scalable, cost‐effective innovations for HIV prevention. Ongoing work will refine safety, accuracy, engagement and adaptability features while evaluating long‐term performance and economic outcomes.

### OAS0102LB The impact of the U.S. funding interruption on HIV services and the HIV epidemic in Mozambique

D. Moiana Uetela ^1^, O. Tiberi^2^, R. Muleia^3^, P. Ramgi^4^, Y. Paulo^2^, I. Gaspar^2^, N. Scott^5^, R. Martin‐Hughes^5^, N.K. Martin^6^

^1^Instituto Nacional de Saude, Research Program on Diseases with Major Health Impact, Marracuene, Mozambique, ^2^Ministry of Health, National STI‐HIV/AIDS Control Program, Maputo, Mozambique, ^3^Instituto Nacional de Saude, Division of Surveys and Health Observation, Marracuene, Mozambique, ^4^Instituto Nacional de Saude, Health and Wellbeing Research Division, Marracuene, Mozambique, ^5^Burnet Institute, Melbourne, Australia, ^6^University of California San Diego, Department of Medicine, Division of Infectious Diseases and Global Public Health, San Diego, United States

**Background**: Mozambique has the eighth highest global HIV prevalence. ART is available in 97% of health facilities and serves approximately 2.1 million of the 2.4 PLHIV. However, the country relies heavily on external funds to ensure HIV service provision, with the United States covering approximately 93% of the country's expenses, mainly through PEPFAR. In January 2025, the U.S. government issued an executive order suspending almost all foreign aid programmes for 90 days. We aimed to assess the impact of this order on HIV service provision and predict the impact of potential fund interruption on the HIV epidemic in Mozambique.

**Methods**: To evaluate the impact of the executive order on HIV service provision, we used the country's district health information system to compare selected health services indicators in February 2024 and February 2025. We analysed the absolute variation and relative change in ART initiation and viral load cascade performance in these time points. To predict the impact on the HIV epidemic, we used a published dynamic HIV transmission and disease progression model (Optima) calibrated to Mozambique data to compare the trend before January 2025 to a counterfactual scenario with funding interruption, assuming a stagnation in the scenario observed in February 2025. We simulated the impact of funding interruption on HIV incidence and HIV‐related mortality until 2030.

**Results**: Comparing February 2024−2025, there was a 25% reduction in ART initiation in adults (≥15 years), from 22,865 to 17,105. Among those on treatment, there was a 38% reduction (37,010) in viral load tests performed, a 37% reduction (27,656) in test results received and a 33% reduction (22,043) in viral suppression. In children (< 15 years), these indicators were 44%, 71% and 43%, respectively, showing a disproportionate impact on paediatric population. The funding interruption was estimated to cumulatively increase 90,522 (23% more than the status quo) new HIV infections and 28,631 (33%) HIV‐related deaths by 2030.

**Conclusions**: The findings highlighted the immediate negative impact of the U.S. funding freeze on HIV service outcomes and the potential alarming effect on the HIV epidemic, in case the funding interruption is sustained. Funding is urgently needed to mitigate these negative impacts.

### OAS0103LB Modelling the impact of cuts in PEPFAR funding for HIV pre‐exposure prophylaxis among key populations in sub‐Saharan Africa

J. Stone ^1^, K. Kipkoech^1^, A. Artenie^1,2^, R. Silhol^3^, M.‐C. Boily^3^, J. Ratevosian^4^, C. Beyrer^4^, P. Vickerman^1^

^1^University of Bristol, Bristol, United Kingdom, ^2^Université de Montréal, Montreal, Canada, ^3^Imperial College London, London, United Kingdom, ^4^Duke University, Durham, United States

**Background**: In January 2025, the US government issued a directive, pausing all foreign aid programmes. This included a 90‐day pausing of all US President's Emergency Plan for AIDS Relief (PEPFAR) funding for HIV pre‐exposure prophylaxis (PrEP) except for pregnant women, with a return to funding for key populations (KPs) looking increasingly pessimistic. We estimated the impact of a funding pause for PrEP among KPs in sub‐Saharan Africa (SSA).

**Methods**: We developed an HIV transmission model incorporating PrEP, parameterized using systematic reviews of population size estimates, HIV prevalence and incidence. We used PEPFAR reporting on numbers of each KP returning for PrEP in July−September 2024 as the estimated number using PrEP provided by PEPFAR. For each country and KP, we estimated the proportion of HIV‐negative KPs receiving PrEP through PEPFAR and modelled the relative and absolute increase in new HIV infections resulting from removing this PrEP over the next year.

**Results**: By late 2024, 191,541 KP individuals across 27 SSA countries received PrEP funded through PEPFAR. The estimated proportion of HIV‐negative KP individuals receiving PrEP through PEPFAR ranged from 1.3% (95% credibility interval: 1.0−1.8) among people who inject drugs (PWID) to 5.5% (4.5−6.6) among female sex workers (FSWs). Stopping PEPFAR's provision of PrEP could lead to 4577 (3398−5757) additional new HIV infections over 1 year; 2215 (1367−2866) among men who have sex with men (MSM), 1838 (1198−2763) among FSWs, 350 (186−617) among transgender women (TGW) and 154 (53−232) among PWID. This corresponds to relative increases in new HIV infections of between 0.7% (0.2−1.1) among PWID to 3.1% (1.9−4.0) among MSM, with country‐level increases estimated to exceed 5% in seven countries for MSM, two countries for PWID and six countries for TGW and FSWs.

**Conclusions**: Mitigation measures, including funding through alternative international donors or domestic government budgets, are needed to prevent the detrimental impact of PEPFAR's funding cuts for PrEP, particularly in high coverage countries.

### OAS0104LB Short‐cycle antiretroviral therapy (ART) with weekends off is inferior to continuous ART in adolescents living with HIV receiving tenofovir disoproxil fumarate/lamivudine/dolutegravir (TLD) in sub‐Saharan Africa: BREATHER Plus 96‐week results

A. Kekitiinwa ^1^, A. Jennings^2^, M. Bwakura Dangarembizi^3^, C. Kityo^4^, A. Siika^5^, M. Archary^6^, L. Jafta^7^, G. Akabwai^1^, M. Chitsamatanga^3^, S. Nakabuye^4^, C. Kiilu^5^, R. Mngqbisa^6^, A. Green^2^, J. Seeley^8^, H. Mugerwa^4^, S. Walker^9^, N. Apoto^1^, D. Burger^10^, S. Salomone^2^, A. South^2^, C. Giaquinto^7^, A. Bamford^2^, M. Thomason^2^, D. Ford^2^, S. Pett^2^, BREATHER Plus trial team

^1^Baylor College of Medicine, Department of Pediatrics, Kampala, Uganda, ^2^Medical Research Council Clinical Trials Unit at University College London (UCL), London, United Kingdom, ^3^University of Zimbabwe Clinical Research Centre, Harare, Zimbabwe, ^4^Joint Clinical Research Centre, Kampala, Uganda, ^5^Moi University Clinical Research Centre, Eldoret, Kenya, ^6^University of KwaZulu‐Natal, Department of Paediatrics and Children Health, King Edward VIII Hospital, Enhancing Care Foundation, Durban, South Africa, ^7^Fondazione Penta ETS, Padova, Italy, ^8^Medical Research Council/Uganda Virus Research Institute and London School of Hygiene and Tropical Medicine Uganda Research Unit, Entebbe, Uganda, ^9^University of York, Centre for Health Economics, York, United Kingdom, ^10^Radboud University Medical Center Nijmegen, Department of Pharmacy, Nijmegen, the Netherlands

**Background**: Short‐cycle therapy (SCT) with 4/5 sequential days‐on ART, 2/3 days‐off ART has previously shown non‐inferior virological outcomes versus daily ART. Most data are in adults with frequent real‐time viral load (VL) testing; dolutegravir‐based SCT data are limited.

**Methods**: BREATHER Plus was a non‐inferiority, 96‐week trial in Kenya/South Africa/Uganda/Zimbabwe. Adolescents (12−19 years), suppressed (VL< 50 copies/ml) on tenofovir disoproxil fumarate/lamivudine/dolutegravir (TLD), with no prior treatment failure, were randomized to continue daily ART (CT) or switch to SCT (5 days‐on, 2 days‐off). The primary outcome was the adjusted Kaplan‐Meier estimated proportion with confirmed VL ≥50 copies/ml by week 96. The non‐inferiority margin and significance level depended on the CT event rate (8% margin, 99% CI for 5% event rate). Routine 6–12 monthly VLs were used for participant management; SCT participants with real‐time confirmed VL ≥50 copies/ml returned to CT. Additional 8–12 weekly VLs were measured retrospectively. A MEMScap sub‐study (*n* = 210) in Uganda/Kenya captured ART bottle openings across weeks 8–32 and 48–72. We present 96‐week data.

**Results**: Four hundred and seventy (239 SCT, 231 CT) participants were enrolled (263 [56%] female, median age 16.5 years [IQR: 14.6−18.1], prior ART duration 11.8 years [IQR: 8.6−14.1]). At week 96, 98% remained in follow‐up. Twenty‐three (10%) SCT versus 11 (5%) CT participants had confirmed VL ≥50 copies/ml, difference SCT‐CT 5% (99% CI: −0.9, 11.5); an 8% higher rate of virological rebound in SCT was not rejected (Figure 1). At the 5% significance level, SCT was inferior to CT (*p* = 0.034). One SCT and two CT participants changed ART regimen for AEs. Proportions with cross‐sectional VL< 50 copies/ml at week 96 were similar (215 [93%] SCT, 211 [93%] CT). Fourteen SCT and 14 CT participants experienced ≥1 SAE (*p* = 0.938). In the MEMSCap sub‐study, 93% and 92% of expected tablets were taken in SCT and CT, respectively, although in both groups, only 71% of weeks were fully adherent to strategy.

**Figure 1:**
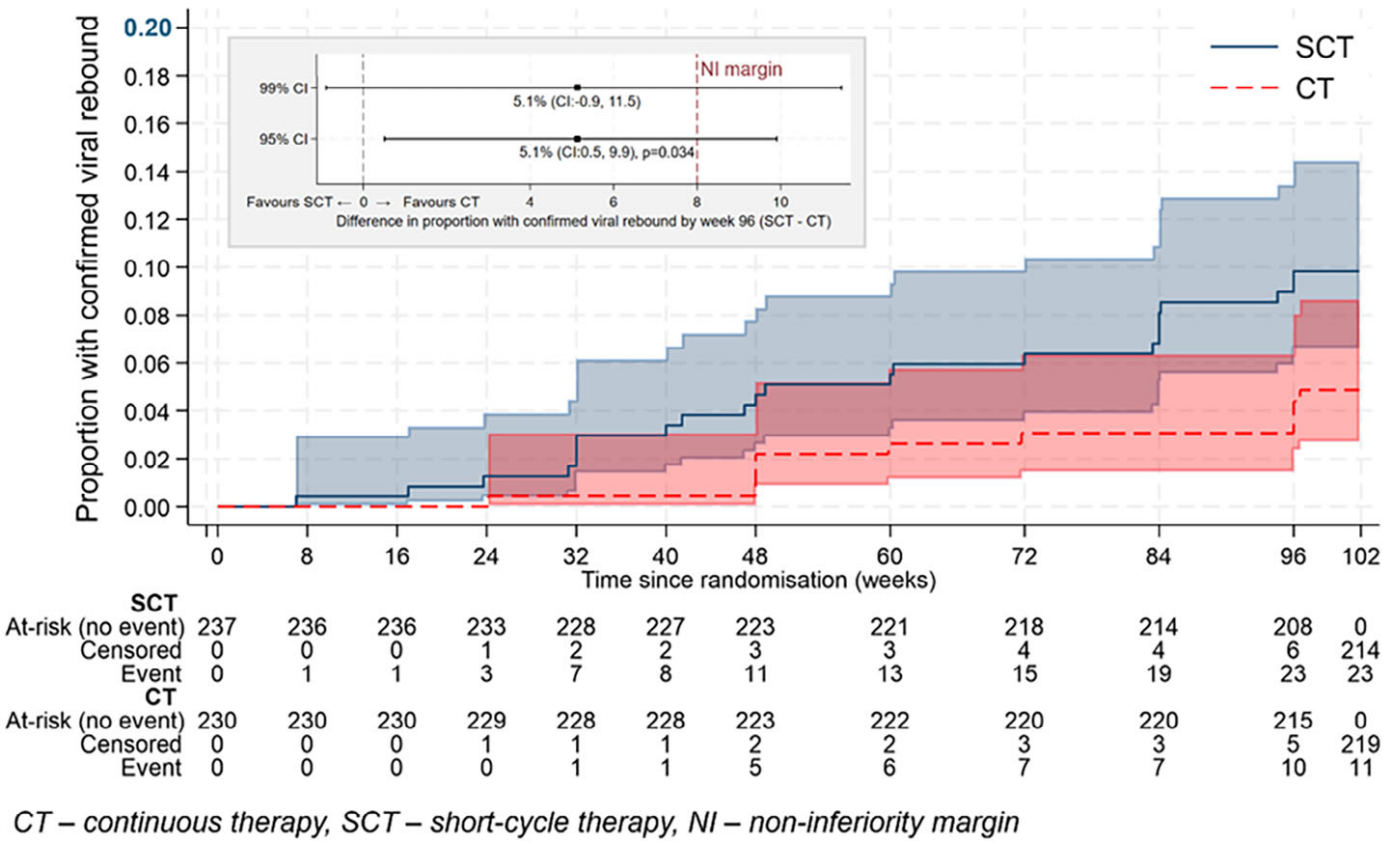
**OAS0104LB: BREATHER plus primary outcome ‐ proportion with confirmed viral rebound (viral load ≥50 copies/mL) by week 96, by trial arm**.

**Conclusions**: TLD‐based SCT cannot be recommended for adolescents living with HIV, using 6–12 monthly VL monitoring.

### OAS0105LB Long‐acting cabotegravir and rilpivirine in adults with suboptimal HIV control in sub‐Saharan Africa: the IMPALA trial 48‐week results

F.V. Cresswell ^1,2,3^, P. Kafeero^1^, C. Norcross^1,3^, V. Tumusiime^1^, L. Achieng Ombajo^4^, N. Garrett^5,6^, N.C. Owarwo^7^, E.A. Laker Odongpiny^7^, S. Mahomed^5^, S. Kassim^6^, I. Yawe^8^, U. Bahemuka^1^, J. Kitonsa^1,3^, V. Ankunda^1^, J. Nabbuto^1^, C. Ogwang^1^, P. Nabaggala^1^, G. Kimbugwe^1^, A. Kakande^1^, A. Onyango^4^, J. Nkuranga^4^, E. Koile^9^, D. van der Vendt^6^, D.S. Lawrence^2^, U. Singh^5^, H. Mugerwa^10^, A. Idahosa^11^, F. Addo Boateng^12^, W. van der Horst^13^, I. Eshun‐Wilsonova^14^, D. Grint^2^, E. Ruzagira^1,2^, on behalf of the IMPALA Study Group

^1^MRC/UVRI‐LSHTM Uganda Research Unit, Entebbe, Uganda, ^2^London School of Hygiene and Tropical Medicine, London, United Kingdom, ^3^Brighton and Sussex Medical School, Global Health and Infection, Brighton, United Kingdom, ^4^University of Nairobi, Nairobi, Kenya, ^5^CAPRISA, Durban, South Africa, ^6^Desmond Tutu Health Foundation, Cape Town, South Africa, ^7^Infectious Diseases Institute, Kampala, Uganda, ^8^Joint Clinical Research Centre, Fort Portal, Uganda, ^9^Jomo Oginga Odinga Teaching and Referral Hospital, Kisumu, Kenya, ^10^Joint Clinical Research Centre, Lubowa, Uganda, ^11^Johnson and Johnson, Lagos, Nigeria, ^12^Johnson and Johnson, Accra, Ghana, ^13^Johnson and Johnson, Beerse, Belgium, ^14^Johnson and Johnson, Cape Town, South Africa

**Background**: In sub‐Saharan Africa, 1.3 million people taking oral antiretroviral therapy (ART) are not virally suppressed. Long‐acting (LA) treatment may benefit those with adherence challenges. We investigated the efficacy and safety of LA cabotegravir (CAB) and rilpivirine (RPV) in a population previously non‐adherent to oral ART.

**Methods**: IMPALA (NCT05546242) is a multicentre, open‐label, randomized, non‐inferiority trial in Uganda, Kenya and South Africa. Participants had recent HIV‐1 viral load (VL) > 1000 copies/ml or poor engagement in care. Viral suppression (< 200 copies/ml) on dolutegravir‐based therapy was required prior to randomization. Participants were randomized 1:1 to receive 2‐monthly CAB+RPV‐LA or to continue oral therapy. The primary endpoint was the proportion with VL < 50 copies/ml at 48 weeks in the intention‐to‐treat‐exposed population (by FDA snapshot algorithm, −10% non‐inferiority margin). Secondary endpoints included confirmed virological failure (CVF, two consecutive VLs ≥200 copies/ml, 4% non‐inferiority margin; a single VL > 1000 copies/ml was a pre‐specified sensitivity analysis).

**Results**: Of 540 participants, 60.0% were female, median age 40 years (IQR 33−48), 99.6% were Black. At 48 weeks, 91.4% (245/268) on CAB+RPV‐LA had VL < 50 copies/ml versus 89.2% (240/269) on oral ART (risk difference [RD]: 2.3%, 95% CI −2.7% to 7.2%) (Figure 1). CVF was infrequent (1.9% [5/268] with CAB+RPV‐LA vs. 0/269 oral arm; RD: 1.9%, 0.3−3.5%). For any VL > 1000 copies/ml during follow‐up, CAB+RPV‐LA was superior (7/268 vs. 18/269; RD: −4.1%, −7.7% to −0.6%). All 4/4 with CVF who switched to once‐daily dolutegravir‐based ART were re‐suppressed. Safety outcomes were comparable. Of those on CAB+RPV‐LA, 93.6% (249/266) preferred injectable therapy.

**Figure 1:**
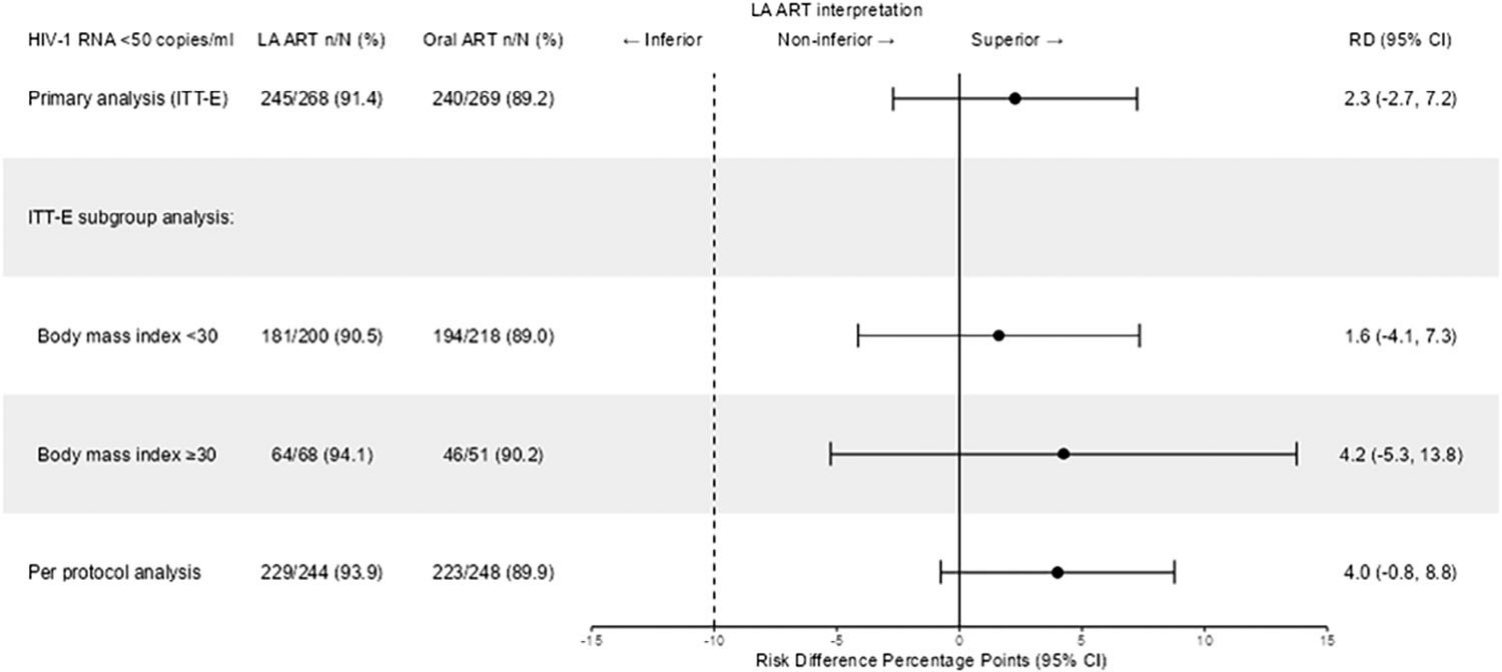
OAS0105LB

**Conclusions**: In people with adherence challenges, CAB+RPV‐LA was non‐inferior to dolutegravir‐based ART for viral suppression and CVF, and performed better when considering any VL > 1000 copies/ml, which is relevant for HIV transmission risk. A strong preference for injectable therapy indicates that CAB+RPV‐LA may play a key role for specific populations in the public health approach.

### OAS0106LB Safety and pharmacokinetics of MK‐8527 oral once‐monthly: a phase 2 study in adults at low risk of HIV‐1 exposure

K. Mayer ^1,2^, P. Kotze^3^, J. Lombaard^4^, Y. Caraco^5^, A. Peer^6^, N. Poovan^7^, S. Khalsa^8^, S. Khetan^9^, A.E. Shapiro^10^, M. Rassool^11^, A. Gonzalez^12^, Y. Singh^13^, G. Sinclair^14^, S. Riddler^15^, S. Buchbinder^16^, M. Vesay^17^, B. Homony^17^, B. Evans^17^, A. Grandhi^17^, C. Zhang^17^, M. Skataric^17^, M.N. Robertson^17^, Y. Kapoor^17^, R.P. Matthews^17^, R.M. Plank^17^

^1^Fenway Health and Department of Medicine, Beth Israel Deaconess Medical Center, Boston, United States, ^2^Harvard Medical School, Boston, United States, ^3^Qhakaza Mbokodo Research Clinic, Ladysmith, South Africa, ^4^Josha Research, Bloemfontein, South Africa, ^5^Hadassah‐Hebrew University Medical Center, Jerusalem, Israel, ^6^Rambam Health Care Campus, Haifa, Israel, ^7^Wits RHI, University of the Witwatersrand, Johannesburg, South Africa, ^8^Albuquerque Clinical Trials, Inc., Albuquerque, United States, ^9^Velocity Clinical Research, Rockville, United States, ^10^University of Washington & Fred Hutchinson Cancer Center ‐ Seattle HIV Vaccine Trials Unit, Seattle, United States, ^11^Helen Joseph Hospital‐Clinical HIV Research Unit, Johannesburg, South Africa, ^12^Advanced Research for Health Improvement, Immokalee, United States, ^13^Desmond Tutu HIV Centre, Institute of Infectious Diseases and Molecular Medicine, University of Cape Town, Cape Town, South Africa, ^14^Prism Health North Texas, Oak Cliff Health Center, Dallas, United States, ^15^University of Pittsburgh, Pittsburgh, United States, ^16^Bridge HIV‐ San Francisco Department of Public Health, San Francisco, United States, ^17^Merck & Co., Inc., Rahway, United States

**Background**: Long‐acting options for HIV‐1 pre‐exposure prophylaxis (PrEP) are needed. MK‐8527 is a novel, oral, nucleoside reverse transcriptase translocation inhibitor (NRTTI) with pharmacokinetic properties supporting once monthly (QM) dosing. We studied the safety and pharmacokinetics of MK‐8527 oral QM in adults at low risk of HIV‐1 exposure.

**Methods**: In this double‐blind, multicentre study (NCT06045507), adults 18–65 years of age were randomized 2:2:2:1 to receive MK‐8527 (3, 6 or 12 mg) or placebo QM for 6 months. Adverse events and laboratory tests were monitored through 8 weeks after the last dose. MK‐8527 in plasma was measured for all participants. MK‐8527‐triphosphate (TP), the active form, in peripheral blood mononuclear cells (PBMCs) was measured in a subset of participants. Pharmacokinetic exposures for MK‐8527 and MK‐8527‐TP were based on non‐compartmental analysis of the sparse data collected in the study.

**Results**: Three hundred and fifty participants were enrolled (58.3% female; median age 28 years; 51.4% White, 41.4% Black/African American, 2.3% Asian) and received at least one dose of study intervention; 328 (93.7%) received all six doses. The incidence of adverse events was similar for MK‐8527 and placebo (Table 1). No clinically meaningful changes were seen in laboratory tests, including total lymphocyte and CD4 T‐cell counts (Table 1). Pharmacokinetic parameters for MK‐8527 and MK‐8527‐TP were dose proportional (Table 2).

**Table 1:** OAS0106LB: Summary of Safety through Follow‐Up Week 8

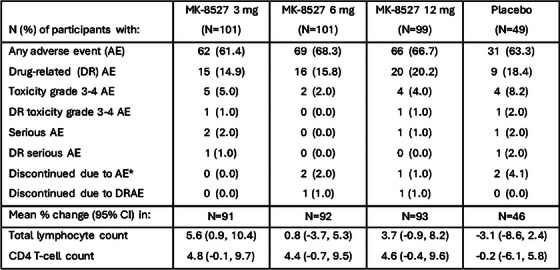

* AE's causing discontinuation of MK‐8527: migraine (*n* = 1) and decreased lymphocyte/CD4 T‐cell count (*n* = 1) in 6 mg group; hypoaesthesia (*n* = 1) in 12 mg group.

**Table 2:** OAS0106LB: Summary of Pharmacokinetic Parameters for MK‐8527 in Plasma and MK‐8527‐TP in PBMCs Following Administration of Multiple Oral Doses of MK‐8527

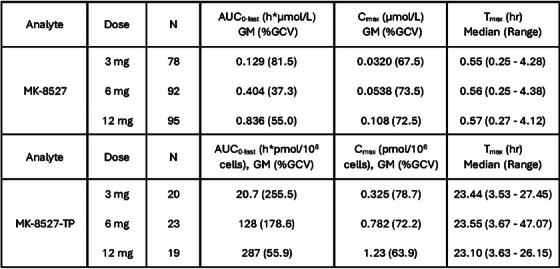

Abbreviations: AUC, area under the concentration time curve; C_max_, Maximum concentration; GCV, geometric coefficient of variation; GM, geometric mean; T_max_. time to maximum concentration.

**Conclusions**: MK‐8527 was well tolerated with a similar safety profile to placebo in adults at low risk of HIV‐1 exposure. The pharmacokinetics of MK‐8527 and MK‐8527‐TP support the continued development of MK‐8527 oral QM for PrEP.

## MPOX ABSTRACTS

### OAM0102 Epidemiological profile of the early cases of Mpox in Uganda, June 2024–January 2025

L.O. Namakula ^1^, R. Migisha^1^, J.O. Kobusingye^1^, E. Mfitundinda^1^, J. Nalwanga^1^, E. Okiror Okello^1^, A.M. Namusisi^1^, B. Ainembabazi^1^, D. Wenani^1^, J. Kobusinge Lubega^1^, H. Katumba^1^, C. Mutesi^1^, G. Abbo^1^, P. Kwizera^1^, B. Kwesiga^1^, D. Kadobera^2^, A.R. Ario^1^

^1^Uganda National Institute of Public Health, Field Epidemiology, Kampala, Uganda, ^2^Division of Global Health Protection, Global Health Center, US Centers for Disease Control and Prevention, Kampala, Uganda

**Background**: Mpox was declared a Public Health Emergency of International Concern in July 2022, and reinstated in August 2024. Uganda reported its first confirmed case on 24 July 2024, and by 15 December 2024, > 2000 confirmed Mpox cases had been registered. We described the epidemiology, exposures and transmission dynamics among the early cases of Mpox in Uganda, to guide containment and control strategies.

**Methods**: All persons tested for Mpox in Uganda provided baseline information for a national line‐list including: age, sex, residence, phone‐contacts, occupation and symptoms. The National Rapid Response Team conducted case investigations on confirmed cases at isolation centres and in communities. Individuals with unreachable phone‐contacts and not in isolation were not interviewed. A confirmed case was a laboratory‐confirmed Mpox infection in a resident of Uganda during 1 June 2024–22 January 2025.

**Results**: By 22 January 2025, 2209 laboratory‐confirmed case‐persons, including 13 deaths (case fatality rate = 0.6%), were reported across 78 (53%) of the country's 146 districts, 6 reported > 40 case‐persons per district. Kampala District was the most affected, accounting for 1205 (55%) case‐persons, with an attack rate of 64 per 100,000 population. Most (1259, 57%) were males, and the mean age was 27 (± 11) years, ranging from 0 to 80 years. A total of 424 (19.4%) confirmed cases provided occupational data, most of whom were students (10.8%) and female sex‐workers (9.9%). Three hundred (13.6%) confirmed cases were fully investigated, of which 71 (23.7%) had chronic illnesses with most 60 (85%) living with HIV. Exposure was established among 120 of the 300 fully investigated case‐persons, most (72, 61%) contracted Mpox through sexual contact, primarily driven by sex‐work. Most (*n* = 82, 41%) of the first 200 Mpox case‐persons sought care at private health facilities, where 85.6% (70/82) were misdiagnosed.

**Conclusions**: The Mpox outbreak in Uganda predominantly affected young adults, with a significant proportion of the early case‐persons seeking care at private health facilities, where misdiagnosis was common. Most of the 300 fully investigated case‐persons acquired Mpox through sexual transmission, mainly through sex‐work. To effectively interrupt transmission, we recommended targeted strategies such as vaccination, including high‐risk categories such as sex‐work networks, and improving surveillance especially within private health‐facilities.

### OAM0103 From displacement to disease: unpacking the syndemic interplay between HIV and Mpox among refugees in South Western Uganda

U. Muhumuza ^1^, E. Surafel^2^, Z. Nahumuza^3^, M. Naturinda^4^

^1^Africa CDC, Kampala, Uganda, ^2^Africa CDC, Addis Ababa, Ethiopia, ^3^International University of Africa, Khartoum, Sudan, the, ^4^Kampala International University, Kampala, Uganda

**Background**: The intersection of protracted displacement, weakening public health infrastructure and emerging zoonoses presents a fertile ground for syndemics in refugee settlements. This study introduces and tests the Syndemic Amplification Hypothesis—that HIV and Mpox in humanitarian settings do not co‐occur by coincidence but are biologically and socially intertwined, amplifying each other's burden through shared pathways of vulnerability. We aimed to determine the prevalence, co‐morbidity patterns, and social drivers of HIV and Mpox among refugees, with a focus on transactional survival sex, mobility and breakdown of social norms.

**Methods**: A prospective cohort study was conducted between October 2024 and March 2025 across Nakivale refugee settlements. We recruited 1030 participants (aged 13–49) through multistage cluster sampling. Testing for HIV and Mpox was combined with behavioural surveillance. Participants followed for 6 months assessing incident Mpox diagnoses and HIV seroconversions. Additionally, 24 digital life‐history interviews conducted using participatory timeline mapping and ethnographic shadowing of 12 youth at risk. Multivariate logistic regression and Cox proportional hazards models identified risk predictors. Qualitative data analysed using grounded theory.

**Results**: Baseline, HIV prevalence was 13.8% (18.6% female, 8.4% male). Mpox prevalence was 5.1% (72% adolescents aged 13–24). Among Mpox diagnoses, 47.2% were also HIV‐acquired. Co‐acquisition individuals had significantly higher risk of severe Mpox manifestations (OR: 3.9; 95% CI: 2.4–6.1) and longer healing times (median: 18 vs. 9 days; *p* < 0.001). Youth aged 15–24, 41.3% engaged in survival sex; condom access was consistently unavailable for 67%. Mobility across border regions (esp. DR Congo) was a significant predictor of Mpox incidence (HR: 2.7; *p* = 0.004). Qualitative analysis revealed a breakdown of normative sexual boundaries, erosion of parental roles, emerging scarcity‐driven sexual economies. Social trust in health workers was low due to stigma and militarized quarantine enforcement during Mpox outbreaks.

**Conclusions**: Our findings demonstrate that Mpox is no longer merely a zoonotic spillover but part of a larger syndemic dynamic in fragile settings. Targeted interventions must integrate syndemic thinking—addressing the social conditions enabling pathogen co‐propagation. We recommend: (1) integrated Mpox‐HIV screening and case management; (2) cross‐border disease surveillance; and (3) creation of safe spaces for youth‐centred care and sexual health education.

### OAM0104 PROJECT trial: proactive regional orthopox genomic epidemiology and cross‐regional outbreak tracking—a multinational investigation into Mpox evolution, vaccine efficacy and transmission dynamics across the Middle East

H. Tanaka^1^, S. Yamamoto^1^, G. Rahal^2^, Z. Khalil ^3^, A. Abdel‐Fattah^3^, K. Nassar^4^, Y. El‐Hadidy^4^, T. Sabry^4^, F. Zaki^4^, O. Khalil^4^, L. Barakat^3^, S. El‐Gazzar^3^, N. El‐Shenawy^4^, R. Sato^1^, A. Kobayashi^1^, K. Nakamura^1^, H. Suzuki^5^, Y. Ito^5^, K. Matsuda^5^, K. Al‐Mansoori^6^, F. Al‐Hashmi^6^, O. Al‐Neyadi^6^, A. Al‐Ketbi^6^, H. Al‐Falasi^6^, S. Al‐Mazrouei^7^, N. Al‐Rashid^7^, B. Al‐Qahtani^7^, L. Al‐Harbi^7^, Z. Al‐Otaibi^7^, Y. Al‐Hadid^8^, R. Al‐Abdallat^8^, S. Al‐Khalili^8^, L. Al‐Qudah^9^, Y. Abi Haidar^2^, M. Wehbe^2^, T. Jabbour^2^, N. El‐Khoury^2^, L. Zoghby^2^, IAS‐2025 group

^1^NCGM Disease Control & Prevention Center (DCC), Research Department, 1‐chōme‐21‐1 Toyama, Shinjuku City, Tokyo, Japan, ^2^Center for Infectious Diseases Research (CIDR) at the American University of Beirut Medical Center (AUBMC), Research Department, AUBMC, Department of Pediatrics and Adolescent Medicine, Phase 1, Lebanon, ^3^October 6 University, Research Department, Egypt‐Cairo, Egypt, ^4^Al‐Azhar University, Research Department, Egypt‐Cairo, Egypt, ^5^National Institute of Infectious Diseases, Toyama Research Office, Research Department, Shinjuku City, Tokyo, Japan, ^6^Kalba Hospital, Research Department, Sharjah Emirate, the United Arab Emirates, ^7^Riyadh Clinical Simulation Center, Research Department, Riyadh, Saudi Arabia, ^8^Information and Research Center ‐ King Hussein Foundation (IRCKHF), Research Department, Amman, Jordan, ^9^Jordan Hospital, Research Department, An‐Nuzha St, Amman, Jordan

**Background**: Mpox cases have surged across the Middle East since 2023, raising concerns over viral evolution, reduced vaccine protection and gaps in regional surveillance—particularly for people with HIV. The PROJECT trial characterized viral adaptation, immunity durability and outbreak dynamics in five Middle Eastern countries.

**Methods**: This prospective, multicentre cohort study enrolled 9126 individuals (including 687 people with HIV [PWH]) from Saudi Arabia, UAE, Egypt, Jordan and Lebanon over 18 months. Participants were stratified into: MVA‐BN‐vaccinated (N = 4573), prior‐Mpox‐exposed (N = 3129) and unvaccinated/unexposed controls (N = 1424), with HIV status documented for all. Full‐length viral sequencing (every 8 weeks) prioritized PWH samples for adaptive mutation analysis. Humoral/cellular immunity (neutralization titres, IFN‐γ ELISPOT) compared PWH (*n* = 687) versus non‐HIV participants. Transmission modelling weighted HIV‐specific risks (e.g. CD4 count < 200) alongside contact‐tracing and mass‐gathering data.

**Results**: Clade IV‐ME Mpox (41.3% prevalence) carries A27L‐L205F mutation enhancing spread. Vaccinated groups showed 64% antibody decline (1:128→1:46; 1:98→1:29 in PWH), with 17.4% breakthrough cases (23.1% in PWH; aHR = 2.21). T‐cell responses dropped 32% (47% in PWH; *p* < 0.001). R₀ = 1.72 (general) → 2.48 (mass gatherings), 28.4% linked to PWH super‐spreading. Asymptomatic rate: 13.6% (6.3% in PWH). Hospitalizations 1.9 × higher overall (3.2 × in PWH). Variant detection delayed 6 weeks (22% PWH undiagnosed pre‐hospitalization).1, 2

**Table 1:** OAM0104: Key findings and implications of Mpox clade IV‐ME

Finding	Data	Implication/clinical significance
A27L‐L205F mutation	Enhances viral adhesion/entry	Increased the ability to spread may require updated vaccines or therapeutics targeting this mechanism.
Neutralizing antibody decline	64% drop (1:128 → 1:46) by 12 months post‐vaccination	Current vaccines lose efficacy over time; booster doses may be needed for sustained protection.
Breakthrough cases	17.4% rate; aHR 2.21 if vaccinated > 10 months prior (95% CI: 1.88–2.59)	Revaccination schedules should prioritize high‐risk groups (e.g. immunocompromised, sexual health clinics).
T‐cell response reduction	32% lower versus Clade II (*p* = 0.003)	Cellular immunity is compromised; vaccines may need adjuvants to enhance T‐cell activation.
Increased transmissibility (R₀)	R₀ = 1.72 (baseline) → 2.48 (mass gatherings)	Targeted interventions (e.g. pre‐event vaccination, testing) critical for concerts/festivals.
Asymptomatic transmission	13.6% of secondary cases	Silent spread complicates containment; contact tracing and PCR screening must be prioritized.
Higher hospitalization in vaccinated	1.9 × higher versus unvaccinated (Egypt/Jordan)	Breakthrough cases may present atypically; clinicians need training to recognize Clade IV‐ME symptoms.
Delayed variant detection	Up to 6‐week lag in Lebanon/Jordan	Investment in regional genomic surveillance is urgent to track emerging variants.

**Table 2:** OAM0104: Mpox Clade IV‐ME: threat assessment matrix

Domain	Virologic feature	Population impact	Countermeasure gap
**The ability to spread**	• A27L‐L205F mutation (↑ adhesion)	• R₀ = 1.72→2.48 in mass gatherings	• Current vaccines target ancestral strains
**Immune escape**	• 32% ↓ T‐cell response (*p* = 0.003)	• 17.4% breakthrough cases (aHR = 2.21*)	• No clade‐specific boosters available
**Transmission**	• 13.6% asymptomatic transmission	• 6‐week variant detection delays in Levant	• Limited PCR screening capacity
**Clinical**	• 1.9 × ↑ hospitalization (vax vs. unvax)	• Atypical symptoms delay diagnosis	• Clinician training outdated

^*^95% CI: 1.88–2.59 for > 10 months post‐vaccination.

**Conclusions**: Clade IV‐ME Mpox exhibits enhanced spread (accelerated in PWH) and waning vaccine protection by 9–12 months (faster in PWH), urging updated boosters for high‐risk groups. Silent transmission and surveillance gaps (notably in PWH) demand strengthened genomic tracking and HIV‐inclusive outbreak responses during mass gatherings.

### OAM0105 Efficacy of modified‐vaccinia Ankara vaccine as pre‐exposure and post‐exposure prophylaxis against monkeypox sexual transmission in non‐human primate model

C. Herate ^1^, A. Ferrier‐Rembert^2^, F. Relouzat^1^, A.‐S. Gallouët^1^, Q. Pascal^1^, H. Letscher^1^, M. Cavarelli^1^, N. Bosquet‐Dereuddre^1^, W. Gros^1^, B. Delache^1^, S. Langlois^1^, H. Timera^2^, F. Jarjaval^2^, L. Bossevot^1^, C. Ludot^1^, C. Brua^3^, M. Lechemia^2^, O. Ferraris^2^, N. Silvestre^3^, R. Le Grand^1^, J.‐N. Tournier^2,4^

^1^Université Paris‐Saclay, Inserm, CEA, Center for Immunology of Viral, Auto‐Immune, Hematological and Viral Diseases (IMVA‐HB/IDMIT), IDMIT, Fontenay‐aux‐Roses, France, ^2^Institut de recherche biomédicale des Armées, Microbiology and infectious diseases department, Bretigny‐sur‐Orge, France, ^3^Transgene SA, Illkirch Graffenstaden, France, ^4^Ecole du Val‐De‐Grâce, Paris, France

**Background**: Two major monkeypox virus (MPXV) outbreaks caused by two distinct clades (I and II) were reported since 2022 causing a human infectious disease with unusual epidemiological and clinical features, notably an increase in human‐to‐human transmission via sexual route. This evolution underscores the need to reassess prevention and control strategies in the context of a sexually transmitted disease.

**Methods**: After establishing a mucosal transmission model of MPXV Clade IIb via the rectal route in cynomolgus macaques—which resulted in moderate disease characterized by skin lesions primarily in the genital area, localized proctitis, fever and lymphadenomegaly—we evaluated the immunogenicity and efficacy of the modified vaccinia Ankara (MVA) vaccine administered in both a pre‐exposure prophylaxis (PrEP) and a post‐exposure prophylaxis (PEP) regimen against MPXV IIb rectal transmission. We conducted a longitudinal follow‐up of the animals, monitoring viral replication as well as antibody and T‐cell responses induced by natural immunity in convalescent animals previously exposed to MPXV.

**Results**: We demonstrated that two doses of MVA induce specific B‐ and T‐cell responses against Clade IIb MPXV and elicit antibodies that cross‐neutralize MPXV Clade I and II viruses. The MVA‐PrEP immunization approach protects against subsequent Clade IIb MPXV rectal challenge with drastic decrease of viral load in different compartment including the genital tract. However, a single dose of MVA administered 4 days post‐exposure, as recommended by authorities, failed to protect macaques against Mpox disease. We also showed that the exposure of non‐vaccinated cynomolgus macaques to clade IIb MPXV via the atraumatic rectal mucosa route induces productive localized infection in the rectum, followed by systemic dissemination and Mpox disease, whereas previously MPXV‐challenged animals are protected against a new exposure via the same route.

**Conclusions**: Our findings validate two doses of MVA‐PrEP as an efficient vaccine protocol against Mpox in the context of the current epidemics, including in cases of mucosal transmission. However, both protection and immunological response data indicate that MVA‐PEP is not effective, as it failed to prevent MPXV production in the rectal or seminal fluids. These findings have a critical impact on outbreak management and highlight the importance of reevaluating MVA post‐exposure prophylaxis protocols.

## AUTHOR INDEX

### A

Abas, M. OAD0106LB

Abba, R. OAB0404

Abbo, G. OAM0102

Abdalla, S. OAB0305

Abdel‐Fattah, A. OAM0104

Abdelrahman, N.G. OAE0603

Abdool Karim, Q. OAC0503

Abdullahi, S.J. OAB0404

Abdulwahab, M. OAB0404

Abi Haidar, Y. OAM0104

Absi, T. OAA0404

Abubakar, A. OAC0402

Acheampong, N.A. OAD0204

Achiche, S. OAD0602

Achieng Ombajo, L. OAB0202, OAS0105LB

Acuña, R. OAD0302, OAD0402

Adam, A. OAD0202

Adamczyk, A. OAA0505

Addo Boateng, F. OAB0202, OAS0105LB

Aderounmu, B. OAE0203

Adewole, D. OAC0203

Adiema, L. OAE0605

Adula, V. OAE0202

Agbaje, A. OAE0604

Agboola, P. OAE0203

Agins, B. OAD0306LB

Agwu, A. OAC0503

Ahimbisibwe, G.M. OAB0302

Ahmad, T. OAE0503

Ainembabazi, B. OAM0102

Ajayi, E. OAE0604

Akabwai, G. OAS0104LB

Akyoo, W. OAC0106LB

Al‐Abdallat, R. OAM0104

Al‐Falasi, H. OAM0104

Al‐Hadid, Y. OAM0104

Al‐Harbi, L. OAM0104

Al‐Hashmi, F. OAM0104

Al‐Ketbi, A. OAM0104

Al‐Khalili, S. OAM0104

Al‐Mansoori, K. OAM0104

Al‐Mazrouei, S. OAM0104

Al‐Neyadi, O. OAM0104

Al‐Otaibi, Z. OAM0104

Al‐Qahtani, B. OAM0104

Al‐Qudah, L. OAM0104

Al‐Rashid, N. OAM0104

Alavian, N. OAA0104

Albano, J. OAB0402

Alcide Jean‐Pierre, M.C. OAE0404

Aldrovandi, G. OAA0405

Alfeld, M. OAA0403

Alitsi, A. OAD0305

Allam, R. OAB0505

Alli, O. OAB0206LB

Allinder, S. OAD0306LB

Alrubayyi, A. OAA0302

Amamilo, I. OAE0405

Amstutz, A. OAB0503

Ancho, A. OAD0103

Anderson, K. OAB0504

Ankrah, E.O. OAD0204

Ankunda, V. OAB0202, OAS0105LB

Annequin, M. OAD0505

Anoke, C. OAB0404

Anugulruengkitt, S. OAC0503

Aparna, B. OAE0103

Apollo, T. OAE0602

Apollon, A. OAA0406LB

Apoto, N. OAS0104LB

Archary, M. OAB0302, OAS0104LB

Archin, N. OAA0104

Arif, M.S. OAA0402

Ario, A.R. OAM0102

Aristegui, I. OAD0302, OAD0402

Arnold, H. OAD0303

Arora, P. OAC0503, OAC0505

Artenie, A. OAS0103LB

Asaad, S. OAE0603

Asiimwe, M. OAC0102

Attada, S. OAB0505

Atteh Omolade, R. OAB0404

Aurora, T. OAD0603

Aviles Guaman, C. OAD0404

Ayakaka, I. OAB0503, OAB0504, OAE0302

Ayer, A. OAD0404

Azebah, N. OAD0304

Azwa, I. OAD0604

### B

Bärnighausen, T. OAC0106LB

Bagnan, L. OAB0504

Bahemuka, U. OAB0202, OAS0105LB

Bailin, S. OAA0404

Baiyegunhi, O.O. OAA0403

Bakary, D. OAE0402

Bakka, W. OAB0506LB

Balhan, L. OAD0505

Balla, S. OAB0306LB

Ballif, M. OAB0504

Bamford, A. OAS0104LB

Banda, A. OAE0202

Bandason, T. OAB0304

Bao, A. OAD0504

Baptiste, S. OAE0502

Baquero, L. OAA0505

Bar, K. OAA0203

Barakat, L. OAM0104

Barnabas, R.V. OAC0406LB

Barnhart, D. OAC0106LB

Baron, H.C. OAD0203

Barr, M. OAA0303

Barrett, B. OAD0106LB

Baveja, A. OAD0603

Bavinton, B.R. OAC0405

Beckey, K. OAB0102

Becquet, V. OAC0105

Behuhuma, O. OAB0302

Bekker, L.‐G. OAC0403, OAC0502, OAC0503, OAC0505, OAE0406LB

Belaunzaran‐Zamudio, P. OAB0206LB

Belec, L. OAC0103

Benalycherif, A. OAB0103

Bennet, J. OAB0206LB

Berardi, J. OAA0202

Bere, T. OAD0106LB

Berenger, C. OAD0505

Berry, M. OAA0306LB

Bershteyn, A. OAC0302, OAC0303

Best, J. OAA0203

Beyrer, C. OAD0503, OAS0103LB

Bhamidipati, K. OAC0302, OAC0303

Bhattacharyya, S. OAA0302

Bidi, L. OAC0404

Bilbilis, K. OAB0203

Birchard, R. OAD0306LB

Bitnun, A. OAA0103

Blanquart, L. OAD0505

Boily, M.‐C. OAS0103LB

Bondarchuk, C. OAE0406LB

Bortella, N. OAB0403

Borthwick, N. OAA0305

Bosman, S. OAE0302

Bosquet‐Dereuddre, N. OAM0105

Bossevot, L. OAM0105

Bouic, P. OAA0502

Boulware, D.R. OAB0502, OAB0506LB

Bourrelly, M. OAD0403, OAD0505

Bowring, A. OAC0602

Boyer, S. OAE0402

Bozinovski, J. OAE0502

Braun, D.L. OAB0102

Breton, G. OAA0202

Brophy, J. OAA0103

Brown, K. OAB0402

Brown, L.B. OAC0502, OAC0503, OAC0504

Browne, S. OAA0502

Brua, C. OAM0105

Brunet, L. OAB0104

Brust, J. OAB0504

Buchbinder, S. OAS0106LB

Buczkowska, M. OAD0405

Bukusi, E. OAE0605

Bunders, M.J. OAA0403

Burg, E. OAA0204

Burger, D. OAB0305, OAS0104LB

Burke, R.M. OAB0503

Buthelezi, S. OAB0306LB

Bwakura Dangarembizi, M. OAS0104LB

### C

Caballero, R. OAD0402

Cadri, A. OAD0602

Cahn, P. OAD0302

Cai, Y. OAA0205

Callander, D. OAD0202

Cameron, P.U. OAA0105

Canagasabey, D. OAC0304

Canepa, G. OAA0506LB

Cao, W. OAA0304

Capparelli, E. OAC0206LB

Caraco, Y. OAS0106LB

Cardozo, N. OAD0302, OAD0402

Carnathan, D.G. OAA0302

Carneal‐Frazer, N. OAB0402

Carr, S. OAA0104

Carter, C.C. OAC0502, OAC0503, OAC0504

Caskey, M. OAA0202

Cavarelli, M. OAM0105

Cavaro, F. OAD0403

Cazaubon, Y. OAB0306LB

Cesar, C. OAD0402

Chabala, C. OAB0305

Chabata, S.T. OAD0606LB

Chaisopa, P. OAE0504

Chan, C. OAC0405

Chanda, G. OAE0403

Chandiwana, N. OAE0406LB

Chang, J.J. OAA0105

Chang, W. OAA0106LB

Charnley, G. OAD0405

Chaturbhuj, D.N. OAA0306LB

Chauhan, A. OAD0603

Chava, N. OAB0505

Chebet, J.J. OAC0106LB

Chen, Y. OAA0304, OAA0306LB

Chetty, T. OAB0306LB

Chibanda, D. OAD0106LB

Chidarikire, T. OAC0303

Chidembo, M. OAB0304

Chikanya, W. OAE0202

Chilikutali, L. OAE0303

Chingombe, B. OAD0606LB

Chitsamatanga, M. OAS0104LB

Christian, C. OAD0404

Chukwu, E. OAE0604

Church, L.W.P. OAA0303

Cicconi, P. OAA0305

Ciglenecki, I. OAD0202

Ciko, Z. OAD0603

Cissé, M. OAD0403

Clarence, E. OAB0306LB

Clark, M. OAA0306LB

Clerc, I. OAA0402

Cobourne, M. OAC0202

Cochran, Q. OAB0104

Cockerham, L. OAA0105

Coffie, P.A. OAC0105

Cohen, C. OAB0204

Cohen, M. OAB0205

Colby, D.J. OAD0504

Collins, S. OAB0106LB

Collins, S.E. OAA0205

Compagnucci, A. OAB0305

Conaway, M. OAB0502

Cook, T. OAB0402

Cooney, E. OAD0503

Cooper, S. OAD0606LB, OAE0606LB

Cose, S. OAA0503

Costa, M. OAD0505

Cotton, M. OAB0302

Cotton, M.F. OAA0502

Cox, S. OAC0502

Crawford, G. OAC0305

Cressey, T.R. OAB0305

Cresswell, F. OAB0202

Cresswell, F.V. OAS0105LB

Cromer, D. OAA0203

Cronin, S. OAA0504

Cross, S. OAD0106LB

Crowther, C. OAB0306LB

Cruces, L. OAA0505

Cruz Molina, N. OAA0505

Cunningham, D.L. OAB0102

### D

D'Alessandro, U. OAE0402

Dahn, M. OAE0503

Dalal, S. OAC0602

Dam, A. OAC0202

Dame, J. OAB0504

Daniels, B. OAB0306LB

Darji, D.N.K. OAB0406LB

Das, C. OAB0505

Das, M. OAC0502, OAC0503, OAC0505

Dassaye, R. OAB0306LB

Daubert, E. OAB0205

Davenport, M. OAA0203

David Gbado, P. OAB0404

Davies, C. OAA0502

Davies, N. OAC0605

Day, S. OAD0203

de Nooy, A. OAC0302, OAC0303

de Ruiter, A. OAB0402, OAD0503

De Smet, E. OAA0204

de Vos, M. OAE0502

Deaton, C. OAC0505

Debroy, P. OAB0206LB

Decroo, T. OAE0302

Dedocoton, R.‐M. OAC0105

Deeks, S. OAA0504

Deeks, S.G. OAA0105

Delache, B. OAM0105

Dembélé Keita, B. OAD0403

Denoeud, L. OAE0303

DeStefanis, T. OAA0203

Dezanet, L. OAC0204

Diallo, A. OAB0103

Diallo, D.D. OAC0505

Diallo, F. OAD0403

Diallo, I. OAB0103

Diallo, Z. OAB0103

Diarra, Z. OAD0403

Dibba, B. OAE0402

Diero, L. OAB0504

Dietz, C.A. OAB0102

Dimitrov, D. OAC0205

Dine, R. OAC0203

Dioukhane, E.M. OAE0503

Dispinseri, S. OAB0306LB

Dlamini, M. OAC0104

Dlamini, T. OAC0104

Do Quang, K. OAD0504

Do, L.A. OAD0504

Doan Thi Kim, P. OAD0504

Docken, S. OAA0203

Dombojena, S. OAD0202

Dong, D. OAA0202

Dorestan, D. OAE0404

Dorward, E. OAC0402

Dourado, I. OAC0204

Doyon, G. OAA0102

Drakes, J. OAE0204LB

Drammeh, S. OAE0402

Du, Q.T. OAB0504

Duarte, M. OAD0302

Duette, G. OAA0504

Dumont, E. OAA0406LB

Dunbar, M. OAB0204

Dupnik, K. OAA0406LB

Dupreez, M. OAE0405

Duran, A. OAD0302

Dzavakwa, N.V. OAB0304

### E

Eakle, R. OAC0402

Eaton, A. OAA0303, OAA0306LB

Ediau, M. OAD0103

Edwards, R.J. OAA0306LB

Effiong, F. OAC0203

Efobi, L. OAD0104

Eholié, S. OAC0105

Eholié, S.P. OAB0103

Ekpor, E. OAC0203

El‐Gazzar, S. OAM0104

El‐Hadidy, Y. OAM0104

El‐Khoury, N. OAM0104

El‐Shenawy, N. OAM0104

Elion, R. OAB0204

Ellis, J. OAB0506LB

Enane, L. OAB0504

Engelmann, F.A. OAA0402

Enock, M. OAD0306LB

Erlandson, K. OAB0206LB

Eron, J. OAA0205, OAB0204

Esen, M. OAB0403

Eshun‐Wilsonova, I. OAB0202, OAS0105LB

Essack, Z. OAD0105

Esser, S. OAB0203

Eustaquio, P. OAC0405

Euvrard, J. OAB0504

Evans, B. OAS0106LB

### F

Fabian, S. OAD0302, OAD0402

Facente, S. OAC0502

Fadiga, F. OAB0103

Fairhead, C. OAB0106LB

Fang, Z. OAA0304

Faye, A. OAD0505

Fayorsey, R. OAE0306LB

Fernandez, N. OAA0305

Ferrand, R.A. OAB0304

Ferrari, G. OAA0104

Ferraris, O. OAM0105

Ferreira, A.‐M. OAA0205

Ferreira, O. OAC0204

Ferrier‐Rembert, A. OAM0105

Fielding, K. OAB0506LB

Figueroa‐Romero, A. OAB0403

Figueroa, M.I. OAD0302, OAD0402

Filiatreau, L.M. OAD0105

Fink, V. OAD0302, OAD0402

Fiorentino, M. OAD0403, OAD0505

Fisher, K. OAA0504

Fitzgerald, D. OAA0406LB

Flomo, J. OAE0503

Ford, D. OAB0302, OAS0104LB

Fouda, G. OAA0306LB

Fougère, Y. OAA0103

Frade, S. OAD0606LB, OAE0606LB

Franks, J. OAE0306LB

French, A. OAB0205

Frontini, E. OAD0402

Fumagalli, M. OAA0202

Fusco, G.P. OAB0104

Fusco, J.S. OAB0104

### G

Gabillard, D. OAB0103

Gabot, A. OAD0405

Gabriel, C. OAA0404

Gachiri, R. OAE0106LB

Gaiha, G.D. OAA0302

Gakuru, J. OAB0506LB

Gallardo‐Cartagena, J.A. OAC0503

Gallouët, A.‐S. OAM0105

Gandhi, M. OAC0106LB

Ganesh, S. OAB0306LB

Ganguli, P. OAD0106LB

Gao, F. OAA0304

Garcia, J.V. OAA0102

Garg, H. OAA0203

Garrett, C. OAD0205

Garrett, N. OAB0202, OAS0105LB

Garrison, L. OAE0605

Gaspar, I. OAS0102LB

Gaur, A. OAC0503

Gausi, B. OAB0405

Gautam, K. OAD0604

Genade, L. OAE0304

George, P. OAE0503

Gerber, F. OAB0503

Gerlo, S. OAA0204

Getz, M. OAA0302

Giaquinto, C. OAB0302, OAB0305, OAS0104LB

Gibb, D. OAB0305

Gibb, D.M. OAB0302

Gibson, B. OAE0503

Gill, K. OAC0503

Gils, T. OAE0302

Glass, T.R. OAB0503

Glesby, M.J. OAA0202

Glidden, D.V. OAC0502

Godoe, J. OAE0503

Goga, A. OAB0306LB

Goldberg, B. OAA0302

Goldsmith, K. OAD0106LB

Golub, J.E. OAE0304

Gonsalves, F. OAD0603

González, R. OAB0403

Gonzalez Polo, V. OAA0505

Gonzalez, A. OAS0106LB

Govathson, C. OAD0606LB

Goyal, K. OAD0603

Goymann, H. OAC0106LB

Grandhi, A. OAS0106LB

Granek, J. OAA0104

Grangeiro, A. OAC0204

Granger, K. OAC0304

Greaves, W. OAB0102

Grebe, E. OAC0502

Greco, D. OAC0204

Green, A. OAS0104LB

Green, K. OAC0304

Grint, D. OAB0202, OAS0105LB

Groenewald, C. OAD0105

Gros, W. OAM0105

Gruber, J. OAB0204

Guadarrama, A. OAD0202

Gudyanga, D. OAD0106LB

Guignard, A. OAD0503

Guindo, M.A. OAD0403

Gulick, R. OAB0206LB

Gwanzura, C. OAE0602

### H

Haberer, J. OAE0605

Hagins, D.P. OAB0102

Hahn, J.A. OAD0103

Hahn, W.O. OAA0303

Haimbe, P. OAE0204LB

Hale, G. OAB0506LB

Hallett, J. OAC0305

Hanevik, K. OAA0503

Hanke, T. OAA0305

Hankemeier, T. OAA0403

Harkoo, I. OAC0505

Harling, G. OAC0106LB

Harms, A. OAA0403

Harrison, T.S. OAB0506LB

Hartogensis, W. OAA0105

Hasson, J.M. OAA0402

Hathaway, C.L. OAC0406LB

Hawkes, M.T. OAA0103

Hayes, P.J. OAA0305

Haynes, B.F. OAA0303

Hecht, F.M. OAA0105

Heitner, J. OAC0406LB

Hemelaar, J. OAB0406LB

Herate, C. OAM0105

Herbert, C. OAA0405

Hernandez, R. OAB0205

Hewa, M. OAE0605

Heysell, S. OAB0502

Hill, A. OAB0106LB

Hinojosa, J.C. OAC0504

Hirt, D. OAB0305

Hoege, D. OAD0306LB

Hoffmann, C.J. OAE0304

Hofmann, E. OAB0102

Holden, L. OAB0302

Holmes, C. OAD0306LB

Holmes, S. OAA0306LB

Homony, B. OAS0106LB

Hope, T. OAA0402

Hsu, R. OAB0104

Huang, H. OAA0302

Hudson, M. OAD0404

Hui, E.E. OAD0604

Hutchings, G. OAD0405

Hyrien, O. OAA0303

### I

Idahosa, A. OAB0202, OAS0105LB

Ilori, T. OAB0404

Imamichi, H. OAA0106LB

Imamichi, T. OAA0106LB

Imperial, M. OAC0505

Innes, S. OAA0502

Irvine, D. OAA0302

Issah, C. OAB0405

Ito, Y. OAM0104

Iwuji, C. OAD0405

### J

Jabbour, T. OAM0104

Jafta, L. OAS0104LB

Jahn, A. OAC0106LB

Jain, A. OAA0506LB

Jallow, A.E. OAE0402, OAE0402

Jammeh, K. OAE0402

Janamnuaysook, R. OAC0205

Janla, S. OAE0504

Jaravani, D. OAE0606LB

Jarjaval, F. OAM0105

Jarvis, J.N. OAB0506LB

Jauregui, J.C. OAD0102

Jaygbeh, A. OAE0503

Jeenarain, N. OAB0306LB

Jennings, A. OAS0104LB

Jeon, E.Y. OAA0302

Jiang, X. OAA0304

Jin, W. OAA0203

Jjemba, E. OAE0104

Johnson, C.C. OAC0302, OAC0303

Johnson, D. OAB0205

Jones, R.B. OAA0202

Jopling, R. OAD0106LB

Jordan, M.R. OAC0205

Joseph Davey, D. OAD0105

Joseph, Y. OAA0406LB

### K

Königs, C. OAB0302

Kachepatsonga, H. OAE0502

Kadobera, D. OAM0102

Kafeero, P. OAB0202, OAS0105LB

Kagimu, E. OAB0506LB

Kakande, A. OAS0105LB

Kakkar, F. OAA0103

Kalitera, L. OAE0303

Kamele, M. OAE0302

Kameswara Prasad, Y. OAE0103

Kamgwira, Y. OAD0306LB

Kamiri Thuo, S. OAE0106LB

Kamissoko, A. OAD0403

Kamori, D. OAC0106LB

Kandolsky, M. OAD0502

Kangogo, G. OAD0406LB

Kantor, A. OAB0206LB

Kanyinji, R. OAE0403

Kapoor, Y. OAS0106LB

Kasale, D. OAD0306LB

Kassim, S. OAB0202, OAS0105LB

Katlama, C. OAB0102

Katumba, H. OAM0102

Kawalazira, G. OAD0306LB

Kayemba, H.D. OAE0104

Keitseng, M. OAE0302

Kekitiinwa, A. OAS0104LB

Kekitiinwa, A.R. OAB0302

Kelleher, A. OAA0504

Kelley, C. OAC0502

Kelley, C.F. OAA0303

Kelly, M. OAA0203

Kelman, I. OAD0405

Kembo, D. OAB0304

Khabala, K. OAD0202

Khalil, O. OAM0104

Khalil, Z. OAM0104

Khalsa, S. OAS0106LB

Khan, P.Y. OAB0304

Khandu, L. OAC0305

Khetan, S. OAS0106LB

Khomkaew, K. OAE0504

Kiene, S.M. OAD0103

Kigozi, E. OAB0506LB

Kigozi, G. OAC0505

Kiilu, C. OAS0104LB

Kikobye, P. OAC0304

Kilonzo, S. OAE0205LB

Kim, H.‐Y. OAC0302, OAC0303

Kimbugwe, G. OAS0105LB

Kimmel, A. OAC0202, OAC0402

Kimuda, S. OAB0506LB

Kinikar, A. OAB0504

Kintu, A. OAC0502, OAC0503, OAC0505

Kipkoech, K. OAS0103LB

Kirongo, D. OAD0202

Kirui, E. OAC0302

Kisia, C. OAC0302

Kissi, E. OAC0105

Kitabu, J. OAE0402

Kithinji, C. OAD0202

Kitonsa, J. OAS0105LB

Kityo, C. OAS0104LB

Kityo, C.M. OAB0302

Klasse, P.J. OAA0306LB

Klein, S.B. OAC0406LB

Knox, J. OAC0106LB

Kobayashi, A. OAM0104

Kobugabe, S. OAE0104

Kobusinge Lubega, J. OAM0102

Kobusingye, J.O. OAM0102

Koethe, J. OAB0206LB

Koethe, J.R. OAA0404

Koile, E. OAS0105LB

Komunyena, J.T. OAC0304

Konda, K.A. OAD0102

Koote, C. OAE0104

Kotze, P. OAS0106LB

Kouanfack, C. OAB0103

Krause, R. OAA0203

Kreuter, A. OAB0203

Kuhn, L. OAA0405

Kumar, N. OAA0105

Kumar, P. OAB0505

Kumarasamy, N. OAB0105LB

Kurada, J. OAB0505

Kwena, Z. OAE0605

Kwesiga, B. OAM0102

Kwizera, P. OAM0102

Kyambadde, P. OAC0304

### L

Labbe, N.R. OAE0404

Labhardt, N.D. OAB0503

Lake, J. OAB0206LB

Laker Odongpiny, E. OAB0202

Laker Odongpiny, E.A. OAS0105LB

Lallemant, M. OAB0305

Landais, E. OAD0603

Landay, A. OAB0206LB

Landman, R. OAB0103, OAB0106LB

Lane, H.C. OAA0106LB

Langer, T. OAA0505

Langlois, S. OAM0105

Lankiewicz, E. OAE0505

Larmarange, J. OAC0105

Lauer, K. OAE0502

Laufer, N. OAA0505

Lawrence, D.S. OAS0105LB

Le Grand, R. OAM0105

León‐Morris, F. OAD0102

Le, T.C. OAD0504

Leavy, J.E. OAC0305

Lebelo, K. OAC0403, OAE0406LB

Lebina, L. OAE0304

Lebouché, B. OAD0602

Lechemia, M. OAM0105

Ledenski, L. OAD0202

Lee, E. OAA0504

Lee, J. OAD0106LB

Leicaj, L. OAA0505

Lemoine, M. OAE0402

Leonard, M. OAB0206LB

Lespinasse, D. OAA0406LB

Letscher, H. OAM0105

Levis, B. OAB0104

Lewin, S.R. OAA0105

Leyritana, K. OAC0405

Li, F. OAA0405

Li, H. OAA0304

Li, K. OAD0406LB

Li, M. OAA0304

Li, X. OAD0205

Li, Z. OAD0205

Liberatus, P. OAE0505

Likumbo, S. OAD0306LB

Lim, D. OAA0302

Lin, G. OAB0102

Linda, L. OAC0102

Liou, M.‐L. OAA0506LB

Little, S.J. OAA0205

Liu, Y. OAA0304

Lockman, S. OAC0206LB

Logue, E. OAA0202

Lombaard, J. OAS0106LB

Longwe, B. OAE0204LB

Lorenzo‐Redondo, R. OAA0402

Loufty, M. OAD0302

Louw, C.E. OAC0505

Loux, T. OAD0406LB

LoVullo, C. OAC0402

Luba, M. OAE0202

Lucas, J. OAD0303

Ludot, C. OAM0105

Lugemwa, A. OAB0302

Lukau, B. OAB0503

Lungu, G. OAB0303

Luong, B.Q. OAD0504

Luque, M.T. OAB0504

Lyerly, A.D. OAD0203

Lynch, R. OAA0203

Lynen, L. OAE0302

### M

Ma, Y. OAD0602

Macdonald, P. OAC0403, OAE0406LB

Machado, D.M. OAB0504

Machekano, R. OAE0303

Machira, Y.W. OAD0603

Mackworth‐Young, C. OAB0304

MacPherson, P. OAB0503

MacWilliam, J. OAE0202

Madonsela, T. OAE0302

Magaji, S. OAB0404

Magare, J.O. OAC0302

Magno, L. OAC0204

Mahawan, L. OAE0504

Mahgoub, H.M.E. OAE0603

Mahlatsi, P. OAB0503

Mahomed, S. OAB0202, OAS0105LB

Maila, B. OAB0303

Maimela, M. OAA0403

Maiorino, L. OAA0302

Majam, M. OAC0303

Makamba, M. OAB0304

Makhema, J. OAC0206LB

Makkan, H. OAD0603

Makobu, K. OAD0202

Makoni, T. OAD0404

Malahleha, M. OAC0505

Malakhova, M. OAD0605

Malunga, S. OAE0202

Mambo, B. OAC0302

Manalu, S. OAB0106LB

Mandara, E. OAE0405

Mande, E. OAB0506LB

Manentsa, M. OAC0503

Mangezi, W. OAD0106LB

Mann, J. OAA0403

Mansoor, Z. OAE0505

Mansouri, K. OAA0306LB

Maphosa, T. OAE0303

Mapukata, P. OAC0403, OAE0406LB

Maradan, G. OAD0403, OAD0505

Marake, N. OAE0405

Marake, N.B. OAB0503

Marcy, O. OAB0504

Maresca, A.F. OAD0505

Margolis, D. OAA0104

Maricich, N. OAD0606LB, OAE0606LB

Marin‐Rojas, J. OAA0504

Marouniak, O. OAD0605

Marques, L. OAB0302

Marquez, S. OAD0106LB

Marsh, A. OAC0303

Martin‐Hughes, R. OAC0602, OAS0102LB

Martin, N.K. OAS0102LB

Martindale, L. OAC0402

Martinez, E. OAB0106LB

Martinson, N. OAE0304

Maseko, M. OAC0104

Masese, R.V. OAD0203

Mashayekhi, M. OAA0404

Masheto, G. OAC0206LB

Matambanadzo, P. OAD0606LB

Matare, T. OAE0602

Matiya, E. OAE0303

Matovu Kiweewa, F. OAC0502, OAC0505

Matovu, N. OAE0305

Matrajt, L. OAC0205

Matsuda, K. OAM0104

Matthews, R.P. OAB0102, OAS0106LB

Maulidi, M. OAC0402

Mavudze, J. OAC0404

Mayer, K. OAS0106LB

Mayi, A. OAE0303

Mazibuko, L. OAA0403

Mazuze, M. OAB0403

Mbabazi, A. OAC0102

Mbambara, T. OAB0303

Mbasa, M. OAD0206LB

Mbewe, M. OAB0303

McComsey, G. OAB0204

Mcfarland, A. OAA0203

McMichael, A. OAA0102

McRaven, M.D. OAA0402

Mdolo, A. OAB0405

Melo, M. OAA0302

Mendes Muxlhanga, A. OAB0403

Mendes, W.B. OAA0105

Mendonca, R. OAD0606LB, OAE0606LB

Menendez, C. OAB0403

Mesplede, T. OAC0205

Meya, D.B. OAB0506LB

Mezzio, D. OAC0504

Mfitundinda, E. OAM0102

Michard, F. OAD0505

Michels, D. OAD0505

Migisha, R. OAM0102

Milaham, M. OAE0405

Milanga, M. OAE0505

Millard, K. OAA0202

Miller, A.P. OAD0103, OAD0105

Miller, M. OAD0503

Min Han, W. OAB0206LB

Minh, N.T. OAD0504

Mirembe, B. OAC0304

Mischlinger, J. OAB0403

Misra, S. OAE0302

Mjwara, G. OAB0306LB

Mkhatshwa, H. OAC0104

Mkhize, N.N. OAB0306LB

Mngadi, K. OAC0504

Mngqbisa, R. OAS0104LB

Mnkandla, D. OAE0502

Mofenson, L. OAB0402

Moga, T. OAC0404

Moh, R. OAB0103

Mohamed, M. OAA0403

Mohammed, T. OAC0206LB

Mohoanyane, M. OAE0405

Moiana Uetela, D. OAS0102LB

Molapo, M. OAE0405

Molatelle, M. OAB0503

Moles, J.P. OAB0306LB

Molina, J.‐M. OAB0102

Mombo‐Ngoma, G. OAB0403

Momper, J. OAC0206LB

Monroe‐Wise, A. OAC0302

Montefiori, D. OAA0303

Montefiori, D.C. OAA0306LB

Moodley, D. OAC0505

Moonga, G. OAB0303

Moore, C.C. OAB0502

Moore, D.A.J. OAB0506LB

Moore, P.L. OAB0306LB

Mora, M. OAD0403, OAD0505

Morack, R. OAB0205

Moran, L. OAB0206LB

Moran, P. OAA0105

Morebotsane, S. OAE0405

Moremi, N. OAE0606LB

Morris, S. OAD0606LB, OAE0606LB

Morroni, C. OAC0206LB

Moses, E. OAD0306LB

Mosha, I. OAC0106LB

Mounzer, K. OAC0504

Moyle, G. OAB0204

Moyo, C. OAB0303

Moyo, L. OAB0303

Moyo, S. OAC0206LB

Mpagama, S. OAB0502

Mpembeni, R. OAC0106LB

Mseu, D. OAE0502

Muchara, A. OAC0402

Mugabi, T. OAB0506LB

Mugerwa, H. OAS0104LB, OAS0105LB

Mugisa, B. OAC0605

Mugo, N.R. OAC0406LB

Mugurungi, O. OAC0404

Muhumuza, U. OAE0105, OAM0103

Mujuru, H.A. OAB0302

Mujuru, P. OAE0602

Mukherjee, J. OAD0603

Mukherjee, T. OAC0202, OAC0402

Mukondwa, R.W. OAD0404

Mukuwapasi, W. OAD0404

Muleia, R. OAS0102LB

Mulenga, A. OAE0403

Mulenga, L. OAE0204LB

Mulenga, M. OAE0204LB

Mulungu, C. OAB0303

Munemo, D. OAB0304

Munjoma, M. OAC0404

Mupanguri, C. OAE0602

Murungu, J. OAD0306LB

Musa, T.F. OAE0503

Musemburi, S. OAD0606LB

Musonda, M. OAC0402

Mutale, K. OAE0202

Mutede, B. OAC0404

Mutesi, C. OAM0102

Mutongore, M. OAE0505

Muwonga Tukisadila, J. OAC0103

Muyindike, W. OAB0504

Muzoora, C. OAB0502, OAB0506LB

Mwamba, J. OAB0303

Mwambi, H. OAA0403

Mwamelo, A. OAE0204LB

Mwangomale, A. OAE0202

Mwansambo, A. OAB0405

Mwendar, G. OAD0202

Mwenya, O. OAB0303

Mwilu, R. OAE0403

Mworeko, L. OAC0505

### N

Na‐Rajsima, S. OAB0302

Nabaggala, P. OAS0105LB

Nabbuto, J. OAS0105LB

Nacimento, A.V. OAA0302

Nadol, P. OAB0505

Nahumuza, Z. OAM0103

Naidoo, K. OAA0403

Naidoo, L. OAB0306LB

Naidoo, M. OAC0503

Naigino, R. OAD0103

Nakabuye, S. OAS0104LB

Nakamura, K. OAM0104

Nalwanga, J. OAM0102

Namakula, L.O. OAM0102

Namakula, R. OAA0503

Namombwe, S. OAB0506LB

Namugga, O. OAA0503

Namusisi, A.M. OAM0102

Nankabirwa, V. OAA0503

Naqvi, N. OAC0402

Nardone, A. OAB0305

Nassar, K. OAM0104

Nath, R. OAD0102

Nathan, A. OAA0302

Nathanson, S. OAA0205

Nattere, D. OAA0104

Naturinda, M. OAM0103

Ncube, G. OAC0404

Ndlangamandla, Q. OAB0306LB

Ndow, G. OAE0402, OAE0402

Ndung'u, T. OAA0403

Ndyetukira, J.F. OAB0506LB

Nelson, A. OAA0306LB

Netea, M.G. OAA0503

Ngandu, N.K. OAB0306LB

Ngosa, D. OAB0303

Nguyen, Q.D. OAB0504

Nguyen, V.K. OAC0106LB

Ngwenya, A. OAE0202

Nhampossa, T. OAB0403

Niangoran, S. OAB0103

Nicholaus, A. OAE0505

Nichols, B.E. OAC0302, OAC0303

Nicola, A. OAA0202

Njuguna, C. OAD0506LB

Njwayo, A.J. OAC0106LB

Nkuranga, J. OAS0105LB

Nliwasa, M. OAB0503

Nonyane, B.A.S. OAE0304

Noppe, Y. OAA0204

Norcross, C. OAB0202, OAS0105LB

Norris, S. OAB0406LB

Nouaman, M. OAC0105

Nsangi, L.J. OAB0506LB

Ntainyane, L. OAE0405

Nthenge, K. OAA0404

Null, M. OAB0502

Nussenzweig, M. OAA0202

Nuwagira, E. OAB0502

Nxumalo, H. OAC0104

Nyagah, W. OAC0603

Nyamayaro, P. OAD0106LB

Nyirenda, R. OAE0303

Nyundu, E.K. OAE0403

### O

O'Cleirigh, C. OAD0106LB

O'Connor, C. OAC0605

O'Halloran, J. OAB0206LB

Obare, L.M. OAA0404

Oberth, G. OAE0502

Ochanda, R.M. OAE0202

Ochiel, A. OAE0605

Ochwal, P. OAE0605

Odhiambo, F. OAB0504

Odoyo, J. OAE0605

Odume, B. OAE0604

Oette, M. OAB0203

Ogbuagu, O. OAC0502

Ogundare, O. OAB0404

Oguttu, B. OAC0206LB

Ogwang, C. OAS0105LB

Ohuma, E. OAB0406LB

Okiror Okello, E. OAM0102

Okochi, H. OAC0106LB

Okoye, A. OAA0205

Olatosi, B. OAD0205

Olawuyi, D. OAC0203

Olete, R.A. OAC0405

Oliveira Leite, B. OAC0204

Olugbenga, D. OAE0604

Omolaja, A. OAE0604

Ondeng'e, K. OAD0603

Ong, J. OAC0405

Onono, M. OAC0406LB

Onyango, A. OAS0105LB

Orellano, G. OAD0302

Osatuyi, T. OAE0604

Osegueda Peña, A.A. OAA0505

Osiyemi, O.O. OAB0102

Ostrowski, M. OAA0505

Otwombe, K. OAA0502

Ouamba, J.P. OAD0202

Oware, K. OAE0605

Owarwo, N. OAB0202

Owarwo, N.C. OAS0105LB

Owira, P. OAD0202

### P

Pacheco Mendez, J. OAA0205

Palanee‐Phillips, T. OAC0505

Palee, P. OAE0504

Palmer, S. OAA0504

Panchia, R. OAC0505

Panduranga Reddy, A. OAB0105LB

Panizoni, E. OAD0302, OAD0402

Pape, J. OAA0406LB

Paradza, M. OAB0304

Parks, R. OAA0303

Parthasaradhi Reddy, B. OAB0105LB

Pascal, Q. OAM0105

Pascoe, S.P. OAA0402

Pastrana, M. OAD0202

Patel, F. OAA0405

Patpeerapong, P. OAE0504

Pattamukkil, A. OAE0304

Paudel, K. OAD0604

Paulo, Y. OAS0102LB

Peer, A. OAS0106LB

Perieres, L. OAE0402

Peter, C. OAE0505

Petersen, Z. OAD0105

Petrov‐Sanchez, V. OAB0103

Pett, S. OAS0104LB

Phanupak, N. OAC0405

Phanuphak, N. OAC0205, OAC0504

Phiphatkhunarnon, P. OAD0604

Phiri, C. OAE0204LB

Pike, C. OAC0403, OAE0406LB

Pinto, J.A. OAB0504

Pita, T.P. OAE0302

Plank, R.M. OAS0106LB

Plazy, M. OAC0105

Poda, A. OAB0103

Pollara, J. OAA0104

Poluru, R.P. OAB0505

Poovan, N. OAS0106LB

Poteat, T. OAD0503

Potloane, D. OAC0503

Potthoff, A. OAB0203

Pozniak, A. OAB0106LB

Prasad, K. OAB0505

Prata Menezes, N. OAB0204

Priya, K. OAE0103

Prochazka Nunez, M. OAC0604

Puryear, S. OAC0505

Puthanakit, T. OAB0302

### Q

Qiao, S. OAD0205

Qiu, J. OAA0304

### R

Radix, A. OAD0503

Radtchenko, J. OAB0204

Ragone, L. OAB0402, OAD0503

Rahal, G. OAM0104

Ramesam, G. OAB0505

Ramgi, P. OAS0102LB

Ramgopal, M. OAC0504

Ramgopal, M.N. OAB0102

Ramjeith, A. OAB0306LB

Ramraj, T. OAB0306LB

Rao, A. OAB0505

Rassool, M. OAS0106LB

Ratevosian, J. OAS0103LB

Ratnakar Reddy, K. OAB0105LB

Rawat, S. OAD0603

Rawlins, S. OAB0102

Read, S. OAA0103

Reddy, K. OAA0403

Reddy, M. OAB0306LB

Reddy, T. OAB0306LB

Redzo, N. OAB0304

Reed, E. OAD0103

Rees, K. OAC0605

Regenold, S. OAA0303

Reidy, W. OAE0306LB

Reisner, S. OAD0503

Reither, K. OAE0302

Relouzat, F. OAM0105

Resar, D. OAE0204LB

Reyes‐Diaz, M. OAD0102

Rhodes, A. OAA0105

Riddler, S. OAS0106LB

Rincon, G. OAD0505

Robertson, M.N. OAS0106LB

Roche, M. OAA0105

Rodrigues, J. OAC0202

Rodriguez, L. OAA0302

Rohr, S. OAA0306LB

Rojo, P. OAB0302

Romero, M. OAD0302

Rono, B. OAE0605

Root, H. OAB0504

Rosen, S. OAC0303

Ross, R. OAB0205

Rousseau, E. OAC0403, OAE0406LB

Rouveix, E. OAD0505

Rouzier, V. OAB0504

Rupasinghe, P.C. OAE0603

Ruzagira, E. OAB0202, OAS0105LB

### S

Saïdi, Y. OAB0302

Sabry, T. OAM0104

Sadiq, A. OAB0506LB

Safren, S. OAD0106LB

Safrit, J.T. OAA0202

Sagaon‐Teyssier, L. OAD0403

Saha, P. OAD0603

Said, B. OAB0502

Saito, S. OAE0306LB

Salomone, S. OAS0104LB

San, E.T. OAE0102

Sanchez‐Samaniego, G. OAB0503

Sandbhor, P. OAD0603

Sangtong, S. OAE0504

Santayakul, S. OAE0504

Sato, B. OAB0405

Sato, R. OAM0104

Saunders, K.O. OAA0303

Sax, P. OAB0204

Scarlatti, G. OAB0306LB

Schappe, T. OAA0104

Scheuerle, A. OAB0402

Schmidt, H.‐M.A. OAC0405, OAC0604

Schneider, J. OAD0503

Schrader, H. OAA0203

Scott, N. OAC0602, OAS0102LB

Scrimieri, F. OAA0106LB

Seaman, M.S. OAA0306LB

Seaton, K. OAA0202

Seeley, J. OAS0104LB

Sefuthi, T. OAE0405

Segal, K. OAC0603

Semphere, R. OAB0503

Sension, M. OAB0104

September, Q. OAB0306LB

Serrao, C. OAD0302

Sevene, E. OAB0403

Shaaban, M.A. OAA0402

Shacham, E. OAD0406LB

Shah, N. OAC0504

Shakwelele, H. OAE0204LB

Shambira, K. OAC0206LB

Shanaube, K. OAB0303

Shanmuganathan, R. OAE0103

Shao, Y. OAC0502

Shapiro, A.E. OAA0303, OAS0106LB

Shapiro, R. OAC0206LB

Shashidhar Reddy, D. OAB0105LB

Shava, E. OAC0206LB

Shaw, B. OAE0404

Shearer, K. OAE0304

Sheira, L. OAC0604

Shepherd, R.A. OAA0105

Sherman, B.T. OAA0106LB

Shete, P. OAD0404

Shi, Q. OAB0504

Shilagwa, S. OAE0202

Shoko, N. OAC0404

Short, W. OAB0402

Shrestha, R. OAD0604

Shubhadeep Sinha, D. OAB0105LB

Sigande, L. OAB0303

Siika, A. OAS0104LB

Silarszka, S. OAC0206LB

Sileo, K. OAD0103

Silhol, R. OAS0103LB

Silondwa, M. OAE0204LB

Silvestre, N. OAM0105

Silvestri, G. OAA0302

Simms, V. OAB0304

Simpson‐Cupido, D. OAE0606LB

Simpson, J. OAA0504

Simwanza, S. OAB0303

Sinclair, G. OAS0106LB

Singchaichit, K. OAE0504

Singh, N. OAC0505

Singh, R. OAC0503

Singh, U. OAS0105LB

Singh, Y. OAC0503, OAS0106LB

Sirisup, K. OAE0504

Sittikarn, S. OAE0504

Skataric, M. OAS0106LB

Smeaton, L. OAB0206LB

Soares, F. OAC0204

Soboyisi, M. OAE0502

Sokoya, D. OAE0604

Sommerfelt, H. OAA0503

Songo, J. OAE0303

Soudeyns, H. OAA0103

South, A. OAS0104LB

Spence, T. OAA0306LB

Spiegelman, D. OAC0106LB

Spire, B. OAD0403, OAD0505

Spyrelis, A. OAD0606LB, OAE0606LB

Sravan, T. OAB0105LB

Sreenivasa Chary, S. OAB0105LB

Sridhar, G. OAB0104

Sriramulu, C. OAB0505

Ssekindi, F. OAB0506LB

Ssonko, S.P. OAE0104

Stafford, S. OAD0606LB, OAE0606LB

Stanton, A. OAD0106LB

Steiner, L. OAE0304

Steinsland, H. OAA0503

Stephens, V.R. OAA0404

Stern, J. OAA0105

Stoeger, L. OAB0403

Stone, J. OAS0103LB

Strehlau, R. OAA0405

Stylianidou, Z. OAA0204

Su, Y.R. OAA0404

Sujan, S.H. OAD0604

Surafel, E. OAM0103

Suwanphatthana, N. OAE0504

Suzuki, H. OAM0104

### T

Taasi, G. OAC0304

Tailor, J. OAC0603, OAC0604

Takarinda, K. OAD0404

Takata, H. OAA0205

Tanaka, H. OAM0104

Tandolkar, I. OAE0202

Tang, B. OAA0502

Tapisha, B. OAB0303

Taruberekera, N. OAC0404

Tarumbiswa, T. OAB0503, OAE0405

Taung, K. OAE0102

Taylor, A.B. OAD0102

Tchouatieu, A. OAB0403

Tebas, P. OAA0202

Tegli, M. OAE0503

Telela, D. OAB0405

Tembo, A. OAC0303

ten Brink, D. OAC0602

Tendo‐Bugondo, C. OAC0304

Teni, M. OAD0406LB

Thahanyane, T. OAE0302

Thakur, P. OAB0105LB

Thaweesee, N. OAC0402

Thior, I. OAC0304

Thogarucheeti, M. OAB0505

Thomas, T. OAB0502

Thomas, Y. OAA0402

Thomason, M. OAS0104LB

Thonyiwa, V. OAC0402

Thorne, C. OAB0402

Thulo, M. OAE0405

Thuruthiyil, C. OAA0402

Tiam, A. OAE0303

Tiberi, O. OAS0102LB

Tidwell, G. OAC0402

Tiemessen, C. OAA0405

Tieosapjaroen, W. OAC0405

Timera, H. OAM0105

Tiri, C. OAC0102

Tobin, N. OAA0405

Tolazzi, M. OAB0306LB

Tomaras, G. OAA0202

Tonen‐Wolyec, S. OAC0103

Tournier, J.‐N. OAM0105

Tran, H.T. OAD0504

Traoré, F. OAD0403

Trautmann, L. OAA0205

Trejo, M.C. OAD0402

Trickey, A. OAD0405

Tripathi, S.K. OAB0105LB

Trout, K. OAD0406LB

Tshazi, A. OAE0302

Tsotako, M. OAE0405

Tuck, R. OAA0306LB

Tugume, G. OAC0102

Tuhebwe, D. OAD0103

Tukundane, A. OAB0506LB

Tumusiime, V. OAB0202, OAS0105LB

Tumwesigye, N. OAD0103

Turaka, P. OAB0505

Turkova, A. OAB0302

Turroja, M. OAA0202

Tuyishime, M. OAA0104

Twiine, A. OAC0102

Tylleskär, T. OAB0306LB

### U

Ubochioma, E. OAE0604

Udunze, O. OAE0604

ul Hadi, S. OAD0603

### V

Vaida, F. OAA0502

Van de Perre, P. OAB0306LB

van de Vijver, D. OAC0205

van der Horst, W. OAS0105LB

van der Vendt, D. OAS0105LB

van Heerden, A. OAE0302

Van Huizen, R. OAD0505

van Kampen, J. OAC0205

van Rein‐van der Horst van, W. OAB0202

van Rooyen, H. OAD0105

van Wyk, J. OAB0104

Vandekerckhove, L. OAA0204

Vannappagari, V. OAB0104, OAB0402, OAD0503

Vanobberghen, F. OAE0302

Vanto, O. OAC0403

Variava, E. OAB0302

Vasconcelos, R. OAC0504

Venkateswara Rao, P. OAE0103

Venter, F. OAB0106LB

Venter, W.D.F. OAC0303

Verde Hashim, C. OAC0604

Vesay, M. OAS0106LB

Vickerman, P. OAS0103LB

Vij, A. OAC0402

Vijayaraman, A. OAE0103

Villanueva, S. OAD0602

Villinger, F. OAA0402

Violari, A. OAB0302

Vlieghe, E. OAE0302

Vo‐Quang, E. OAE0402, OAE0402

Vujcich, D. OAC0305

### W

Walker, S. OAS0104LB

Wallach, S. OAE0306LB

Wallin, J.J. OAA0205

Walmsley, S. OAD0302

Walusiku‐Mwewa, B. OAE0403

Wambua, S. OAE0202

Wang, S. OAA0405

Wang, T. OAA0405

Wanjalla, C.N. OAA0404

Wanyenze, R. OAD0103

Ward, M. OAA0406LB

Warren, M. OAC0603

Wati, D.K. OAB0504

Webb, K. OAD0404

Webber, T. OAA0502

Weber, K. OAB0205

Wee, E.G.‐T. OAA0305

Wehbe, M. OAM0104

Wejda, M. OAA0204

Welch, S. OAB0302

Wele, A. OAB0506LB

Wenani, D. OAM0102

West, N. OAD0404

Westin, M. OAC0204

White, E. OAB0302

Wickersham, J.A. OAD0604

Wiehe, K. OAA0306LB

Wieland, U. OAB0203

Wilkin, T. OAA0202

Wilkinson, L.S. OAC0605

Williams, J. OAE0103

Williams, W. OAA0306LB

Win, K.K. OAE0102

Wirtz, A. OAD0503

Witkowski, W. OAA0204

Wohlfeiler, M. OAB0104

Wolfe, C. OAA0104

Wong, P. OAC0503, OAC0504, OAC0505

Wongsa, A. OAC0205

Wongwaiphanit, C. OAE0504

Wulan, N. OAC0602

Wyncoll, J. OAB0302

### X

Xia, H. OAD0205

Xiao, L. OAA0506LB

Xin, X. OAA0304

### Y

Yamamoto, S. OAM0104

Yang, H. OAA0102

Yang, Y. OAA0304

Yao, W. OAA0304

Yawe, I. OAB0202, OAS0105LB

Yazdanpanah, Y. OAD0505

Yeldandi, V. OAB0505

Yim, R. OAA0302

Yonaba, C. OAB0504

Yoseph, M. OAC0206LB

Yotebieng, M. OAB0504

Young, T.A. OAD0303

Yu, J. OAA0304

### Z

Zahara, N. OAE0105

Zaki, F. OAM0104

Zalazar, V. OAD0302, OAD0402

Zash, R. OAC0206LB

Zeballos, D. OAC0204

Zhang, C. OAB0402, OAS0106LB

Zhang, Q. OAA0304

Zhang, X. OAA0404

Zhang, Y. OAA0304

Zhang, Z. OAA0403

Zhao, Y. OAC0502, OAC0503

Zhou, W. OAA0304

Zimuto, N. OAC0404

Zoghby, L. OAM0104

Zumbo, P. OAA0406LB

